# Pyrazole‐Derived Antibacterial Compounds Effectively Treat Methicillin‐Resistant *Staphylococcus Aureus* Infections by Inhibiting Aspartate Transcarbamoylase

**DOI:** 10.1002/advs.76704

**Published:** 2026-07-20

**Authors:** Xiaorong Yang, Xinyuan Cao, Lixia Dai, Feng Yang, Jing Wang, Yudong Ma, Tong Bu, Zile Gong, Xueyan Liu, Changcai Bai, Xiaolou Miao, Xiaofei Shang

**Affiliations:** ^1^ Key Laboratory of New Animal Drug Project Lanzhou Institute of Husbandry and Pharmaceutical Sciences Key Laboratory of Veterinary Pharmaceutical Development of Ministry of Agriculture Chinese Academy of Agricultural Sciences Lanzhou Gansu P. R. China; ^2^ People's Hospital of Ningxia Hui Autonomous Region Ningxia Medical University Yinchuan P. R. China; ^3^ College of Pharmacy Ningxia Medical University Yinchuan P. R. China; ^4^ College of Veterinary Medicine Gansu Agricultural University Lanzhou P. R. China; ^5^ School of Pharmacy Gansu University of Chinese Medicine Lanzhou P. R. China

**Keywords:** aspartate transcarbamoylase, biofilm eradication, methicillin‐resistant *Staphylococcus aureus*, pyrazole‐amide derivative

## Abstract

The persistent threat of methicillin‐resistant *Staphylococcus aureus* (MRSA) underscores the critical need for antimicrobials with novel mechanisms of action. Herein, we describe the discovery of Py‐27, a pyrazole‐derived compound exhibiting potent bactericidal activity against MRSA. A chemoproteomic approach identified aspartate transcarbamoylase (ATCase), a key enzyme in *de novo* pyrimidine synthesis, as the specific target of Py‐27. Biophysical and enzymatic assays confirmed its high‐affinity binding and inhibition of ATCase, while genetic knockout established ATCase as a druggable metabolic vulnerability in MRSA. Mechanistically, Py‐27 disrupts pyrimidine synthesis, thereby impairing DNA replication, perturbing metabolic homeostasis, and ultimately inducing lethal oxidative damage. Notably, Py‐27 eradicates mature biofilms while exhibiting a low resistance propensity and favorable toxicological profiles. In animal infection models, Py‐27 demonstrated superior in vivo efficacy compared to vancomycin and promoted infection resolution and tissue repair. This study demonstrates the potential of pyrazole‐amide compounds targeting ATCase for the treatment of MRSA infections.

## Graphical abstract

This study unveils a novel antibacterial mechanism against MRSA, in which the compound Py‐27 selectively targets and inhibits aspartate transcarbamoylase (ATCase), a key enzyme in pyrimidine synthesis. This inhibition disrupts DNA replication, induces oxidative damage, leading to potent bactericidal activity. The findings highlight ATCase as a druggable target and support pyrazole‐amide compounds as a promising therapeutic strategy against MRSA infections.

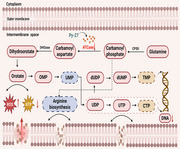

## 1 Introduction

Antimicrobial resistance (AMR) in bacteria has emerged as one of the major global challenges. It is estimated that by 2050, AMR could directly cause 1.91 million deaths and indirectly lead to nearly 10 million deaths, representing an annual increase of approximately 70% compared with 2022 [1]. Recently, the World Health Organization (WHO) updated the list of priority bacterial pathogens [2]. Among high‐priority pathogens, methicillin‐resistant *Staphylococcus aureus* (MRSA) remains a common “hard‐to‐treat” bacterium in healthcare systems and a major threat in healthcare‐associated infections. Their persistent, recurrent infections and multidrug resistance pose severe public health challenges [3]. Globally, MRSA has shown the largest increase in mortality, directly causing 130 000 deaths in 2021, more than double the 57 200 deaths recorded in 1990 [4]. While antibiotics such as vancomycin, linezolid, teicoplanin, and daptomycin remain the mainstay of clinical treatment for MRSA infections, the emergence of resistant strains has progressively eroded their efficacy [5, 6, 7]. Compounding this issue is the stagnation in the development of novel antibacterial agents [8]. Therefore, there is an urgent need to develop new antibacterial drugs with novel targets and antimicrobial mechanisms to combat MRSA infections.

Pyrazole, an important class of nitrogen‐containing five‐membered heterocyclic compounds, features a diunsaturated five‐membered ring with two nitrogen atoms at positions 1 and 2. Owing to its unique chemical structure, pyrazole exhibits a broad spectrum of biological activities [9, 10], including antibacterial, anti‐inflammatory, antiviral, and anticancer properties, garnering significant attention in organic chemistry, medicinal chemistry, and drug discovery [11, 12, 13]. Given its structural versatility and promising antibacterial potential, the development of pyrazole‐based antimicrobial agents has been extensively explored [14, 15, 16, 17], particularly in the fight against MRSA [18]. Pyrazole derivatives may possess modes of action distinct from those of *β*‐lactams and glycopeptide antibiotics. The abundance of modifiable sites on the pyrazole scaffold offers a valuable opportunity for discovering novel antibacterial lead compounds with new targets and mechanisms of action.

Pyrimidine nucleotides are core components for the synthesis of DNA, RNA, and other functional proteins, playing a crucial role in all organisms [19]. There are two pyrimidine synthesis pathways: the salvage pathway and the *de novo* pathway. Generally, slowly dividing or differentiating cells preferentially use the salvage pathway to uptake nucleosides from the external environment or produced by nucleic acid hydrolysis; however, this cannot meet the continuous demand for nucleic acids in proliferating cells, and some pathogens even lack functional salvage pathways [20, 21, 22]. For bacteria, due to the extremely low concentration of pyrimidines in the blood that is insufficient to sustain bacterial growth, pathogens must rely on their own pyrimidine synthesis pathways. Previous studies have demonstrated that defects in several key enzymes in the pyrimidine synthesis pathway can reduce bacterial titers in the blood by a thousandfold [23]. Currently, crizotinib has been confirmed to reduce ATP production by targeting the CTP (cytidine triphosphate) synthase *pyrG*, exhibiting broad‐spectrum antibacterial activity against Gram‐positive bacteria [24]. In addition, the pyrimidine biosynthesis pathway regulated by the *pyr* operon has also been proven to be associated with the colonization of *Staphylococcus aureus* [25]. This indicates that the nucleotide biosynthesis pathway not only plays a central role in bacterial survival and proliferation but also has a close connection with virulence factors that underlie bacterial pathogenicity [26]. More importantly, although the *de novo* pyrimidine synthesis pathway is a highly conserved pathway essential for cell proliferation in all living organisms, the characteristic of covalently linking the first several active enzymes into a CAD multi‐enzyme complex is unique to humans and animals. In contrast, in other organisms, these enzymes exist as active monofunctional proteins [20]. This implies that inhibiting the *de novo* pyrimidine synthesis pathway is expected to become a new direction for the development of novel anti‐MRSA drugs.

Aspartate transcarbamoylase (ATCase), encoded by the *pyrB* gene, is a key enzyme that catalyzes the second step of the *de novo* pyrimidine synthesis pathway. This reaction involves the condensation of L‐aspartate (LASP) and carbamoyl phosphate (CP) to form carbamoyl aspartate (CP‐ASP) and inorganic phosphate. Crucially, this step is the first committed and irreversible reaction in the pyrimidine nucleotide production pathway [27]. ATCase has already been validated as a promising target for the development of antimalarial [21, 28, 29, 30], anti‐mycobacterium tuberculosis [31], and anticancer drugs [32, 33]. Compared to other enzymes in the *de novo* pyrimidine synthesis pathway, ATCase, as the rate‐limiting step enzyme, represents an even more ideal drug target.

To identify novel antibacterial lead compounds with new targets and antimicrobial mechanisms, this study performed structural optimization using pyrazole as the core scaffold, identified Py‐27 as a potent candidate, and applied a chemoproteomic approach to reveal aspartate transcarbamoylase (ATCase), a key enzyme in *de novo* pyrimidine biosynthesis, as the primary antibacterial target of Py‐27. Functional and mechanistic studies demonstrated that Py‐27 inhibits the enzymatic activity of ATCase, thereby disrupting the *de novo* pyrimidine synthesis pathway, inhibiting DNA synthesis, perturbing metabolic homeostasis, and altering bacterial redox balance, ultimately exerting its antibacterial effects. Py‐27 also exhibits a low propensity for resistance development, retains activity against clinically resistant MRSA strains, and can effectively disrupt biofilms. In animal infection models, Py‐27 reduces bacterial burden and contributes to infection resolution and tissue repair. Overall, this study is the first to discover a novel anti‐MRSA lead compound targeting ATCase, reaffirms the potential of chemical synthesis to expand the antibacterial drug library, and provides a promising clue for addressing the global threat of MRSA.

## 2 Results

### 2.1 Structure‐Activity Relationship of Pyrazole‐Amide Hybrids With Antibacterial Activity

**FIGURE 1:**
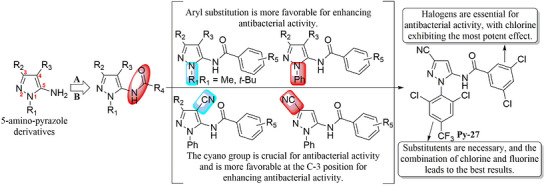
Synthesis of pyrazole‐amide derivatives and their antibacterial structure‐activity relationship.

Pyrazole compounds have demonstrated significant potential in treating MRSA infections due to their unique structural features [18]. However, systematic studies linking phenotypic confirmation to mechanistic discovery, particularly the identification and validation of antibacterial targets, remain scarce. The aryl amide pharmacophore plays a critical role in the structure of many bioactive molecules owing to the distinctive physicochemical properties of aryl substituents and their advantages in drug‐target interactions [34]. Compared with alkyl substituents, aromatic rings contain a delocalized *π*‐electron system, which can enhance the stability of the drug‐target complex. Different from the flexible structure of alkyl substituents, the rigidity of aryl rings helps lock the drug molecule in a specific three‐dimensional conformation; in particular, this structural rigidity is also reflected in the geometric configuration of the amide bond itself, thereby affecting the overall conformation of the molecule [35]. Moreover, the flat and rigid structure of aryl groups may enable them to more easily fill the hydrophobic or aromatic regions in the target binding pocket, forming a tighter and more stable binding [36]. Therefore, to discover more potent pyrazole derivative antibacterial agents, we used 5‐amino‐pyrazole derivatives as the starting material and reacted it with substituted benzoyl chlorides (General Procedure A, See Text S1 for detail) or substituted benzoic acids (General Procedure B, See Text S1 for detail) to construct the aryl amide bond (Figure 1), synthesizing a series of pyrazole‐amide hybrid derivatives (Py‐1 to Py‐45, Table 1, See the Text S1 for details). The antibacterial activities of these compounds against MRSA (ATCC 43300) were evaluated, as shown in Table S2. Among all tested compounds, the pyrazole‐amide hybrid Py‐27 exhibited the most potent activity.

**TABLE 1:** Structure, yield, MIC, and structure–Activity relationship analysis of the pyrazole–amide derivatives.

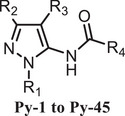							
Entry	R_1_	R_2_	R_3_	R_4_	Yield	MRSA µg/mL	Structure‐activity relationship
Py‐1	Ph	H	H	Ph	78%	>64	Cyano substituents at the C‐3 or C‐4 position have a crucial influence on the antibacterial activity of pyrazole derivatives.
Py‐2	Ph	H	H	4‐CF_3_‐Ph	67%	>64	
Py‐3	Ph	H	H	3,5‐diCl‐Ph	61%	>64	
Py‐4	Ph	H	H	2,4‐diCl‐Ph	61%	>64	
Py‐5	Ph	H	H	4‐CH_3_‐Ph	71%	>64	
Py‐6	Ph	H	H	3‐Br‐Ph	79%	>64	
Py‐7	Ph	H	H	4‐I‐Ph	68%	>64	
Py‐8	Ph	H	H	2‐CF_3_‐Ph	65%	>64	
Py‐9	Ph	H	H	3‐CF_3_‐Ph	71%	>64	
Py‐10	Ph	*t*‐bu	H	3,5‐diCl‐Ph	61%	>64	
Py‐11	Ph	Me	Br	3,5‐diCl‐Ph	56%	>64	
Py‐12	Me	H	H	3,5‐diCl‐Ph	91%	>64	
Py‐13	Me	H	H	4‐CF_3_‐Ph	74%	>64	
Py‐14	Me	Ph	H	3,5‐diCl‐Ph	44%	>64	
Py‐15	Me	*t*‐bu	H	3,5‐diCl‐Ph	84%	>64	
Py‐16	*t*‐bu	H	CN	4‐CF_3_‐Ph	30%	64	(a) The cyano group is crucial for antibacterial activityand is more favorable at the C‐3 position forenhancing antibacterial activity. (b) A substituted aryl group at the *N*‐1 position, with halogen substituents (F, Cl) on the aryl ring, is more favorable for enhancing the antibacterial activity of pyrazole derivatives. (c) When R_4_ is a halogen group, it is more favorable for improving the antibacterial activity of pyrazole derivatives.
Py‐17	Ph	H	CN	4‐CF_3_‐Ph	64%	>64	
Py‐18	Ph	H	CN	3,5‐diCl‐Ph	77%	32	
Py‐19	4‐Me‐Ph	H	CN	4‐CF_3_‐Ph	65%	>64	
Py‐20	4‐Me‐Ph	H	CN	3,5‐diCl‐Ph	59%	32	
Py‐21	4‐Cl‐Ph	H	CN	4‐CF_3_‐Ph	58%	32	
Py‐22	4‐Cl‐Ph	H	CN	3,5‐diCl‐Ph	61%	8	
Py‐23	4‐F‐Ph	CO_2_Et	CN	3,5‐diCl‐Ph	38%	32	
Py‐24	4‐NO_2_‐Ph	H	CN	3,5‐diCl‐Ph	31%	>64	
Py‐25	4‐Cl‐Ph	CN	H	3,5‐diCl‐Ph	63%	0.5	
Py‐26	2,4,6‐triCl‐Ph	CN	H	3,5‐diCl‐Ph	53%	0.5	
Py‐27	2,6‐diCl‐4‐CF_3_‐Ph	CN	H	3,5‐diCl‐Ph	95%	0.125	
Py‐28	2‐F‐4‐Cl‐Ph	CN	H	3,5‐diCl‐Ph	58%	0.25	
Py‐29	2‐Cl‐5‐Me‐Ph	CN	H	3,5‐diCl‐Ph	43%	2	
Py‐30	4‐Me‐Ph	CN	H	3,5‐diCl‐Ph	76%	0.5	
Py‐31	4‐OMe‐Ph	CN	H	3,5‐diCl‐Ph	72%	8	
Py‐32	2,4‐diMe‐Ph	CN	H	3,5‐diCl‐Ph	60%	2	
Py‐33	2,6‐diCl‐4‐CF_3_‐Ph	CN	H	4‐I‐Ph	57%	1	
Py‐34	2,6‐diCl‐4‐CF_3_‐Ph	CN	H	3,5‐diF‐Ph	84%	1	
Py‐35	2,6‐diCl‐4‐CF_3_‐Ph	CN	H	2,4‐diCl‐Ph	69%	2	
Py‐36	2,6‐diCl‐4‐CF_3_‐Ph	CN	H	4‐OCF_3_‐Ph	41%	0.5	
Py‐37	2,6‐diCl‐4‐CF_3_‐Ph	CN	H	2‐Cl‐4‐F‐Ph	63%	8
Py‐38	2,6‐diCl‐4‐CF_3_‐Ph	CN	H	4‐CF_3_‐Ph	75%	0.25
Py‐39	2,6‐diCl‐4‐CF_3_‐Ph	CN	H	4‐Me‐Ph	56%	>64
Py‐40	2,6‐diCl‐4‐CF_3_‐Ph	CN	H	3‐CF_3_‐Ph	62%	0.5
Py‐41	2,6‐diCl‐4‐CF_3_‐Ph	CN	H	3‐Br‐Ph	78%	1
Py‐42	2,6‐diCl‐4‐CF_3_‐Ph	CN	H	3,5‐diNO_2_‐Ph	47%	8
Py‐43	2,6‐diCl‐4‐CF_3_‐Ph	CN	H	3,5‐diCF_3_‐Ph	69%	0.5
Py‐44	2,6‐diCl‐4‐CF_3_‐Ph	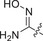	H	3,5‐diCF_3_‐Ph	45%	16	The cyano group is crucial for the antibacterial activity of the compound.
Py‐45	2,6‐diCl‐4‐CF_3_‐Ph	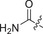	H	3,5‐diCF_3_‐Ph	41%	>64

The results of preliminary structure‐activity relationship (SAR) studies indicated that the introduction of an aryl group at the N‐1 position was more favorable for enhancing antibacterial activity. The antibacterial activity results of different substituents at the C‐3 and C‐4 positions revealed the importance of the cyano group for antibacterial activity, with the cyano group at the C‐3 position being more conducive to improving the antibacterial activity of the compound. Furthermore, halogen substituents on the aryl ring, particularly chlorine and fluorine, were more favorable for enhancing the activity of the compound (Figure 1, Table 1).

**FIGURE 2:**
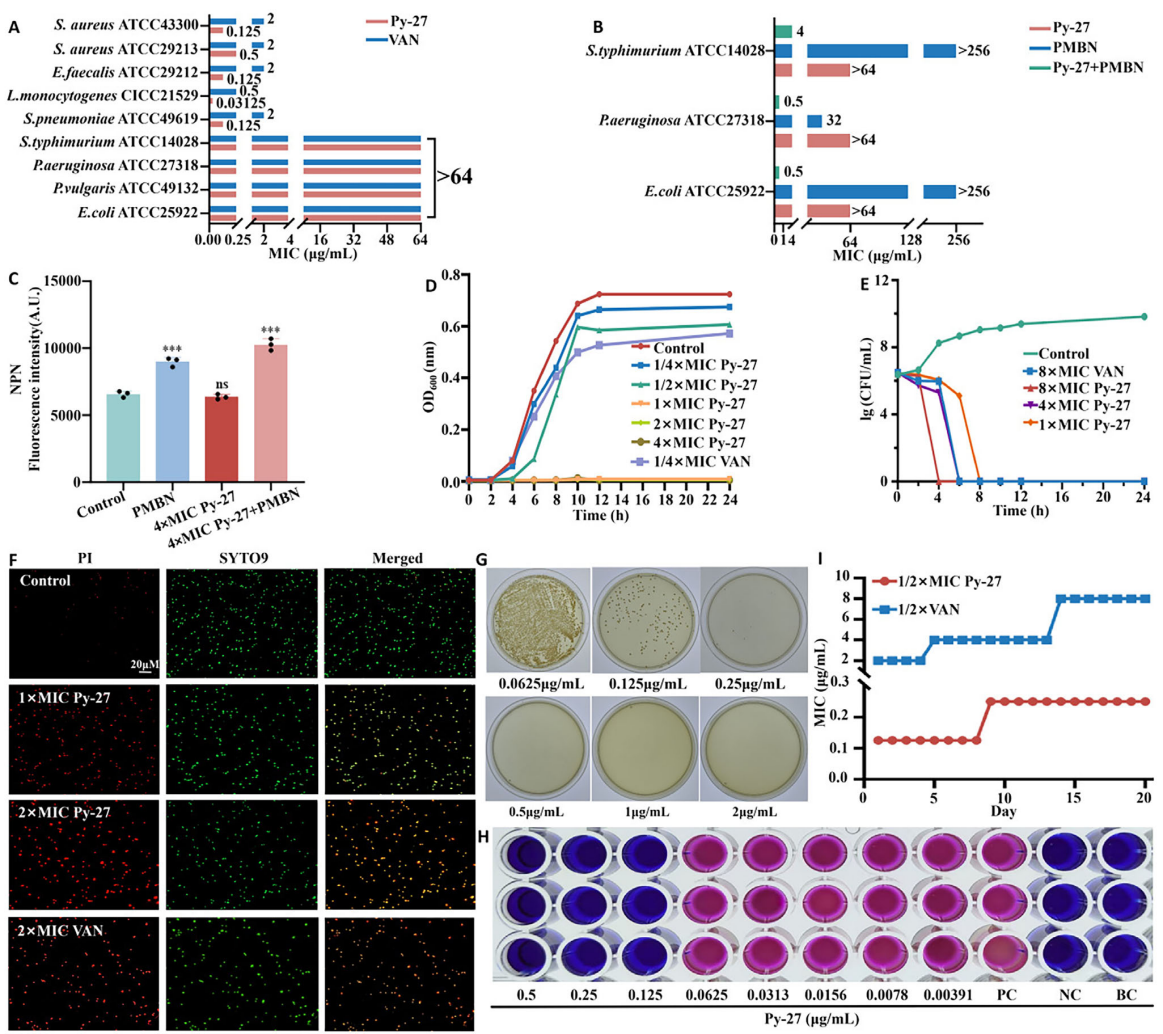
The antibacterial properties of Py‐27 in vitro. (A) The activity of Py‐27 against pathogenic bacteria (*n* = 3). (B) Effect of PMBN on the MIC of Py‐27 against Gram‐negative bacteria (*n* = 3). (C) The result of outer membrane permeability of *E. coli* (*n* = 3). (D) Growth curve of Py‐27 against MRSA (*n* = 3). (E) Time‐kill curve of Py‐27 against MRSA (*n* = 3). (F) Fluorescence photos of MRSA stained with SYTO 9 (green, live bacteria) and PI (red, dead bacteria) after corresponding treatments. (G) MBC of Py‐27 against MRSA. (H) MIC of Py‐27 against MRSA. PC: positive control (MH containing MRSA without Py‐27); NC: negative control (MH containing Py‐27 without MRSA); BC: blank control (MH only) (*n* = 3). (I) The development of drug resistance to Py‐27 over 20 days of consecutive subculturing (*n* = 3). Data were presented as mean ± SD. Statistical significance was analyzed by ordinary one‐way ANOVA (^***^p < 0.001; and ns, not significant, respectively).

In addition, the antibacterial activity of Py‐27 against other bacterial strains, including *Escherichia coli* (ATCC 25922), *Pseudomonas aeruginosa* (ATCC 27318), *Proteus vulgaris* (ATCC 49132), *Salmonella* typhimurium (ATCC 14028), *Enterococcus faecalis* (ATCC 29212), *Streptococcus pneumoniae* (ATCC 49619), and *Listeria monocytogenes* (CICC 21529), was tested. The results showed that Py‐27 was ineffective against the tested Gram‐negative bacteria, indicating selective activity against Gram‐positive bacteria (MIC: 0.03125 to 0.125 µg/mL, Figure 2A).

However, Py‐27 showed no significant activity against Gram‐negative bacteria, which may be related to the structural differences of bacteria. Gram‐negative bacteria possess an outer membrane that can block the entry of large molecules. Py‐27 might be prevented from entering the cell by the outer membrane of Gram‐negative bacteria. To verify this hypothesis, the outer membrane permeability of Gram‐negative bacteria was artificially modulated. The minimum inhibitory concentrations (MICs) of Py‐27 against *E. coli*, *P. aeruginosa*in and *S. typhimurium* were determined in the presence of polymyxin B nonapeptide (PMBN), a well‐established outer membrane permeabilizer. Following PMBN treatment, the MIC values of Py‐27 against *E. coli*, *P. aeruginosa*in and *S. typhimurium* dropped sharply from over 64 to 0.5, 0.5, and 4 µg/mL, respectively (Figure 2B). These results demonstrated that limited outer membrane penetration is the key factor restricting the anti‐Gram‐negative activity of Py‐27. To further examine the role of the permeability barrier, an NPN (*N*‐Phenyl‐1‐naphthylamine) uptake assay was performed on *E. coli* (Figure 2C). When used alone, Py‐27 yielded a fluorescence intensity comparable to the untreated control group, suggesting that it does not significantly disrupt outer membrane permeability by itself. In contrast, PMBN significantly increased NPN fluorescence, and the combination of Py‐27 and PMBN produced the highest fluorescence signal among all groups (*p* < 0.001). These results further demonstrate that the outer membrane barrier of Gram‐negative bacteria is the primary determinant limiting the antibacterial activity of Py‐27.

### 2.2 In Vitro Antibacterial Activity and Bactericidal Assessment of Py‐27

Determining whether a compound is bactericidal or bacteriostatic is of paramount importance for clinical application [37]. Therefore, after confirming the potent anti‐MRSA activity of Py‐27, we further evaluated its bactericidal assessment. The growth curve showed that Py‐27 completely inhibited bacterial growth at concentrations above the minimum inhibitory concentration (MIC). At 1/2 × MIC, the growth rate of the strain was slowed, and the bacterial count was significantly reduced. In contrast, at 1/4 × MIC, Py‐27 had almost no significant effect on bacterial growth (Figure 2D). Furthermore, we monitored the viability of MRSA exposed to different concentrations of Py‐27 at various time points (Figure 2E). Py‐27 at concentrations of 8 × MIC, 4 × MIC, and MIC killed the bacteria within 8 h and eliminated most bacteria over a longer period. Notably, at 8 × MIC, Py‐27 completely eradicated the bacteria within 4 h, a bactericidal rate significantly faster than that of the control drug vancomycin at 8 × MIC. Under fluorescence microscopy, SYTO 9 stained all bacteria green, while propidium iodide (PI) stained only dead bacteria red. The control group showed only minimal red fluorescence. However, as the concentration of Py‐27 increased, the intensity of red fluorescence significantly increased, indicating that Py‐27 likely causes bacterial death by disrupting the cell membrane (Figure 2F). Based on the ratio of the minimum bactericidal concentration (MBC, 0.25 µg/mL, Figure 2G) to the minimum inhibitory concentration (MIC, 0.125 µg/mL, Figure 2H), and the results of the live/dead fluorescence staining, we confirmed that Py‐27 is a potent bactericidal candidate drug.

The efficacy of an antibacterial compound against clinically resistant bacteria and its impact on the development of bacterial resistance are critical indicators for evaluating its clinical potential [38]. Therefore, we first assessed the antibacterial activity of Py‐27 against clinically isolated MRSA strains. The results showed that Py‐27 exhibited superior activity compared to vancomycin against 45 clinical MRSA isolates (Table S3, Isolated from dairy farms in four Chinese provinces, all were resistant to ampicillin). Continuous exposure of MRSA to 1/2 × MIC of Py‐27 for 20 days showed a markedly lower tendency for resistance development relative to vancomycin (Figure 2I) and ciprofloxacin (Figure S12), highlighting its potential for clinical application.

### 2.3 Activity of Py‐27 Against MRSA Biofilms

**FIGURE 3:**
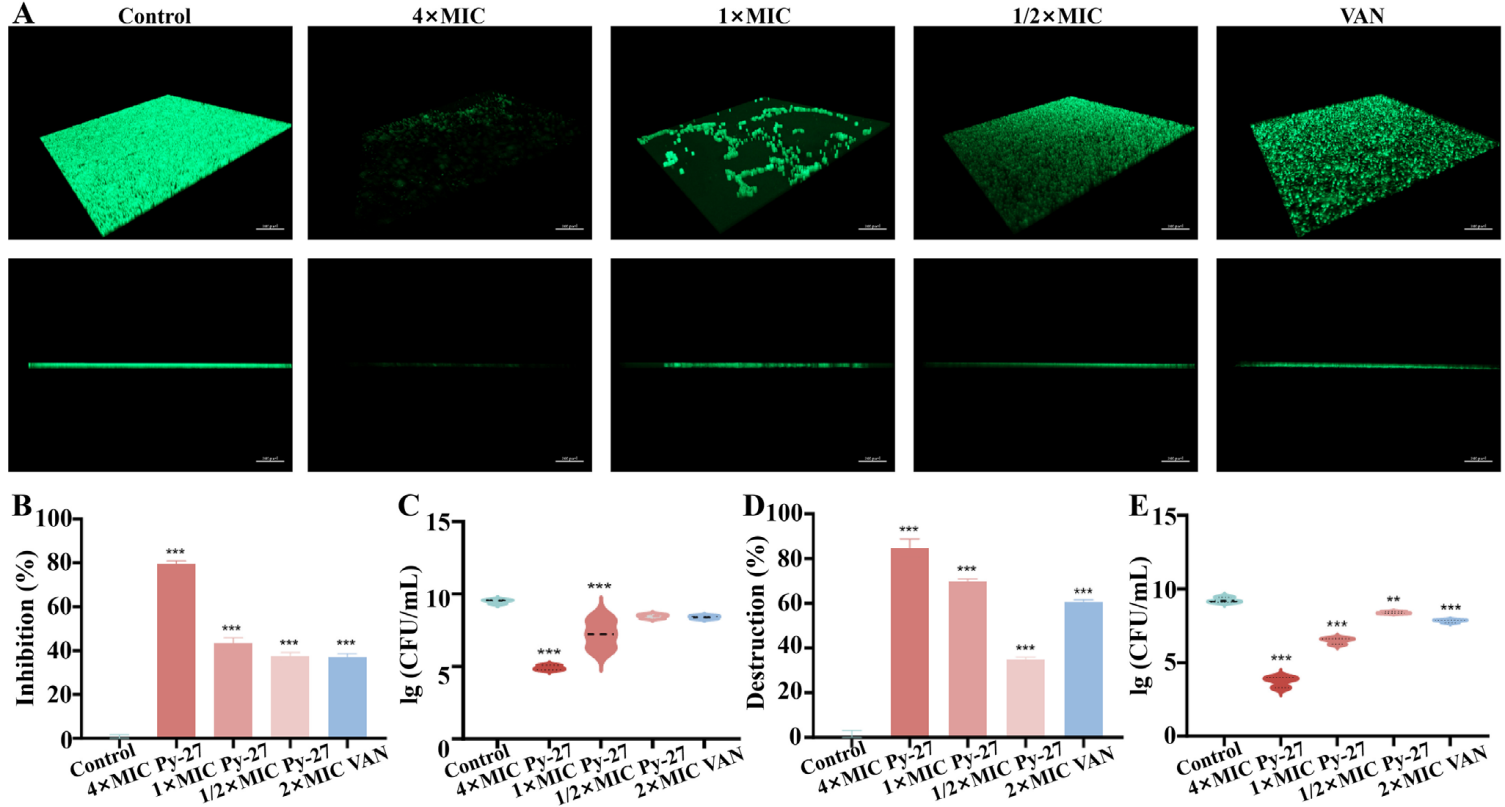
The antibiotic biofilm activity of Py‐27. (A) 3D structure of MRSA mature biofilms (stained with SYTO 9) after different treatments at 24 h by CLSM (right, side view; left, top view). (B) Inhibition rate of immature biofilm after treatment of Py‐27. (C) Count of viable bacteria of MRSA immature biofilms in different groups. (D) Destruction rate of mature biofilm after corresponding treatments. (E) Count of viable bacteria of MRSA mature biofilms in different groups. All experiments were conducted with three biological replicates and data were presented as mean ± SD. Statistical significance was analyzed by ordinary one‐way ANOVA (^**^p < 0.01, ^***^p < 0.001).

Biofilms are structured communities of bacteria attached to biotic or abiotic surfaces and encased in a self‐produced extracellular polymeric matrix. This structure significantly enhances bacterial tolerance to various antimicrobial agents posing a major challenge to the treatment of bacterial infections and contributing to the chronicity and severity of many clinical diseases [39]. Therefore, we further evaluated the activity of Py‐27 in inhibiting MRSA biofilm formation and eradicating mature biofilms. Crystal violet staining results showed that Py‐27 at a sub‐inhibitory concentration (1/2 × MIC) significantly inhibited MRSA biofilm formation (Figure 3B). Quantification of viable bacteria in the residual biofilm revealed that while 1/2 × MIC Py‐27 inhibited biofilm formation, it did not significantly reduce the viable bacterial load (Figure 3C). 3D structural imaging and crystal violet staining results showed that Py‐27 at concentrations of 1 × MIC and 4 × MIC, Py‐27 effectively eradicated over 65% of mature MRSA biofilms (Figure 3A, D). Confocal laser scanning microscopy (CLSM) imaging of the 3D biofilm structure clearly showed that the 4 × MIC Py‐27 group exhibited the weakest green fluorescence signal and the thinnest biofilm thickness, indicating a potent biofilm eradication capability (Figure 3A, relevant quantitative bar charts are provided in Figure S13A). Additionally, quantitative analysis of viable bacteria within the biofilm (Figure 3E) confirmed that Py‐27 not only efficiently cleared mature MRSA biofilms, but also effectively killed the bacteria embedded within the mature biofilm, demonstrating significant anti‐biofilm activity.

### 2.4 Safety Evaluation of Py‐27

**FIGURE 4:**
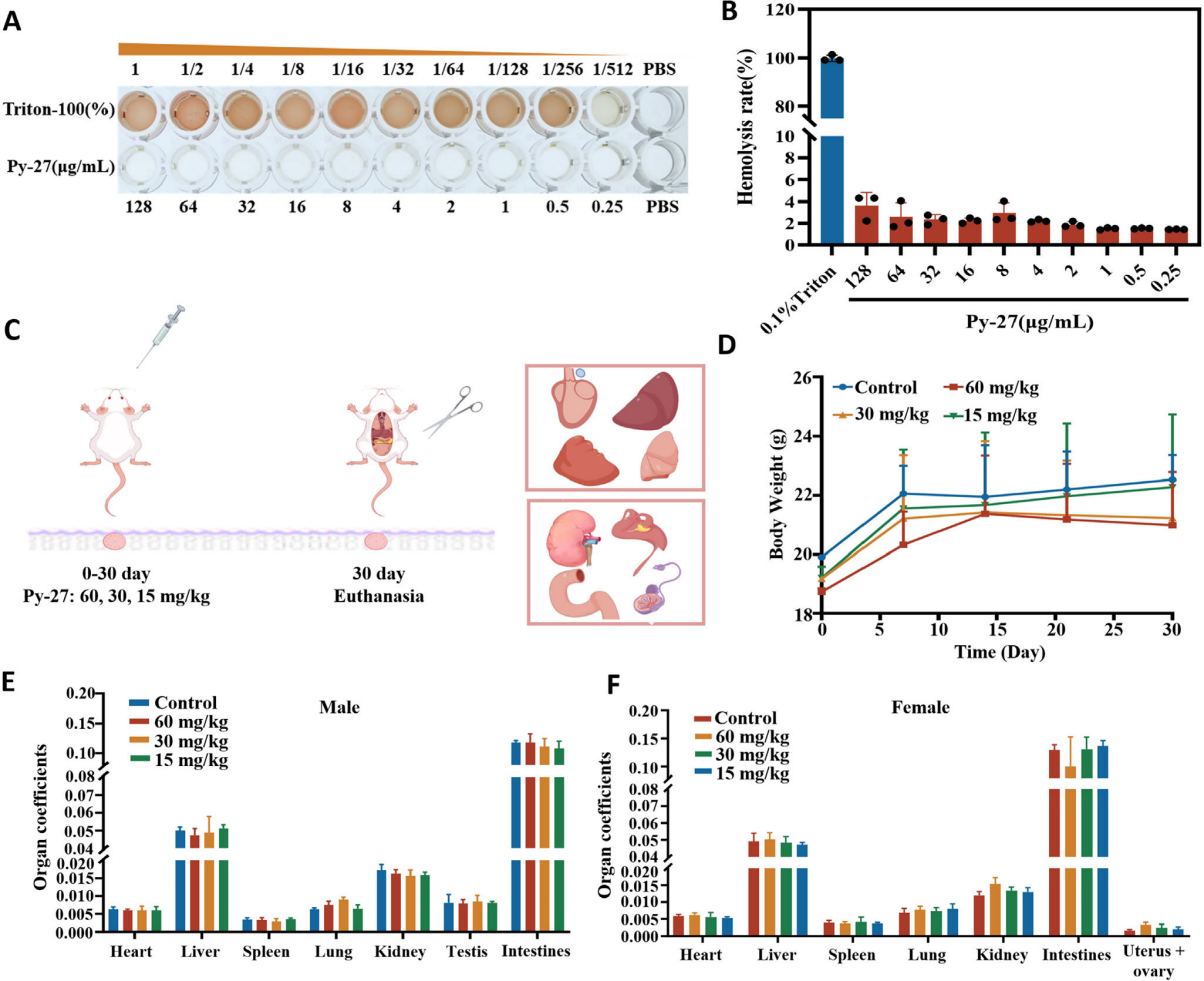
Safety evaluation of Py‐27. (A) Representative images for the Py‐27 and Triton X‐100 hemolysis rates. (B) Quantitative statistical data for the Py‐27 and Triton X‐100 hemolysis rates. (C) The protocol of 30‐day feeding test (*n* = 3). (D) Change of body weight in each group (*n* = 10). (E) Organ coefficients in each group of male mice on day 30 (*n* = 10). (F) Organ coefficients in each group of female mice on day 30 (*n* = 3). Data were presented as mean ± SD. Statistical significance was analyzed by ordinary two‐way ANOVA.

The safety profile of an active compound is a fundamental prerequisite and bottom line for drug development and clinical application. This study conducted a preclinical safety evaluation of Py‐27. First, we assessed the hemolytic activity of Py‐27 to determine its potential to damage red blood cells. In vitro hemolysis assay results showed no visible hemolysis after co‐incubation of compound Py‐27 with sheep red blood cell suspension. Even at a concentration of 128 µg/mL (1024 times the MIC), Py‐27 did not exhibit any hemolytic reaction, indicating no potential risk of inducing hemolysis (Figure 4A, B).

In the acute toxicity study, Py‐27 was orally administered to BALB/c mice, yielding an LD_50_ of 0.852 g/kg (low toxicity) with no observed treatment‐related pathological lesions. Based on the acute toxicity results, a subchronic toxicity study was conducted by administering Py‐27 orally once daily at doses of 15, 30, and 60 mg/kg/day for 30 consecutive days (Figure 4C). Throughout the study period, no Py‐27‐related deaths, clinical symptoms, general appearance abnormalities, or behavioral changes were observed. All animals exhibited normal weight gain, independent of the administered substance and dose (Figure 4D). There were no significant differences in organ coefficients among the groups (Figure 4E, F). Furthermore, blood routine (Figure S1A–H) and blood biochemical (Figure S1I–P) remained within the normal range, and no inflammatory infiltration was observed in major organs, confirming its in vivo biosafety (Figure S1Q). Based on the comprehensive analysis of acute and subchronic toxicity results, Py‐27 was well‐tolerated in mice at a daily oral dose of up to 60 mg/kg.

Subsequently, we systematically evaluated the genotoxicity of Py‐27 through the Ames test, micronucleus test, and chromosome aberration test. In all tested strains, the number of colonies in the Py‐27 treatment group was comparable to that of the control group, while the positive control group showed a significant increase in colony count (Table S4). No significant increase in colony count was observed after metabolic activation of Py‐27 using the S9 mixture. Py‐27 does not induce mutations in the tested strains, confirming that it lacks intrinsic mutagenicity. The sperm abnormality rate in mice treated with Py‐27 showed no significant difference from the negative control group, whereas the cyclophosphamide‐treated group exhibited a significant increase (Table S5), indicating that Py‐27 is not teratogenic. In addition, the number of micronuclei in polychromatic erythrocytes in the bone marrow of BALB/c mice administered orally by Py‐27 was detected. A micronucleus incidence of 3–5‰ in the 0.43 g/kg dose group was found, compared to 3–6‰ in the negative control group and 27–32‰ in the positive control group. There was no statistically significant difference in micronucleus incidence between the Py‐27 treatment group and the negative control group (*p* > 0.05), while the positive control group showed a significantly higher micronucleus rate than the negative control group (*p* < 0.05), indicating that Py‐27 is not carcinogenic (Table S6). The above evaluation results indicate that Py‐27, as an anti‐MRSA lead compound, possesses a favorable safety profile.

### 2.5 Py‐27 Showed Potential Therapeutic Capabilities in Different Infection Models

Given the excellent anti‐MRSA activity, low propensity for resistance development, and favorable safety profile of Py‐27, we proceeded to evaluate its in vivo efficacy prior to further investigation of its antibacterial mechanism.

**FIGURE 5:**
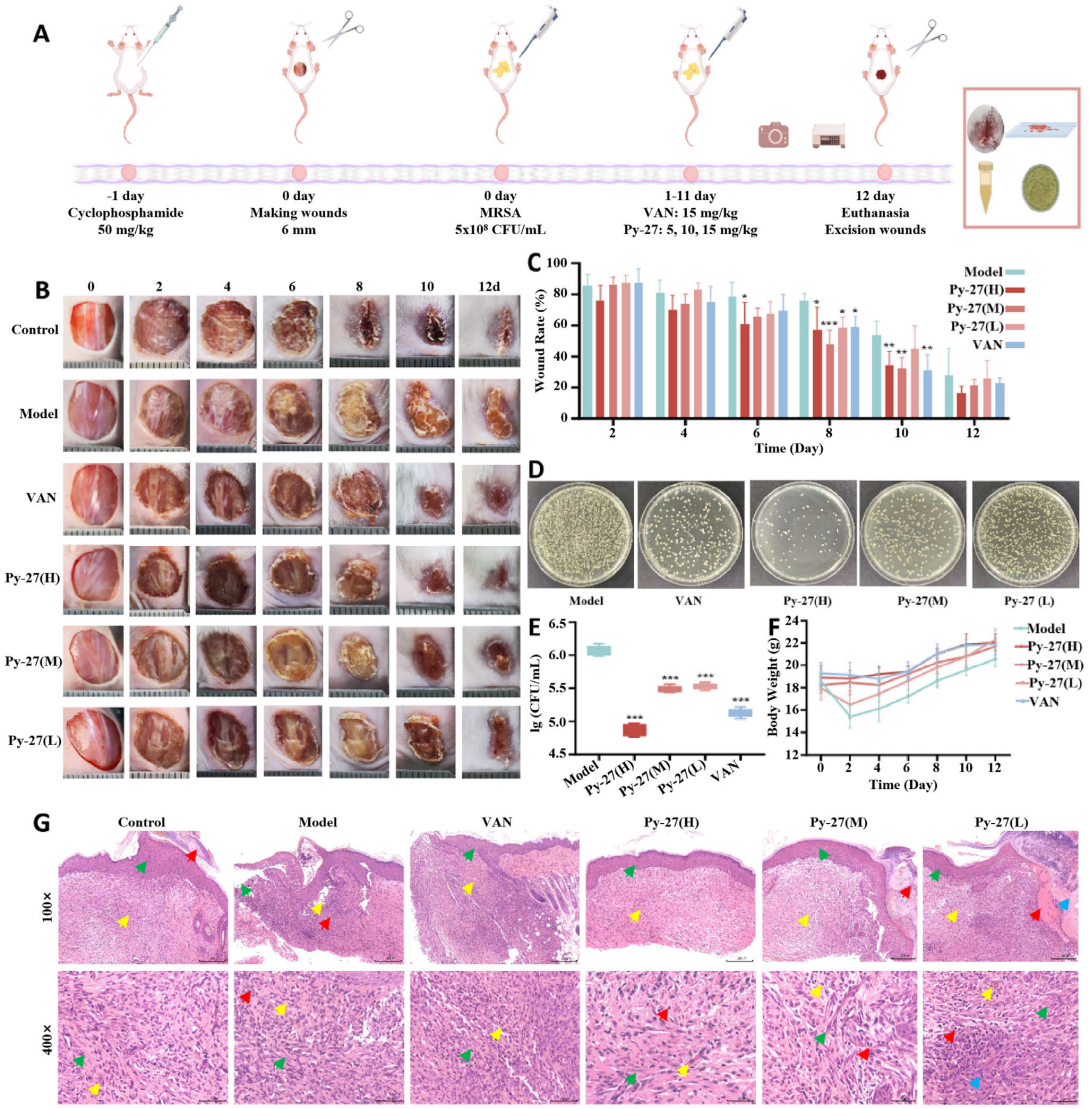
The efficacy of Py‐27 in mice skin wound infection model. (A) The protocol was established for the mice skin wound infection. (B) Representative photos of the wounds were recorded. (C) The trend of wound area in mice (*n* = 6). (D) Photos of wound secretion coating on the seventh day. (E) Bacterial count from the wound tissues (*n* = 3). (F) Change of body weight in each group (*n* = 6). (G) Pathological assay of infected skin that received different treatments based on H&E staining. Data were presented as mean ± SD. Statistical significance was analyzed by ordinary one‐way or two‐way ANOVA (^*^p < 0.05, ^**^p < 0.01, ^***^p < 0.001).

Skin infection is a common disease caused by MRSA, occurring both in community and hospital settings [40]. To evaluate the therapeutic potential of Py‐27, a skin infection model was first used for assessment (Figure 5A). MRSA infection led to delayed wound healing and exacerbated the infection severity, while bacterial infection wounds in mice treated with Py‐27 exhibited accelerated healing progress (Figure 5B, C). The Py‐27(H) (15 mg/kg) treatment group significantly reduced the skin bacterial load, and its efficacy was superior to that of vancomycin at the same dose (Figure 5D, E). All animals showed normal weight gain, which was independent of the administered substance and dose (Figure 5F). Further histological examination revealed that animals in the control group exhibited effective skin tissue repair, characterized by a continuous epidermal layer, well‐developed granulation tissue in the dermis, and minimal inflammatory cell infiltration. In contrast, mice in the model group showed impaired wound healing after skin trauma. Notably, treatment with Py‐27(H) also exhibited significantly enhanced tissue regeneration ability, and its skin repair effect was superior to that of the blank control group (Figure 5G).

**FIGURE 6:**
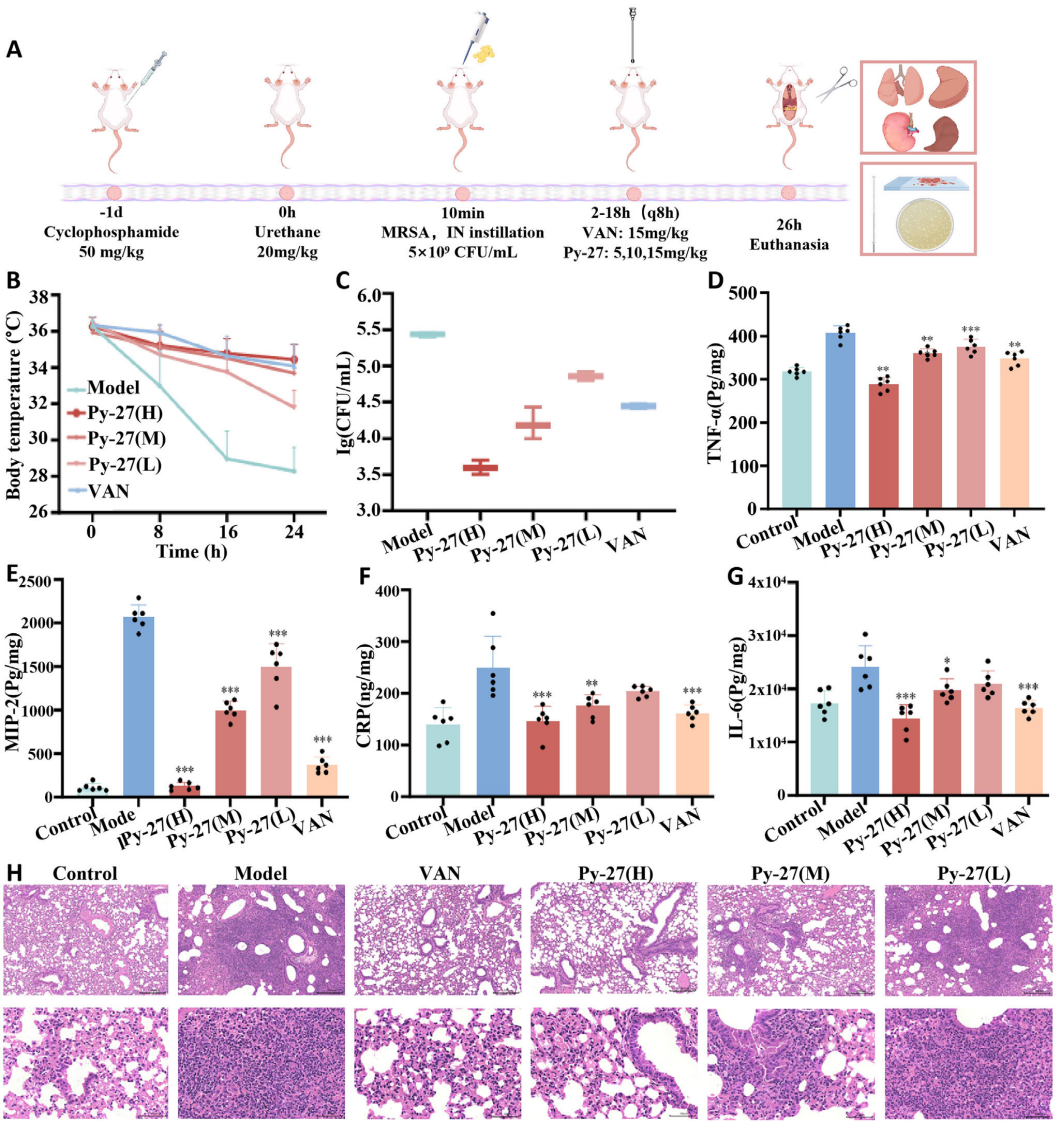
Therapeutic effect Py‐27 in acute pneumonia infection. (A) Experimental protocol for acute pneumonia infection. (B) Core body temperature of MRSA pneumonia mice in corresponding groups (*n* = 6). (C) Quantitative analysis of MRSA after different treatments at 24 h (*n* = 6). (D–G) Quantitative analysis of the levels of TNF‐α, MIP‐2, CRP and IL‐6 in lung homogenates of different treatments after infection (*n* = 6). (H) H&E staining of Lung. Data were presented as mean ± SD. Statistical significance was analyzed by ordinary one‐way ANOVA (^*^p < 0.05, ^**^p < 0.01, ^***^p < 0.001).

MRSA infections in the hospital environment are not limited to the skin; it is also a major pathogen of hospital‐acquired pneumonia (including ventilator‐associated pneumonia) and can cause community‐acquired pneumonia [41]. Therefore, we applied Py‐27 to a model of acute pneumonia induced by MRSA to evaluate its therapeutic potential (Figure 6A). The Py‐27 group demonstrated the best ability to maintain core body temperature, while the model group's temperature dropped to 28°C 12 h post‐infection (Figure 6B). Assessment of the bacterial load in the lungs revealed that the Py‐27(H) (15 mg/kg) treatment group had the lowest colony‐forming units (CFU), indicating the best in vivo antibacterial activity (Figure 6C). We subsequently evaluated the effect of Py‐27 to further assess its efficacy in treating acute MRSA pneumonia. Moreover, the Py‐27(H) group had the lowest production of inflammatory factors, indicating the mildest inflammatory response and a therapeutic effect superior to that of vancomycin (Figure 6D–G). These results were further confirmed by H&E staining. Histological analysis showed varying degrees of structural loss in the lung tissue of all groups, with interstitial vessels exhibiting edema, hemorrhage, and necrosis, and inflammatory cells infiltrating the alveolar spaces (Figure 6H). In the Py‐27(H) treatment group, the alveolar structure remained clearly discernible, with only partial alveolar wall thickening due to edema and focal infiltration of inflammatory cells in the alveolar spaces. The degree of lung damage was significantly reduced compared to the other groups, and no significant pathological changes were observed in the kidneys, liver, or spleen (Figure S2).

Overall, Py‐27 exhibited superior therapeutic efficacy to vancomycin in both MRSA‐induced skin wound infection models and acute pneumonia models. These findings support that Py‐27 can serve as an effective therapeutic approach for MRSA‐induced skin and lung infections in mouse models from both macroscopic and microscopic perspectives, which also highlighting its potential for further investigation into its bactericidal mechanisms.

### 2.6 Discovery of Anti‐MRSA Target From Chemoproteomics Analysis

The pyrazole‐amide derivative Py‐27 has demonstrated superior anti‐MRSA activity compared to vancomycin in both in vitro and in vivo evaluations, along with a favorable safety profile. Based on these results, we planned to identify the biological target of Py‐27 using chemoproteomics. We designed and synthesized a molecular probe derived from compound Py‐27 (Text S2). All synthesized compounds were characterized and confirmed by nuclear magnetic resonance (NMR) spectroscopy and high‐resolution mass spectrometry (HRMS). A qualified molecular probe should exhibit biological activity comparable to that of the parent compound to ensure it binds to the same target. In vitro antibacterial activity evaluation showed that the probe molecule Py‐27‐P and the parent compound Py‐27 had minimum inhibitory concentrations (MICs) of 0.5 and 0.125 µg/mL against MRSA, respectively. This met the requirements for a probe molecule, allowing its application in chemoproteomics to search for the biological binding targets of Py‐27.

**FIGURE 7:**
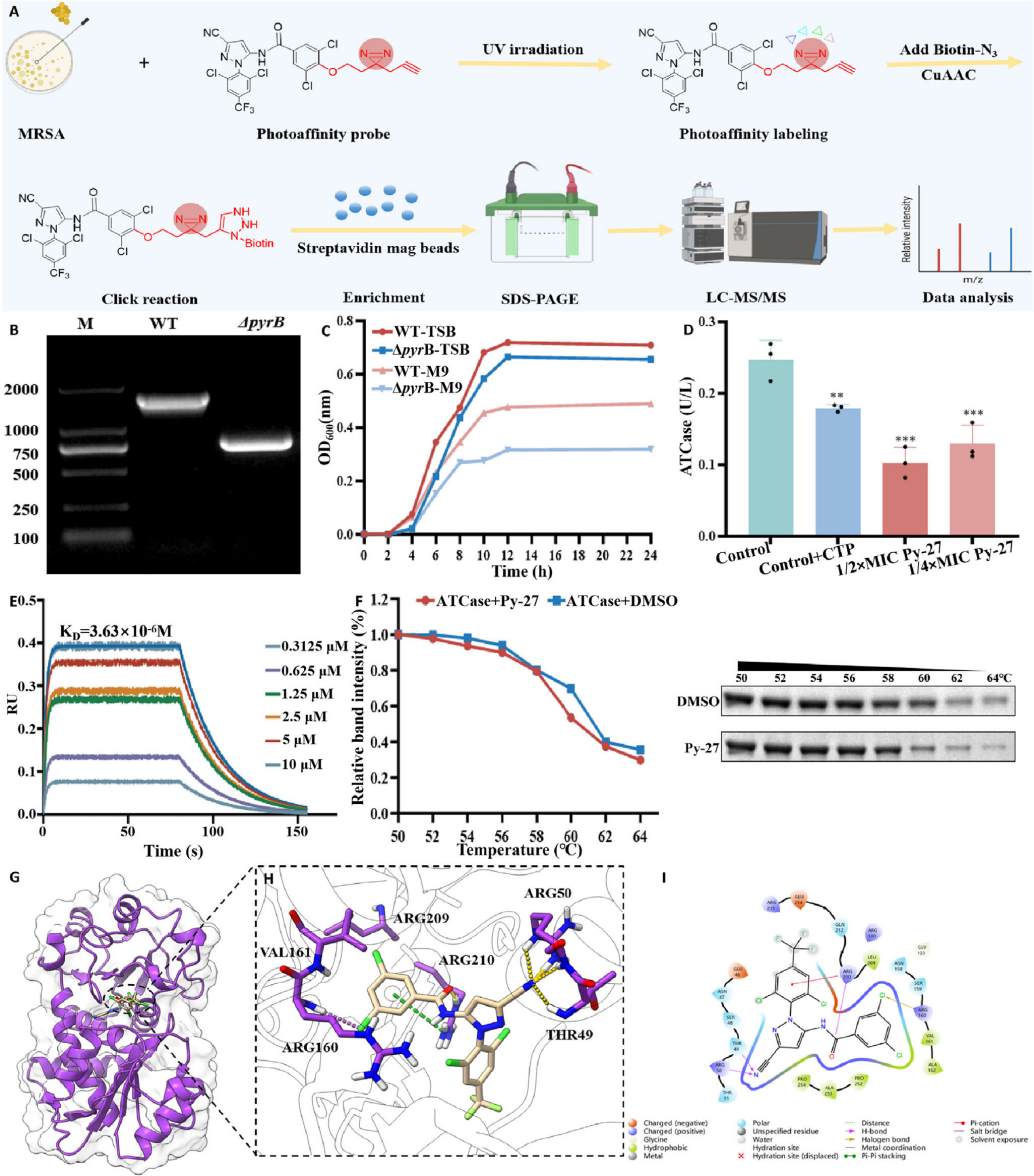
ATCase Binding by Py‐27. (A) Comprehensive workflow for ABPP‐based profiling of potential Py‐27 targets. (B) PCR identification of Δ*pyrB*. M: DL2000 DNA marker. (C) Growth curve of WT and Δ*pyrB* with TSB or M9 (*n* = 3). (D) The activity of aspartate transcarbamoylase (ATCase) was quantified by ELISA (*n* = 3). (E) Kinetic analysis by BLI of binding of Py‐27 to ATCase. (F) Cellular thermal shift assays of ATCase protein with or without Py‐27 treatment (*n* = 3). (G) Three‐dimensional binding diagram of the ATCase and Py‐27. (H) Magnified view of the binding site. (I) Two‐dimensional interaction diagram of the binding site. Data were presented as mean ± SD. Statistical significance was analyzed by ordinary one‐way ANOVA (^**^p < 0.01, ^***^p < 0.001).

The workflow for photoaffinity labeling and protein target identification is illustrated in the flowchart (Figure 7A). Probe labeling optimization in the MRSA (ATCC 43300) strain showed concentration‐dependent efficiency, and 250 µm Py‐27‐P was determined to be the optimal concentration (Figure S3A). Competitive experiments confirmed target specificity through dose‐responsive inhibition by Py‐27 (Figure S3B). Potential target proteins were labeled via a click reaction between the Py‐27 probe and biotin azide, followed by enrichment using streptavidin magnetic beads. Based on the mass spectrometry analysis results, 64 proteins with high peptide matching scores were identified from the MRSA strain, both in situ and in lysates. Py‐27 primarily affects translation, carbohydrate metabolism, lipid metabolism, and nucleotide metabolism (Figure S4A, B). The top ten candidate targets with the highest scores are shown (Table 1). Molecular docking was employed to evaluate the binding capability of compound Py‐27 to these top‐ranked target proteins (Figure S5A), with binding energies ranging from −41.8978 to −14.5208 kJ/mol (Table 1). Furthermore, the impact of Py‐27 on the expression of genes encoding these target proteins was assessed by q‑PCR (Figure S5B). Among them, the expression of *pyrB* was the most significantly downregulated (Table 2). Integrating the results from molecular docking and q‑PCR, we hypothesize that Py‐27 exerts its antibacterial activity by targeting the ATCase protein (encoded by *pyrB*) and disrupting its function.

**TABLE 2:** Top ten possible target proteins were identified by mass spectrometry, molecular docking and Q‐PCR.

Accession	Gene	MW [kDa]	Unique Peptides	Coverage [%]	‐CDOCKER INTERACTION ENERGY	Relative gene expression
Q2FW22	*rplR*	13.1	11	71	30.106	0.62
Q2FZ25	*rpsB*	29.1	13	49	24.2083	0.57
Q2G0G1	*adh*	36	11	36	41.6439	0.49
Q2FXM8	*pfkA*	34.8	8	30	14.5208	0.59
Q2FWF5	*fabZ*	16.1	6	27	27.4015	0.69
Q2FYJ3	*tdcB*	37.3	7	27	36.8046	0.23
Q2FZ75	*pyrB*	33.2	6	22	40.7895	0.19
Q2G224	*deoC*	23.5	4	19	37.2383	0.25
Q2FUX3	*isaB*	19.4	4	18	29.0695	0.37
Q2FZQ3	*fabI*	28	4	14	41.8978	1.00

To further confirm the potential of ATCase as an antibacterial target, we constructed an MRSA (ATCC 43300) Δ*pyrB* mutant strain via allelic replacement. Successful deletion of the *pyrB* gene was confirmed by PCR (Figure 7B) and DNA sequencing (Figure S6), and the deletion of *pyrB* significantly impaired bacterial growth (Figure 7C). Due to the unsuccessful complementation of the Δ*pyrB* mutant, we employed an exogenous metabolite rescue strategy as an alternative to genetic complementation. Compared to the M9 medium containing only basic nutrients, the TSB medium, which is rich in exogenous pyrimidines or precursors, supported a higher growth rate and yield during the logarithmic growth phase. In the M9 medium, the Δ*pyrB* mutant exhibited significant changes in growth characteristics compared to the wild‐type (WT), revealing a lag phase approximately 2 h longer and a significantly reduced final cell density. However, when incubated in the nutrient‐rich TSB medium, bacterial growth nearly recovered to wild‐type levels, except for the prolonged 2 h lag phase. This indicates that the provision of exogenous nucleotide sources in the nutrient‐rich medium restored the growth of the mutant [42].

Furthermore, the MIC values of Py‐27, vancomycin, and ampicillin against MRSA showed unexpected results after the mutation. The MIC of Py‐27 decreased from 0.125 µg/mL in the WT to 0.03125 µg/mL in the Δ*pyrB* mutant strain (Figure S7A). The MIC of ampicillin decreased from 256 µg/mL in the WT to 16 µg/mL in the Δ*pyrB* mutant (Figure S7B). In contrast, the MIC of vancomycin remained unchanged (Figure S7C). This result indicates that for vancomycin, the drug's target and mode of action did not significantly change between the WT and Δ*pyrB* mutant strains. Bacterial resistance arises from the synergistic action of complex mechanisms, including enzymatic inactivation, target alteration, drug efflux, and reduced membrane permeability [43]. The link between virulence factors such as efflux pumps and biofilms and resistance has been extensively studied [44, 45]. For ampicillin, although the in vitro MIC showed a significant decrease in the Δ*pyrB* mutant, the strain still exhibited resistance to MRSA. This suggests that the primary resistance mechanism for ampicillin remains intact in the Δ*pyrB* mutant. The observed MIC reduction may be due to alterations in auxiliary factors that influence the resistance level of MRSA. The nucleotide biosynthesis pathway is not only central to bacterial survival and proliferation but also closely linked to virulence factors essential for pathogenesis [26]. This implies that in the Δ*pyrB* mutant, the deficiency in nucleotides caused by the disruption of the *de novo* pyrimidine synthesis pathway affects multiple metabolic pathways and biological functions within the bacterium, leading to a weakening of bacterial virulence factors [46].

These results indicate that ATCase, encoded by the *pyrB* gene, is crucial for the normal proliferation and virulence factors of MRSA, making it a highly promising antibacterial target. Simultaneously, these results also suggest that the antibacterial activity of Py‐27 was partly realized by the inhibition of ATCase.

### 2.7 ATCase Binding by Py‐27

Aspartate transcarbamylase (ATCase), encoded by the *pyrB* gene, is a key regulatory enzyme in the second step of *de novo* pyrimidine synthesis in most organisms [47]. It mediates the carbamylation reaction between L‐aspartate (Asp) and carbamyl phosphate (CP), producing N‐carbamyl‐L‐aspartate (CA) and inorganic phosphate (Pi). The activity of ATCase is finely regulated by metabolites from the purine and pyrimidine metabolic pathways: cytidine triphosphate (CTP) acts as an allosteric inhibitor, while adenosine triphosphate (ATP) serves as an activator, binding to different sites on the regulatory subunits of the enzyme, respectively. This feedback mechanism ensures the metabolic balance between purine and pyrimidine synthesis and plays a crucial role in maintaining cellular homeostasis and supporting key biological processes. To initially verify whether Py‐27 binds to ATCase and inhibits its biological activity, we designed a series of experiments based on the property that this protein is regulated by the allosteric inhibitor CTP, combined with liquid chromatography‐mass spectrometry (LC‐MS) technology. Based on HPLC retention time and mass spectrometric fragmentation patterns, we performed quantitative analysis of N‐carbamoyl‐L‐aspartate (Figure S8). The results showed that Py‐27 at concentrations of 1/2 × MIC and 1/4 × MIC significantly reduced the level of N‐carbamoyl‐L‐aspartate, with an inhibitory effect stronger than that of CTP. The inhibitory activity of Py‐27 and CTP against ATCase were further validated using an ELISA assay. Py‐27, at both 1/2 × MIC and 1/4 × MIC concentrations, significantly inhibited ATCase activity, with an effect stronger than that of CTP (Figure 7D). After obtaining purified ATCase protein through expression and purification (Figure S9), the binding affinity of Py‐27 to the ATCase protein was studied using kinetic and thermodynamic experiments. BLI results showed a binding rate constant (K_on_) of 1.43 × 10^4^/Ms and a dissociation rate constant (K_off_) of 5.20 × 10^−2^/s. The equilibrium dissociation constant (K_D_) calculated from the ratio of K_off_ to K_on_ was 3.63 × 10^−6^ m, indicating that compound Py‐27 has a strong affinity for the target protein (Figure 7E). As further confirmation of binding, the Cellular Thermal Shift Assay (CETSA) was used to observe the intracellular binding of Py‐27 to the ATCase protein. Results similar to the BLI assay were obtained, showing that Py‐27 binds to ATCase and alters its thermal stability (Figure 7F).

### 2.8 Binding Mode and Stability of the ATCase and Py‐27

To further elucidate the binding mode of Py‐27 with ATCase, we performed molecular docking using the crystal structure of the ATCase protein (PDB ID: 6PNZ). The results revealed that Py‐27 forms a stable complex with the ATCase protein through multiple non‐covalent interactions (Figure 7G–I). Py‐27 is located within a binding cavity formed by residues such as THR49, ARG50, ARG160, VAL161, ARG209, and ARG210. It forms a halogen‐hydrogen bond with ARG160, a cation‐*π* interaction with ARG210, and multiple hydrogen bonds with THR49, ARG50, and ARG210. These interactions collectively enhance the directionality and stability of the binding.

Molecular dynamics (MD) simulations further validated the stability of the complex. Analyses of the root mean square deviation (RMSD), root mean square fluctuation (RMSF), and radius of gyration (Rg) for the ATCase and Py‐27 complex indicated that it maintained a compact and stable conformation during the simulation, with low fluctuations in the core structure (Figure S10A–C). The average number of hydrogen bonds throughout the simulation was 0.23, with few and transient amino acid residues involved, suggesting that hydrogen bonding is not the primary driving force for binding (Figure S10D). Ramachandran analysis revealed no significant structural transitions overall, demonstrating high conformational stability of ATCase during the simulation (Figure S10E). Free energy landscape (FEL) analysis showed that the complex transitioned from a high‐energy, unstable conformation (start) to a low‐energy, stable conformation (end) during the simulation (Figure S10F–G). Structurally, the core conformations of the three states showed minimal differences, all exhibiting a stable binding mode with the protein enveloping the ligand, with only minor displacements observed at the solvent‐exposed ends, supporting the binding stability of the simulated system. MM/GBSA binding free energy calculation showed a total binding free energy of −29.60 kcal/mol for the complex, with van der Waals interactions (−42.86 kcal/mol) and electrostatic interactions (−15.86 kcal/mol) identified as the primary driving forces for binding (Figure S10I). Residue energy decomposition analysis revealed that key residues, including ASN47 (−1.54 kcal/mol), ARG210 (−1.71 kcal/mol), and PRO254 (−1.84 kcal/mol), contributed significantly to the binding (Figure S10H).

Overall, the ATCase and Py‐27 complex exhibits synergistic effects of multiple interaction forces at the molecular level, demonstrating high stability and strong binding affinity in both kinetic and energetic terms.

### 2.9 Bactericidal Mechanism of Py‐27

**FIGURE 8:**
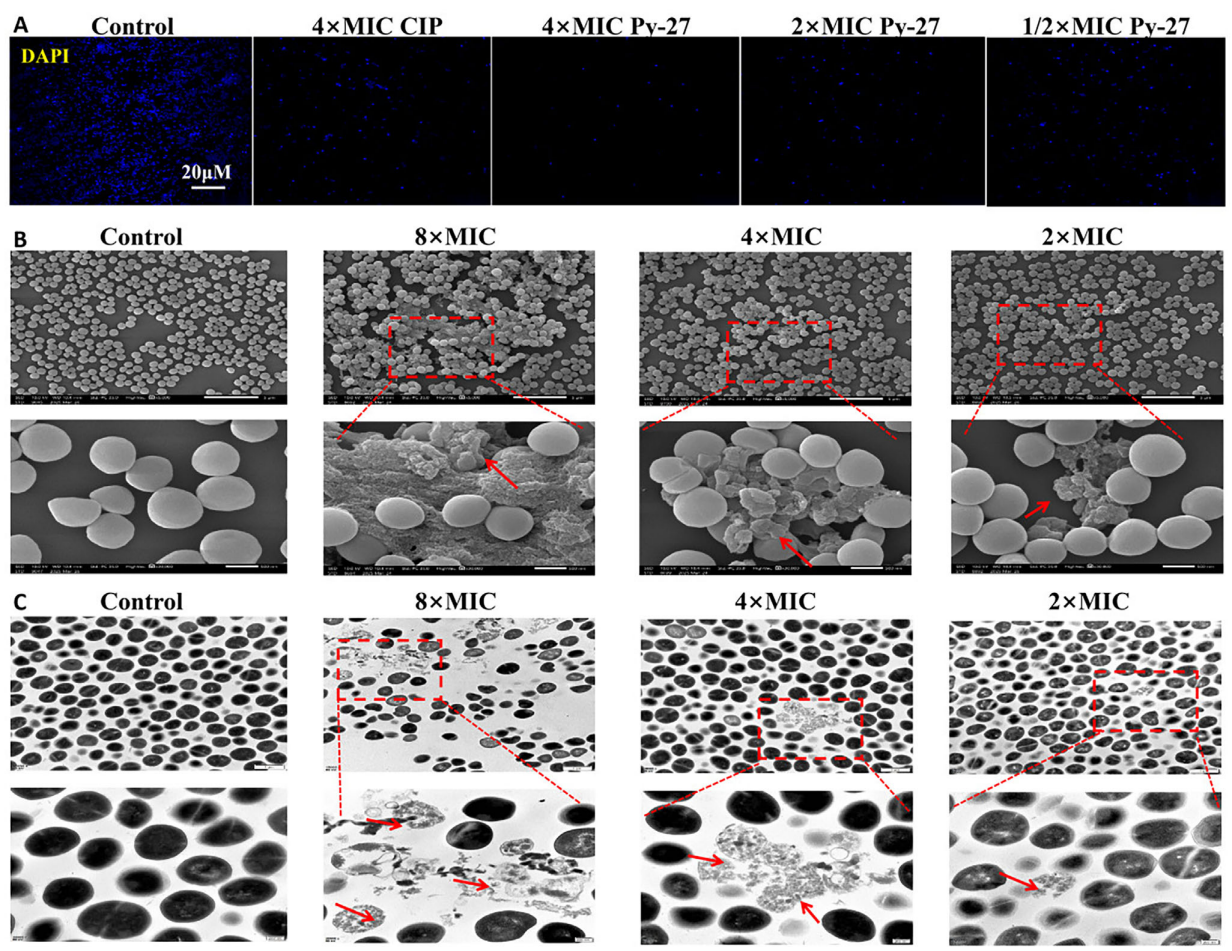
Bactericidal mechanism of Py‐27. (A) DAPI staining fluorescence photographs of MRSA after treatment with Py‐27. (B) Representative SEM images (top) and corresponding higher magnification images (bottom) of MRSA in different groups. (C) Representative TEM images (top) and corresponding higher magnification images (bottom) of MRSA in different groups.

ATCase is a key enzyme in the first committed and irreversible step of the *de novo* pyrimidine synthesis pathway, playing a crucial regulatory role in pyrimidine metabolism. The pyrimidine nucleotides produced are fundamental building blocks for DNA and RNA. Inhibition of DNA and RNA synthesis is the most direct consequence of disrupting the *de novo* pyrimidine synthesis pathway. Compared with the control group, the DNA content of MRSA was significantly reduced after treatment with ciprofloxacin (which exerts bactericidal activity by inhibiting bacterial DNA synthesis) and Py‐27 at 1/2 × MIC. This indicates that Py‐27 interferes with pyrimidine metabolism, leading to the inhibition of DNA synthesis (Figure 8A, relevant quantitative bar charts are provided in Figure S13B). Inhibition of DNA synthesis represents a well‐established antibacterial mechanism [48].

Furthermore, Py‐27 is a highly effective bactericidal compound. Bactericidal effects are often achieved by generating harmful endogenous reactive oxygen species (ROS), leading to oxidative damage to the cell [49, 50, 51]. We observed the cell surface changes induced by Py‐27 using scanning electron microscopy (SEM) and transmission electron microscopy (TEM). Untreated MRSA exhibited a smooth, uniform spherical morphology with an intact cell wall, a defined membrane, and homogeneous, dense cytoplasm, capable of forming a septum for division (Figure 8B, C). In contrast, Py‐27 treatment caused severe surface deformation, including shrinkage, collapse, or fusion, along with a clear loss of membrane integrity and leakage of cellular contents (Figure 8B, C). Transcriptomic results analysis revealed 278 differentially expressed genes (DEGs) upon Py‐27 treatment, with 167 upregulated and 117 downregulated (Figure S11A). Gene Ontology (GO) enrichment analysis indicated that Py‐27 could affect processes such as uridine monophosphate (UMP) biosynthesis and metabolic processes (Figure S11B), while Kyoto Encyclopedia of Genes and Genomes (KEGG) analysis highlighted impacts on arginine biosynthesis, ABC transporters, and pyrimidine metabolism (Figure S11C). Moreover, for most bacteria, the *de novo* pyrimidine synthesis pathway is the primary source of pyrimidine nucleotides, which are essential for numerous cellular functions, including DNA replication, energy storage, and signaling [26]. Inhibition of pyrimidine biosynthesis could consequently affect several key metabolic pathways and functions, including arginine biosynthesis [52], carbohydrate metabolism [53], and lipid metabolism [54]. Therefore, by inhibiting ATCase and disrupting pyrimidine metabolism, Py‐27 likely induces profound metabolic dysregulation, which contributes to its bactericidal effect by triggering oxidative stress.

**FIGURE 9:**
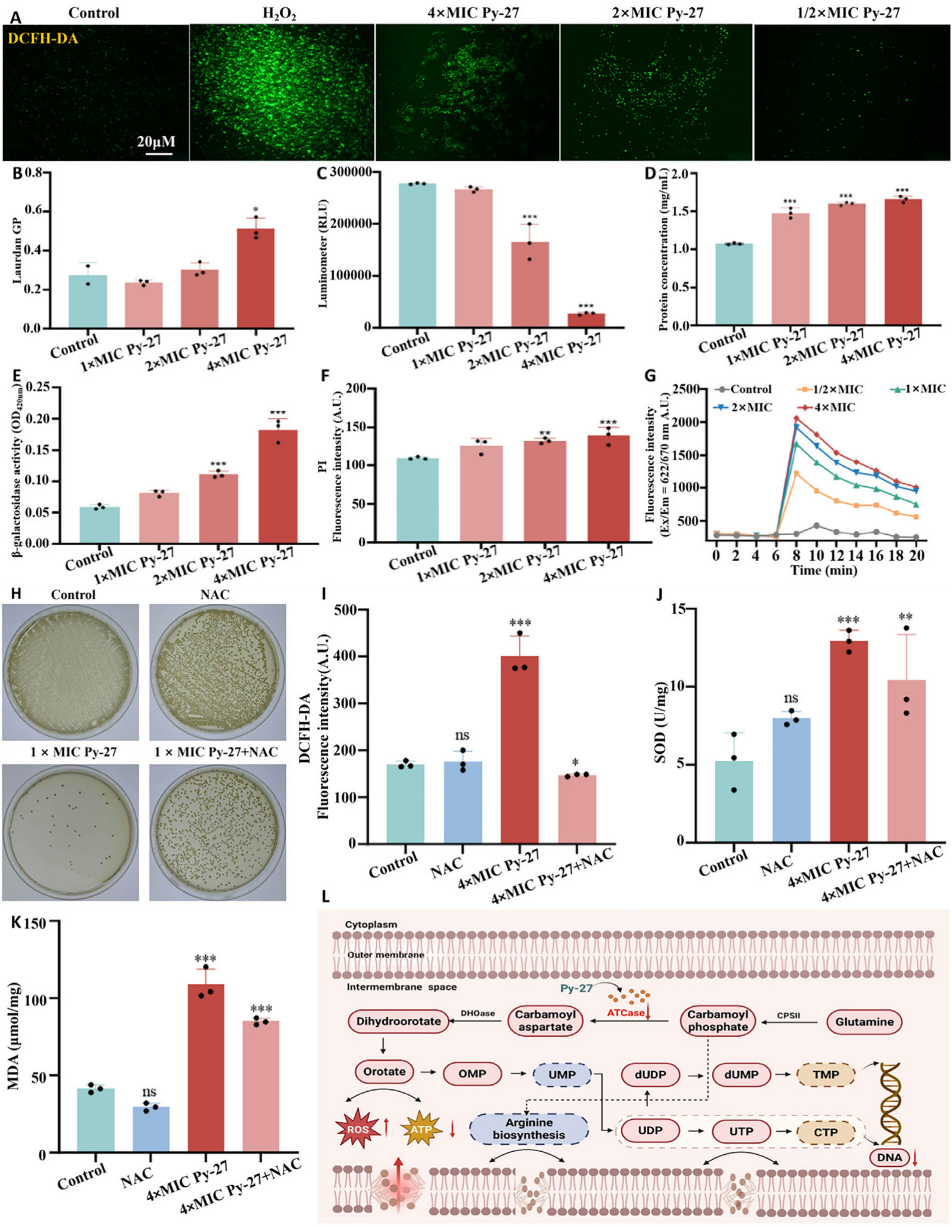
Py‐27 promotes oxidative damage and induces cell death. (A)DCFH‐DA staining fluorescence photographs of ROS (green) of MRSA after treatment with Py‐27. (B) Membrane fluidity with the fluorescent probe Laurdan after corresponding treatments (*n* = 3). (C) ATP concentration after corresponding treatments (*n* = 3). (D) Protein leakage for MRSA after corresponding treatments (*n* = 3). (E) Levels of β‐galactosidase after corresponding treatments (*n* = 3). (F) PI was used to measure cell membrane permeability after corresponding treatments (*n* = 3). (G) The cell membrane depolarization was tested by DiSC3(5) after corresponding treatments (*n* = 3). (H) Photographs and of MRSA colonies after treatment with the Py‐27 and NAC. (I) ROS levels after treatment with the Py‐27 and NAC (*n* = 3). (J) SOD activity (*n* = 3). (K) MDA levels (*n* = 3). (L) Schematic representation of the proposed mechanism by which Py‐27 inhibits DNA synthesis and induces oxidative damage. Created with BioRender.com. Data were presented as mean ± SD. Statistical significance was analyzed by ordinary one‐way ANOVA (^*^p < 0.05, ^**^p < 0.01, ^***^p < 0.001; and ns, not significant, respectively).

Given that disruption of membrane homeostasis often contributes to reactive oxygen species (ROS) accumulation, we first assessed membrane fluidity using the fluorescent probe Laurdan. Py‐27 treatment significantly altered membrane fluidity, indicating a disruption of membrane homeostasis (Figure 9B). This was accompanied by a dose‐dependent decrease in intracellular ATP levels (Figure 9C) and a concurrent dose‐dependent increase in intracellular ROS content (Figure 9A). Subsequently, the concentration of proteins in the bacterial extracellular content showed an increasing trend after treatment with different concentrations of Py‐27 (4 × MIC, 2 × MIC, and MIC), confirming the loss of cell membrane integrity (Figure 9D). Furthermore, the activity of β‐galactosidase, a tetrameric enzyme detectable only upon membrane damage, also exhibited a dose‐dependent significant increase following Py‐27 treatment (Figure 9E). Correspondingly, compared to the control, the PI fluorescence intensity in the Py‐27‐treated group increased significantly, indicating enhanced membrane permeability (Figure 9F), while a sharp increase in DiSC_3_(5) fluorescence intensity indicated a decrease in membrane potential (Figure 9G).

Furthermore, to clarify the dominant mechanism underlying the bactericidal action of Py‐27, we performed verification assays using the ROS scavenger NAC. MIC results showed that NAC alone exhibited no antibacterial activity. Unexpectedly, the combination of Py‐27 and NAC did not markedly alter the antibacterial potency of Py‐27, and the MIC value remained at 0.125 µg/mL. Subsequent colony counting (Figure 9H) revealed that the bacterial number in the Py‐27 + NAC group was higher than that in the Py‐27 monotherapy group, suggesting that NAC supplementation partially alleviated the bactericidal effect of Py‐27 against MRSA.

DCFH‐DA staining (Figure 9I) demonstrated that Py‐27 treatment remarkably elevated the fluorescence intensity in MRSA (*p* < 0.001), indicating obvious intracellular ROS accumulation. In contrast, the fluorescence intensity was distinctly decreased in the Py‐27 + NAC group and was comparable to that of the control group, confirming that NAC efficiently eliminated excessive intracellular ROS (*p* < 0.05). Collectively, these findings indicated that the bactericidal activity of Py‐27 was not fully ROS‐dependent.

Based on the observed membrane damage, we further determined the levels of malondialdehyde (MDA), a marker of lipid peroxidation, and superoxide dismutase (SOD), a core endogenous antioxidant enzyme, to verify whether ATCase inhibition by Py‐27 triggered oxidative stress and oxidative damage. The results (Figure 9J,K) showed that compared with the untreated control, Py‐27 significantly increased MDA content and SOD activity (*p* < 0.001), which confirmed that ATCase inhibition mediated by Py‐27 induced severe oxidative damage. Notably, both MDA content and SOD activity remained significantly elevated in the Py‐27 + NAC group, illustrating that exogenous NAC could not fundamentally block intracellular oxidative stress but only scavenged excessive ROS generated in the early stage of metabolic disturbance.

Combined with the time‐kill curve of Py‐27 (Figure 2C), treatment with 4 × MIC Py‐27 for 4 h only achieved partial bacterial killing without entering the logarithmic bactericidal phase. At this stage, ATCase inhibition by Py‐27 induced metabolic disorders, accompanied by transient ROS accumulation. Therefore, NAC could effectively eliminate early accumulated ROS but failed to reverse the sustained increases in MDA and SOD activity. Moreover, the MIC results and colony count data of the Py‐27+NAC group further demonstrate that the inhibition of pyrimidine metabolism targeting ATCase is the initial and core mechanism of action of Py‐27, while oxidative damage acts as a time‐dependent secondary effect. This secondary effect accumulates with prolonged exposure time. Therefore, the Py‐27+NAC combination can only partially alleviate the bactericidal activity of Py‐27 against MRSA, rather than completely inhibiting it.

Taken together, these results indicate that Py‐27 inhibits ATCase, thereby disrupting the de novo pyrimidine synthesis pathway. This in turn affects DNA synthesis, induces intracellular metabolic disturbance, and triggers oxidative stress, leading to excessive accumulation of reactive oxygen species. Simultaneously, it weakens ATP production and reduces antioxidant capacity. This cascade of reactions ultimately results in cell membrane rupture, fusion, and altered permeability, thereby exerting its bactericidal effect (Figure 9L). Such multifaceted effects may lead to more severe oxidative injury, thereby enhancing bactericidal efficacy and potentially preventing resistance development caused by a single factor.

## 3 Discussion

The declining efficacy of existing antibiotics and the stagnation in the development of new drugs make the discovery of novel antimicrobial agents with new mechanisms of action, capable of overcoming resistance and improving therapeutic outcomes, particularly important and urgent [55].

To date, the pyrazole core has been utilized in the development of various veterinary drugs, pesticides, and human medicines, such as the anti‐parasitic agent fipronil, the insecticide pyrazophos, the fungicide pyraclostrobin, the anti‐inflammatory analgesic celecoxib, and the lung cancer therapeutic crizotinib. Therefore, pyrazole is considered a promising and versatile scaffold for drug development [12]. Its unique structure allows for extensive substitution, enabling the modulation of diverse biological responses [11]. Notably, several published studies have demonstrated that different substituted pyrazole derivatives exhibit distinct anti‐MRSA mechanisms [56, 57, 58, 59, 60], highlighting the potential of the pyrazole scaffold's rich modification sites for discovering novel antibacterial lead compounds with new targets and mechanisms. However, systematic studies that bridge phenotypic confirmation to mechanistic discovery, particularly the identification and validation of antibacterial targets, remain scarce.

Based on the important role of the amide pharmacophore in bioactive molecules, we designed and synthesized a series of pyrazole‐amide hybrids, enabling the generation of new structures through a simple synthetic route, and investigated their structure‐activity relationship. Notably, the promising lead compound Py‐27 exhibited excellent bactericidal activity against Gram‐positive bacteria, including MRSA, with an efficiency superior to vancomycin. Compared to previously reported antibacterial pyrazole compounds, the pyrazole‐amide derivative discovered in this study exhibits superior in vitro and in vivo anti‐MRSA activity, a low propensity for resistance development, and a favorable safety profile. More importantly, this work integrates phenotypic validation with mechanistic discovery‐specifically, the identification and verification of the antibacterial target‐in a systematic investigation, and for the first time confirms ATCase as a viable target for antimicrobial drugs.

However, due to its inability to penetrate the outer membrane of Gram‐negative bacteria, it shows no significant activity against them. This specificity of Py‐27 may help protect beneficial Gram‐negative flora in clinical settings [61, 62]. Of course, this also limits the broad impact and application scope of Py‐27. Therefore, subsequent studies will continue to explore combination therapy strategies to expand the application range of Py‐27. The propensity for resistance development is a critical aspect to evaluate in the development of new antibacterial agents. Py‐27 demonstrated a low tendency for resistance development and exhibited significant activity against clinically resistant strains. This suggests that, compared to traditional antibiotics, Py‐27 may possess a different mechanism of action or exert its antibacterial effect through a combination of multiple mechanisms. Chemical proteomics is an effective strategy for revealing the targets of small‐molecule compounds [63, 64].

The *de novo* pyrimidine synthesis pathway is essential for bacterial growth. Particularly in the blood, the lack of nucleotide precursors, rather than other nutrients, is a key limiting factor for bacterial growth in serum [42]. This indicates that *de novo* nucleotide biosynthesis is the single most critical metabolic function for bacterial growth in the blood, suggesting that the corresponding enzymes are potential antibacterial targets for treating blood infections [23]. Concurrently, both inhibiting the *de novo* pyrimidine synthesis pathway [24] and hijacking the pyrimidine salvage pathway [65] have been shown to inhibit the growth of *S. aureus*. In this study, by constructing an MRSA Δ*pyrB* mutant strain, we confirmed that a defect in *pyrB* (the gene encoding ATCase) impairs MRSA growth. However, this mutational defect was overcome in a nutrient‐rich environment that provides exogenous pyrimidines or precursors, a result consistent with observations in *E. coli* Δ*pyrB* mutants [42]. This confirms that ATCase is indeed a viable drug target against MRSA. Furthermore, comprehensive computational characterization confirmed a high‐affinity, specific interaction between Py‐27 and ATCase. Molecular docking revealed a binding mode stabilized by multiple non‐covalent interactions that enhance directionality and stability. Crucially, molecular dynamics simulations demonstrated the stability of the complex, and MM/GBSA calculations yielded a total binding free energy of −29.60 kcal/mol, primarily driven by van der Waals and electrostatic interactions. These computational predictions were strongly validated by experimental evidence. Py‐27 significantly inhibited ATCase activity and reduced the level of N‐carbamoyl‐L‐aspartate, confirming its dose‐dependent binding. BLI results showed an equilibrium dissociation constant (K_D_) of 3.63 × 10^−6^ m, indicating a strong affinity of compound Py‐27 for ATCase. The CETSA results showed that Py‐27 binds to ATCase and alters its thermal stability. The convergence of computational and experimental evidence provides strong support for ATCase being the primary target of Py‐27. Moreover, we found that Py‐27 inhibits DNA synthesis, which is the most direct consequence of disrupting the *de novo* pyrimidine synthesis pathway. Simultaneously, the bactericidal effect of Py‐27 is mediated by a ROS‐induced oxidative damage mechanism, indicating a close link between the *de novo* pyrimidine synthesis pathway and the bacterial redox balance [66]. Additionally, the MIC of Py‐27 against the Δ*pyrB* mutant decreased to 0.03125 µg/mL compared to the wild‐type (WT), suggesting that Py‐27 combats MRSA through multiple pathways and providing a rationale for its low propensity for resistance development.

The *de novo* pyrimidine synthesis pathway is also crucial for bacterial virulence factors [26]. These factors are key elements that enable pathogens to infect the host and cause disease, including adhesion factors, invasion factors, toxins, and immune evasion factors. The *pyr*‐operon regulated *de novo* pyrimidine synthesis pathway has been shown to be associated with *S. aureus* colonization [25]. In *E. coli* mutants with a defective *pyrB* gene, transcription of the csgDEFG operon is inhibited, impairing the production of curli fibers and cellulose, key components of the biofilm matrix, thereby preventing the production of the master biofilm regulator protein CsgD [67]. In *P. aeruginosa* with a defective *pyrE* gene, inhibition of the pyrimidine synthesis pathway leads to a significant reduction in motility and biofilm formation [46]. Coumarin derivatives have been shown to inhibit MRSA biofilm formation by targeting *PyrR* in the *de novo* pyrimidine synthesis pathway [68]. In this study, Py‐27 not only inhibited MRSA biofilm formation but also effectively eradicated mature MRSA biofilms, making them more sparse and thinner, and effectively killed the bacteria within the mature biofilm, demonstrating significant anti‐biofilm activity. Interestingly, the MIC of ampicillin decreased from 256 µg/mL in the wild‐type (WT) to 16 µg/mL in the Δ*pyrB* mutant strain. Although the in vitro MIC of the Δ*pyrB* mutant strain was significantly reduced, the strain remained resistant to ampicillin, indicating that the primary resistance mechanism for ampicillin is still present in the Δ*pyrB* mutant. Furthermore, studies have found that even in the absence of the *ampC* gene, strains can achieve high‐level resistance to ampicillin through a combination of single‐point mutations in multiple genes involved in efflux pumps, transcriptional regulators, and porins, even in the absence of ampicillin exposure [69]. Moreover, the critical role of efflux pumps in the development of resistance and in virulence factors such as biofilms has been well‐established [45, 70, 71]. Therefore, targeting ATCase to inhibit the *de novo* pyrimidine synthesis pathway may affect the activity of efflux pumps, thereby influencing efflux pump‐mediated resistance and virulence factors like biofilms. Our research provides a new strategy for mitigating the resistance of existing antibacterial drugs.

Given the significant antibacterial activity of Py‐27 and its potential to effectively mitigate resistance development, further in‐depth work is required to expand its antibacterial spectrum against both standard and resistant strains. Our study demonstrates that Py‐27 targets ATCase, representing an antibacterial mechanism of action distinct from currently used clinical antibiotics. Of course, we cannot rule out the possibility that Py‐27 may affect other biological pathways and molecular targets. Safety is a critical indicator and prerequisite for the clinical application of antibacterial drugs. The evaluation results of hemolytic activity, acute toxicity, subchronic toxicity, and genotoxicity indicate that Py‐27 has a favorable safety profile. Its therapeutic efficacy was further evaluated through in vivo studies. In both the skin infection model and the acute pneumonia infection model, Py‐27 not only demonstrated superior therapeutic effects compared to vancomycin but also exhibited the ability to promote skin repair and suppress inflammatory responses. Although Py‐27 has shown potential as an antibacterial candidate, further research is needed to evaluate its efficacy in different biological environments and against various MRSA infectious diseases, and to expand the scope of side effect investigations. Furthermore, the environmental pollution caused by antibiotics is an issue that cannot be ignored. It is particularly important to conduct an environmental risk assessment for Py‐27, including its degradability in aquatic and soil environments, the health risks to biological communities, and the potential transfer of resistance genes.

## 4 Conclusion

In summary, our research results indicate that Py‐27 exhibits potent bactericidal activity against methicillin‐resistant *Staphylococcus aureus* (MRSA) standard strains and clinically isolated strains, with a low tendency to induce drug resistance. Its antibacterial mechanism of action is believed to be based on binding to ATCase, thereby activating multiple pathways. Py‐27 is expected to be an ideal candidate drug for the development of novel therapies against MRSA‐related infections.

## 5 Methods

All research complies with the relevant ethical regulations. Study protocols were approved by the Animal Welfare and Ethics Committee of Lanzhou Institute of Husbandry and Pharmaceutical Sciences Chinese Academy of Agricultural Sciences (Lanzhou, China) for all animal experiments.

### 5.1 Bacteria and Reagents

Bacterial strains and plasmids used in this study are listed in Table S1. Antibiotics namely vancomycin, ciprofloxacin and ampicillin were purchased from Shanghai Macklin Biochemical Technology Co., Ltd (Shanghai, China). M9 minimal medium were purchased from Beijing Leagene Biotechnology Co., Ltd (Beijing, China).

### 5.2 Animals

Pathogen‐free male BALB/c mice, with body weights ranging from 18 to 22 g, were provided by Lanzhou Veterinary Research Institute, Chinese Academy of Agricultural Sciences. All mice were kept in a specific‐pathogon‐free grade laboratory at room temperature (20°C–25°C) and constant humidity (40%–70%) under a 12 h light‐dark cycle. The standard diet and water were provided ad libitum. The animal study was reviewed and approved by the Institutional Animal Care and Use Committee of Lanzhou Institute of Husbandry and Pharmaceutical Science, Chinese Academy of Agricultural Sciences (Approval No. 2025–65).

### 5.3 General Information for Chemical Synthesis

All chemical reagents and solvents were purchased from commercial suppliers and used without further purification. The progress of all reactions was monitored by thin‐layer chromatography (TLC). Purification of compounds was performed by column chromatography on silica gel. Melting points were determined using a JHS‐5 melting point apparatus (Shanghai Jiahang Instrument Co., Ltd.). High‐resolution mass spectrometry (HRMS) data were recorded on a Q Exactive‐Vanquish Core HPLC (Thermo Fisher Scientific). ^1^H and ^13^C nuclear magnetic resonance (NMR) spectra were recorded on Bruker‐AM‐400 MHz and Bruker‐DMX 500 MHz spectrometers, respectively, using tetramethylsilane (TMS) as an internal standard. Detailed information on the synthesis and characterization of the compounds used in this study is provided in the Supporting Information.

### 5.4 Growth Curves of Bacteria

The bacterial suspensions of MRSA (ATCC 43300) strains were treated with different concentrations of Py‐27 (1/4 × MIC, 1/2 × MIC, 1 × MIC, 2 × MIC, and 4 × MIC) and VAN (vancomycin, 1/4 × MIC) at 37°C with continuous shaking (180 rpm). Several samples were taken at various times (0, 2,4, 6, 8, 10, 12, and 24 h) during the culture phase in order to measure and record the OD_600_ using EnSpire2300 microplate reader.

### 5.5 Time‐Kill Curves

MRSA (ATCC 43300) suspensions were incubated at 37°C with shaking (180 rpm). The bacteria were treated with Py‑27 at 1×, 4×, and 8× MIC. Diluted bacterial suspensions of the mixture at different time points (0, 2, 4, 6, 8, 10, 12, 24 h) were plated on TSA plates to count the number of surviving bacteria. The sterilizing kinetic curve was generated by counting the bacterial colonies.

### 5.6 Live/Dead Bacterial Staining

Bacterial viability was assessed using a Live/Dead Bacterial Staining Kit. MRSA (ATCC 43300) suspensions (1 × 10^8^ CFU/mL) were washed, treated with Py‐27 (1 × MIC, 2 × MIC) and VAN (2 × MIC) for 2 h, and then stained with SYTO 9 and propidium iodide for 20 min in the dark. After washing, samples were examined by CLSM(ZEISS LSM800).

### 5.7 MIC and MBC Determination [72]

The minimum inhibitory concentrations (MICs) of the tested compounds were measured by the standard broth microdilution assay as previously describe. Briefly, compounds were serially diluted twofold in MHB, and equal volumes of the bacterial suspension in logarithmic growth phase (1 × 10^6^ CFU/mL) were added into 96‐well plates. After 16 h of incubation at 37°C, the MIC was defined as the lowest concentration of compounds with no visible growth. The minimum bactericidal concentration (MBC) was the lowest compound concentration at which liquid from the clarified wells of the MIC assay was applied to the TSA plate and incubated at 37°C for 24 h without bacterial growth.

### 5.8 Outer Membrane Permeability [73]

The logarithmically growing *E. coli* bacterial suspension (1 × 10^8^ CFU/mL) were added to black 96‐well microplates preloaded with 10 µm NPN together with PMBN (256 µg/mL), Py‐27 (4 × MIC), or their combination. Following incubation at 37°C for 1 h under light‐protected conditions, fluorescence was recorded at 350 nm excitation and 420 nm emission.

### 5.9 Bacterial Resistance Studies [74]

The resistance development assay was performed by serially passaging MRSA (ATCC 43300) in MHB containing 1/2 × MIC levels of Py‐27, VAN and CIP (Ciprofloxacin). Daily for 20 days, cultures from the previous day's second‑highest concentration with visible growth were used to determine the new MIC via broth microdilution and to inoculate the next passage.

### 5.10 In Vitro Antibiofilm Capacity [75]

Biofilm inhibition and eradication assays were performed using MRSA (ATCC 43300) suspensions (5 × 10^7^ CFU/mL). For inhibition assays, suspensions were treated with Py‑27 (1/2×, 1×, or 4× MIC) or vancomycin (4× MIC) for 24 h before biofilm formation. For eradication assays, mature biofilms were first established by 24 h static incubation and then treated with the same compound concentrations. After treatment, mixed solutions were removed, and biofilms were gently washed with PBS. Biofilm biomass was quantified by crystal violet staining, and viable bacteria in the biofilms were counted. Three‐dimensional biofilm architecture was visualized using CLSM (ZEISS, LSM800).

### 5.11 Hemolysis Assay of Py‐27 [76]

A 2% (v/v) suspension of sheep erythrocytes in sterile saline was prepared. Then, 100 µL of the suspension was mixed with 100 µL of Py‐27 (at various concentrations in saline) in a 96‐well plate. Triton X‐100 (0.1%) and saline served as the positive and negative controls, respectively. After incubation at 37°C for 3 h, the samples were centrifuged (1200 g, 15 min), and the absorbance of the supernatant was measured at 540 nm to determine hemolysis (1).

(1) $$\text{Hemolysis rate}=\frac{\text{OD540}(\text{Py-27})-\text{OD540}(\text{Negative})}{\text{OD540}(\text{Positive})-\text{OD540}(\text{Negative})}\times 100\%$$

### 5.12 Acute Oral Toxicity [77]

LD_50_ was determined through an acute oral toxicity assay performed following Korbor's method under the maximal feasible dose guideline of GB 15193.3‐2014. Twenty‐four SPF grade BALB/c mice of 18∼22 g were selected. After 5 days acclimation, mice were randomly divided into four groups, with six mice in each group (three males and three females). Py‐27 was administered at the doses of 1.8, 1.2, 0.6, 0.4 g/kg, respectively, and the mice were gavaged orally with a fasting period of 12 h before gavage and no water was prohibited. All surviving mice were monitored regularly for any indications of toxicity for 14 days and subsequently euthanized.

### 5.13 30‐Day Feeding Test in Mice

Forty BALB/c mice, half male and half female, were randomly divided into four groups, solvent control group and three Py‐27 groups at low, medium and high doses (15, 30 and 60 mg/kg), and were fed by gavage orally for 30 consecutive days. The animals were weighed once a week during the experimental period and their weight gain was calculated. After 30 consecutive days of feeding, the animals were weighed after an overnight fast of 16 h. Blood was collected from the eyeballs and put to death for hematological, serum biochemical and histopathological examinations and determination of organ coefficients.

### 5.14 Salmonella Typhimurium Revert Mutation (Ames) Test [78]

The mutagenic potential of Py‐27 was evaluated using the Salmonella reverse mutation assay (Ames test) with the histidine‐deficient strains TA97A, TA98, and TA102. A standard spot test was performed by mixing 0.1 mL of overnight bacterial culture with 2 mL of top agar (maintained at 40°C) and, where required, 0.5 mL of S9 metabolic activation mixture. The mixture was poured onto minimal glucose agar plates and allowed to solidify. A sterile 6‐mm filter‐paper disc was placed on the surface of the top agar, and 10 µL of Py‐27 (5 mg/mL) or positive control was applied to the disc. Plates were incubated at 37°C for 48 h, after which the presence or absence of a mutagenic halo was recorded.

### 5.15 Mouse Sperm Morphology Test [78]

Fifteen SPF‐grade male BALB/c mice of 7∼12 weeks of age and weighing 25∼30 g were selected. The animals were purchased and environmentally acclimatized for 3∼5 days, and then randomly divided into three groups of 5 animals each, which were divided into Py‐27 administration group (0.43 g/kg), negative control group (soybean oil solution); positive control group (cyclophosphamide, 40 mg/kg). Animals in each group were gavaged at 2% volume of body weight. The animals were gavaged once a day for 5 consecutive days. The animals in each group were killed 35 days after the first injection of the samples, and the samples were prepared, fixed, stained with 2% eosin, and 1000 intact spermatozoa were examined microscopically in each mouse to calculate the malformation rate.

### 5.16 Mouse Bone Marrow Polymorphophilic Erythrocyte Micronucleus Test [78]

Thirty SPF‐grade BALB/c mice of 7∼12 weeks of age and weighing 25∼30 g were selected. Animals were purchased and environmentally acclimatized to feed for 3∼5 days, and then randomly divided into three groups of 10 mice (half male and half female) each, which were divided into the Py‐27 administration group (0.43 g/kg), the negative control group (soybean oil solution); and the positive control group (cyclophosphamide, 40 mg/kg). The drug was administered by gavage, twice, with 24 h interval between the two administrations, and the mice were executed by cervical dislocation method 6 h after the second administration of the drug. Femurs were taken and routinely sectioned. The slices were read by double‐blind microscopic method, and 1000 polyphilic erythrocytes (PCE) were observed in each mouse, and the number of polyphilic erythrocytes with micronuclei was recorded, and the micronucleus rate was calculated.

### 5.17 Mouse Skin Wound Infection Model [79]

Male BALB/c mice were adopted in the SPF laboratory environment for 1 week and randomly divided into six groups (Control, saline; Model, saline; VAN, 15 mg/kg; Py‐27, 5, 10, 15 mg/kg), 6 per group. 50 mg/kg cyclophosphamide was injected intraperitoneally before infection 24 h to obtain immunocompromised mice. On the day of the experiment, mice were anesthetized with urethane (20 mg/kg). The back hair of the mice was shaved with a depilatory cream and wounds of 5 mm in diameter were cut with scissors. 50 µL of PBS bacterial suspension containing 1.0 × 10^8^ CFU/mL MRSA (ATCC 43300) were inoculated in all groups except the Control group. Treatment was initiated 3 h after infection, while mice in the Model group received an equal volume of saline. For drug administration, Py‐27 and VAN were first dissolved in DMSO and then diluted with saline to the corresponding dosing concentrations, with the final DMSO concentration adjusted to 1%. Subsequently, 100 µL of the prepared drug solution was topically applied to the wound site once daily. The changes in wound and body weight were also recorded, and the mice were executed by cervical dislocation on day 12. Wound tissues, including all accumulated pus, were collected from 3 mice in each group, homogenized in sterile PBS and inoculated onto agar plates, which were placed in a thermostat for 24 h for colony counting. The remaining 3 skin samples were prepared and fixed in 4% paraformaldehyde for HE staining.

### 5.18 Mouse Model of Acute Pneumonia Infection [80]

Male BALB/c mice were adopted in the SPF laboratory environment for 1 week and randomly divided into six groups (Control, saline; Model, saline; VAN, 15 mg/kg; Py‐27, 5, 10, 15 mg/kg), 6 per group. Except for the blank control group, mice in each group were inoculated intranasally with 30 µL of 5 × 10^9^ CFU/mL MRSA (ATCC 43300) strains. The mice were placed vertically for 30 s. After 2 h of infection, Py‐27 and VAN were administered by intragastric administration every 8 h for a total of 24 h. After treatment, the mice were necropsied, and the organs (lungs, livers, kidneys, and spleens) were collected. The bacterial load in lung tissues was recorded. The levels of TNF‐α, CRP, MPO and MIP‐2 in lung tissues were determined by ELISA kit (Solarbio, Beijing, China). Lung, liver, kidney and spleen tissues were fixed with 4% paraformaldehyde for H&E staining.

### 5.19 Experimental Procedure for Activity‐Based Protein Profiling (ABPP) [81]

The protein supernatant of MRSA (ATCC 43300) strains was first prepared and then pretreated Py‐27‐P for 1 h at 37°C in incubators. After washed with PBS twice, the cells were cross‐linked with 365 nm UV light for 30 min at room temperature. Subsequently, the sample solutions were co‐incubated with the pre‐mixed click chemistry reaction conditions (500 mm TCEP, 50 mm TBTA, 500 mm CuSO_4_, 225 mm Biotin‐Azide) for 2 h at 25°C, then incubated with streptavidin beads at 4°C with vortexing overnight. Beads were washed sequentially with 500 µL PBS for 5 times. The proteins eluted from magnetic beads were analyzed by silver staining or Western blotting, and the different bands of silver staining were enzymatically hydrolyzed and removed silver ions, followed by LC‐MS/MS measurement and analysis (GENEPIONEER Biotech Co., Ltd).

### 5.20 Molecular Docking [82]

Automated docking was performed using the 3D structure of ATCase (PDB ID: 6PNZ) in Discovery Studio 4.0. Water molecules and other small ligands were removed from the ATCase structure, and hydrogen atoms were added. The receptor active site was defined to identify key amino acids within the predicted binding pocket. The conformation with the highest ‐CDOCKER_INTERACTION_ENERGY score was selected as the best binding mode, and two‐ and three‐dimensional ligand‐protein interaction diagrams were generated.

### 5.21 Transcriptomics [83]

Py‐27 (final concentration of 1/2 × MIC) was added to the bacterial suspension and incubated at 37°C with shaking at 200 rpm for 15 h. The bacterial cultures were then washed three times with 0.01 mol/L PBS (pH 7.4) and centrifuged at 12 000 g for 10 min at 4°C to collect the bacterial precipitates. The samples were immediately transferred to liquid nitrogen for rapid freezing and then stored at −80°C. Total RNA was extracted from the samples, and the concentration and purity of the extracted RNA were determined. The integrity of the RNA was assessed by agarose gel electrophoresis, RNA integrity was assessed using the Bioanalyzer 2100 system (Agilent Technologies, CA, USA). Genes with adjusted *p* < 0.05, identified by DESeq, were considered differentially expressed. The GO functional enrichment analysis and KEGG pathway enrichment analysis of the differentially expressed gene sets were conducted using the clusterProfiler software.

### 5.22 Total RNA Extraction and Quantitative Reverse Transcription (qRT)‐PCR

The overnight cultures of MRSA (ATCC 43300) were diluted to an OD_600_ of 0.05 in fresh TSB and cultivated to mid‐exponential phase (OD_600_ = 1). The cells were collected by centrifugation at 12 000 g for 2 min and then immediately treated with 100 µL of lysozyme (0.5 mg/mL) in a 37°C water bath for 1 h. Total RNA was extracted from MRSA using the *SteadyPure* Universal RNA Extraction Kit (AG21017, Accurate Biotechnology) according to the manufacturer's instructions. The complementary DNA synthesis were achieved by using a SPARKscript II All‐in‐one RT SuperMix for qPCR (AG0305, SparkJade). qRT‐PCR was performed with 2ΧSYBR Green qPCR Mix (AH0104, SparkJade) using the StepOne real‐time PCR system (Applied Biosystems). AII qRT‐PCR assays were repeated at least three times. Table S7 list all primers used in the quantitative PCR.

### 5.23 Constructions of *pyrB* Knockout Bacteria

The *pyrB* knockout strain of MRSA (ATCC 43300) was constructed using plasmid pBT2. Briefly, the upstream and downstream fragments of *pyrB* were amplifed from the genomic DNA of MRSA. The PCR products were then cloned into the pBT2 by T4 DNA Ligase (Takara), the constructed plasmid was named pBT2‐*pyrB*. Plasmid pBT2‐*pyrB* of the transformed RN4220, was used to introduced into MRSA by electroporation. Taken the advantage of the characteristics of pBT2 vector that was sensitive to temperature, and then repeatedly passaged at 42°C, the *pyrB* deletion mutant MRSA Δ*pyrB* was screened. Finally, the genome editing was confirmed by PCR amplification and further confirmed through DNA sequencing.

### 5.24 Liquid Chromatography‑Mass Spectrometry (LC‑MS) Analysis [83]

The MRSA (ATCC 43300) bacterial suspension was cultured to the logarithmic growth phase (OD_600_ = 0.5) and then treated with 1/4 × MIC or 1/2 × MIC of Py‐27 and CTP for 12 h. An appropriate volume of the culture was centrifuged at 4°C (10 000 g for 10 min). The supernatant was discarded, and the bacterial pellet was collected. The pellet was resuspended in pre‐cooled PBS and centrifuged again (10 000 g for 10 min), with this washing step repeated 2–3 times. The bacterial cells were then treated with lysozyme and incubated at 37°C for 30 min. Acetonitrile was added, followed by vortex mixing and incubation on ice for 10 min. The mixture was centrifuged (10 000 g for 10 min), and the supernatant was collected. A 10‐µL aliquot was applied to the ZORBAX Eclipse Plus C18 600 Bar (2.1 × 100 mm, 1.8 µm) and eluted under acetonitrile and formic acid (0.1%) at a flow rate of 0.2 mL/min. Then, AB SCIEX QTRAP 5500 mass spectrometer was used to analyze the chromatographic peak area and retention time of N‐carbamoyl‐L‐aspartate in negative ion mode.

### 5.25 Enzyme‐Linked Immunosorbent Assay (ELISA) of ATCase

Centrifuge to collect the bacterial cells treated with Py‐27 (4°C, 5000 g, 10 min), resuspend the cells in PBS, add 0.5 mg/mL lysozyme and incubate in a 37°C water bath for 30 min, then centrifuge (12 000 g, 20 min, 4°C) to collect the supernatant. After determining the protein concentration by the BCA method, the activity was measured according to the microorganism aspartate carbamoyltransferase ELISA detection kit (Yuanju BioTech Center, Shanghai).

### 5.26 Protein Expression and Purification

Plasmid pET‐22b(+)‐*pyrB* were constructed after codon optimization in *E. coli*. The plasmid was then transformed into BL21 (DE3)‐competent cells. After overnight incubation, single colonies were picked and transferred to LB medium containing ampicillin resistance (30 µg/mL). The cultures were shaken at 37°C until the OD_600_ reached approximately 0.6‐0.8, before induction of fusion protein expression by 0.5 mmol/L isopropyl thio‐β‐D‐galactoside (IPTG) at 15°C for 16 h. Then, the cells were harvested by centrifugation. After centrifugation, the bacterial sediment was suspended in lysis buffer (PBS, 300 mm NaCl, 10% Glycerol, pH 7.4) and disrupted by ultrasonication. The supernatant was passed through the Ni NTA Beads 6FF column at a flow rate of 1.0 mL/min by a peristaltic pump at 4°C overnight, then passed onto the binding buffer (PBS‐NaCl, pH 7.4 with 50 mm imidazole) in 20 mL. The bacterial proteins were then removed with elution buffer, which contained 500 mm imidazole in 10 mL to obtain the target protein. The fractions of pure product, total protein, supernatant, and precipitate were then analysed by SDS‐PAG. The protein concentration was determined by BCA kit.

### 5.27 BioLacyer Interferometry (BLI)

BLI experiments were performed using an Octet system (Pall, USA) placed in PBS pH 7.4, 0.05% (v/v) Tween‐20 and 0.1% BSA running buffer at 25°C. Freshly prepared ATCase protein was coupled to the tip of a Forte Bio Octet NTA instrument. A dilution series of Py‐27 (0.3125 to 10 µm) was used to measure the dose‐response curve of association and dissociation. The dissociation period was set at 60 s.

### 5.28 Cellular Thermal Shift Assay (CETSA)

The ATCase was treated with Py‐27 or DMSO and incubated at 4°C for 30 min. After the mixture was centrifuged at 18 000 g for 20 min, the supernatant was divided into 6 tubes, incubated at different temperatures (50°C, 52°C, 54°C, 56°C, 58°C, 60°C, 62°C, and 64°C) and immediately cooled on ice for 3 min for 5 min each. The samples were centrifuged at 18 000 g for 20 min to obtain the supernatant. All the samples were analyzed by SDS‐PAGE to detect the Py‐27 protein content.

### 5.29 Molecular Dynamics Simulation

Molecular dynamics simulations of the complexes obtained by molecular docking were performed using Gromacs 2020 software. The CHARMM36 force field was used for the protein, GAFF2 for the ligand, and the TIP3P water model was employed to solvate the protein–ligand system. Sodium and chloride ions were added to neutralize the system charge. The steepest descent algorithm was used to minimize its energy, after which the system was run in constrained NVT and NPT for 2000 ps to bring the system to equilibrium. The NPT was combined on top of the NVT equilibrium to stabilize the system. Subsequently, MD simulations were run at 300 K for 100 ns, with trajectories saved every 100 ps. To analyze the simulation results, the trajectory‐based root mean square deviation (RMSD), root mean square fluctuation (RMSF), protein radius of gyration (Rg), and number of hydrogen bonds were calculated.

### 5.30 DAPI Fluorescence Staining Analysis

1 × 10^8^ CFU/mL bacterial were treated with Py‐27 at 1/2 × MIC for 3 h at 37°C with shaking (180 rpm) in the dark. After incubation, cells were collected by centrifugation (5000 g, 5 min), washed three times with PBS, and then stained with 10 µm DAPI for 30 min at 37°C in the dark. DAPI staining fluorescence photographs was visualized using CLSM (ZEISS, LSM800).

### 5.31 Intracellular ROS Levels Measurement [84, 85]

1 × 10^8^  CFU/mL bacterial suspensions were treated with Py‐27 at 1/2×, 2×, and 4× MIC, or with H_2_O_2_ (10 mm) as a positive control for 4 h at 37°C with shaking (180 rpm) in the dark. After incubation, cells were collected by centrifugation (5000 g, 5 min), washed three times with PBS, and then stained with 10 µm DCFH‐DA for 30 min at 37°C in the dark. DCFH‐DA staining fluorescence photographs was visualized using CLSM (ZEISS, LSM800).

The logarithmically growing bacterial suspension (1 × 10^8^  CFU/mL) were treated with NAC (16 mm), Py‐27 (4 × MIC), or their combination at 37°C for 4 h. Untreated bacteria served as the control. After treatment, DCFH‐DA was added to a final concentration of 10 µm, and the mixtures were incubated for 30 min at 37°C in the dark. The cells were subsequently washed and resuspended in PBS, and fluorescence intensity was measured at 488 nm/522 nm (excitation/emission).

### 5.32 Membrane Fluidity Assay [84]

Bacterial membrane fluidity was assessed using the fluorescent probe Laurdan. Overnight suspensions were harvested, washed, and resuspended in 0.01 mol/L PBS (pH 7.4) to an OD_600_ of 0.5. The suspensions were stained with 10 µm Laurdan for 10 min, washed twice, and then treated with Py‑27 (1×, 2×, or 4× MIC) for 35 min. Cells were collected by centrifugation (5000 g, 5 min), rinsed with PBS, and the Laurdan fluorescence intensity was measured at emission wavelengths of 435 and 490 nm (excitation 350 nm) using the EnSpire 2300 Multimode Plate Reader. the final results were calculated by quantifying the generalized polarization (GP) of Laurdan using the Formula (2).

(2) $$\text{GP}=\frac{\text{I435}-\text{I490}}{\text{I435}+\text{I490}}$$

### 5.33 ATP Determination

Extracellula ATP levels were determined using the Enhanced ATP Assay Kit (Beyotime, Cat. No. S0027). Overnight cultures of the bacterial fluid were centrifuged and resuspended in 0.01 mol/L PBS (pH 7.4), and the bacterial suspensions were adjusted to an OD_600_ of approximately 0.5. After treatment with Py‑27 (1×, 2×, or 4× MIC) for 2 h, the bacterial cultures were centrifuged at 12 000 g for 5 min at 4°C, and the supernatants were collected. The assay solution was added to a 96‐well plate and incubated for another 5 min at room temperature. The supernatants were added to the well and mixed quickly before recording in the model of luminescence using the EnSpire 2300 Multimode Plate Reader.

### 5.34 Scanning Electron Microscope (SEM)

Bacterial suspensions of MRSA (ATCC 43300) (1 × 10^8^ CFU/mL) were treated with Py‑27 at 2×, 4×, and 8× MIC for 12 h at 37°C. And then washed with PBS, fixed with 3% glutaraldehyde (24 h, 4°C), and dehydrated through a graded ethanol series. Following substitution with isoamyl acetate, samples were critical‑point dried, sputter‑coated with gold, and imaged by scanning electron microscopy (JSM‐IT700HR, JEOL, Japan).

### 5.35 Transmission Electron Microscopy (TEM)

MRSA (ATCC 43300) suspensions (1 × 10^8^ CFU/mL) were treated with Py‑27 (2×, 4×, or 8× MIC) for 12 h at 37°C. Cells were harvested by centrifugation (5000 g, 10 min, 4°C), washed with PBS (pH 7.2), and fixed overnight at 4°C in 3% glutaraldehyde. After PBS washing, samples were post‑fixed with 1% osmium tetroxide for 3 h at room temperature, dehydrated through a graded acetone series, and embedded in Epon‑812. Ultrathin sections (60‑‐90 nm) were stained with uranyl acetate and lead citrate, then examined by transmission electron microscopy (JEM‐1400FLASH, JEOL, Japan).

### 5.36 Protein Leakage Assay

The intracellular soluble protein content of MRSA (ATCC 43300) was measured using a BCA protein assay kit. After treatment with Py‐27 (1×, 2×, or 4×MIC) for 2 h, the bacterial cultures were centrifuged at 5000 g for 10 min at 4°C. The supernatant was collected and analyzed for protein concentration according to the kit protocol.

### 5.37 Bacterial Membrane Permeability

Extracellular β‑galactosidase activity was measured after treated MRSA (ATCC 43300) with Py‐27 (1×, 2×, or 4× MIC) for 12 h at 37°C. Supernatants were collected, mixed with 3 mm ONPG (2‑nitrophenyl‑β‑D‑galactopyranoside), and incubated for 2 h at 37°C. The absorbance at 420 nm was recorded using a Multiskan Go microplate reader (Thermo Scientific).

### 5.38 Membrane Integrity Assay

The overnight culture of bacterial suspension was centrifuged and resuspended in PBS, and the bacterial suspensions were adjusted to an OD_600_ of approximately 0.5 after three times washed with PBS buffer. Bacterial suspensions were treated with Py‐27 (1×, 2×, or 4× MIC) at 37°C for 2 h without light. Subsequently, a final concentration of 10 nm propidium iodide (PI) dye was added for 30 min. After centrifugation at 5000 g for 5 min, the bacterial cells were collected and rinsed with sterile PBS. And the fluorescence values were measured at Ex/Em 535/615 nm.

### 5.39 Membrane Hyperpolarization Study [84]

Bacterial membrane depolarization was assessed using the fluorescent probe DiSC_3_(5). A suspension containing 10 µm DiSC_3_(5) was incubated in a black 96‑well plate at 37°C for 30 min in the dark. Baseline fluorescence (Ex/Em = 622/670 nm) was measured every 2 min until stable (6 min). Subsequently, Py‑27 (1×, 2×, or 4× MIC) was added, and fluorescence intensity was recorded every 2 min for an additional 14 min. A dye‑only control (without bacteria) was included as background.

### 5.40 SOD Activity and MDA Levels Assays [86]

The logarithmically growing bacterial suspension (1 × 10^8^ CFU/mL) was mixed with NAC (16 mm), Py‐27(4 × MIC), or their combination at 37°C for 4 h. The bacterial cells were then collected by centrifugation and washed with 1 × PBS. SOD Assay Kit and MDA Assay Kit purchased from Shanghai Beyotime Biotechnology Co., Ltd., were respectively used to detect the activity following the manufacturer's protocols.

### 5.41 Statistical Analysis

All experiments were performed with biological replicates and data were shown as mean ± SD. Statistical analyses were performed using one‐ or two‐way ANOVA with GraphPad Prism 10.1.2 software. The statistical significance was indicated by ^*^ *p* < 0.05, ^**^ *p* < 0.01, ^***^ *p* < 0.001; and ns, not significant, respectively.

## Author Contributions

X. Yang, X. Cao, X. Shang: Writing – original draft, Review, Editing, Preparation of figure. X. Miao, F. Yang, J. Wang, X. Liu, L. Dai, Y. Ma, T. Bu, Z. Gong: Preparation of figure, Visualization, Methods, Investigation. C. Bai: Validation, Visualization. X. Yang, X. Shang: Conceptualization, Supervision, Project administration, Funding acquisition.

## Conflicts of Interest

The authors declare no conflicts of interest.

## Supporting information




**Supporting File**: advs76704‐sup‐0001‐SuppMat.docx.

## Data Availability

The data that support the findings of this study are available from the corresponding author upon reasonable request.
