# Supplementary material for: Pyrazole‐Derived Antibacterial Compounds Effectively Treat Methicillin‐Resistant Staphylococcus Aureus Infections by Inhibiting Aspartate Transcarbamoylase
Source: Adv Sci (Weinh). 2026 Jul 20:e76704. Online ahead of print. doi: 10.1002/advs.76704 (PMC13383702; doi:10.1002/advs.76704)
Supplement: Supplementary file 1 — Supporting File: advs76704‐sup‐0001‐SuppMat.docx. [file ADVS-9999-e76704-s001.docx]

Supplementary Materials for

**Pyrazole-Derived antibacterial compounds effectively treat Methicillin-Resistant *Staphylococcus aureus* infections by inhibiting aspartate transcarbamoylase**

Xiaorong Yang†^1^, Xinyuan Cao†^2,3^, Lixia Dai†^1,4^, Feng Yang^1^, Jing Wang^1^, Yudong Ma^1^, Tong Bu^1,5^, Zile Gong^1,5^, Xueyan Liu^1^, Changcai Bai^3^, Xiaolou Miao^1,5^, Xiaofei Shang*^1,4,5^.

^1^ Key Laboratory of New Animal Drug Project, Gansu Province, Key Laboratory of Veterinary Pharmaceutical Development of Ministry of Agriculture, Lanzhou Institute of Husbandry and Pharmaceutical Sciences, Chinese Academy of Agricultural Sciences, Lanzhou, P.R. China.

^2^ People's Hospital of Ningxia Hui Autonomous Region, Ningxia Medical University, Yinchuan, P.R. China.

^3^ College of Pharmacy, Ningxia Medical University, Yinchuan, P.R. China.

^4^ College of Veterinary Medicine, Gansu Agricultural University, Lanzhou, P.R. China.

^5^ School of Pharmacy, Gansu University of Chinese Medicine, Lanzhou, P.R. China.

*Corresponding author. Email: shangxiaofei@caas.cn.

**This file includes:**

Supplementary Text

Figs. S1 to S13

Tables S1 to S8

Data S1: NMR spectra

Supplementary Text

**Text 1. Chemical Synthesis of Pyrazole-Amide Derivatives**

**General Procedure A:** In a dry round-bottom flask, a 5-aminopyrazole derivative (5.00 mmol) was dissolved in dichloromethane (25 mL). After stirring at room temperature, the corresponding aryl acyl chloride (10.00 mmol) was added, followed by triethylamine (15.00 mmol). After the addition was complete, the reaction mixture was stirred for 4-12 hours. The reaction progress was monitored by TLC. Upon complete consumption of the starting material, the reaction was quenched with water. The resulting organic phase was dried over anhydrous Na_2_SO_4_, filtered, and concentrated under reduced pressure. The crude product was purified by silica gel column chromatography (PE/EtOAc 4:1) to afford the pyrazole-amide derivative.

**General Procedure B:** In a dry round-bottom flask, a 5-aminopyrazole derivative (5.00 mmol) was dissolved in pyridine (25 mL). After stirring at 0°C for 10 minutes, the corresponding carboxylic acid (7.50 mmol) was added, followed by the slow dropwise addition of POCl₃ (10.00 mmol). After the addition was complete, the reaction mixture was stirred at room temperature for 6-12 hours. The reaction progress was monitored by TLC. Upon complete consumption of the starting material, the reaction was quenched with 2N hydrochloric acid. The mixture was extracted with ethyl acetate (3 × 10 mL). The combined organic phase was dried over anhydrous Na_2_SO_4_, filtered, and concentrated under reduced pressure. The crude product was purified by silica gel column chromatography (PE/EtOAc 4:1) to afford the pyrazole-amide derivative.

- 1. *N*-(1-phenyl-1*H*-pyrazol-5-yl)benzamide **(Py-1)**

Synthesize according to general procedure A. Yellow solid, 78% yield, mp 113±1 ℃; ^1^H NMR (500 MHz, Chloroform-*d*) δ 8.17 (brs, 1H), 7.75 - 7.68 (m, 2H), 7.63 (d, *J* = 2.0 Hz, 1H), 7.57 - 7.47 (m, 5H), 7.47 - 7.34 (m, 3H), 6.79 (d, *J* = 2.0 Hz, 1H). ^13^C NMR (126 MHz, Chloroform-*d*) δ 163.93, 140.52, 137.90, 135.62, 133.13, 132.41, 129.88, 128.89, 128.55, 127.05, 124.59, 98.98. HRMS (ESI): calcd for C_16_H_13_N_3_OH (M+H^+^) 264.11314; found 264.11328.

- 1. *N*-(1-phenyl-1*H*-pyrazol-5-yl)-4-(trifluoromethyl)benzamide **(Py-2)**

Synthesize according to general procedure A. White solid, 67% yield, mp 181±1 ℃; ^1^H NMR (400 MHz, Chloroform-*d*) δ 7.72 (d, *J* = 2.0 Hz, 1H), 7.61 - 7.56 (m, 6H), 7.39 - 7.37 (m, 2H), 7.26 (d, *J* = 4.5 Hz, 2H), 6.44 (d, *J* = 2.1 Hz, 1H). ^13^C NMR (101 MHz, Chloroform-*d*) δ 170.70, 140.79, 137.50, 136.23, 136.07, 134.37 (q, *J* = 33.33 Hz), 129.48, 129.11, 128.95, 125.56 (q, *J* = 4.04 Hz), 124.57, 123.21 (q, *J* = 274.72 Hz), 105.79. HRMS (ESI): calcd for C_17_H_12_F_3_N_3_OH (M+H^+^) 332.10052; found 332.10043.

- 1. 3,5-dichloro-*N*-(1-phenyl-1*H*-pyrazol-5-yl)benzamide **(Py-3)**

Synthesize according to general procedure A. White solid, 61% yield, mp 169±1 ℃; ^1^H NMR (400 MHz, Chloroform-*d*) δ 7.73 (d, *J* = 1.9 Hz, 1H), 7.46 (s, 1H), 7.43 - 7.41 (m, 2H), 7.27 - 7.19 (m, 6H), 6.42 (d, *J* = 1.9 Hz, 1H). ^13^C NMR (101 MHz, Chloroform-*d*) δ 169.35, 140.83, 137.28, 135.67, 135.57, 135.47, 132.78, 129.55, 129.15, 126.90, 124.73, 105.87. HRMS (ESI): calcd for C_16_H_11_Cl_2_N_3_OH (M+H^+^) 332.03519; found 332.03516.

- 1. 2,4-dichloro-*N*-(1-phenyl-1*H*-pyrazol-5-yl)benzamide **(Py-4)**

Synthesize according to general procedure A. White solid, 61% yield, mp 169±1 ℃; ^1^H NMR (400 MHz, Chloroform-*d*) δ 7.73 (d, *J* = 1.9 Hz, 1H), 7.46 (s, 1H), 7.43 - 7.41 (m, 2H), 7.27 - 7.19 (m, 6H), 6.42 (d, *J* = 1.9 Hz, 1H). ^13^C NMR (101 MHz, Chloroform-*d*) δ 169.35, 140.83, 137.28, 135.67, 135.57, 135.47, 132.78, 129.55, 129.15, 126.90, 124.73, 105.87. HRMS (ESI): calcd for C_16_H_11_Cl_2_N_3_OH (M+H^+^) 332.03519; found 332.03516.

- 1. 4-methyl-*N*-(1-phenyl-1*H*-pyrazol-5-yl)benzamide **(Py-5)**

Synthesize according to general procedure A. White solid, 71% yield, mp 163±1 ℃; ^1^H NMR (400 MHz, Chloroform-*d*) δ 8.15 (brs, 1H), 7.64 - 7.58 (m, 3H), 7.55 - 7.48 (m, 4H), 7.47 - 7.41 (m, 1H), 7.23 (d, *J* = 7.7 Hz, 2H), 6.79 (d, *J* = 2.0 Hz, 1H), 2.39 (s, 3H). ^13^C NMR (101 MHz, Chloroform-*d*) δ 163.90, 143.07, 140.47, 137.94, 135.73, 130.27, 129.81, 129.51, 128.45, 127.07, 124.54, 98.94, 21.45. HRMS (ESI): calcd for C_17_H_15_N_3_OH (M+H^+^) 278.12879; found 278.12872.

- 1. 3-bromo-*N*-(1-phenyl-1*H*-pyrazol-5-yl)benzamide **(Py-6)**

Synthesize according to general procedure A. White solid, 79% yield, mp 112±1 ℃; ^1^H NMR (400 MHz, Chloroform-*d*) δ 7.71 (d, *J* = 2.0 Hz, 1H), 7.59 - 7.52 (m, 3H), 7.42 - 7.36 (m, 4H), 7.31 - 7.28 (m, 1H), 7.20 - 7.12 (m, 2H), 6.41 (d, *J* = 2.0 Hz, 1H). ^13^C NMR (101 MHz, Chloroform-*d*) δ 170.51, 140.62, 137.51, 136.42, 135.79, 135.04, 131.80, 129.91, 129.39, 128.91, 127.06, 124.82, 122.59, 105.58. HRMS (ESI): calcd for C_16_H_12_BrN_3_OH (M+H^+^) 342.02365; found 342.02364.

- 1. 4-iodo-*N*-(1-phenyl-1*H*-pyrazol-5-yl)benzamide **(Py-7)**

Synthesize according to general procedure A. White solid, 68% yield, mp 170±1 ℃; ^1^H NMR (500 MHz, DMSO-*d*_6_) δ 10.44 (brs, 1H), 7.90 (d, *J* = 8.5 Hz, 2H), 7.72 (d, *J* = 1.9 Hz, 1H), 7.64 (d, *J* = 8.0 Hz, 2H), 7.56 - 7.52 (m, 2H), 7.48 - 7.44 (m, 2H), 7.37 - 7.33 (m, 1H), 6.48 (d, *J* = 1.9 Hz, 1H). ^13^C NMR (126 MHz, DMSO-*d*_6_) δ 165.43, 139.75, 138.83, 137.57, 137.46, 135.69, 132.54, 131.06, 129.53, 129.11, 127.37, 123.27, 104.16, 100.13. HRMS (ESI): calcd for C_16_H_12_IN_3_OH (M+H^+^) 390.00978; found 390.00974.

- 1. *N*-(1-phenyl-1*H*-pyrazol-5-yl)-2-(trifluoromethyl)benzamide **(Py-8)**

Synthesize according to general procedure A. White solid, 65% yield, mp 138±1 ℃; ^1^H NMR (500 MHz, Chloroform-*d*) δ 7.89 (brs, 1H), 7.72 - 7.68 (m, 1H), 7.60 - 7.53 (m, 3H), 7.50 - 7.44 (m, 3H), 7.43 - 7.34 (m, 3H), 6.74 (d, *J* = 2.0 Hz, 1H). ^13^C NMR (126 MHz, Chloroform-*d*) δ 164.44, 140.25, 137.53, 135.02, 134.31, 134.29, 132.15, 130.58, 129.65, 128.67, 128.47, 127.28 (q, *J* = 32.76 Hz), 126.60 (q, *J* = 4.80 Hz), 124.91, 123.33 (q, *J* = 274.68 Hz), 99.54. HRMS (ESI): calcd for C_17_H_12_F_3_N_3_OH (M+H^+^) 332.10052; found 332.10046.

- 1. *N*-(1-phenyl-1*H*-pyrazol-5-yl)-3-(trifluoromethyl)benzamide **(Py-9)**

Synthesize according to general procedure A. White solid, 71% yield, mp 121±1 ℃; ^1^H NMR (400 MHz, DMSO-*d*_6_) δ 10.67 (brs, 1H), 8.30 - 8.08 (m, 2H), 7.98 (d, *J* = 7.8 Hz, 1H), 7.84 - 7.69 (m, 2H), 7.57 (d, *J* = 7.8 Hz, 2H), 7.48 (t, *J* = 7.7 Hz, 2H), 7.36 (t, *J* = 7.4 Hz, 1H), 6.52 (d, *J* = 1.9 Hz, 1H). ^13^C NMR (101 MHz, DMSO-*d*_6_) δ 164.65, 139.81, 138.82, 135.52, 134.08, 131.78, 129.95, 129.33 (q, *J* = 32.32 Hz), 129.13, 128.70 (q, *J* = 4.04 Hz), 127.45, 124.29 (q, *J* = 4.04 Hz),123.83 (q, *J* = 273.71 Hz), 123.35, 104.07. HRMS (ESI): calcd for C_17_H_12_F_3_N_3_OH (M+H^+^) 332.10052; found 332.10043.

- 1. *N*-(3-(*tert*-butyl)-1-phenyl-1*H*-pyrazol-5-yl)-4-(trifluoromethyl)benzamide **(Py-10)**

Synthesize according to general procedure A. White solid, 61% yield, mp 193±1 ℃; ^1^H NMR (500 MHz, DMSO-*d*_6_) δ 10.57 (brs, 1H), 7.93 - 7.79 (m, 3H), 7.55 - 7.49 (m, 2H), 7.48 - 7.43 (m, 2H), 7.35 - 7.31 (m, 1H), 6.41 (s, 1H), 1.31 (s, 9H). ^13^C NMR (126 MHz, DMSO-*d*_6_) δ 163.20, 160.90, 138.97, 136.43, 135.13, 134.47, 131.51, 129.09, 127.00, 126.48, 123.09, 100.50, 32.14, 30.15. HRMS (ESI): calcd for C_20_H_19_Cl_2_N_3_OH (M+H^+^) 388.09779; found 388.09778.

- 1. *N*-(4-bromo-3-methyl-1-phenyl-1*H*-pyrazol-5-yl)-3,5-dichlorobenzamide **(Py-11)**

Synthesize according to general procedure A. White solid, 56% yield, mp 224±1 ℃; ^1^H NMR (500 MHz, DMSO-*d*_6_) δ 10.74 (brs, 1H), 7.93 - 7.89 (m, 3H), 7.50 - 7.46 (m, 4H), 7.39 - 7.35 (m, 1H), 2.27 (s, 3H). ^13^C NMR (126 MHz, DMSO-*d*_6_) δ 163.47, 146.89, 138.32, 135.50, 134.75, 133.93, 132.02, 129.35, 127.86, 126.50, 122.92, 94.58, 12.58. HRMS (ESI): calcd for C_17_H_12_BrCl_2_N_3_OH (M+H^+^) 423.96136; found 423.96130.

- 1. 3,5-dichloro-*N*-(1-methyl-1*H*-pyrazol-5-yl)benzamide **(Py-12)**

Synthesize according to general procedure A. White solid, 91% yield, mp 167±1 ℃; ^1^H NMR (500 MHz, DMSO-*d*_6_) δ 10.53 (brs, 1H), 7.99 (d, *J* = 1.9 Hz, 2H), 7.90 (t, *J* = 1.9 Hz, 1H), 7.40 (d, *J* = 2.0 Hz, 1H), 6.25 (d, *J* = 1.9 Hz, 1H), 3.71 (s, 3H). ^13^C NMR (126 MHz, DMSO-*d*_6_) δ 162.98, 137.47, 136.64, 135.63, 134.41, 131.42, 126.74, 100.43, 35.77. HRMS (ESI): calcd for C_11_H_9_Cl_2_N_3_OH (M+H^+^) 270.01954; found 270.01951.

- 1. *N*-(1-methyl-1*H*-pyrazol-5-yl)-4-(trifluoromethyl)benzamide **(Py-13)**

Synthesize according to general procedure A. White solid, 74% yield, mp 155±1 ℃; ^1^H NMR (400 MHz, DMSO-*d*_6_) δ 10.59 (brs, 1H), 8.18 (d, *J* = 8.1 Hz, 2H), 7.93 (d, *J* = 8.0 Hz, 2H), 7.41 (d, *J* = 1.8 Hz, 1H), 6.28 (s, 1H), 3.72 (s, 3H). ^13^C NMR (101 MHz, DMSO-*d*_6_) δ 164.52, 137.44, 137.21, 135.86, 131.83 (q, *J* = 32.12 Hz), 128.83, 125.49 (q, *J* = 3.94 Hz), 123.85 (q, *J* = 273.71 Hz), 100.46, 35.70. HRMS (ESI): calcd for C_12_H_10_F_3_N_3_OH (M+H^+^) 270.08487; found 270.08484.

- 1. 3,5-dichloro-*N*-(1-methyl-3-phenyl-1*H*-pyrazol-5-yl)benzamide **(Py-14)**

Synthesize according to general procedure A. White solid, 44% yield, mp 214±1 ℃; ^1^H NMR (500 MHz, DMSO-*d*_6_) δ 10.63 (brs, 1H), 8.02 (d, *J* = 1.9 Hz, 2H), 7.94 - 7.91 (m, 1H), 7.81 - 7.76 (m, 2H), 7.40 (t, *J* = 7.6 Hz, 2H), 7.36 - 7.24 (m, 1H), 6.73 (s, 1H), 3.77 (s, 3H). ^13^C NMR (126 MHz, DMSO-*d*_6_) δ 163.07, 148.26, 136.95, 136.57, 134.44, 133.28, 131.49, 128.64, 127.49, 126.78, 124.78, 97.71, 36.00. HRMS (ESI): calcd for C_17_H_13_Cl_2_N_3_OH (M+H^+^) 346.05084; found 346.05081.

- 1. *N*-(3-(*tert*-butyl)-1-methyl-1*H*-pyrazol-5-yl)-3,5-dichlorobenzamide **(Py-15)**

Synthesize according to general procedure A. Yellow liquid, 84% yield; ^1^H NMR (500 MHz, Chloroform-*d*) δ 8.17 (brs, 1H), 7.69 (s, 2H), 7.52 (t, *J* = 1.8 Hz, 1H), 6.09 (s, 1H), 3.65 (s, 3H), 1.25 (s, 9H). ^13^C NMR (126 MHz, Chloroform-*d*) δ 163.88, 160.77, 135.82, 135.62, 134.33, 132.11, 126.04, 97.34, 35.36, 32.14, 30.30. HRMS (ESI): calcd for C_15_H_17_Cl_2_N_3_OH (M+H^+^) 326.08214; found 326.08191.

- 1. *N*-(1-(*tert*-butyl)-4-cyano-1*H*-pyrazol-5-yl)-3,5-dichlorobenzamide **(Py-16)**

Synthesize according to general procedure B. Yellow liquid, 30% yield; ^1^H NMR (500 MHz, Chloroform-*d*) δ 8.32 (d, *J* = 8.3 Hz, 2H), 7.80 (d, *J* = 8.4 Hz, 2H), 7.72 (s, 1H), 1.62 (s, 9H). ^13^C NMR (126 MHz, Chloroform-*d*) δ 169.97, 149.87, 147.40, 139.49, 136.98, 135.06 (q, *J* = 32.76 Hz), 130.25, 125.92 (q, *J* = 3.78 Hz), 123.35 (q, *J* = 273.42 Hz), 113.15, 84.15, 61.84, 29.07. HRMS (ESI): calcd for C_16_H_15_F_3_N_4_OH (M+H^+^) 337.12707; found 337.12711.

- 1. *N*-(4-cyano-1-phenyl-1*H*-pyrazol-5-yl)-4-(trifluoromethyl)benzamide **(Py-17)**

Synthesize according to general procedure A. Yellow solid, 64% yield, mp 188±1 ℃; ^1^H NMR (500 MHz, DMSO-*d*_6_) δ 11.30 (brs, 1H), 8.40 (s, 1H), 8.09 (d, *J* = 8.1 Hz, 2H), 7.94 (d, *J* = 8.3 Hz, 2H), 7.61 - 7.50 (m, 4H), 7.50 - 7.43 (m, 1H). ^13^C NMR (126 MHz, DMSO-*d*_6_) δ 164.89, 142.56, 140.73, 137.37, 135.72, 132.47 (q, *J* = 31.50 Hz), 129.55, 128.99, 128.85, 125.86 (q, *J* = 3.78 Hz), 123.79, 123.72 (q, *J* = 273.42 Hz), 112.58, 90.64. HRMS (ESI): calcd for C_18_H_11_F_3_N_4_OH (M+H^+^) 357.09577; found 357.09576.

- 1. 3,5-dichloro-*N*-(4-cyano-1-phenyl-1*H*-pyrazol-5-yl)benzamide **(Py-18)**

Synthesize according to general procedure A. Yellow solid, 77% yield, mp 197±1 ℃; ^1^H NMR (500 MHz, DMSO-*d*_6_) δ 11.39 (brs, 1H), 8.36 (s, 1H), 7.93 - 7.89 (m, 3H), 7.60 - 7.52 (m, 4H), 7.47 - 7.44 (m, 1H). ^13^C NMR (126 MHz, DMSO-*d*_6_) δ 163.32, 142.58, 137.53, 134.62, 134.47, 132.03, 129.51, 128.82, 127.78, 126.72, 123.73, 112.76, 89.79. HRMS (ESI): calcd for C_17_H_10_Cl_2_N_4_OH (M+H^+^) 357.03044; found 357.03040.

- 1. *N*-(4-cyano-1-(*p*-tolyl)-1*H*-pyrazol-5-yl)-4-(trifluoromethyl)benzamide **(Py-19)**

Synthesize according to general procedure A. Pale yellow solid, 65% yield, mp 209±1 ℃; ^1^H NMR (400 MHz, DMSO-*d*_6_) δ 11.23 (brs, 1H), 8.36 (s, 1H), 8.09 (d, *J* = 8.0 Hz, 2H), 7.94 (d, *J* = 8.1 Hz, 2H), 7.45 (d, *J* = 8.1 Hz, 2H), 7.32 (d, *J* = 8.0 Hz, 2H), 2.32 (s, 3H). ^13^C NMR (101 MHz, DMSO-*d*_6_) δ 164.88, 142.34, 140.86, 138.58, 135.88, 135.01, 132.37 (q, *J* = 32.32 Hz), 129.86, 128.81, 125.80 (q, *J* = 3.84 Hz), 123.71 (q, *J* = 273.71 Hz), 123.59, 112.67, 90.29, 20.57. HRMS (ESI): calcd for C_19_H_13_F_3_N_4_OH (M+H^+^) 371.11142; found 371.11139.

- 1. 3,5-dichloro-*N*-(4-cyano-1-(*p*-tolyl)-1*H*-pyrazol-5-yl)benzamide **(Py-20)**

Synthesize according to general procedure A. Pale yellow solid, 59% yield, mp 178±1 ℃; ^1^H NMR (500 MHz, Chloroform-*d*) δ 8.68 (brs, 1H), 7.86 (s, 1H), 7.60 (d, *J* = 1.9 Hz, 2H), 7.51 (t, *J* = 1.9 Hz, 1H), 7.30 (d, *J* = 8.3 Hz, 2H), 7.24 (d, *J* = 8.2 Hz, 2H), 2.37 (s, 3H). ^13^C NMR (126 MHz, Chloroform-*d*) δ 162.93, 142.00, 139.88, 139.24, 135.75, 134.61, 134.50, 132.74, 130.28, 126.29, 123.92, 112.56, 89.50, 21.16. HRMS (ESI): calcd for C_18_H_12_Cl_2_N_4_OH (M+H^+^) 371.04609; found 371.04608.

- 1. *N*-(1-(4-chlorophenyl)-4-cyano-1*H*-pyrazol-5-yl)-4-(trifluoromethyl)benzamide **(Py-21)**

Synthesize according to general procedure B. Yellow solid, 58% yield, mp 205±1 ℃; ^1^H NMR (500 MHz, DMSO-*d*_6_) δ 11.32 (brs, 1H), 8.40 (s, 1H), 8.10 (d, *J* = 8.1 Hz, 2H), 7.95 (d, *J* = 8.3 Hz, 2H), 7.63 - 7.60 (m, 4H). ^13^C NMR (126 MHz, DMSO-*d*_6_) δ 164.80, 142.83, 140.96, 136.27, 135.73, 133.40, 132.48 (q, *J* = 31.50 Hz), 130.11, 129.61, 128.91, 125.85 (q, *J* = 3.78 Hz), 125.50, 123.72 (q, *J* = 273.42 Hz), 112.49, 90.50. HRMS (ESI): calcd for C_18_H_10_ClF_3_N_4_OH (M+H^+^) 391.05680; found 391.05673.

- 1. 3,5-dichloro-*N*-(1-(4-chlorophenyl)-4-cyano-1*H*-pyrazol-5-yl)benzamide **(Py-22)**

Synthesize according to general procedure A. Pale yellow solid, 61% yield, mp 203±1 ℃; ^1^H NMR (500 MHz, DMSO-*d*_6_) δ 11.37 (brs, 1H), 8.36 (s, 1H), 7.93 (t, *J* = 1.9 Hz, 1H), 7.91 (d, *J* = 1.9 Hz, 2H), 7.64 - 7.59 (m, 4H). ^13^C NMR (126 MHz, DMSO-*d*_6_) δ 163.27, 142.85, 136.43, 135.52, 134.62, 133.21, 132.08, 129.56, 127.78, 126.79, 125.44, 112.63, 89.80. HRMS (ESI): calcd for C_17_H_9_Cl_3_N_4_OH (M+H^+^) 390.99147; found 390.99142.

- 1. Ethyl 4-cyano-5-(3,5-dichlorobenzamido)-1-(4-fluorophenyl)-1*H*-pyrazole-3-carboxylate **(Py-23)**

Synthesize according to general procedure A. Pale yellow solid, 38% yield, mp 138±1 ℃; ^1^H NMR (500 MHz, DMSO-*d*_6_) δ 11.49 (brs, 1H), 7.96 (t, *J* = 1.9 Hz, 1H), 7.89 (d, *J* = 1.9 Hz, 2H), 7.68 - 7.65 (m, 2H), 7.43 - 7.39 (m, 2H), 4.41 (q, *J* = 7.1 Hz, 2H), 1.34 (t, *J* = 7.1 Hz, 3H). ^13^C NMR (126 MHz, DMSO-*d*_6_) δ 163.77, 162.25 (d, *J* = 246.96 Hz), 159.60, 143.82, 143.15, 135.27, 135.18, 133.71 (d, *J* = 2.52 Hz), 132.88, 127.28, 127.27 (d, *J* = 8.82 Hz), 117.14 (d, *J* = 22.68 Hz), 111.61, 92.31, 62.21, 14.44. HRMS (ESI): calcd for C_20_H_13_Cl_2_FN_4_O_3_H (M+H^+^) 447.04215; found 447.04227.

- 1. 3,5-dichloro-*N*-(4-cyano-1-(4-nitrophenyl)-1*H*-pyrazol-5-yl)benzamide **(Py-24)**

Synthesize according to general procedure A. White solid, 31% yield, mp 211±1 ℃; ^1^H NMR (500 MHz, DMSO-*d*_6_) δ 11.53 (brs, 1H), 8.48 (s, 1H), 8.37 (d, *J* = 9.1 Hz, 2H), 8.01 - 7.88 (m, 5H). ^13^C NMR (126 MHz, DMSO-*d*_6_) δ 163.24, 146.80, 143.63, 142.43, 141.28, 135.09, 134.71, 132.34, 126.91, 125.20, 124.36, 112.25, 90.73. HRMS (ESI): calcd for C_17_H_9_Cl_2_N_5_O_3_H (M+H^+^) 402.01552; found 402.01553.

- 1. 3,5-dichloro-*N*-(1-(4-chlorophenyl)-3-cyano-1*H*-pyrazol-5-yl)benzamide **(Py-25)**

Synthesize according to general procedure A. Pale yellow solid, 63% yield, mp 226±1 ℃; ^1^H NMR (500 MHz, DMSO-*d*_6_) δ 10.98 (brs, 1H), 7.89 (t, *J* = 1.9 Hz, 1H), 7.85 (d, *J* = 1.9 Hz, 2H), 7.66 (d, *J* = 8.9 Hz, 2H), 7.61 (d, *J* = 8.9 Hz, 2H), 7.23 (s, 1H). ^13^C NMR (126 MHz, DMSO-*d*_6_) δ 163.4, 137.6, 136.4, 135.7, 134.5, 133.7, 131.9, 129.6, 126.7, 125.9, 124.6, 113.9, 108.1. HRMS (ESI): calcd for C_17_H_9_Cl_3_N_4_OH (M+H^+^) 390.99147; found 390.99146.

- 1. 3,5-dichloro-*N*-(3-cyano-1-(2,4,6-trichlorophenyl)-1*H*-pyrazol-5-yl)benzamide **(Py-26)**

Synthesize according to general procedure A. Pale yellow solid, 53% yield, mp 268±1 ℃; ^1^H NMR (500 MHz, DMSO-*d*_6_) δ 11.14 (brs, 1H), 8.03 (s, 2H), 7.88 (t, *J* = 2.0 Hz, 1H), 7.70 (d, *J* = 1.9 Hz, 2H), 7.32 (s, 1H). ^13^C NMR (126 MHz, DMSO-*d*_6_) δ 163.10, 136.64, 134.67, 134.37, 131.75, 131.59, 129.26, 126.80, 126.10, 113.60, 104.04. HRMS (ESI): calcd for C_17_H_7_Cl_5_N_4_OH (M+H^+^) 458.91353; found 458.91345.

- 1. 3,5-dichloro-*N*-(3-cyano-1-(2,6-dichloro-4-(trifluoromethyl)phenyl)-1*H*-pyrazol-5-yl)benz-amide **(Py-27)**

Synthesize according to general procedure A. White solid, 95% yield, mp 249±1 ℃; ^1^H NMR (500 MHz, DMSO-*d*_6_) δ 11.20 (brs, 1H), 8.31 (s, 2H), 7.88 (t, *J* = 1.9 Hz, 1H), 7.69 (d, *J* = 1.9 Hz, 2H), 7.35 (s, 1H). ^13^C NMR (126 MHz, DMSO-*d*_6_) δ 163.05, 139.83, 136.12, 135.93, 135.12, 134.38, 132.83 (q, *J* = 34.02 Hz), 131.80, 126.77, 126.67 (q, *J* = 3.78 Hz), 126.45, 122.26 (q, *J* = 273.42 Hz), 113.47, 104.15. HRMS (ESI): calcd for C_18_H_7_Cl_4_F_3_N_4_OH (M+H^+^) 492.93988; found 492.93991.

- 1. 3,5-dichloro-*N*-(1-(4-chloro-2-fluorophenyl)-3-cyano-1*H*-pyrazol-5-yl)benzamide **(Py-28)**

Synthesize according to general procedure A. Pale yellow solid, 58% yield, mp 205±1 ℃; ^1^H NMR (500 MHz, DMSO-*d*_6_) δ 11.03 (brs, 1H), 7.89 (t, *J* = 1.9 Hz, 1H), 7.80 (dd, *J* = 10.2, 2.3 Hz, 1H), 7.78 (d, *J* = 1.9 Hz, 2H), 7.70 (t, *J* = 8.4 Hz, 1H), 7.50 - 7.48 (m, 1H), 7.25 (s, 1H). ^13^C NMR (126 MHz, DMSO-*d*_6_) δ 163.09, 156.02 (d, *J* = 255.78 Hz), 139.17, 135.76, 135.58 (d, *J* = 10.08 Hz), 134.48, 131.86, 129.73, 126.63, 125.61 (d, *J* = 2.52 Hz), 125.53, 124.22 (d, *J* = 11.34 Hz), 117.75 (d, *J* = 22.68 Hz), 113.64, 106.28. HRMS (ESI): calcd for C_17_H_8_Cl_3_FN_4_OH (M+H^+^) 408.98205; found 408.98209.

- 1. 3,5-dichloro-*N*-(1-(2-chloro-5-methylphenyl)-3-cyano-1*H*-pyrazol-5-yl)benzamide **(Py-29)**

Synthesize according to general procedure A. Beige solid, 43% yield, mp 213±1 ℃; ^1^H NMR (500 MHz, DMSO-*d*_6_) δ 10.95 (brs, 1H), 7.86 (t, *J* = 1.9 Hz, 1H), 7.73 (d, *J* = 2.0 Hz, 2H), 7.57 (d, *J* = 8.2 Hz, 1H), 7.49 (d, *J* = 2.1 Hz, 1H), 7.42 - 7.39 (m, 1H), 7.24 (s, 1H), 2.36 (s, 3H). ^13^C NMR (126 MHz, DMSO-*d*_6_) δ 163.11, 138.90, 138.23, 136.04, 134.36, 134.21, 132.46, 131.71, 130.09, 129.94, 127.04, 126.67, 124.79, 113.85, 105.84, 20.09. HRMS (ESI): calcd for C_18_H_11_Cl_3_N_4_OH (M+H^+^) 405.00712; found 405.00708.

- 1. 3,5-dichloro-*N*-(3-cyano-1-(*p*-tolyl)-1*H*-pyrazol-5-yl)benzamide **(Py-30)**

Synthesize according to general procedure A. Beige solid, 76% yield, mp 185±1 ℃; ^1^H NMR (500 MHz, DMSO-*d*_6_) δ 10.89 (brs, 1H), 7.90 (t, *J* = 1.9 Hz, 1H), 7.85 (d, *J* = 1.9 Hz, 2H), 7.48 (d, *J* = 8.4 Hz, 2H), 7.34 (d, *J* = 8.4 Hz, 2H), 7.21 (s, 1H), 2.35 (s, 3H). ^13^C NMR (126 MHz, DMSO-*d*_6_) δ 163.43, 138.87, 137.17, 135.76, 135.11, 134.51, 131.84, 129.89, 126.64, 124.03, 123.88, 114.01, 108.34, 20.62. HRMS (ESI): calcd for C_18_H_12_Cl_2_N_4_OH (M+H^+^) 371.04609; found 371.04608.

- 1. 3,5-dichloro-*N*-(3-cyano-1-(4-methoxyphenyl)-1*H*-pyrazol-5-yl)benzamide **(Py-31)**

Synthesize according to general procedure A. White solid, 72% yield, mp 165±1 ℃; ^1^H NMR (500 MHz, DMSO-*d*_6_) δ 10.85 (brs, 1H), 7.89 (t, *J* = 1.8 Hz, 1H), 7.84 (d, *J* = 2.0 Hz, 2H), 7.51 (d, *J* = 9.0 Hz, 2H), 7.20 (s, 1H), 7.07 (d, *J* = 9.0 Hz, 2H), 3.79 (s, 3H). ^13^C NMR (126 MHz, DMSO-*d*_6_) δ 163.47, 159.57, 137.17, 135.77, 134.51, 131.83, 130.38, 127.77, 126.64, 125.70, 123.79, 114.54, 108.20, 55.53. HRMS (ESI): calcd for C_18_H_12_Cl_2_N_4_O_2_H (M+H^+^) 387.04101; found 387.04105.

- 1. 3,5-dichloro-*N*-(3-cyano-1-(2,4-dimethylphenyl)-1*H*-pyrazol-5-yl)benzamide **(Py-32)**

Synthesize according to general procedure A. Pale yellow solid, 60% yield, mp 187±1 ℃; ^1^H NMR (500 MHz, DMSO-*d*_6_) δ 10.72 (brs, 1H), 7.85 (s, 1H), 7.73 (d, *J* = 2.0 Hz, 2H), 7.26 - 7.16 (m, 3H), 7.13 (dd, *J* = 8.1, 2.0 Hz, 1H), 2.31 (s, 3H), 2.03 (s, 3H). ^13^C NMR (126 MHz, DMSO-*d*_6_) δ 163.44, 139.72, 138.19, 135.79, 134.91, 134.44, 133.63, 131.74, 131.66, 127.02, 126.78, 126.52, 123.94, 114.11, 107.12, 20.66, 16.92. HRMS (ESI): calcd for C_19_H_14_Cl_2_N_4_OH (M+H^+^) 385.06174; found 385.06171.

- 1. *N*-(3-cyano-1-(2,6-dichloro-4-(trifluoromethyl)phenyl)-1*H*-pyrazol-5-yl)-4-iodobenzamide **(Py-33)**

Synthesize according to general procedure B. White solid, 57% yield, mp 252±1 ℃; ^1^H NMR (500 MHz, DMSO-*d*_6_) δ 11.07 (brs, 1H), 8.28 (s, 2H), 7.90 (d, *J* = 8.5 Hz, 2H), 7.48 (d, *J* = 8.4 Hz, 2H), 7.36 (s, 1H). ^13^C NMR (126 MHz, DMSO-*d*_6_) δ 165.02, 140.27, 137.45, 136.00, 135.14, 132.74 (q, *J* = 34.02 Hz), 129.84, 126.62 (q, *J* = 3.78 Hz), 126.38, 122.26 (q, *J* = 274.68 Hz), 113.55, 103.49, 100.44. HRMS (ESI): calcd for C_18_H_8_Cl_2_F_3_IN_4_OH (M+H^+^) 550.91447; found 550.91455.

- 1. *N*-(3-cyano-1-(2,6-dichloro-4-(trifluoromethyl)phenyl)-1*H*-pyrazol-5-yl)-3,5-difluorobenz-amide **(Py-34)**

Synthesize according to general procedure B. White solid, 84% yield, mp 223±1 ℃; ^1^H NMR (500 MHz, DMSO-*d*_6_) δ 11.15 (brs, 1H), 8.30 (s, 2H), 7.58 - 7.53 (m, 1H), 7.43 - 7.39 (m, 2H), 7.37 (s, 1H). ^13^C NMR (126 MHz, DMSO-*d*_6_) δ 163.17 (t, *J* = 2.52 Hz), 163.05 (d, *J* = 12.6 Hz), 161.08 (d, *J* = 12.6 Hz), 139.82, 136.17 (t, *J* = 8.82 Hz), 135.89, 135.08, 132.81 (q, *J* = 34.02 Hz), 126.68 (q, *J* = 3.78 Hz), 126.44, 122.25 (q, *J* = 274.68 Hz), 113.46, 111.55 (d, *J* = 27.72 Hz), 111.54 (d, *J* = 13.86 Hz), 108.01 (t, *J* = 25.2 Hz), 104.12. HRMS (ESI): calcd for C_18_H_7_Cl_2_F_5_N_4_OH (M+H^+^) 460.99898; found 460.99899.

- 1. 2,4-dichloro-*N*-(3-cyano-1-(2,6-dichloro-4-(trifluoromethyl)phenyl)-1*H*-pyrazol-5-yl)benzamide **(Py-35)**

Synthesize according to general procedure B. White solid, 69% yield, mp 234±1 ℃; ^1^H NMR (500 MHz, DMSO-*d*_6_) δ 11.28 (brs, 1H), 8.30 (s, 2H), 7.74 (d, *J* = 1.8 Hz, 1H), 7.55 (dd, *J* = 8.3, 1.9 Hz, 1H), 7.52 (d, *J* = 8.3 Hz, 1H), 7.41 (s, 1H). ^13^C NMR (126 MHz, DMSO-*d*_6_) δ 163.48, 139.66, 135.73, 135.63, 135.55, 133.78, 133.14 (q, *J* = 34.02 Hz), 131.24, 130.46, 129.34, 127.60, 126.59 (q, *J* = 3.78 Hz), 122.25 (q, *J* = 274.68 Hz), 113.47, 102.50. HRMS (ESI): calcd for C_18_H_7_Cl_4_F_3_N_4_OH (M+H^+^) 492.93988; found 492.93991.

- 1. *N*-(3-cyano-1-(2,6-dichloro-4-(trifluoromethyl)phenyl)-1*H*-pyrazol-5-yl)-4-(trifluorometh-oxy)benzamide **(Py-36)**

Synthesize according to general procedure A. Pale yellow solid, 41% yield, mp 203±1 ℃; ^1^H NMR (500 MHz, Chloroform-*d*) δ 7.83 (s, 2H), 7.74 (d, *J* = 8.8 Hz, 2H), 7.67 (brs, 1H), 7.30 (d, *J* = 8.3 Hz, 2H), 7.16 (s, 1H). ^13^C NMR (126 MHz, Chloroform-*d*) δ 163.07, 152.59 (q, *J* = 2.52 Hz), 138.27, 136.11, 135.05, 134.86 (q, *J* = 35.28 Hz), 130.46, 129.41, 127.92, 126.54 (q, *J* = 3.78 Hz), 121.83 (q, *J* = 274.68 Hz), 120.95, 120.19 (q, *J* = 259.56 Hz), 113.01, 103.46. HRMS (ESI): calcd for C_19_H_8_Cl_2_F_6_N_4_O_2_H (M+H^+^) 509.00013; found 509.00009.

- 1. 2-chloro-*N*-(3-cyano-1-(2,6-dichloro-4-(trifluoromethyl)phenyl)-1*H*-pyrazol-5-yl)-4-fluorobenzamide **(Py-37)**

Synthesize according to general procedure B. White solid, 63% yield, mp 242±1 ℃; ^1^H NMR (500 MHz, DMSO-*d*_6_) δ 11.26 (brs, 1H), 8.30 (s, 2H), 7.58 - 7.55 (m, 2H), 7.41 (s, 1H), 7.36 - 7.32 (m, 1H). ^13^C NMR (126 MHz, DMSO-*d*_6_) δ 163.61, 162.69 (d, *J* = 250.74 Hz), 161.69, 139.79, 135.64, 135.61, 133.13 (q, *J* = 34.02 Hz), 131.63 (d, *J* = 3.78 Hz), 131.55 (d, *J* = 11.34 Hz), 131.06 (d, *J* = 8.82 Hz), 126.61 (q, *J* = 3.78 Hz), 122.28 (q, *J* = 273.42 Hz), 117.31 (d, *J* = 26.46 Hz), 114.75 (d, *J* = 22.68 Hz), 113.51, 102.51. HRMS (ESI): calcd for C_18_H_7_Cl_3_F_4_N_4_OH (M+H^+^) 476.96943; found 476.96936.

- 1. *N*-(3-cyano-1-(2,6-dichloro-4-(trifluoromethyl)phenyl)-1*H*-pyrazol-5-yl)-4-(trifluorometh-yl)benzamide **(Py-38)**

Synthesize according to general procedure A. White solid, 75% yield, mp 192±1 ℃; ^1^H NMR (400 MHz, Chloroform-*d*) δ 7.84 - 7.77 (m, 5H), 7.74 (d, *J* = 8.3 Hz, 2H), 7.17 (s, 1H). ^13^C NMR (101 MHz, Chloroform-*d*) δ 163.23, 138.13, 136.09, 135.43, 135.02, 134.90 (q, *J* = 34.6 Hz), 134.71 (q, *J* = 33.1 Hz), 127.90, 127.87, 126.54 (q, *J* = 3.7 Hz), 126.16 (q, *J* = 3.7 Hz), 123.25 (q, *J* = 271.1 Hz), 121.82 (q, *J* = 272.4 Hz), 112.98, 103.60. HRMS (ESI): calcd for C_19_H_8_Cl_2_F_6_N_4_OH (M+H^+^) 493.00521; found 493.00522.

- 1. *N*-(3-cyano-1-(2,6-dichloro-4-(trifluoromethyl)phenyl)-1*H*-pyrazol-5-yl)-4-methylbenz-amide **(Py-39)**

Synthesize according to general procedure B. White solid, 56% yield, mp 254±1 ℃; ^1^H NMR (400 MHz, DMSO-*d*_6_) δ 10.96 (brs, 1H), 8.29 (s, 2H), 7.62 (d, *J* = 8.1 Hz, 2H), 7.34 (s, 1H), 7.31 (d, *J* = 8.0 Hz, 2H), 2.34 (s, 3H). ^13^C NMR (101 MHz, DMSO-*d*_6_) δ 165.40, 142.75, 140.58, 136.10, 135.16, 132.69 (q, *J* = 33.7 Hz), 130.16, 129.04, 128.11, 126.61 (q, *J* = 3.9 Hz), 126.32, 122.29 (q, *J* = 272.4 Hz), 113.63, 103.39, 21.05. HRMS (ESI): calcd for C_19_H_11_Cl_2_F_3_N_4_OH (M+H^+^) 439.03348; found 439.03354.

- 1. *N*-(3-cyano-1-(2,6-dichloro-4-(trifluoromethyl)phenyl)-1*H*-pyrazol-5-yl)-3-(trifluorometh-yl)benzamide **(Py-40)**

Synthesize according to general procedure A. Yellow solid, 62% yield, mp 174±1 ℃; ^1^H NMR (400 MHz, DMSO-*d*_6_) δ 11.26 (brs, 1H), 8.31 (s, 2H), 8.03 - 7.94 (m, 3H), 7.79 - 7.75 (m, 1H), 7.39 (s, 1H). ^13^C NMR (101 MHz, DMSO-*d*_6_) δ 164.34, 140.02, 135.92, 135.13, 133.91, 132.82 (q, *J* = 33.7 Hz), 132.10, 129.98, 129.26 (q, *J* = 32.1 Hz), 129.01 (q, *J* = 3.9 Hz), 126.66 (q, *J* = 3.8 Hz), 126.44, 124.63 (q, *J* = 3.8 Hz), 123.69 (q, *J* = 270.8 Hz), 122.25 (q, *J* = 272.2 Hz), 113.51, 103.92. HRMS (ESI): calcd for C_19_H_8_Cl_2_F_6_N_4_OH (M+H^+^) 493.00521; found 493.00525.

- 1. 3-bromo-*N*-(3-cyano-1-(2,6-dichloro-4-(trifluoromethyl)phenyl)-1*H*-pyrazol-5-yl)benz-amide **(Py-41)**

Synthesize according to general procedure A. Yellow solid, 78% yield, mp 212±1 ℃; ^1^H NMR (500 MHz, DMSO-*d*_6_) δ 11.14 (brs, 1H), 8.31 (s, 2H), 7.85 - 7.79 (m, 2H), 7.71 - 7.69 (m, 1H), 7.50 - 7.46 (m, 1H), 7.36 (s, 1H). ^13^C NMR (126 MHz, DMSO-*d*_6_) δ 164.65, 140.58, 136.45, 135.65, 135.61, 133.25 (q, *J* = 34.02 Hz), 131.31, 131.04, 127.64, 127.12 (q, *J* = 3.78 Hz), 126.87, 122.74 (q, *J* = 274.68 Hz), 122.12, 114.00, 104.26. HRMS (ESI): calcd for C_18_H_8_BrCl_2_F_3_N_4_OH (M+H^+^) 502.92834; found 502.92841.

- 1. *N*-(3-cyano-1-(2,6-dichloro-4-(trifluoromethyl)phenyl)-1*H*-pyrazol-5-yl)-3,5-dinitrobenz-amide **(Py-42)**

Synthesize according to general procedure A. Yellow solid, 47% yield, mp 118±1 ℃; ^1^H NMR (400 MHz, DMSO-*d*_6_) δ 11.59 (brs, 1H), 8.99 (t, *J* = 2.1 Hz, 1H), 8.86 (d, *J* = 2.1 Hz, 2H), 8.32 (s, 2H), 7.41 (s, 1H). ^13^C NMR (101 MHz, DMSO-*d*_6_) δ 162.01, 148.05, 139.51, 135.87, 135.36, 135.07, 132.88 (q, *J* = 34.34 Hz), 128.30, 126.74 (q, *J* = 4.04 Hz), 126.54, 122.23 (q, *J* = 274.72 Hz), 121.88, 113.40, 104.85. HRMS (ESI): calcd for C_18_H_6_Cl_2_F_3_N_6_O_5_ (M-H^+^) 512.97343; found 512.97401.

- 1. *N*-(3-cyano-1-(2,6-dichloro-4-(trifluoromethyl)phenyl)-1*H*-pyrazol-5-yl)-3,5-bis(trifluoro-methyl)benzamide **(Py-43)**

Synthesize according to general procedure A. Yellow solid, 69% yield, mp 185±1 ℃; ^1^H NMR (400 MHz, DMSO-*d*_6_) δ 11.41 (s, 1H), 8.40 (s, 1H), 8.31 (s, 2H), 8.27 (s, 2H), 7.39 (s, 1H). ^13^C NMR (101 MHz, DMSO-*d*_6_) δ 163.07, 139.64, 135.81, 135.22, 135.07, 132.89 (q, *J* = 34.34 Hz), 130.56 (q, *J* = 33.33 Hz), 128.83 (q, *J* = 2.02 Hz), 126.70 (q, *J* = 4.04 Hz), 126.50, 122.86 (q, *J* = 273.71 Hz), 122.22 (q, *J* = 274.72 Hz), 113.44, 104.44. HRMS (ESI): calcd for C_20_H_7_Cl_2_F_9_N_4_OH (M+H^+^) 560.99260; found 560.99261.

- 1. 3,5-dichloro-*N*-(1-(2,6-dichloro-4-(trifluoromethyl)phenyl)-3-(*N*'-hydroxycarbamimidoyl)-1*H*-pyrazol-5-yl)benzamide **(Py-44)**

In a dry round-bottom flask, dissolve Py-27 (0.60 mmol, 1 eq) in ethanol (10 mL). After stirring at room temperature, sequentially add NH₂OH·HCl (3.00 mmol, 5 eq) and Na₂CO₃ (1.50 mmol, 2.5 eq). After reagent addition, the reaction mixture was refluxed at 80°C. Reaction progress was monitored by thin-layer chromatography. Upon complete consumption of the starting material, the mixture was cooled to room temperature, the solvent was removed under reduced pressure, and the residue was extracted with ethyl acetate. Organic layers were combined, washed with water and saturated saline solution, dried over anhydrous Na₂SO₄, filtered, and concentrated under reduced pressure. The crude product was purified by silica gel column chromatography (PE/EtOAc 2:1) to afford Py-44. White solid, 45% yield; ^1^H NMR (500 MHz, DMSO-*d*_6_) δ 10.87 (brs, 1H), 9.78 (brs, 1H), 8.24 (s, 2H), 7.86 (t, *J* = 2.0 Hz, 1H), 7.69 (d, *J* = 1.9 Hz, 2H), 6.81 (s, 1H), 5.55 (brs, 2H). ^13^C NMR (126 MHz, DMSO-*d*_6_) δ 162.77, 147.13, 145.87, 138.86, 137.19, 136.67, 135.59, 134.29, 132.00 (q, *J* = 34.02 Hz), 131.48, 126.70, 126.35 (q, *J* = 3.78 Hz), 122.38 (q, *J* = 274.68 Hz), 96.23. HRMS (ESI): calcd for C_18_H_10_Cl_4_F_3_N_5_O_2_H (M+H^+^) 525.96135; found 525.95886.

- 1. 1-(2,6-dichloro-4-(trifluoromethyl)phenyl)-5-(3,5-dichlorobenzamido)-1*H*-pyrazole-3-carboxamide **(Py-45)**

In a dry round-bottom flask, dissolve Py-27 (0.40 mmol, 1 eq) in acetone (10 mL). Stir at room temperature, then add 0.1 M NaOH solution (20 mL). After adding the reagent, reflux the reaction mixture at 60°C. Monitor the reaction progress by thin-layer chromatography. Upon complete consumption of the starting material, the mixture was cooled to room temperature. The solvent was removed under reduced pressure, and the residue was extracted with ethyl acetate. Organic layers were pooled, washed with water and saturated saline solution, dried over anhydrous Na₂SO₄, filtered, and concentrated under reduced pressure. The crude product was purified by silica gel column chromatography (PE/EtOAc 4:1) to afford Py-45. White solid, 41% yield; ^1^H NMR (500 MHz, DMSO-*d*_6_) δ 10.94 (brs, 1H), 8.26 (s, 2H), 7.87 (t, *J* = 1.9 Hz, 1H), 7.71 (brs, 1H), 7.70 (d, *J* = 1.9 Hz, 2H), 7.44 (brs, 1H), 7.02 (s, 1H). ^13^C NMR (126 MHz, DMSO-*d*_6_) δ 162.85, 162.35, 148.46, 139.25, 136.88, 136.57, 135.48, 134.31, 132.25 (q, *J* = 34.02 Hz), 131.54, 126.72, 126.39 (q, *J* = 3.78 Hz), 122.34 (q, *J* = 274.68 Hz), 99.49. HRMS (ESI): calcd for C_18_H_9_Cl_4_F_3_N_4_O_2_H (M+H^+^) 510.95045; found 510.95050.

**Text 2. Chemical Synthesis of the probe molecule Py-27-P**

The probe molecule Py-27-P was synthesized according to the procedure outlined below.

**Synthesis of Py-27-1:** 5-Amino-1-(2,6-dichloro-4-(trifluoromethyl)phenyl)-1H-pyrazole-3-carbonitrile (5.5 mmol, 1.1 eq.), N,N'-dicyclohexylcarbodiimide (DCC, 7.5 mmol, 1.5 eq.), 4-dimethylaminopyridine (DMAP, 7.5 mmol, 1.5 eq.), and 3,5-dichloro-4-methoxybenzoic acid (5 mmol, 1 eq.) were added to a dry 100 mL round-bottom flask. The mixture was dissolved in 30 mL of dichloromethane (DCM) and stirred at room temperature overnight. After the reaction was confirmed to be complete by TLC monitoring, the mixture was filtered to remove insoluble impurities. The filtrate was washed with water and brine, then dried over anhydrous sodium sulfate. The DCM was removed by rotary evaporation under reduced pressure. The crude product was recrystallized from an ethyl acetate/petroleum ether system to afford Py-27-1 as a white solid in 57% yield. Mp 229±1 ℃; ^1^H NMR (500 MHz, DMSO-*d*_6_) δ 11.10 (brs, 1H), 8.30 (s, 2H), 7.80 (s, 2H), 7.33 (s, 1H), 3.87 (s, 3H). ^13^C NMR (126 MHz, DMSO-*d*_6_) δ 162.68, 154.79, 139.89, 135.96, 135.08, 132.80 (q, *J* = 34.02 Hz), 130.27, 128.95, 128.61, 126.68 (q, *J* = 3.78 Hz), 126.42, 122.26 (q, *J* = 274.68 Hz), 113.48, 104.28, 60.92. HRMS (ESI): calcd for C_19_H_9_Cl_4_F_3_N_4_O_2_H (M+H^+^) 522.95045; found 522.95050.

**Synthesis of Py-27-2:** Py-27-1 (0.5 mmol, 1 eq) was weighed and transferred into a 25 mL dried round-bottom flask. Under an argon atmosphere, 5 mL of DCM was added to dissolve the substrate, and the mixture was stirred at 0 °C for 10 minutes. Boron tribromide (BBr_3_, 3 mmol, 6 eq) was diluted in 1 mL of DCM, and then slowly added dropwise to the reaction system at 0 °C. The reaction mixture was allowed to warm to room temperature naturally and stirred overnight. After the reaction was completed (monitored by TLC), the system was cooled to 0 °C, and the reaction was quenched by adding water. The mixture was extracted with DCM, and the combined organic phases were washed with saturated sodium bicarbonate solution and saturated brine successively, then dried over anhydrous sodium sulfate. After concentration under reduced pressure, the crude product was purified by column chromatography (PE/EA = 2:1) to afford Py-27-2 as a pale yellow solid in 65% yield. ^1^H NMR (500 MHz, Chloroform-*d*) δ 7.82 (s, 2H), 7.77 (brs, 1H), 7.63 (s, 2H), 7.60 (brs, 1H), 7.08 (s, 1H). HRMS (ESI): calcd for C_18_H_7_Cl_4_F_3_N_4_O_2_H (M+H^+^) 508.93480; found 508.93506.

**Synthesis of Py-27-P:** Py-27-2 (0.15 mmol, 1 eq.) was weighed into a dry 10 mL round-bottom flask and dissolved in 2 mL of DMF. 3-(but-3-yn-1-yl)-3-(2-iodoethyl)-3H-diazirine (0.23 mmol, 1.5 eq.) and triethylamine (0.45 mmol, 3 eq.) were added to the reaction mixture. The reaction was heated to 60°C and stirred in the dark. After completion of the reaction, as monitored by TLC, the mixture was cooled to room temperature and extracted with ethyl acetate. The combined organic phases were washed with water and brine, then dried over anhydrous sodium sulfate. After concentration under reduced pressure, the crude product was purified by column chromatography (PE/EA = 4:1) to afford Py-27-P as a pale yellow liquid in 49% yield. ^1^H NMR (500 MHz, Chloroform-*d*) δ 7.82 (s, 2H), 7.62 (s, 2H), 7.57 (brs, 1H), 7.09 (s, 1H), 3.94 (t, *J* = 6.3 Hz, 2H), 2.07 (td, *J* = 7.5, 2.7 Hz, 2H), 1.99 (t, *J* = 2.7 Hz, 1H), 1.95 (t, *J* = 6.3 Hz, 2H), 1.79 (t, *J* = 7.5 Hz, 2H). ^13^C NMR (126 MHz, Chloroform-*d*) δ 161.80, 155.14, 137.83, 136.01, 135.15, 134.99, 132.75 (q, *J* = 34.02 Hz), 130.43, 129.28, 128.12, 128.07, 126.54 (q, *J* = 3.78 Hz), 122.15 (q, *J* = 274.42 Hz), 112.87, 104.26, 82.63, 69.28, 68.57, 33.84, 32.47, 29.69, 13.32. HRMS (ESI): calcd for C_25_H_15_Cl_4_F_3_N_6_O_2_H (M+H^+^) 629.00355; found 629.00366.

Supplementary Figures


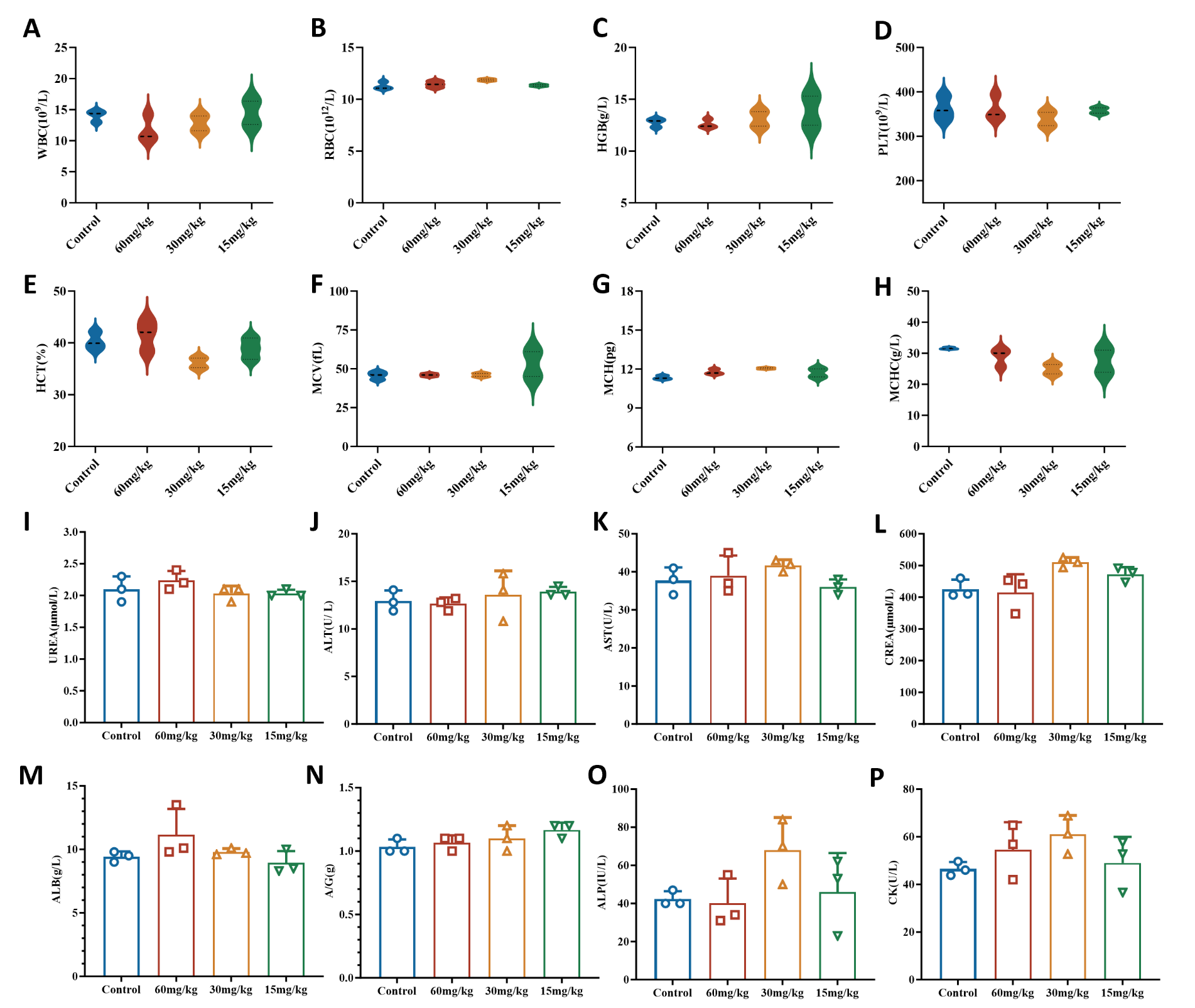


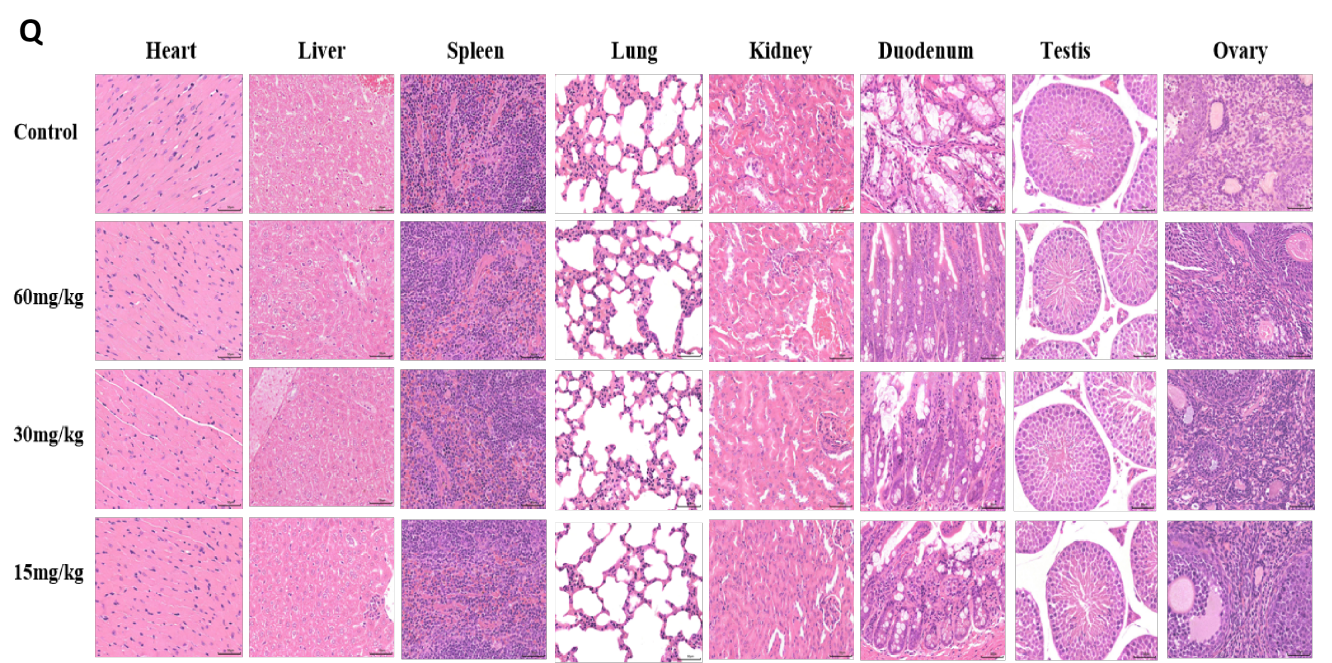


**Fig. S1. Blood routine and blood biochemical results in subchronic toxicity studies.** (**A** to **H**) Blood routine analysis (WBC, white blood cell; RBC, red blood cell; HGB, hemoglobin; PLT, platelet; HCT, hematocrit; MCV, mean cell volume; MCH, mean corpuscular hemoglobin; MCHC, mean corpuscular hemoglobin concentration); (**I** to **P**) Blood biochemistry analysis (UREA, urea; ALT, alanine transferase; AST, aspartate transferase; CREA, creatinine; ALB, albumin; A/G, ALB/GLB; ALP, alkaline phosphatase; CK, creatine kinase) were performed on healthy BALB/c mice 30 days after treatment with Py-27; (**Q**) H&E staining of Heart, Liver, Spleen, Lung, Kidney, Duodenum, Testis and Ovary of healthy BALB/c mice in each group on day 30. All experiments were performed as three biologically independent experiments. Data were presented as mean ± SD..


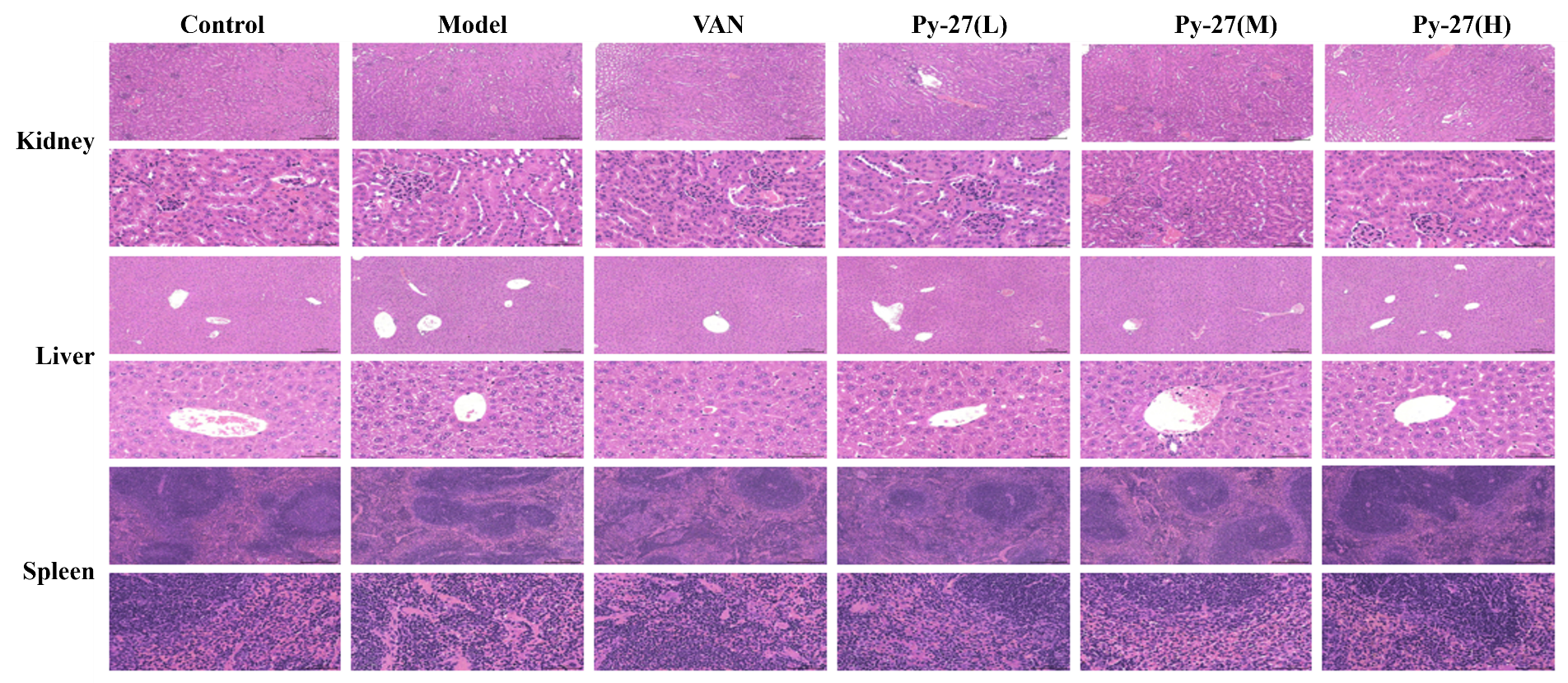


Fig. S2. H&E staining of Kidney, Liver and Spleen in acute pneumonia infection model.


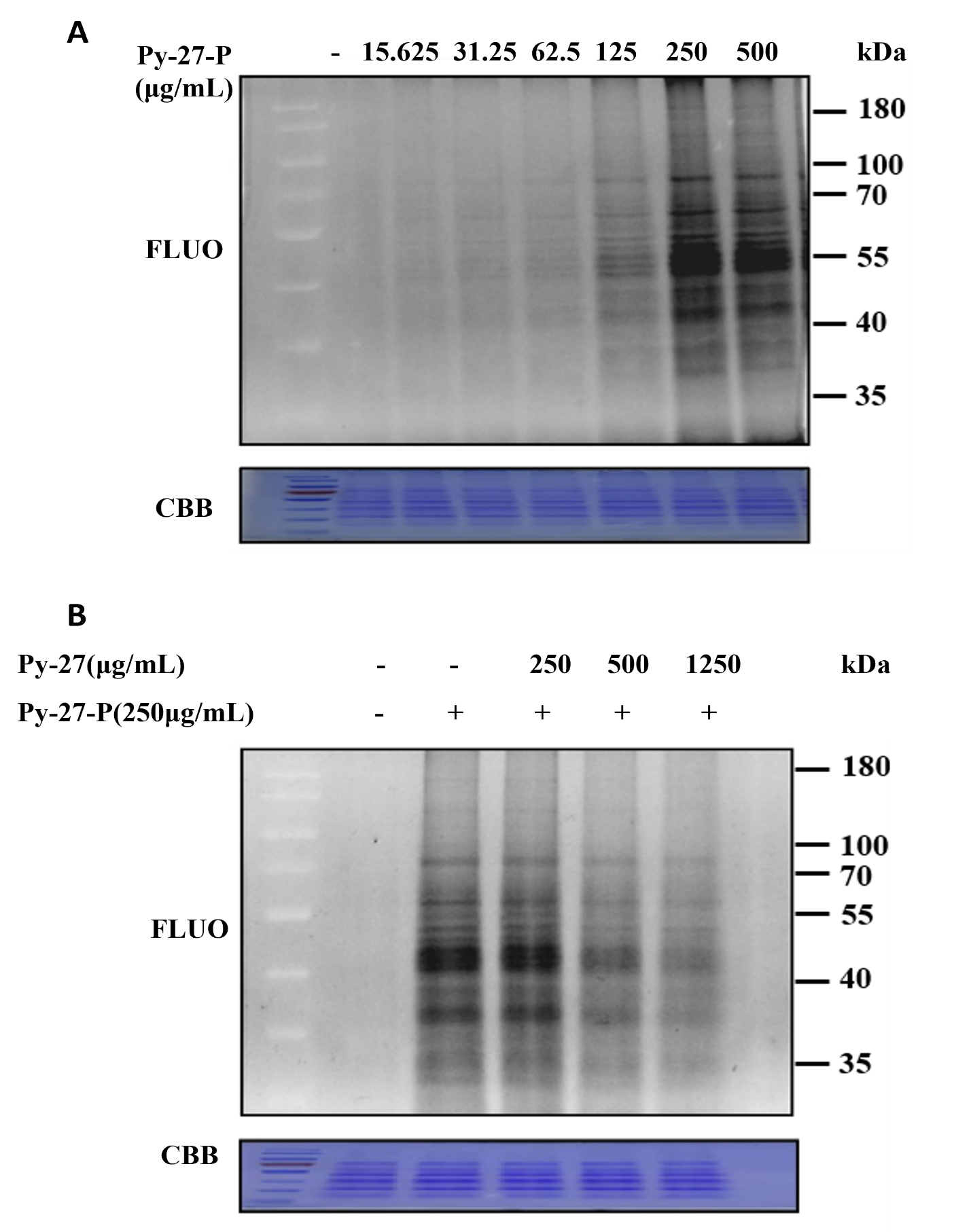


Fig. S3. Probe labeling optimization in the MRSA. (**A**) Dose-dependent *in situ* protein labeling with Py-27-P; (**B**) Py-27 competes with Py-27-P for *in situ* protein labeling.

**(A)**


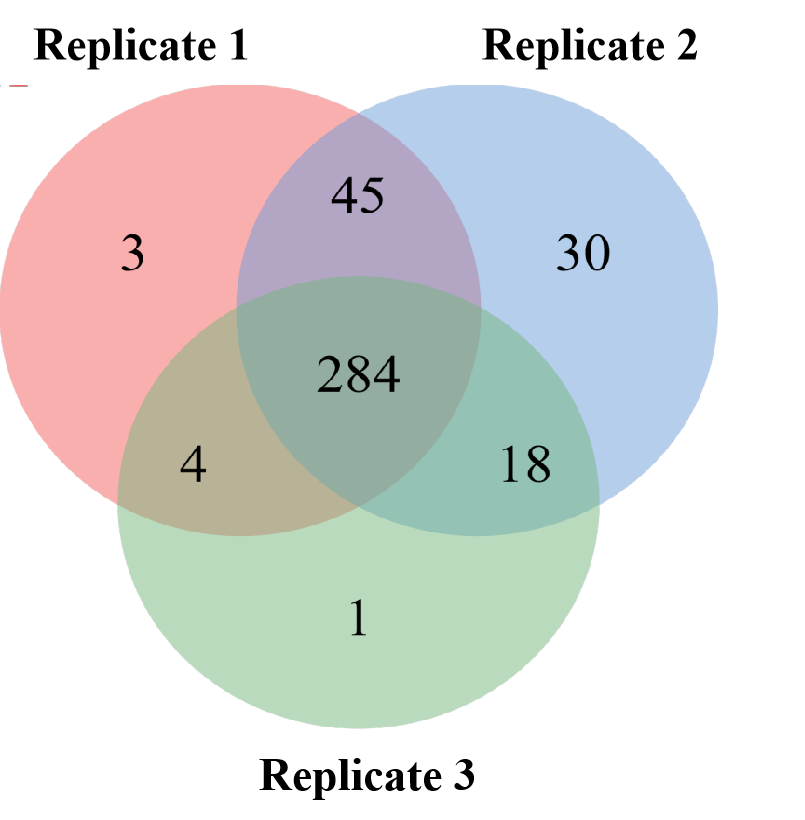


**(B)**


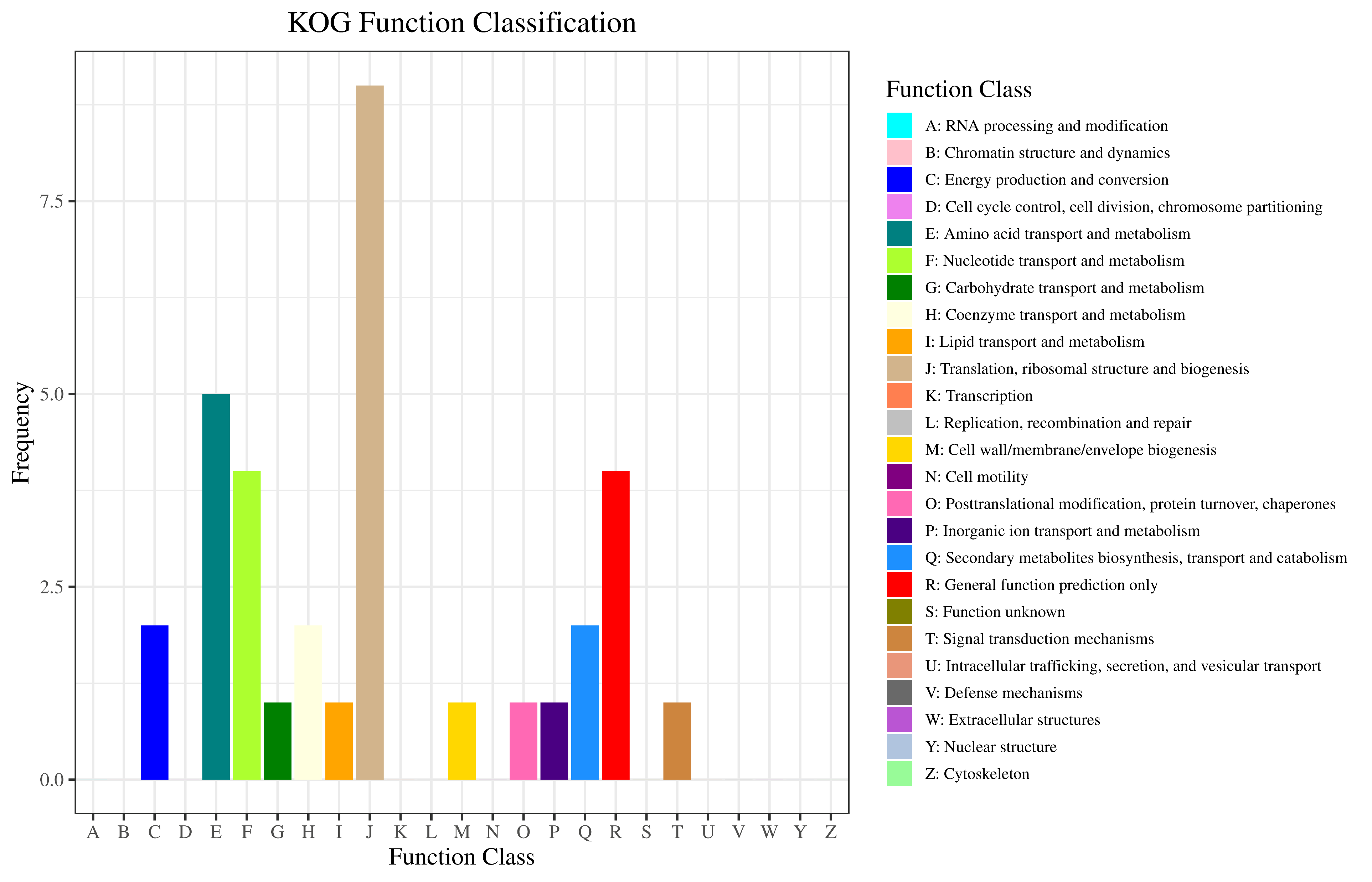


**Fig. S4. Based on the mass spectrometry analysis results.** (A) Venn diagrams showing the proteins with high peptide. (B) KOG classification of proteins with high peptide.

**(A)**

**
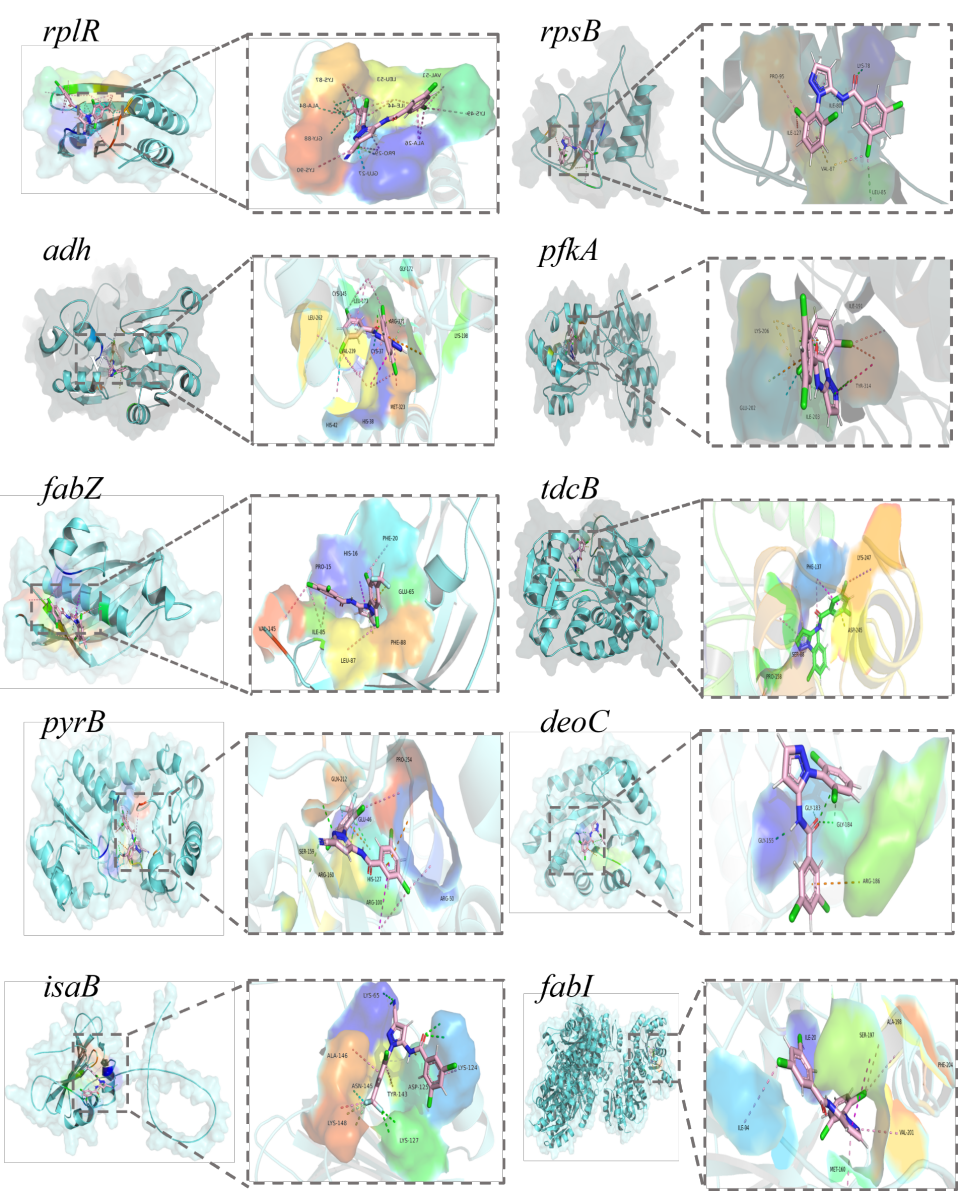
**

**(B)
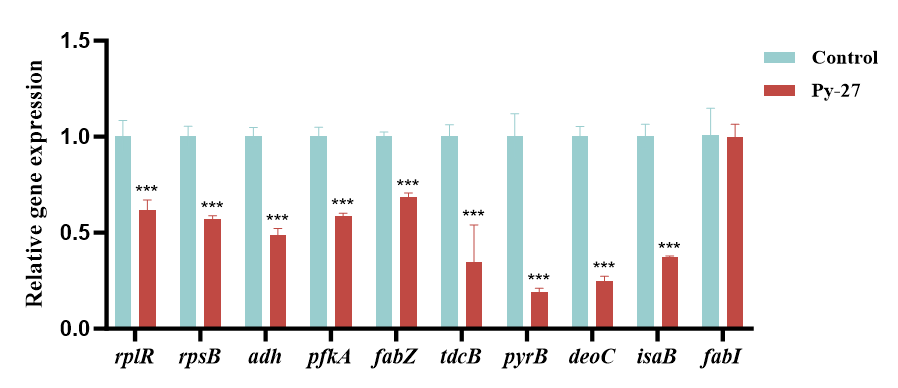
**

**Fig. S5. Molecular docking and qPCR veriﬁcations.** (**A**) Molecular docking of top ten possible target proteins; (**B**) qPCR veriﬁcations on gene after the treatment of Py-27. All experiments were performed as three biologically independent experiments. Data were presented as mean ± SD. Statistical signiﬁcance was analyzed by ordinary two-way ANOVA (^***^*P* < 0.001).

**
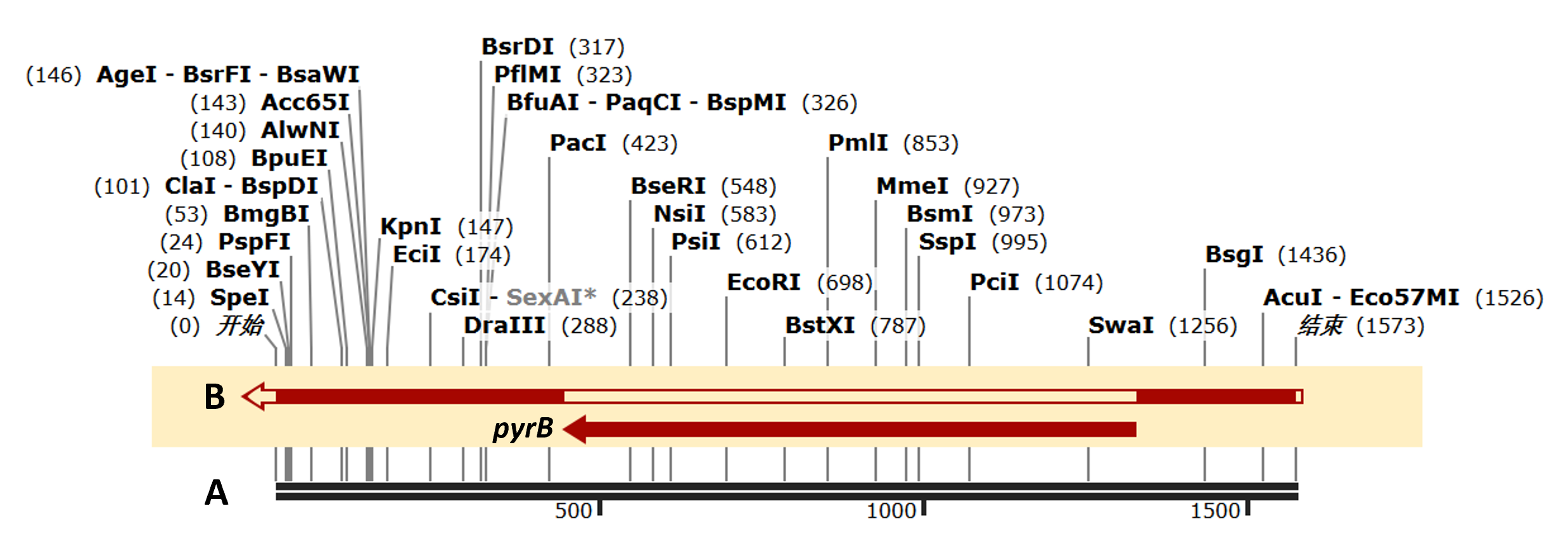
**

**Fig. S6. DNA mutations were identified by sequence alignment between wild type (A) and Δ*pyrB* (B).**

**(A)**

**
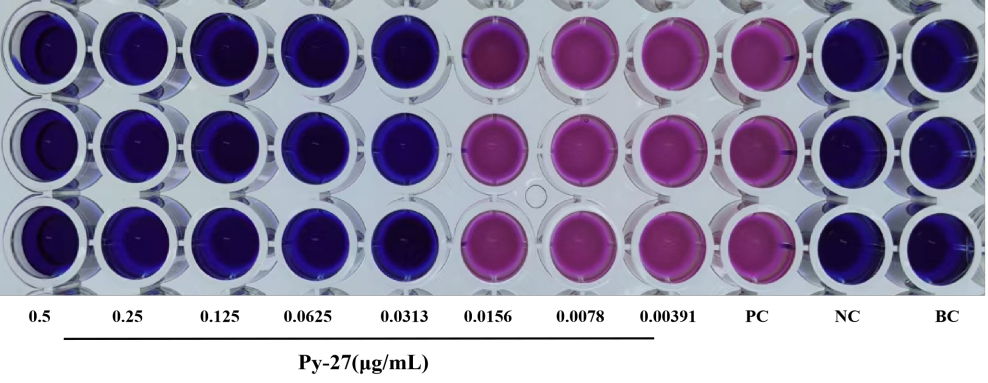
**

**(B)**

**
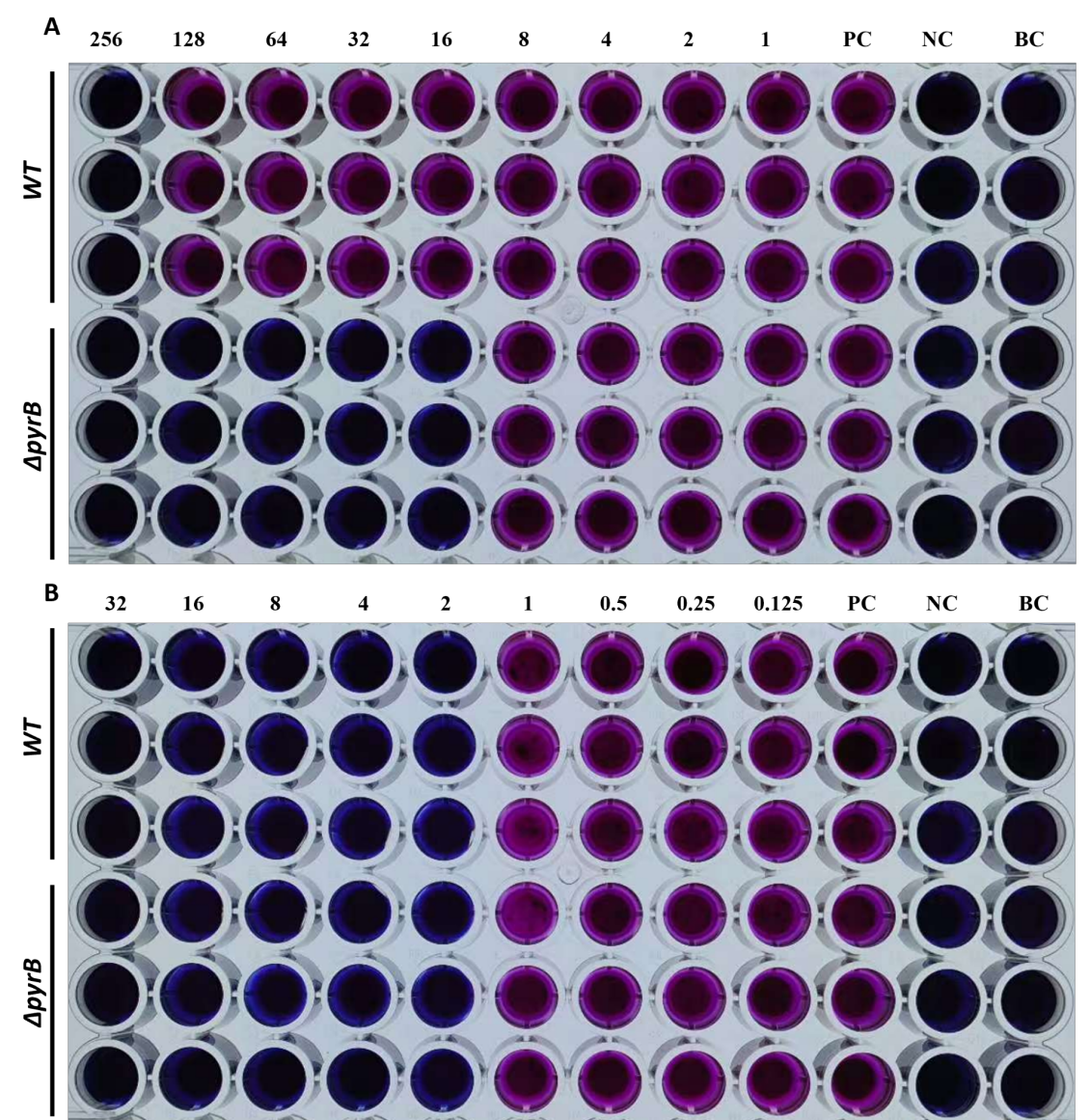
**

**(C)**

**
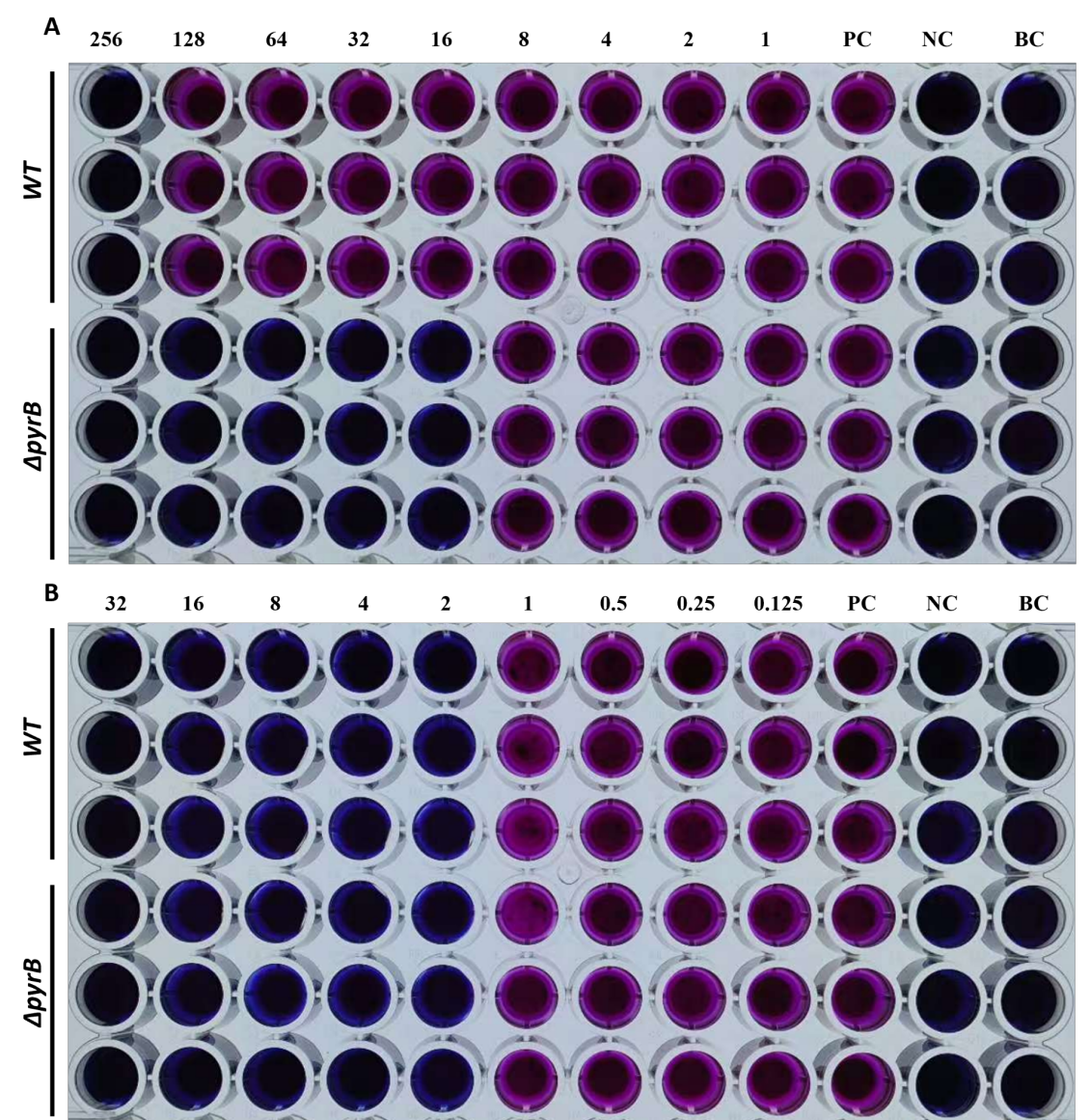
**

**Fig. S7. MIC of Py-27 against Δ*pyrB* (A), MIC of Ampicillin (B) and Vancomycin (C) against WT and Δ*pyrB*.** PC: positive control (MH containing MRSA without Ampicillin or Vancomycin); NC: negative control (MH containing Ampicillin or Vancomycin without MRSA); BC: blank control (MH only).


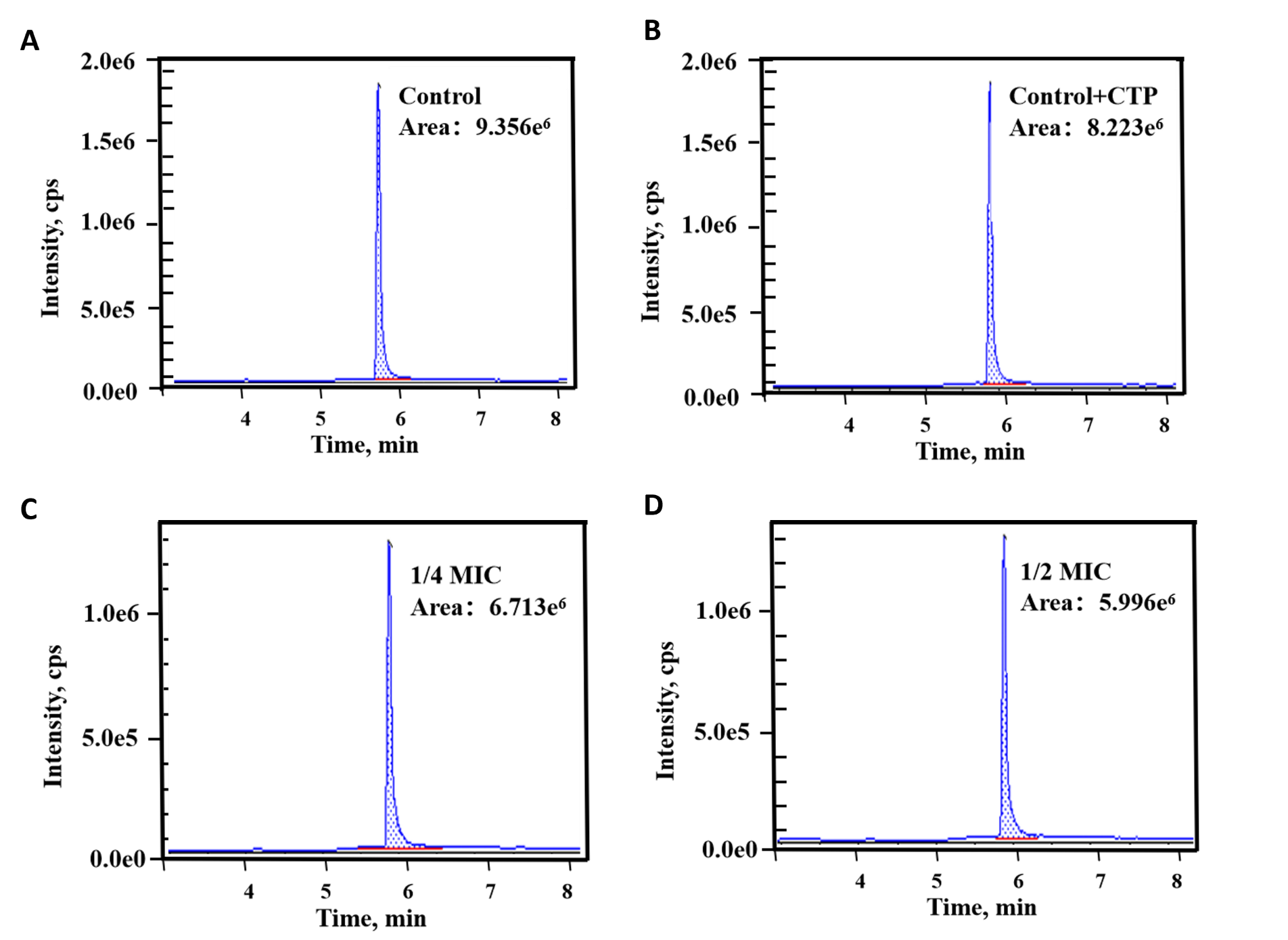


**Fig. S8. The content of N-carbamoyl-L-aspartate was determined using LC/MS.** (**A**) Control; (**B**) CTP; (**C**) 1/4×MIC Py-27; (**D**) 1/2×MIC Py-27.


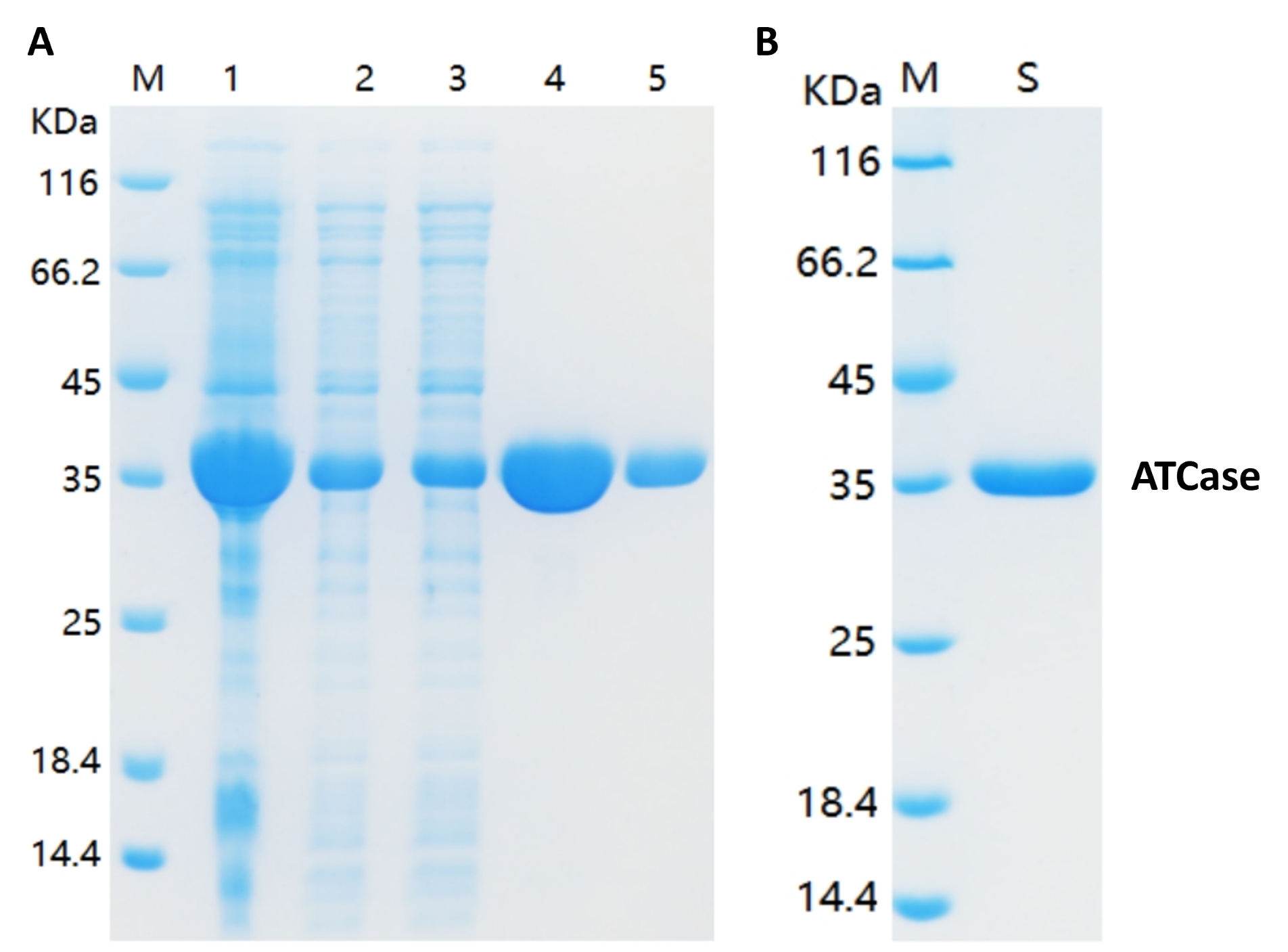


**Fig. S9. ATCase protein expression and purification.** (**A**) The result of the affinity purification; (**B**) The result of protein dialysis.

**
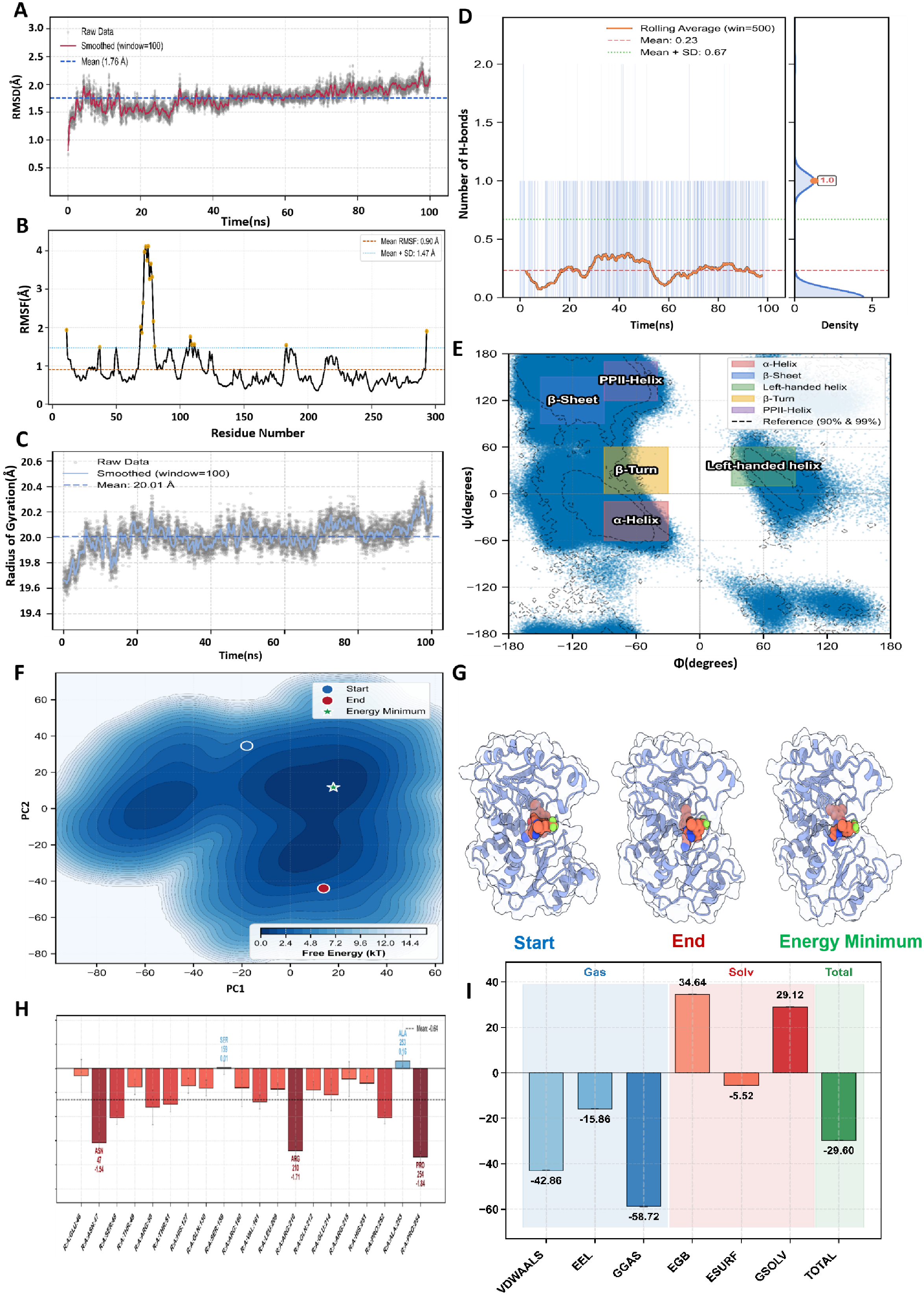
**

**Fig. S10. Binding Mode and Stability of the ATCase and Py-27.** Binding Mode and Stability of the ATCase and Py-27. **(A)** RMSD variation curves of the complexes during 100 ns kinetic simulations; **(B)** RMSF values of amino acid residues in the complex system; **(C)** Rg of backbone atoms; **(D)** Number of hydrogen bonds between ATCase and Py-27; **(E)** Ramachandran of ATCase; **(F)** Free energy landscape; **(G)** Structures of Py-27 with ATCase at Start, End and Energy Minimum; **(H)** Decomposition of MM-GBSA free energy for each residue; **(I)** MM/GBSA binding free energy.

**(A)**


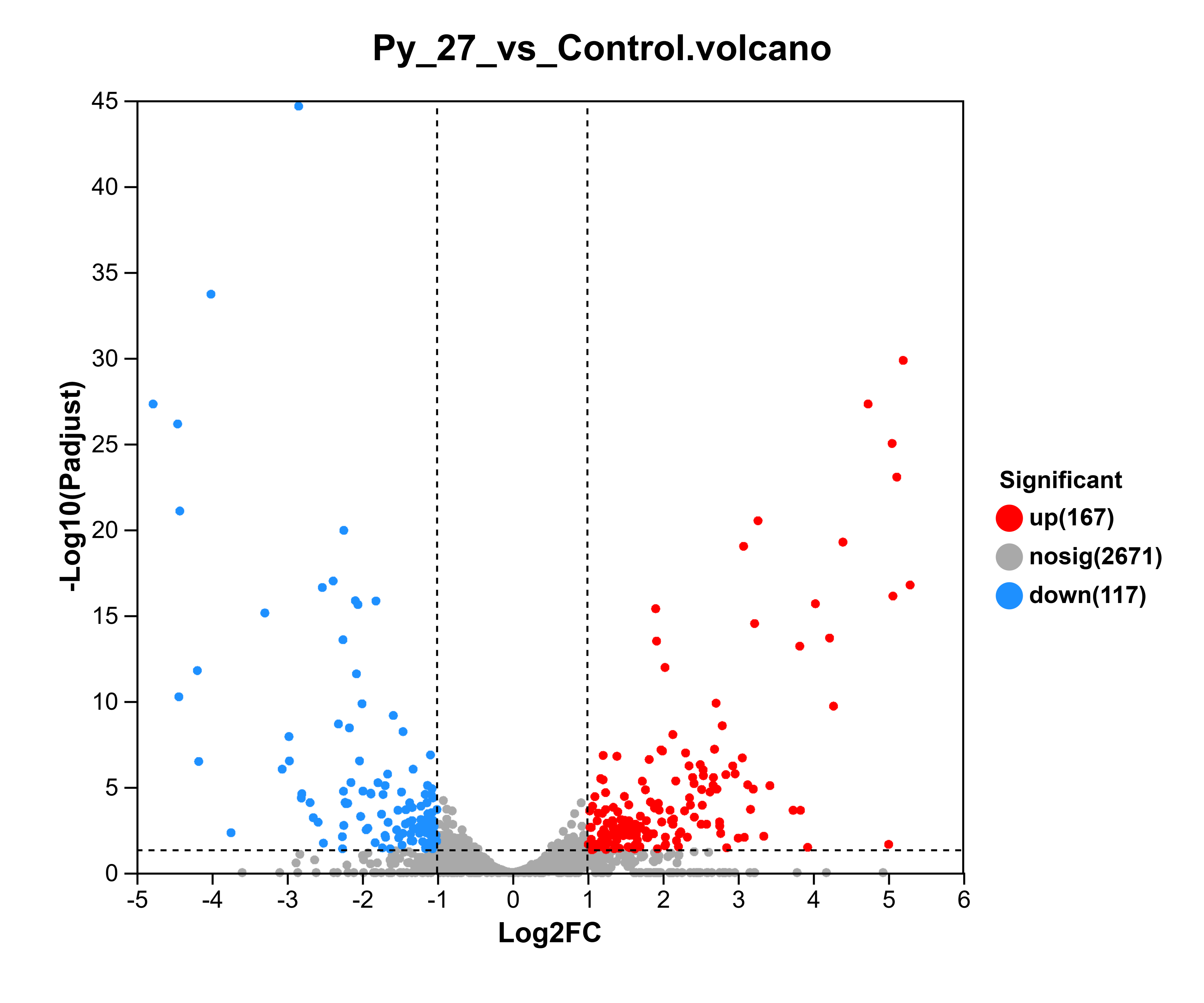


**(B)**

**
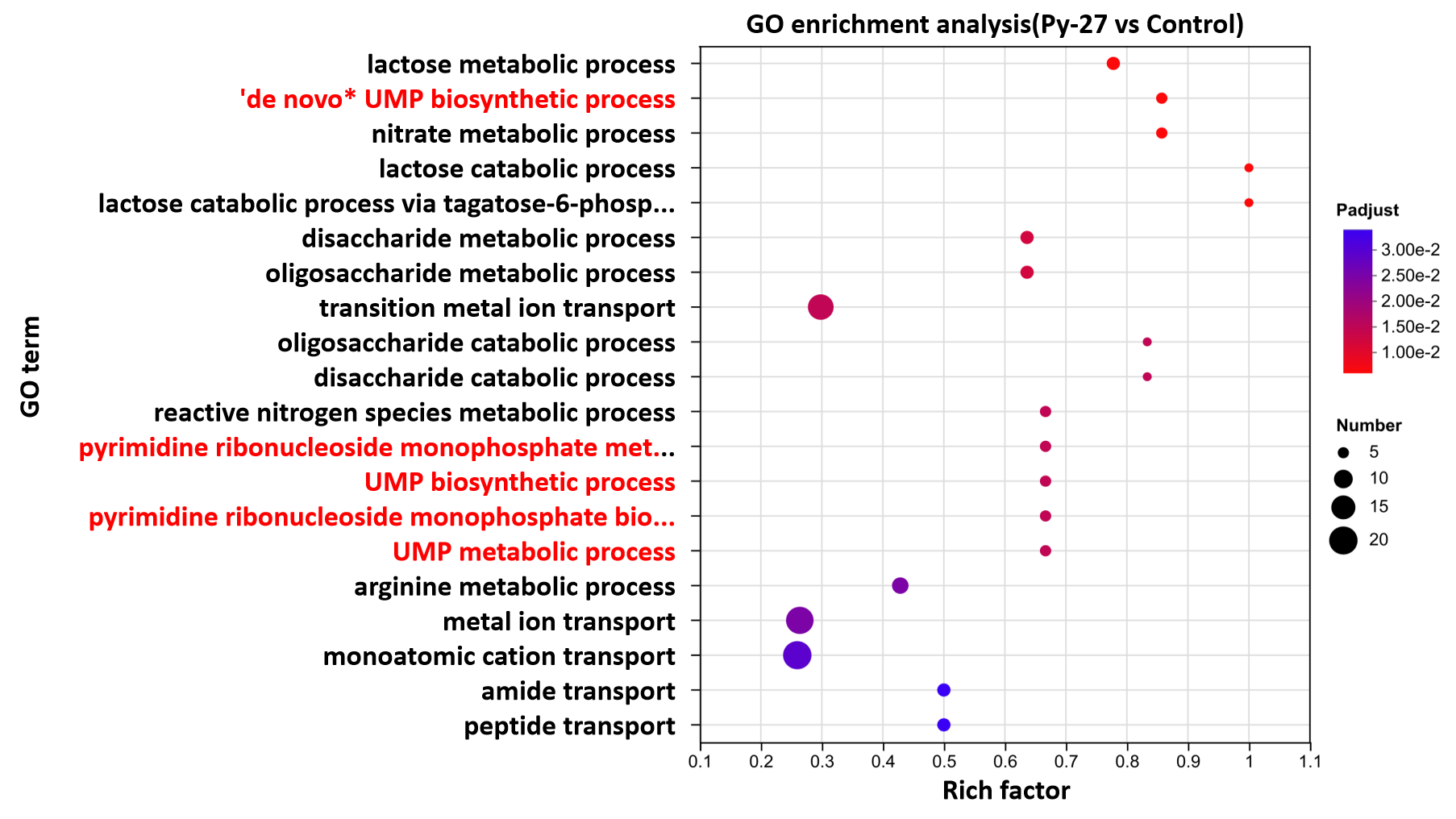
**

**(C)**

**
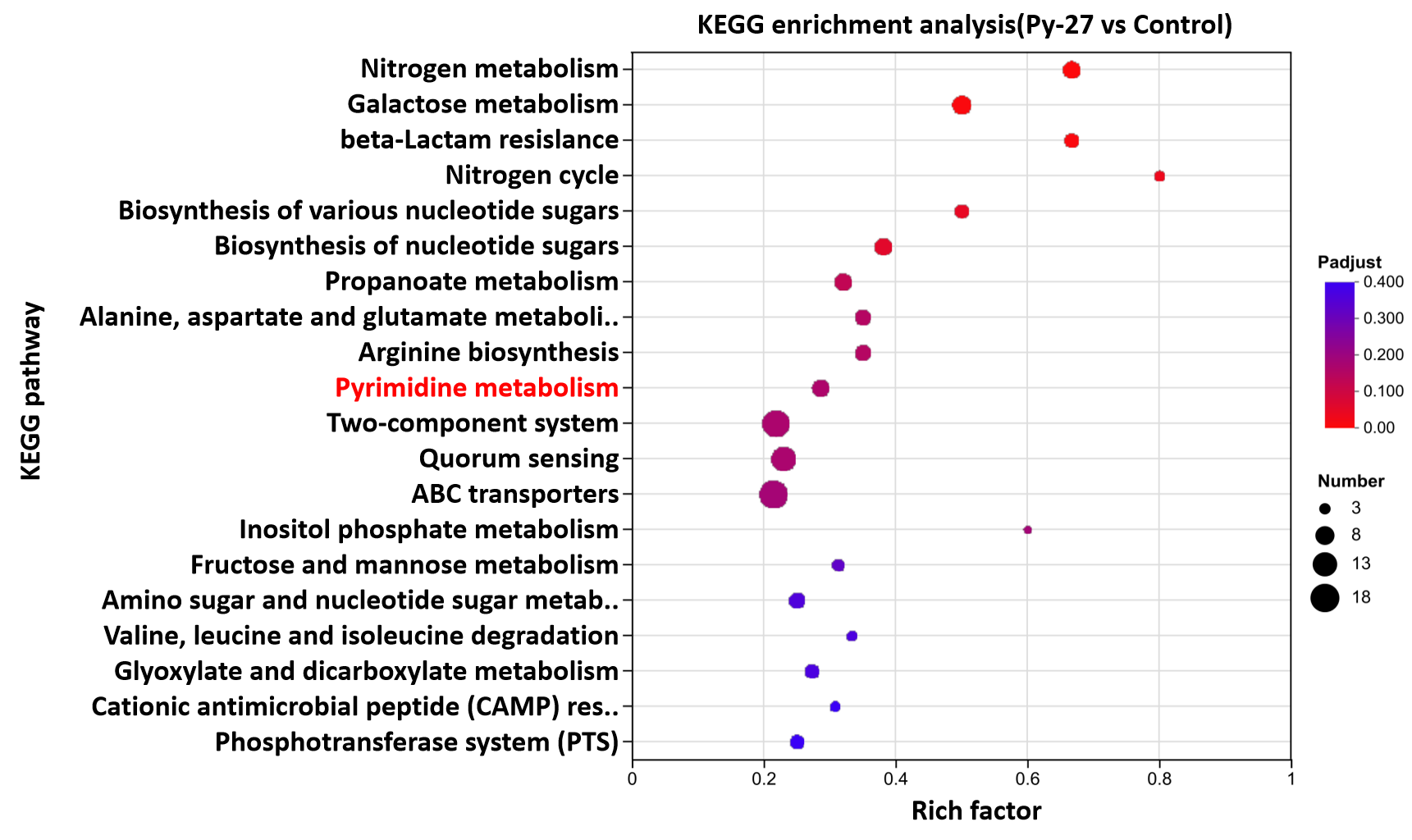
**

**Fig. S11. Transcriptomic results analysis.** (A) Volcano plot analyses of total DEGs in MRSA after treatment with blank TSB or Py-27. Blue and red dots represent downregulated and upregulated genes, respectively. Gray dots represent the genes that were not statistically different. (B) GO analysis of DEGs. (C) KEGG analysis of DEGs.

**Fig. S12. Trends in resistance development.** Py-27, vancomycin (VAN), and ciprofloxacin (CIP) after 20 successive subculturing passages (n=3). Data are presented as mean ± SD.

**(A)
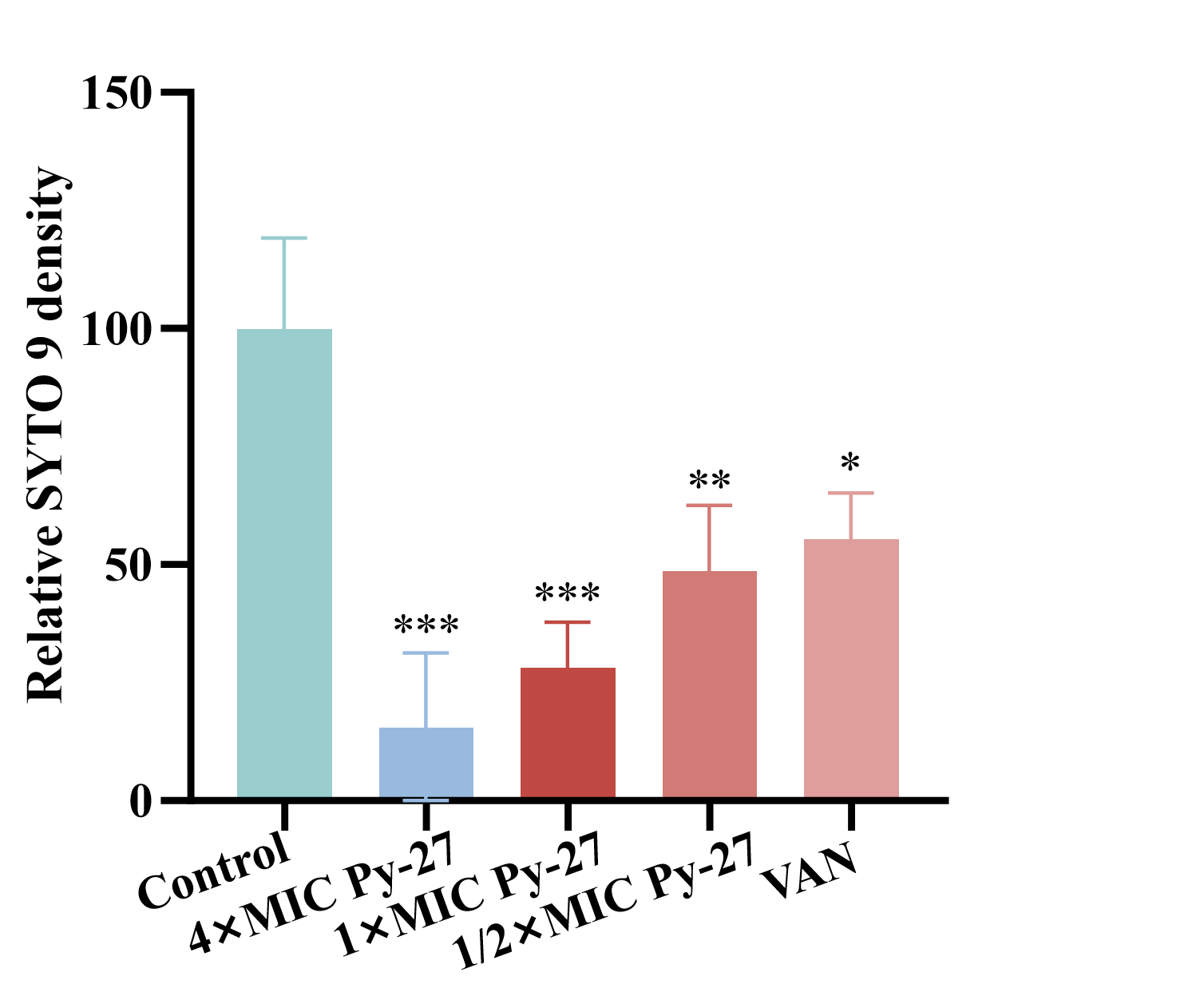
 (B)
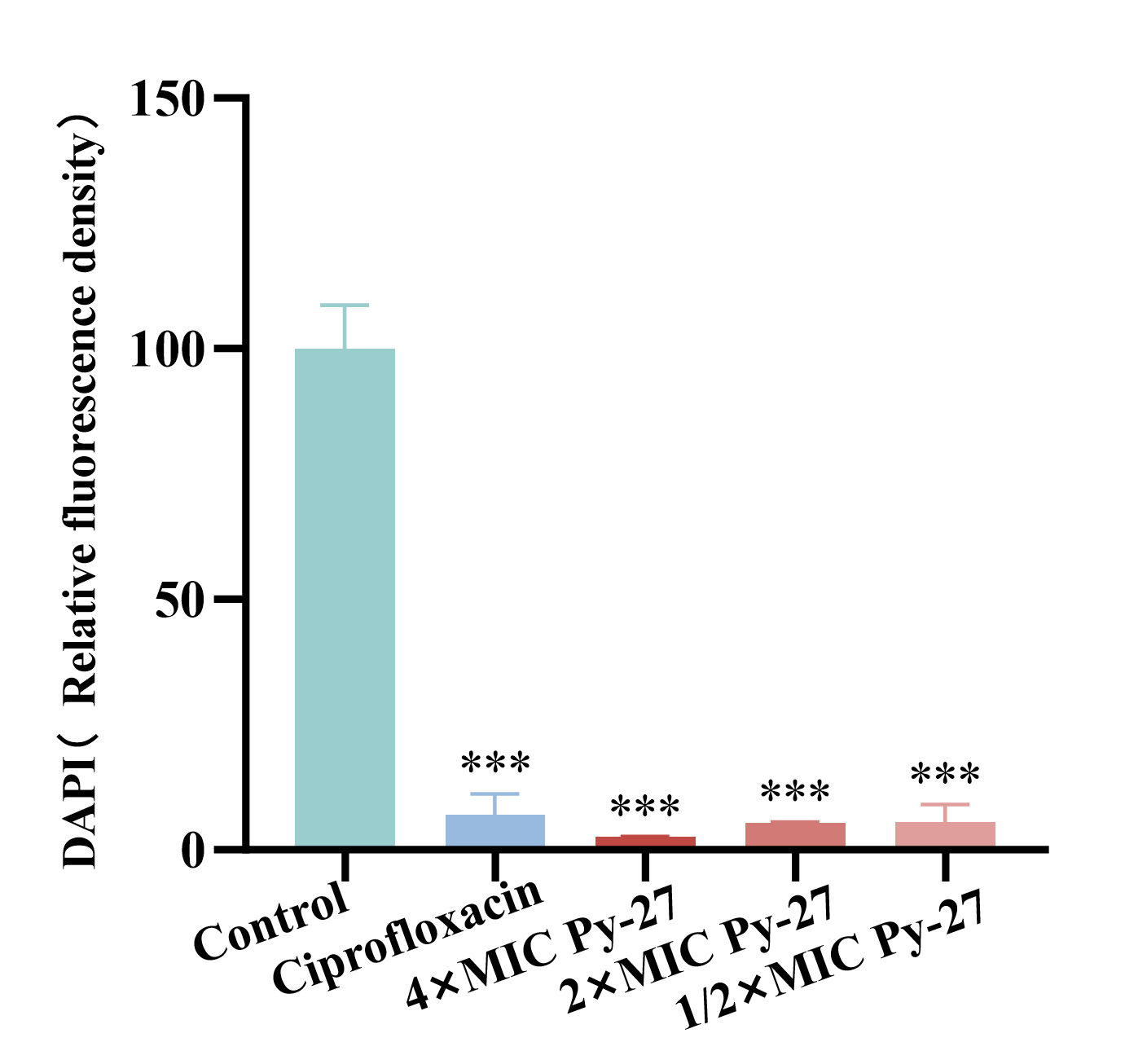
**

**Fig. S13. (A) Statistical analysis of the SYTO9 fluorescence intensity (n=3). (B) Statistical analysis of the DAPI ﬂuorescence intensity (n=3).** Data were presented as mean ± SD. Statistical signiﬁcance was analyzed by ordinary one-way ANOVA(^*^*P* < 0.05,^**^*P* < 0.01,^***^*P* < 0.001).

**Supplementary Tables**

**Table S1. Strains or plasmids used in this study.**

| **Strains or plasmids** | **Description** | **Reference /source** |
| --- | --- | --- |
| *Staphylococcus aureus*  ATCC 43300 | Standard strain | ATCC |
| *Staphylococcus aureus*  ATCC 29213 | Standard strain | ATCC |
| *Enterococcus faecalis*  ATCC 29212 | Standard strain | ATCC |
| *Listeria monocytogenes*  CICC 21529 | Standard strain | CICC |
| *Streptococcus pneumoniae*  ATCC 49619 | Standard strain | ATCC |
| *Escherichia coli*  ATCC 25922 | Standard strain | ATCC |
| *Proteus vulgaris*  ATCC 49132 | Standard strain | ATCC |
| *Pseudomonas aeruginosa*  ATCC 27318 | Standard strain | ATCC |
| *Salmonella typhimurium*  ATCC 14028 | Standard strain | ATCC |
| *Staphylococcus aureus*  RN 4220 | Standard strain  (Restriction-deficient  transformation recipient) | ATCC |
| ATCC 43300 Δ*pyrB* | *ΔpyrB* knockout strain | This study |
| pBT2 | *S. aureus* genome editing vector | Lab stock |
| pBT2-*pyrB* | pBT2 *with upstream and*  *downstream of the pyrB gene* | This study |

**^*^**ATCC, American Type Culture Collection; CICC, China Center of Industrial Culture Collection.

**Table S2. MIC values of a series of pyrazole-amide hybrids.**

| Compound | R_1_ | R_2_ | R_3_ | R_4_ | MIC (μg/mL) | |
| --- | --- | --- | --- | --- | --- | --- |
|  |  |  |  |  | ***S.aureus***  (ATCC 29213) | **MRSA**  (ATCC 43300) |
| Py-1 | Ph | H | H | Ph | ＞64 | ＞64 |
| Py-2 | Ph | H | H | 4-CF_3_-Ph | ＞64 | ＞64 |
| Py-3 | Ph | H | H | 3,5-diCl-Ph | ＞64 | ＞64 |
| Py-4 | Ph | H | H | 2,4-diCl-Ph | ＞64 | ＞64 |
| Py-5 | Ph | H | H | 4-CH_3_-Ph | ＞64 | ＞64 |
| Py-6 | Ph | H | H | 3-Br-Ph | ＞64 | ＞64 |
| Py-7 | Ph | H | H | 4-I-Ph | ＞64 | ＞64 |
| Py-8 | Ph | H | H | 2-CF_3_-Ph | ＞64 | ＞64 |
| Py-9 | Ph | H | H | 3-CF_3_-Ph | ＞64 | ＞64 |
| Py-10 | Ph | *t*-bu | H | 3,5-diCl-Ph | ＞64 | ＞64 |
| Py-11 | Ph | Me | Br | 3,5-diCl-Ph | ＞64 | ＞64 |
| Py-12 | Me | H | H | 3,5-diCl-Ph | ＞64 | ＞64 |
| Py-13 | Me | H | H | 4-CF_3_-Ph | ＞64 | ＞64 |
| Py-14 | Me | Ph | H | 3,5-diCl-Ph | ＞64 | ＞64 |
| Py-15 | Me | *t*-bu | H | 3,5-diCl-Ph | ＞64 | ＞64 |
| Py-16 | *t*-bu | H | CN | 4-CF_3_-Ph | 64 | 64 |
| Py-17 | Ph | H | CN | 4-CF_3_-Ph | ＞64 | ＞64 |
| Py-18 | Ph | H | CN | 3,5-diCl-Ph | 32 | 32 |
| Py-19 | 4-Me-Ph | H | CN | 4-CF_3_-Ph | ＞64 | ＞64 |
| Py-20 | 4-Me-Ph | H | CN | 3,5-diCl-Ph | 16 | 32 |
| Py-21 | 4-Cl-Ph | H | CN | 4-CF_3_-Ph | 32 | 32 |
| Py-22 | 4-Cl-Ph | H | CN | 3,5-diCl-Ph | 8 | 8 |
| Py-23 | 4-F-Ph | CO_2_Et | CN | 3,5-diCl-Ph | 32 | 32 |
| Py-24 | 4-NO_2_-Ph | H | CN | 3,5-diCl-Ph | ＞64 | ＞64 |
| Py-25 | 4-Cl-Ph | CN | H | 3,5-diCl-Ph | 0.5 | 0.5 |
| Py-26 | 2,4,6-triCl-Ph | CN | H | 3,5-diCl-Ph | 0.5 | 0.5 |
| Py-27 | 2,6-diCl-4-CF_3_-Ph | CN | H | 3,5-diCl-Ph | 0.125 | 0.125 |
| Py-28 | 2-F-4-Cl-Ph | CN | H | 3,5-diCl-Ph | 0.25 | 0.25 |
| Py-29 | 2-Cl-5-Me-Ph | CN | H | 3,5-diCl-Ph | 2 | 2 |
| Py-30 | 4-Me-Ph | CN | H | 3,5-diCl-Ph | 0.5 | 0.5 |
| Py-31 | 4-OMe-Ph | CN | H | 3,5-diCl-Ph | 8 | 8 |
| Py-32 | 2,4-diMe-Ph | CN | H | 3,5-diCl-Ph | 2 | 2 |
| Py-33 | 2,6-diCl-4-CF_3_-Ph | CN | H | 4-I-Ph | 0.5 | 1 |
| Py-34 | 2,6-diCl-4-CF_3_-Ph | CN | H | 3,5-diF-Ph | 1 | 1 |
| Py-35 | 2,6-diCl-4-CF_3_-Ph | CN | H | 2,4-diCl-Ph | 1 | 2 |
| Py-36 | 2,6-diCl-4-CF_3_-Ph | CN | H | 4-OCF_3_-Ph | 0.5 | 0.5 |
| Py-37 | 2,6-diCl-4-CF_3_-Ph | CN | H | 2-Cl-4-F-Ph | 4 | 8 |
| Py-38 | 2,6-diCl-4-CF_3_-Ph | CN | H | 4-CF_3_-Ph | 0.25 | 0.25 |
| Py-39 | 2,6-diCl-4-CF_3_-Ph | CN | H | 4-Me-Ph | ＞64 | ＞64 |
| Py-40 | 2,6-diCl-4-CF_3_-Ph | CN | H | 3-CF_3_-Ph | 1 | 0.5 |
| Py-41 | 2,6-diCl-4-CF_3_-Ph | CN | H | 3-Br-Ph | 1 | 1 |
| Py-42 | 2,6-diCl-4-CF_3_-Ph | CN | H | 3,5-diNO_2_-Ph | 4 | 8 |
| Py-43 | 2,6-diCl-4-CF_3_-Ph | CN | H | 3,5-diCF_3_-Ph | 0.5 | 0.5 |
| Py-44 | 2,6-diCl-4-CF_3_-Ph |  | H | 3,5-diCF_3_-Ph | 16 | 16 |
| Py-45 | 2,6-diCl-4-CF_3_-Ph |  | H | 3,5-diCF_3_-Ph | ＞64 | ＞64 |
| **VAN** | - | - | - | - | 2 | 2 |

**^*^**The MIC is the lowest concentration that completely inhibits microbial growth; > means no activity at the tested concentration; VAN, vancomycin.

**Table S3. MIC of Py-27 against MRSA clinical isolate strains (μg/mL).**

| **Strains** | **Py-27** | **Vancomycin** | **Ampicillin** | **Strains** | **Py-27** | **Vancomycin** | **Ampicillin** |
| --- | --- | --- | --- | --- | --- | --- | --- |
| 1# | 0.125 | 4 | ＞32 | 24# | 0.125 | 4 | ＞32 |
| 2# | 0.125 | 2 | ＞32 | 25# | 0.125 | 4 | ＞32 |
| 3# | 0.125 | 4 | ＞32 | 26# | 0.0625 | 4 | ＞32 |
| 4# | 0.125 | 2 | ＞32 | 27# | 0.0625 | 4 | ＞32 |
| 5# | 0.125 | 2 | ＞32 | 28# | 0.125 | 4 | ＞32 |
| 6# | 0.125 | 4 | ＞32 | 29# | 0.125 | 4 | ＞32 |
| 7# | 0.125 | 4 | ＞32 | 30# | 0.125 | 4 | ＞32 |
| 8# | 0.125 | 4 | ＞32 | 31# | 0.125 | 4 | ＞32 |
| 9# | 0.125 | 4 | ＞32 | 32# | 0.125 | 4 | ＞32 |
| 10# | 0.5 | 4 | ＞32 | 33# | 0.125 | 2 | ＞32 |
| 11# | 0.125 | 2 | ＞32 | 34# | 0.125 | 4 | ＞32 |
| 12# | 0.125 | 4 | ＞32 | 35# | 0.5 | 4 | ＞32 |
| 13# | 0.25 | 2 | ＞32 | 36# | 0.25 | 4 | ＞32 |
| 14# | 0.5 | 4 | ＞32 | 37# | 0.125 | 4 | ＞32 |
| 15# | 0.25 | 4 | ＞32 | 38# | 0.125 | 4 | ＞32 |
| 16# | 0.5 | 4 | ＞32 | 39# | 0.5 | 8 | ＞32 |
| 17# | 0.0625 | 2 | ＞32 | 40# | 0.125 | 8 | ＞32 |
| 18# | 0.125 | 4 | ＞32 | 41# | 0.5 | 8 | ＞32 |
| 19# | 0.125 | 8 | ＞32 | 42# | 0.125 | 8 | ＞32 |
| 20# | 0.125 | 8 | ＞32 | 43# | 0.125 | 8 | ＞32 |
| 21# | 0.5 | 4 | ＞32 | 44# | 0.125 | 8 | ＞32 |
| 22# | 0.125 | 4 | ＞32 | 45# | 0.125 | 8 | ＞32 |
| 23# | 0.0625 | 4 | ＞32 |  |  |  |  |

**^*^**Total of 45 methicillin-resistant *Staphylococcus aureus* (MRSA) clinical isolates associated with bovine mastitis were collected from dairy farms in four provinces of China, namely Gansu, Shanghai, Sichuan, and Guizhou, and were provided by the Natural Products Research Team of Lanzhou Institute of Husbandry and Pharmaceutical Sciences, Chinese Academy of Agricultural Sciences. All isolates were tested in triplicate by the CLSI broth microdilution method using 96-well plates.

**Table S4. Ames test results for Py-27.**

| **Groups** | **TA97** | | **TA98** | | **TA102** | |
| --- | --- | --- | --- | --- | --- | --- |
|  | **-S9** | **+S9** | **-S9** | **+S9** | **-S9** | **+S9** |
| Py-27  (5mg/μL) | 28.7 ± 1.5 | 27.0 ± 4.6 | 2.3 ± 2.5 | 3.3 ± 2.5 | 160.3 ± 15.9 | 127.0 ± 7.5 |
| Negative  control | 29.0 ± 2.6 | 27.0 ± 7.22 | 3.6 ± 1.5 | 2.7 ± 2.5 | 123.0 ± 11.0 | 117.7 ± 5.0 |
| Positive  control | 302.7 ± 8.0^***^ | 287.0 ± 9.2^***^ | 21.3 ± 3.5^***^ | 22.3 ± 4.0^***^ | 96.0 ± 6.6^***^ | 207.3 ± 8.6^***^ |

All experiments were performed as three biologically independent experiments. Data were presented as mean ± SD. Statistical signiﬁcance was analyzed by ordinary one-way ANOVA (^***^*P* < 0.001).

**Table S5. Sperm abnormality test for Py-27 in mice.**

| **Groups** | | Py-27 (0.43g/kg) | Negative control | Positive control |
| --- | --- | --- | --- | --- |
| Animals | | 5 | 5 | 5 |
| Sperm count | | 5×1000 | 5×1000 | 5×1000 |
| Abnormal sperm count | | 129 | 132 | 692 |
| Sperm abnormality rate (%) | | 2.58 | 2.64 | 13.84^***^ |
| Percentage of deformed sperm species (%) | No hook | 23.26 | 21.97 | 24.57 |
|  | Banana-shaped | 5.43 | 4.55 | 5.78 |
|  | Amorphism | 23.26 | 25.00 | 24.71 |
|  | Fat head | 16.28 | 17.42 | 15.46 |
|  | Folded tail | 11.63 | 12.12 | 9.83 |
|  | Double heads | 12.40 | 10.61 | 10.26 |
|  | Double tails | 7.75 | 8.33 | 9.39 |
|  | Other | 0 | 0 | 0 |

All experiments were performed as five biologically independent experiments. Statistical signiﬁcance was analyzed by ordinary one-way ANOVA (^***^*P* < 0.001).

**Table S6. Results of *in vitro* mammalian erythrocyte micronucleus test for Py-27.**

| **Groups** | Number of mice examined | **Micronucleus analysis** | | **PCE analysis** | |
| --- | --- | --- | --- | --- | --- |
|  |  | Number of PCE examined per animal | MNPCE rate (‰) | Number of RBC examined per animal | PCE/RBC (%) |
| Py-27  (0.43g/kg) | 10 | 1000 | 4.000 | 996 | 99.6 |
| Negative control  (--) | 10 | 1000 | 4.333 | 897 | 89.7 |
| Positive Control  (40mg/kg) | 10 | 1000 | 29.333^*^ | 962 | 96.2 |

Negative control: soybean oil; Positive control: cyclophosphamide; MNPCE: micronucleated polychromatic erythrocytes; RBC: Red blood cells; PCE: polychromatic erythrocytes; All experiments were performed as ten biologically independent experiments. Statistical signiﬁcance was analyzed by ordinary one-way ANOVA (^*^*P* < 0.05)

**Table S7. Primers used in this study.**

| **Primer** | **Oligonucleotide (5′-3′)** | **Application** |
| --- | --- | --- |
| RT- *rplR*-F | AGACAGCGACATTGCTACTAC | qRT-PCR |
| RT- *rplR*-R | ATATCCTCCACGGTCAAATACG | qRT-PCR |
| RT-*rpsB*-F | CGATGCTATCCGTGCAGTTA | qRT-PCR |
| RT-*rpsB*-R | TGTTCTGCAGCTACTTCTTCAT | qRT-PCR |
| RT-*adh*-F | CAACAGGTCGTGAAACACTTTG | qRT-PCR |
| RT-*adh*-R | CACAGCATAGTCTGCAGTAACA | qRT-PCR |
| RT-*pfkA*-F | CAAGTCACGCACGAACATTTAT | qRT-PCR |
| RT-*pfkA*-R | TGTCTCAGCACCAACTGATAAT | qRT-PCR |
| RT-*fabZ*-F | AGTATCAGGAAACGAACCATTCT | qRT-PCR |
| RT-*fabZ*-R | ATAGCTACCGCACCTGTTTG | qRT-PCR |
| RT-*tdcB*-F | CAGGCGGGAATGTTGACTTA | qRT-PCR |
| RT-*tdcB*-R | ACCACACCCTTGCTTGTATC | qRT-PCR |
| RT-*pyrB*-F | AGACATGGGCTTGCAGAAGA | qRT-PCR |
| RT-*pyrB*-R | CCTCTATTCACAGGTGCCGG | qRT-PCR |
| RT-*deoC*-F | CATCAGGTGGCGTACGTAAT | qRT-PCR |
| RT-*deoC*-R | GAATCTGCTTCTAAACCTTGCATAA | qRT-PCR |
| RT-*isaB*-F | AGCTTATGGCAAGGACTTGAA | qRT-PCR |
| RT-*isaB*-R | ACGACAAACCAGATGCCTAAT | qRT-PCR |
| RT-*fabI*-F | AAGGTGTGGGTGGTTTCAATA | qRT-PCR |
| RT-*fabI*-R | CACCTGTAACGCCACTTGATA | qRT-PCR |
| RT-*16S rRNA*-F | CGCAATGGGCGAAAGC | qRT-PCR |
| RT-*16S rRNA*-R | TACGATCCGAAGACCTTCATCA | qRT-PCR |

**Table S8. Preliminary Pharmacokinetic Data for Py-27.**

Dissolve Py-27 in DMSO, then add water to prepare a suspension， and orally administered to male and female Sprague-Dawley rats (200-250 g, n = 3 per sex) at a dosage of 200 mg/kg following overnight fasting. Blood samples were collected via the jugular vein into EDTA-K₂ tubes at predetermined time points and centrifuged at 3200×g for 5 min at 4 ℃. Plasma concentrations of Py-27 were quantified using a validated LC-MS/MS method. Briefly, 50 μL of plasma was mixed with 450 μL of acetonitrile containing tolbutamide as the internal standard. After vortexing and centrifugation, 200 μL of the supernatant was transferred to a 96-well plate. Pharmacokinetic parameters, including C_max_, T_max_, AUC_0-t_, AUC_0-∞_, t_1/2_, MRT, and CL, were calculated using noncompartmental analysis with Phoenix WinNonlin 8.3 (Certara).

| 200 mg/kg Py-27 | | Py-27 | | | | | | | | | |
| --- | --- | --- | --- | --- | --- | --- | --- | --- | --- | --- | --- |
|  |  | **(h)** | | | | | | | | | |
|  |  | **0** | **0.25** | **0.5** | **1** | **2** | **4** | **8** | **10** | **24** | **48** |
| **Sex** | **Subject** | **Concentration** | | | | | | | | | |
|  |  | **(ng/mL)** | | | | | | | | | |
| M | Gp3M13 | BLQ | 23.0 | 130 | 404 | 1190 | 2040 | 3620 | 3700 | 7730 | 1820 |
| M | Gp3M14 | BLQ | 17.2 | 97.6 | 349 | 978 | 2370 | 3980 | 4190 | 5340 | 2030 |
| M | Gp3M15 | BLQ | 8.83 | 67.5 | 282 | 882 | 2130 | 3890 | 4060 | 6300 | 1960 |
| M | Mean | 0.00 | 16.3 | 98.4 | 345 | 1020 | 2180 | 3830 | 3980 | 6460 | 1940 |
| M | SD | 0.00 | 7.12 | 31.3 | 61.1 | 158 | 171 | 187 | 254 | 1200 | 107 |
| F | Gp3F16 | BLQ | 9.76 | 72.7 | 256 | 647 | 1440 | 2170 | 2660 | 4010 | 4330 |
| F | Gp3F17 | BLQ | 21.2 | 82.2 | 332 | 884 | 2160 | 3530 | 4150 | 4930 | 6850 |
| F | Gp3F18 | BLQ | 22.5 | 114 | 347 | 908 | 1880 | 3160 | 3510 | 5320 | 7790 |
| F | Mean | 0.00 | 17.8 | 89.6 | 312 | 813 | 1830 | 2950 | 3440 | 4750 | 6320 |
| F | SD | 0.00 | 7.01 | 21.6 | 48.8 | 144 | 363 | 703 | 747 | 673 | 1790 |
|  | N | 6 | 6 | 6 | 6 | 6 | 6 | 6 | 6 | 6 | 6 |
|  | Mean | 0.00 | 17.1 | 94.0 | 328 | 915 | 2000 | 3390 | 3710 | 5610 | 4130 |
|  | SD | 0.00 | 6.37 | 24.5 | 52.7 | 175 | 319 | 665 | 581 | 1280 | 2660 |
|  | CV% | NC | 37.3 | 26.1 | 16.1 | 19.2 | 15.9 | 19.6 | 15.7 | 22.8 | 64.3 |
|  | Median | 0.00 | 19.2 | 89.9 | 340 | 896 | 2090 | 3580 | 3880 | 5330 | 3180 |
|  | Q1 | 0.00 | 9.53 | 71.4 | 276 | 823 | 1770 | 2910 | 3300 | 4700 | 1930 |
|  | Q3 | 0.00 | 22.6 | 118 | 363 | 1030 | 2210 | 3910 | 4160 | 6660 | 7090 |
|  | Min | 0.00 | 8.83 | 67.5 | 256 | 647 | 1440 | 2170 | 2660 | 4010 | 1820 |
|  | Max | 0.00 | 23.0 | 130 | 404 | 1190 | 2370 | 3980 | 4190 | 7730 | 7790 |
|  | Geometric Mean | NC | 15.9 | 91.4 | 325 | 900 | 1980 | 3330 | 3670 | 5490 | 3450 |
|  | Geometric CV% | NC | 45.2 | 26.2 | 16.5 | 19.9 | 17.5 | 22.8 | 17.4 | 22.6 | 74.2 |

| **Sex** | **Subject** | **T_max_** | **C_max_** | **AUC_0-t_** | **AUC_0-∞_** | **t_1/2_** | **MRT_0-t_** | **λz** | **Rsq** | **N_first** | **N_last** |
| --- | --- | --- | --- | --- | --- | --- | --- | --- | --- | --- | --- |
|  |  | **h** | **ng/mL** | **h*ng/mL** | **h*ng/mL** | **h** | **h** | **1/h** |  | **h** | **h** |
| M | Gp3M13 | 24.0 | 7730 | 217000 | NA | NA | 22.9 | NA | NA | NA | NA |
| M | Gp3M14 | 24.0 | 5340 | 180000 | NA | NA | 22.6 | NA | NA | NA | NA |
| M | Gp3M15 | 24.0 | 6300 | 195000 | NA | NA | 22.8 | NA | NA | NA | NA |
| M | Mean | 24.0 | 6460 | 198000 | NC | NC | 22.8 | NC | NC | NC | NC |
| M | SD | 0.00 | 1200 | 18700 | NC | NC | 0.176 | NC | NC | NC | NC |
| F | Gp3F16 | 48.0 | 4330 | 161000 | NA | NA | 28.5 | NA | NA | NA | NA |
| F | Gp3F17 | 48.0 | 6850 | 228000 | NA | NA | 29.2 | NA | NA | NA | NA |
| F | Gp3F18 | 48.0 | 7790 | 239000 | NA | NA | 30.5 | NA | NA | NA | NA |
| F | Mean | 48.0 | 6320 | 210000 | NC | NC | 29.4 | NC | NC | NC | NC |
| F | SD | 0.00 | 1790 | 42100 | NC | NC | 0.988 | NC | NC | NC | NC |
|  | N | 6 | 6 | 6 | 0 | 0 | 6 | 0 | 0 | 0 | 0 |
|  | Mean | 36.0 | 6390 | 204000 | NC | NC | 26.1 | NC | NC | NC | NC |
|  | SD | 13.1 | 1370 | 29800 | NC | NC | 3.69 | NC | NC | NC | NC |
|  | CV% | 36.5 | 21.4 | 14.7 | NC | NC | 14.1 | NC | NC | NC | NC |
|  | Median | 36.0 | 6580 | 206000 | NC | NC | 25.7 | NC | NC | NC | NC |
|  | Q1 | 24.0 | 5090 | 175000 | NC | NC | 22.7 | NC | NC | NC | NC |
|  | Q3 | 48.0 | 7750 | 231000 | NC | NC | 29.5 | NC | NC | NC | NC |
|  | Min | 24.0 | 4330 | 161000 | NC | NC | 22.6 | NC | NC | NC | NC |
|  | Max | 48.0 | 7790 | 239000 | NC | NC | 30.5 | NC | NC | NC | NC |
|  | Geometric Mean | 33.9 | 6260 | 202000 | NC | NC | 25.9 | NC | NC | NC | NC |
|  | Geometric CV% | 39.4 | 23.2 | 15.1 | NC | NC | 14.2 | NC | NC | NC | NC |


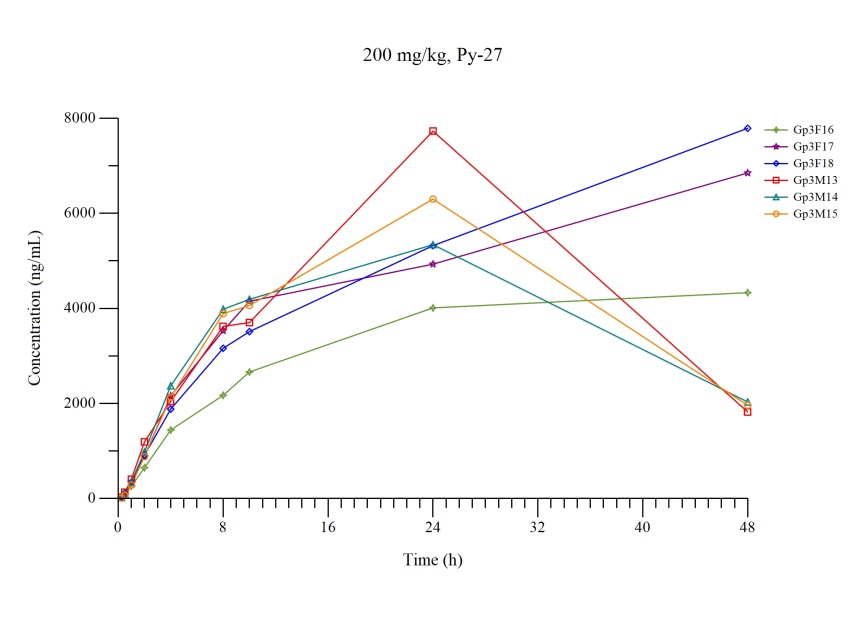


Preliminary conclusion from the pilot study: Py-27 exhibits good oral bioavailability.

**Supplementary** Data S1**: NMR spectra**

^1^H NMR Spectra of **Py-1**


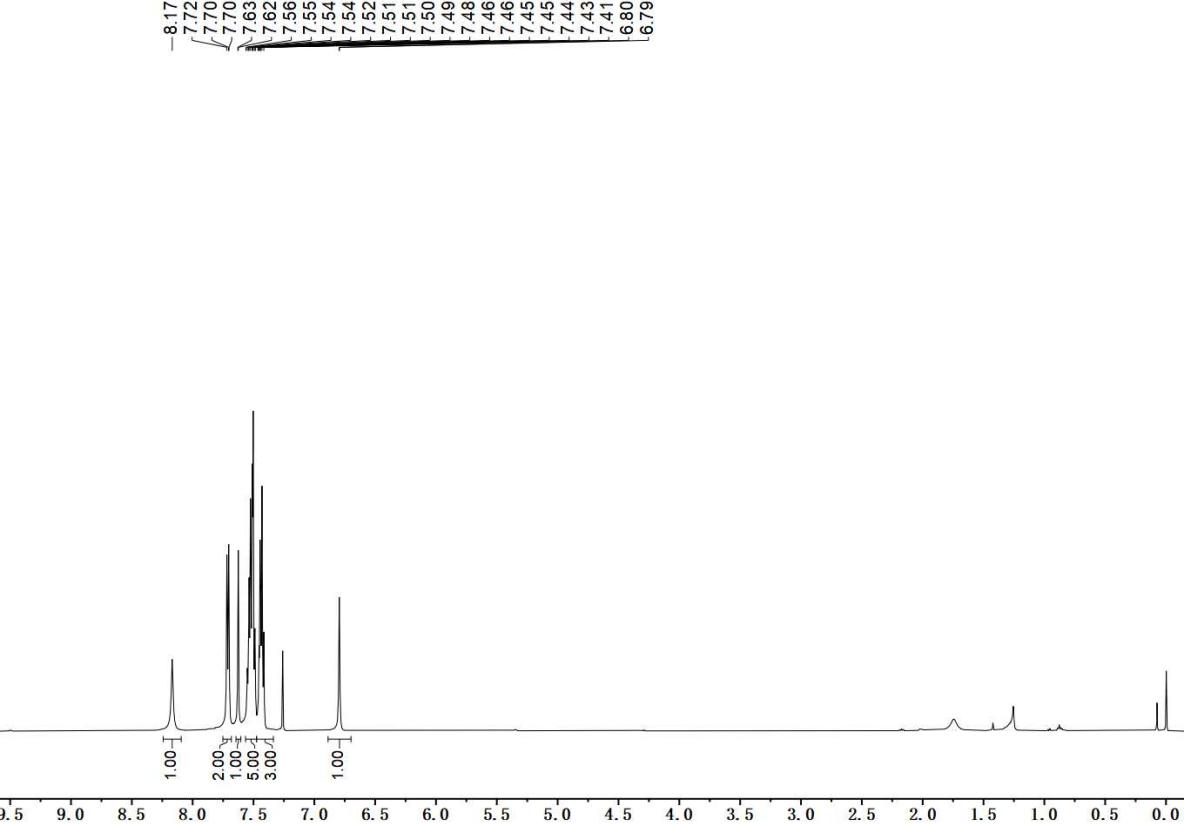


^13^C NMR Spectra of **Py-1**


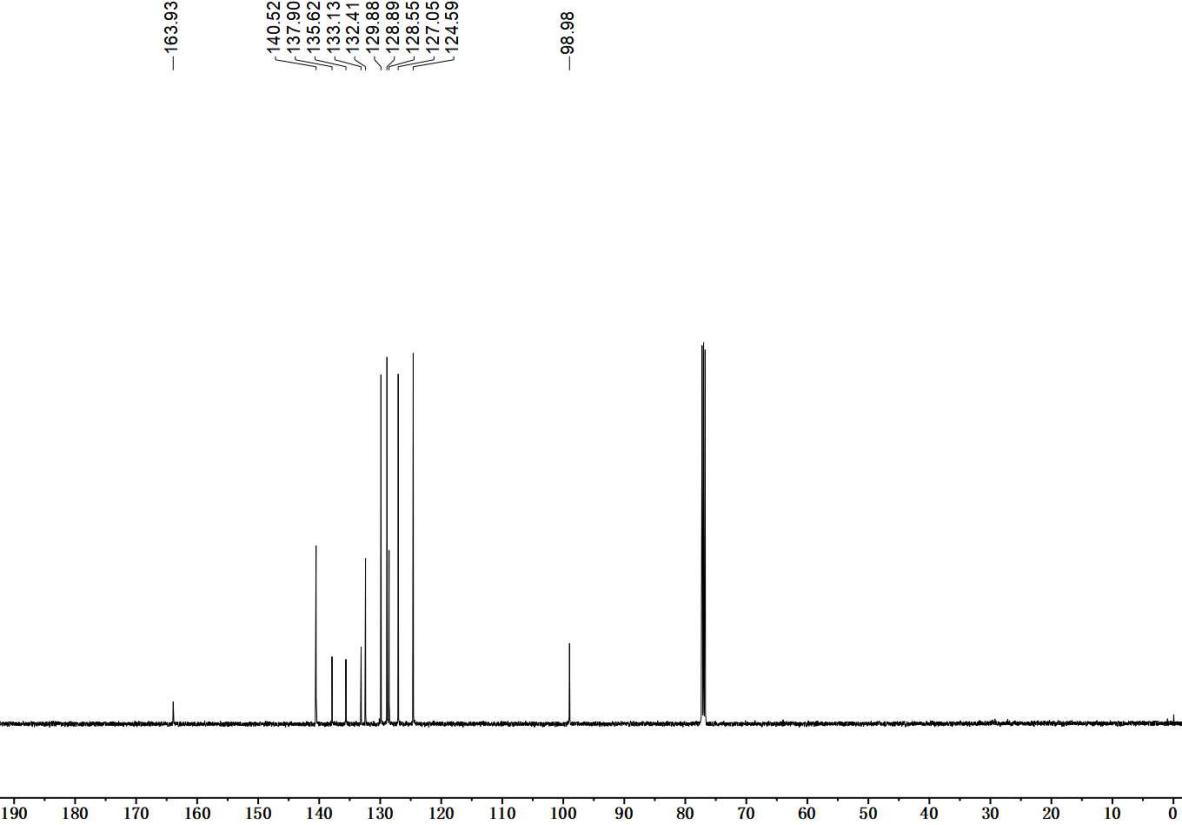


^1^H NMR Spectra of **Py-2**


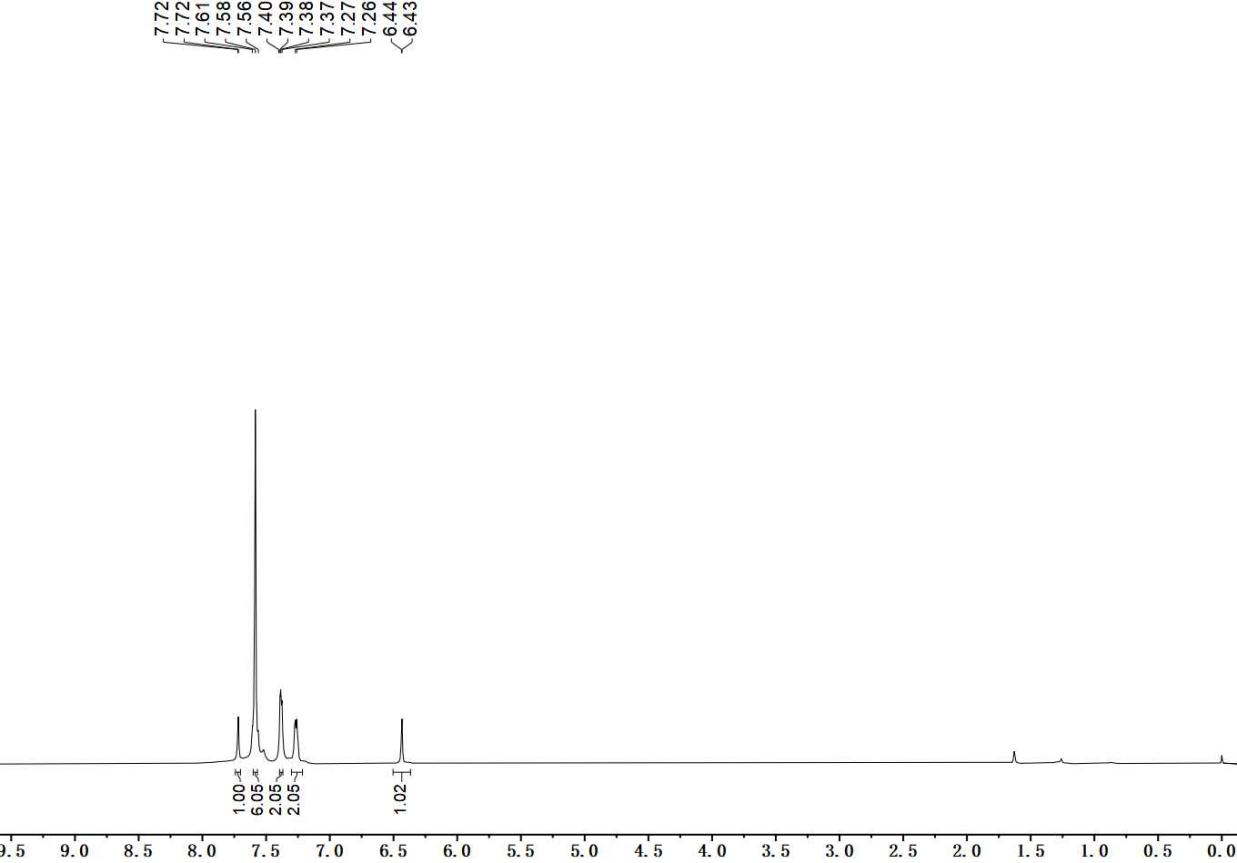


^13^C NMR Spectra of **Py-2**


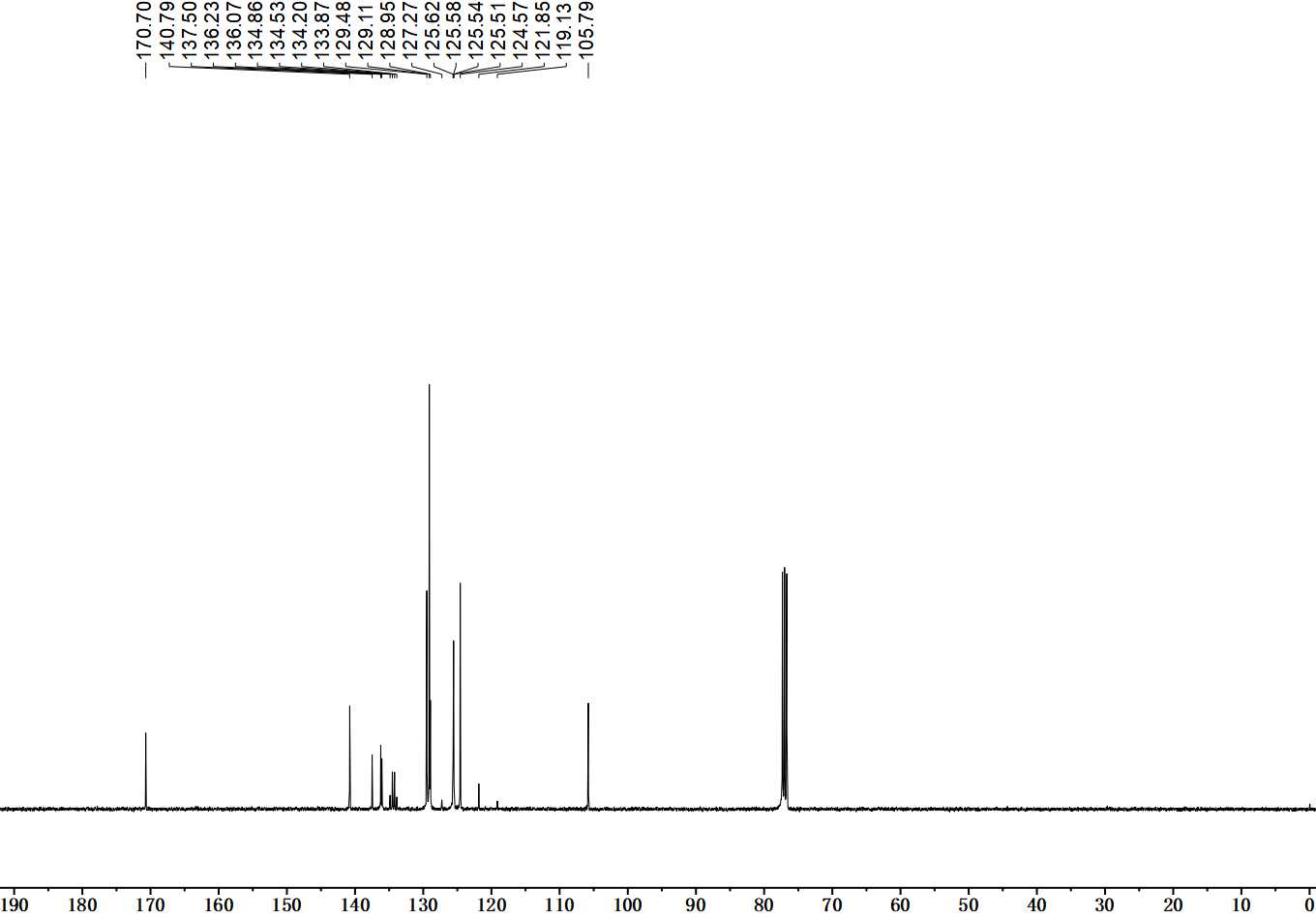
^1^H NMR Spectra of **Py-3**

**
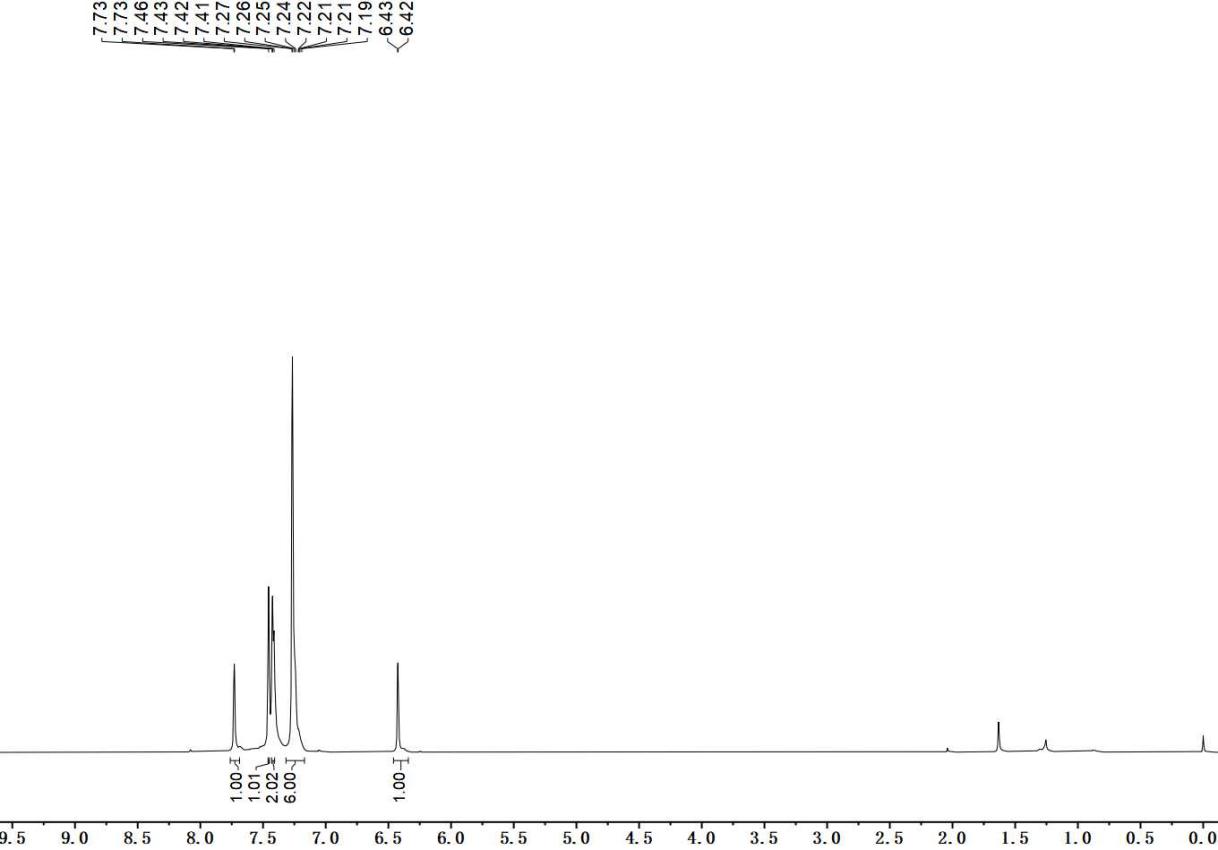
**

^13^C NMR Spectra of **Py-3**

**
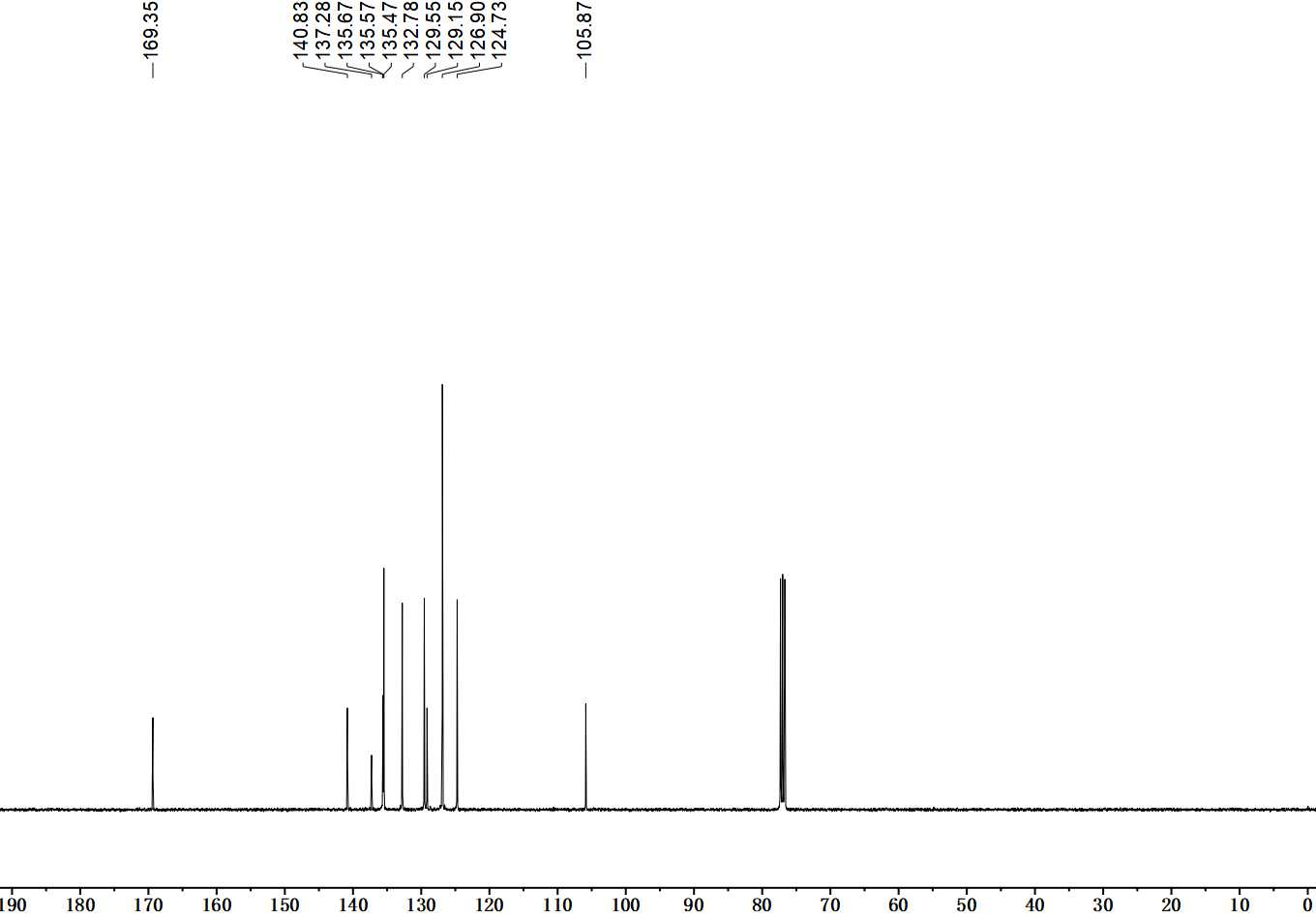
**

^1^H NMR Spectra of **Py-4**

**
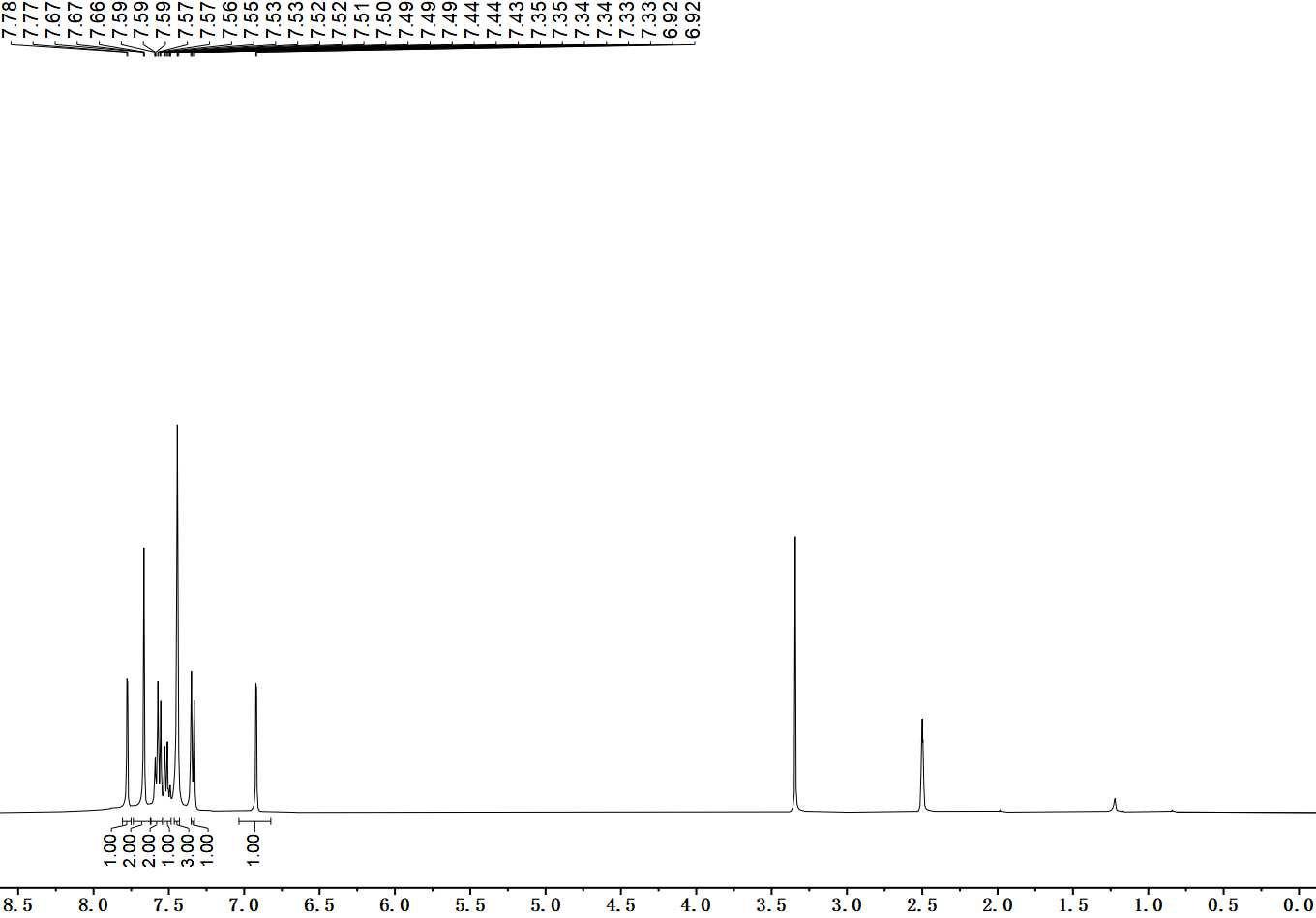
**

^13^C NMR Spectra of **Py-4**

**
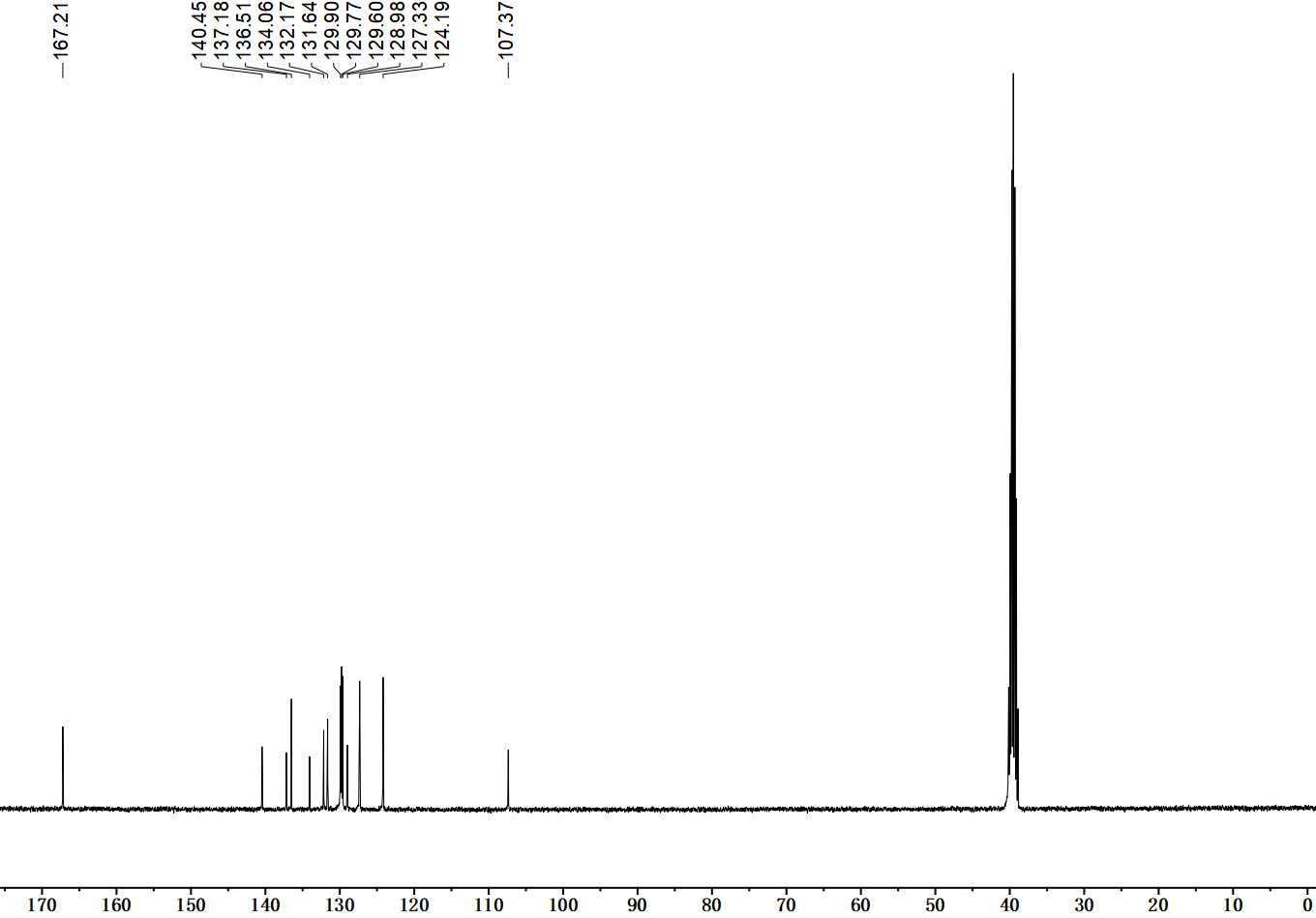
**

^1^H NMR Spectra of **Py-5**

**
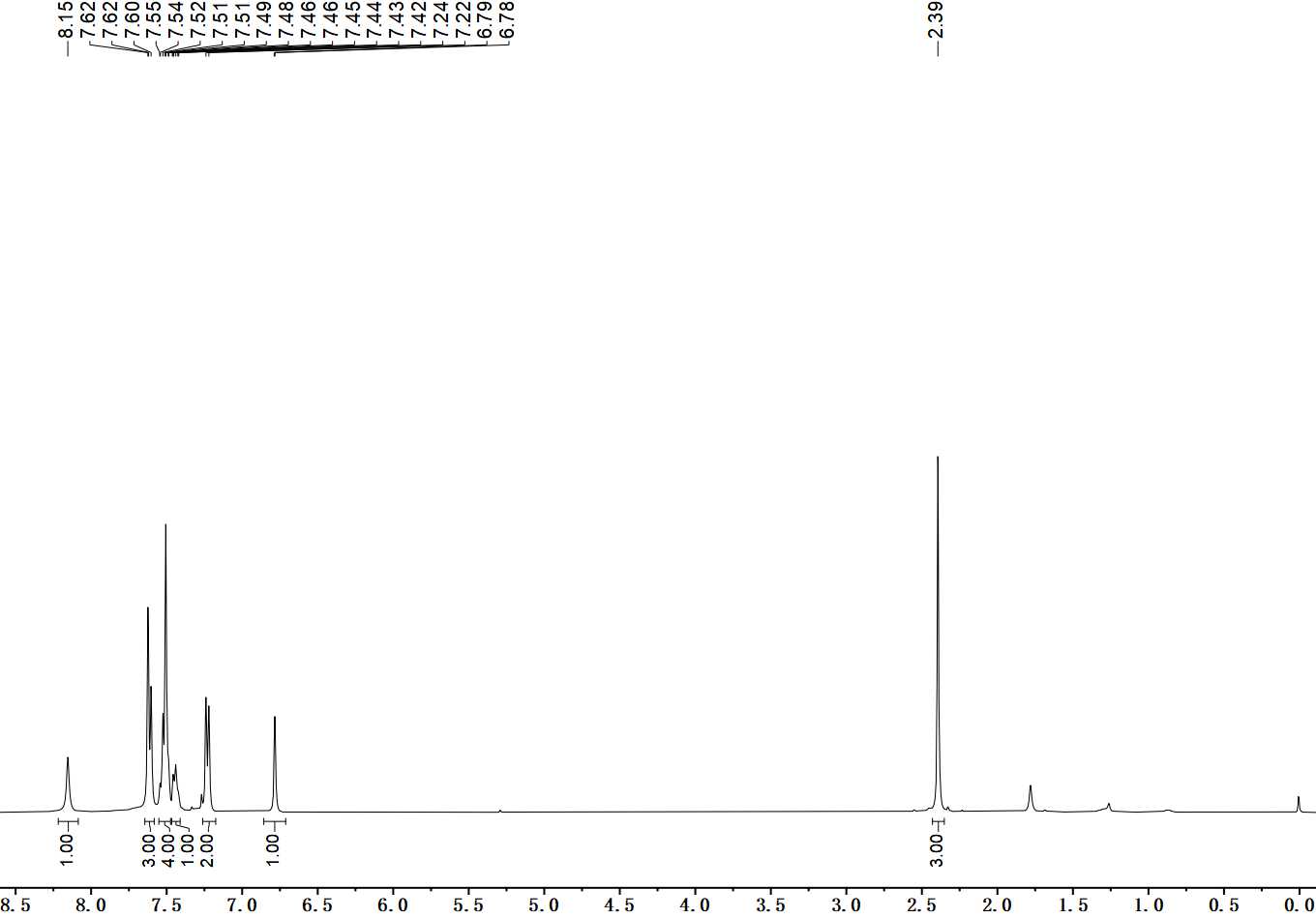
**

^13^C NMR Spectra of **Py-5**

**
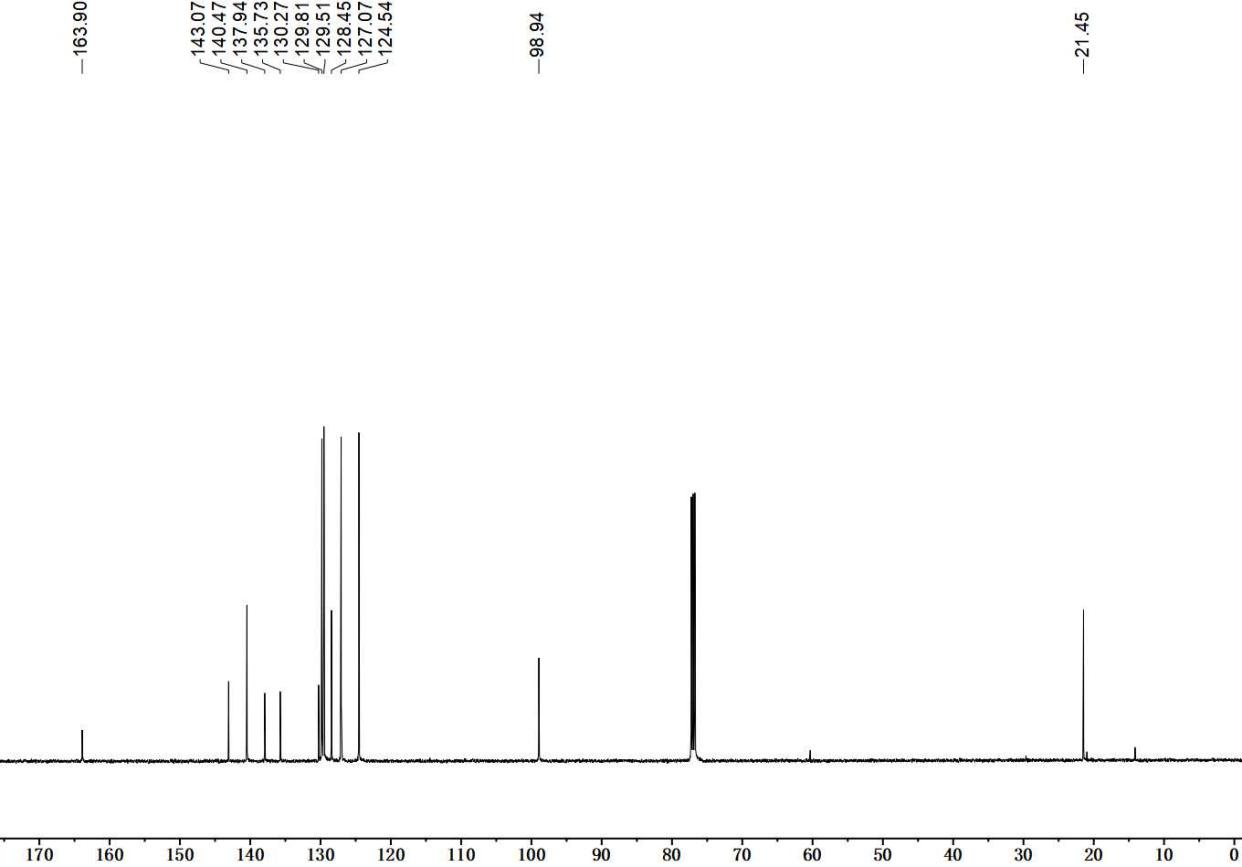
**

^1^H NMR Spectra of **Py-6**

**
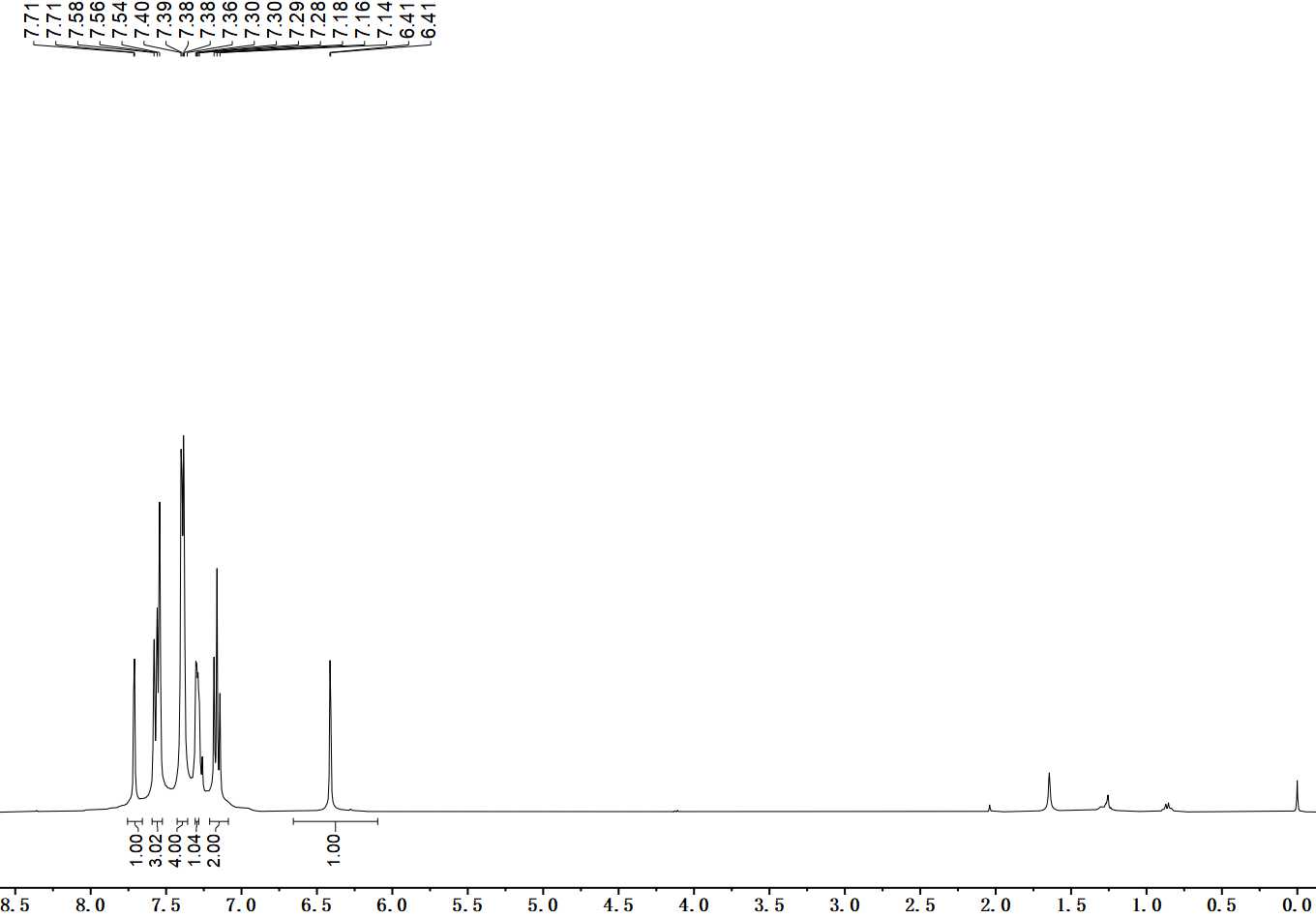
**

^13^C NMR Spectra of **Py-6**

**
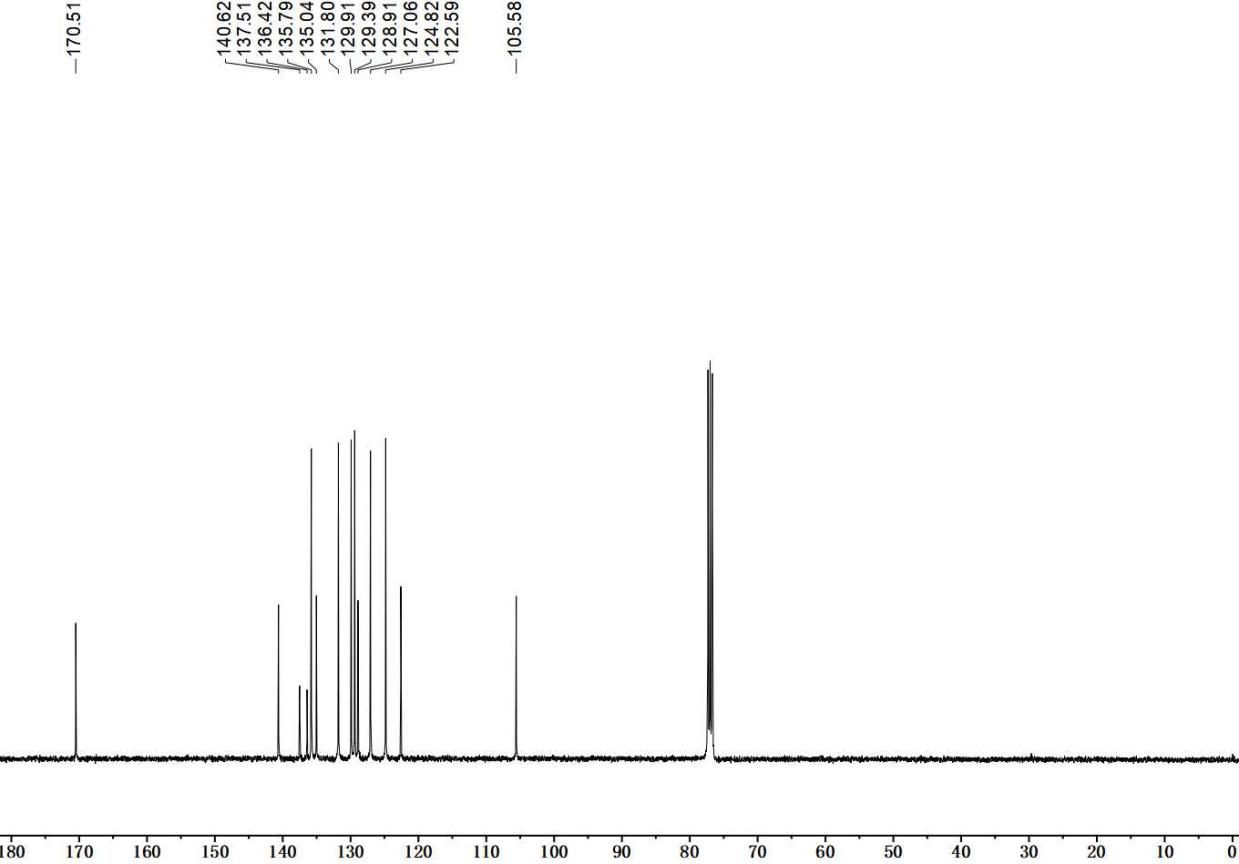
**

^1^H NMR Spectra of **Py-7**

**
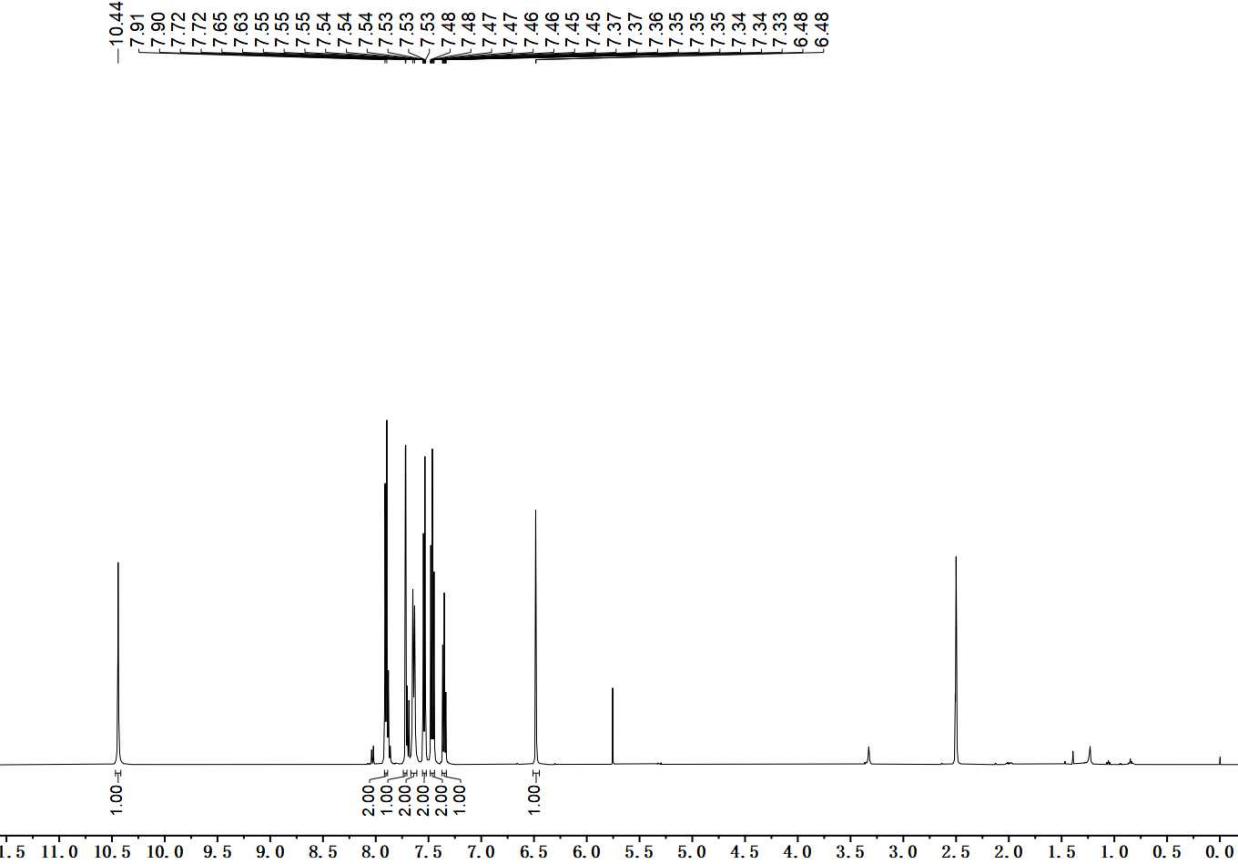
**

^13^C NMR Spectra of **Py-7**

**
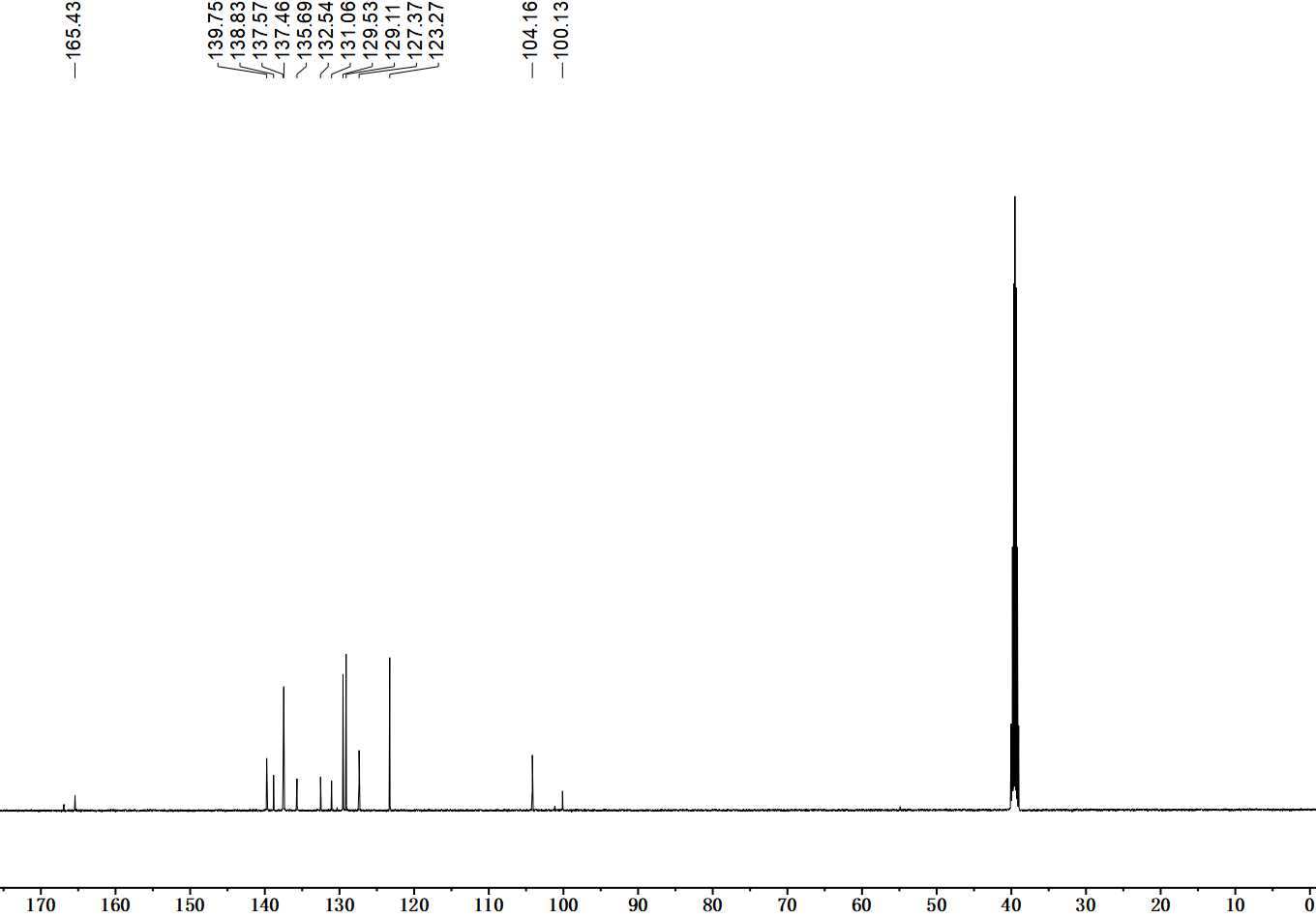
**

^1^H NMR Spectra of **Py-8**

**
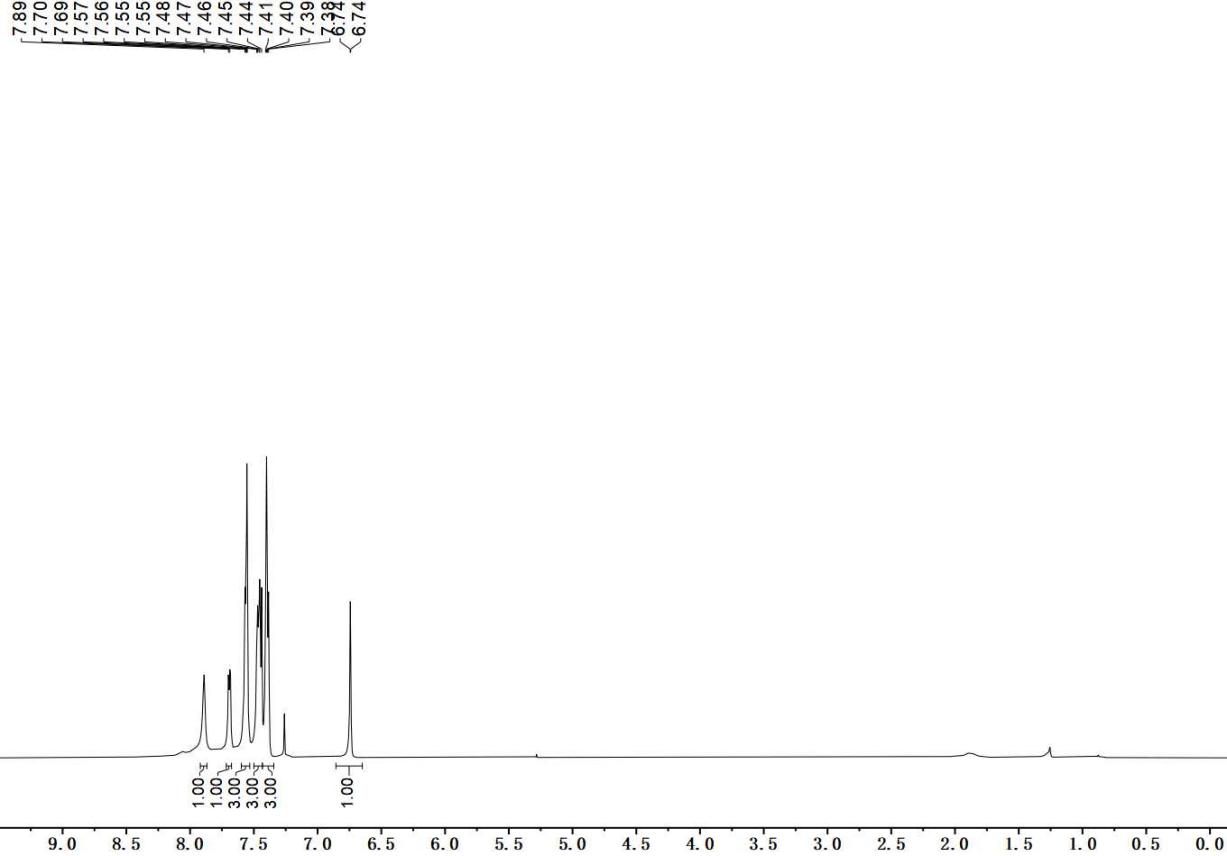
**

^13^C NMR Spectra of **Py-8**

**
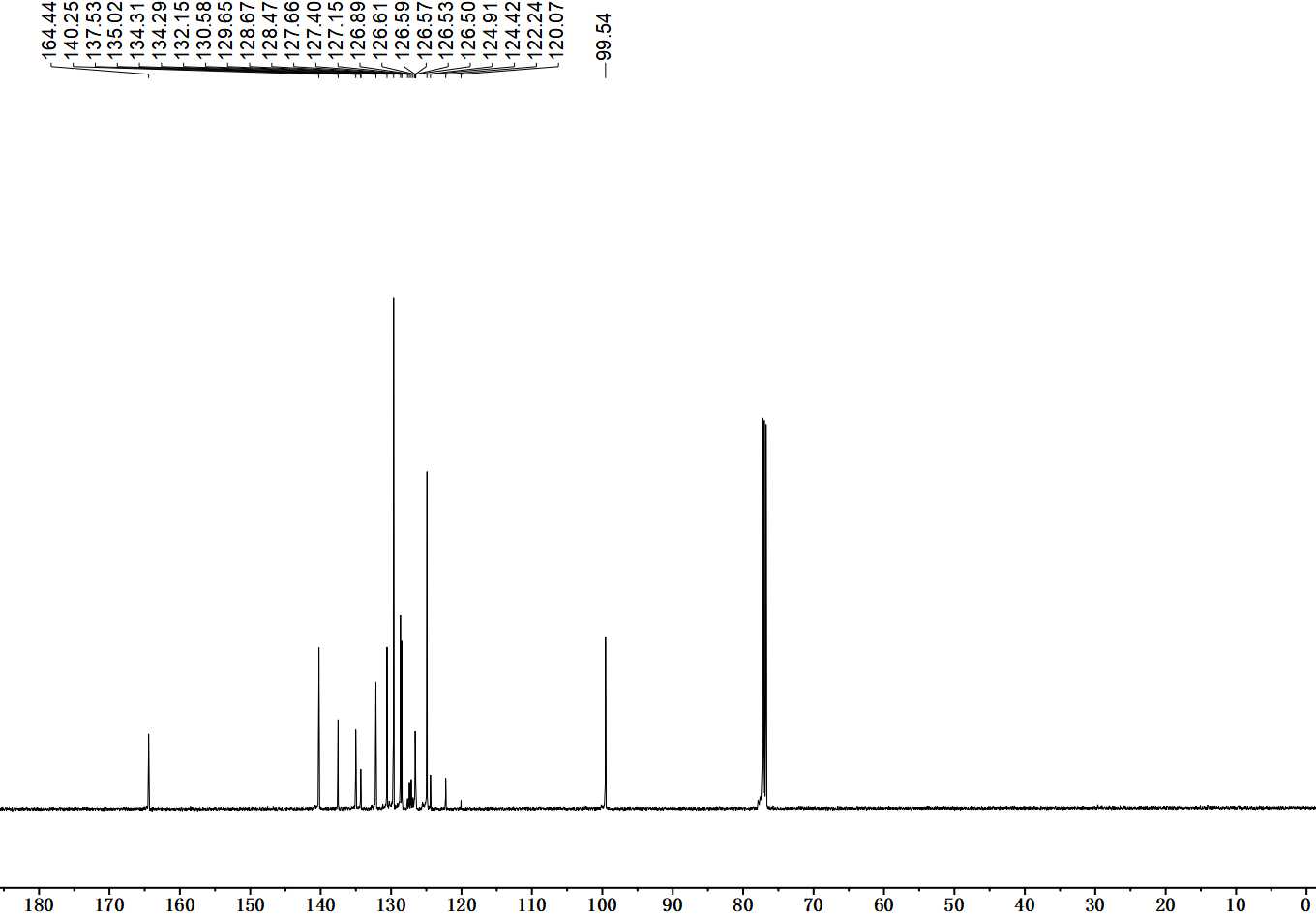
**

^1^H NMR Spectra of **Py-9**

**
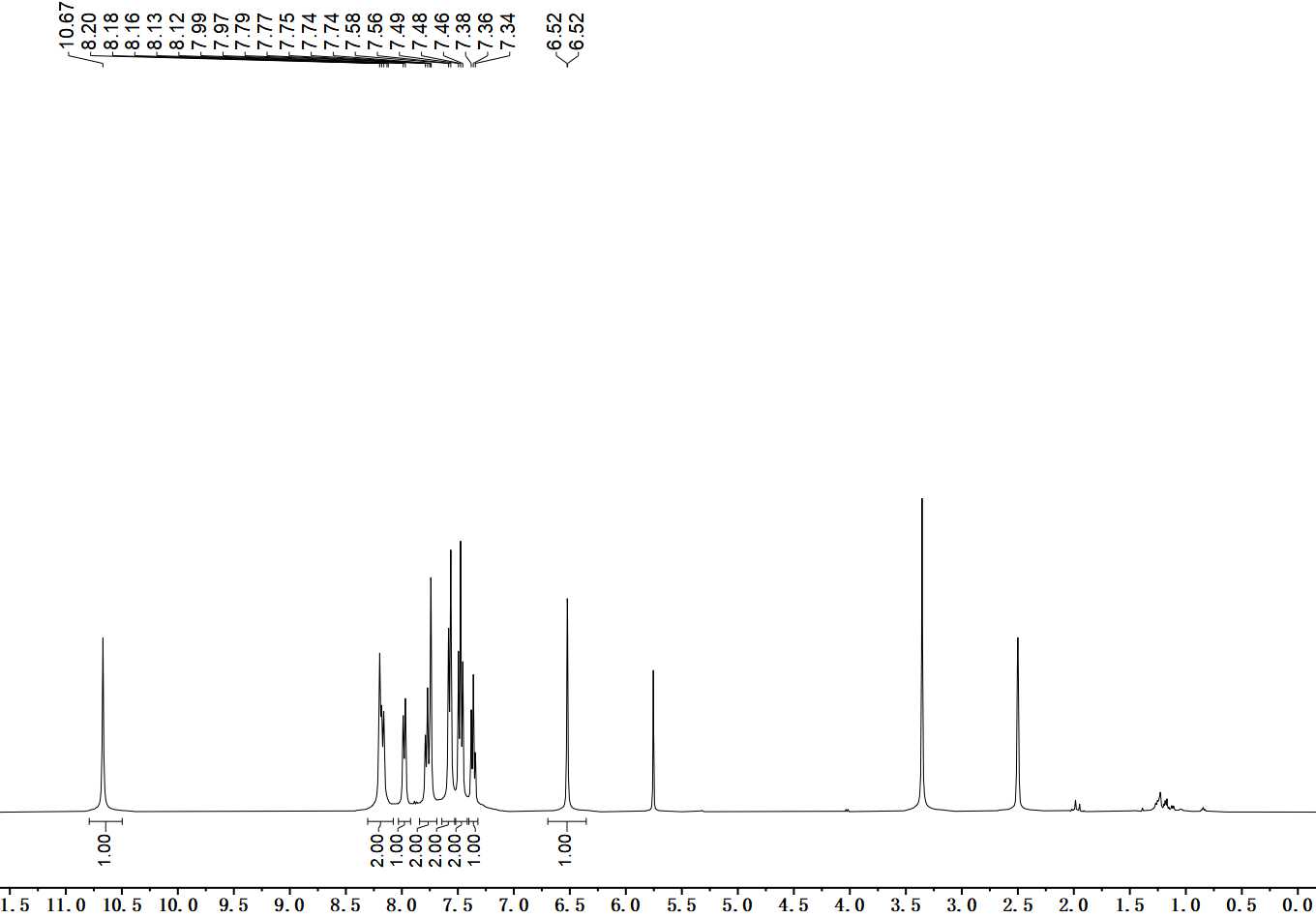
**

^13^C NMR Spectra of **Py-9**

**
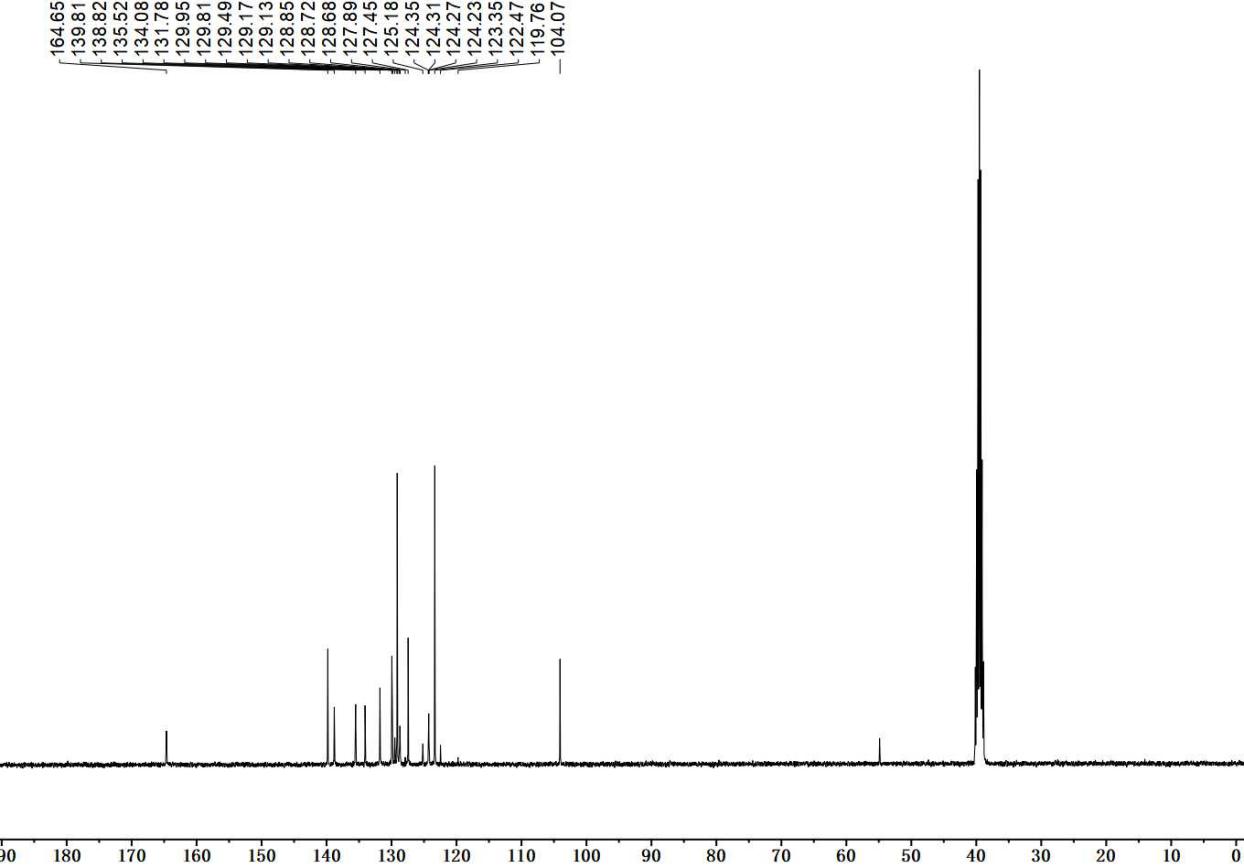
**

^1^H NMR Spectra of **Py-10**

**
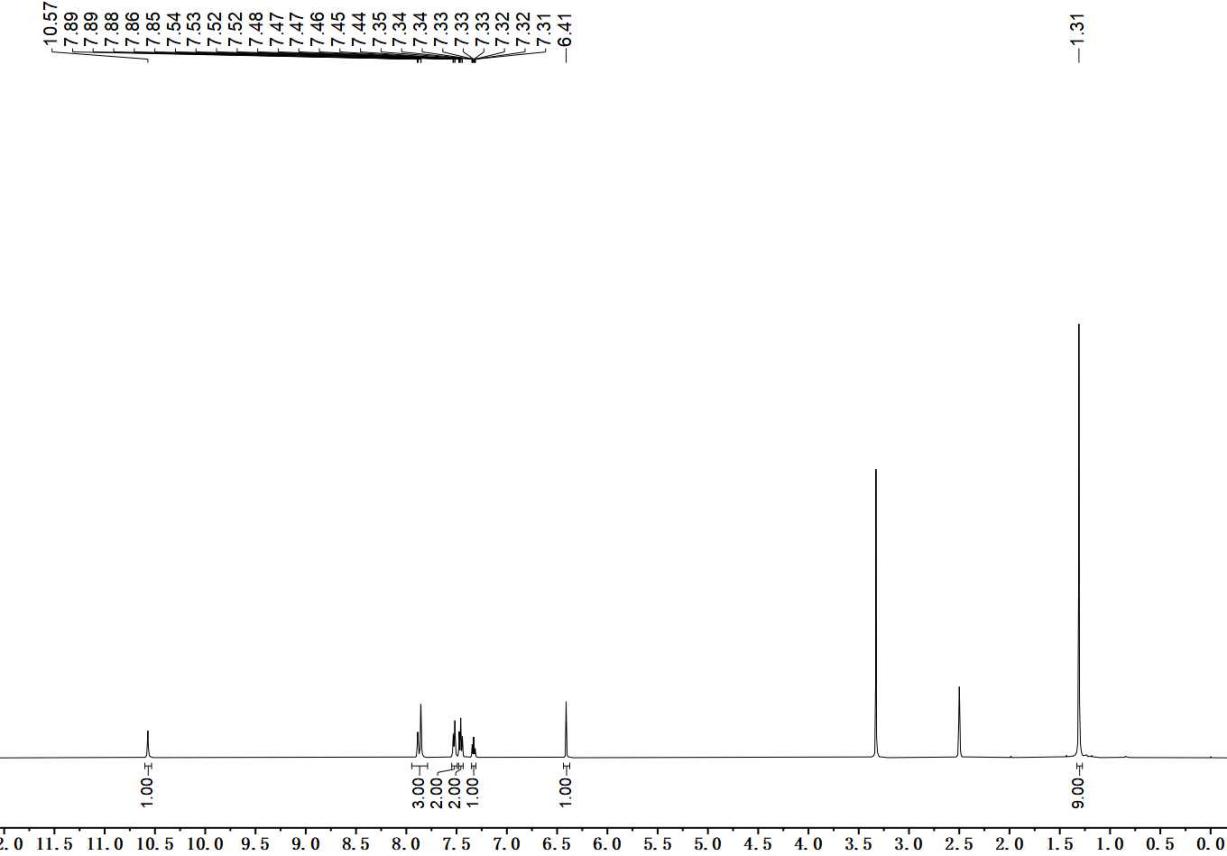
**

^13^C NMR Spectra of **Py-10**

**
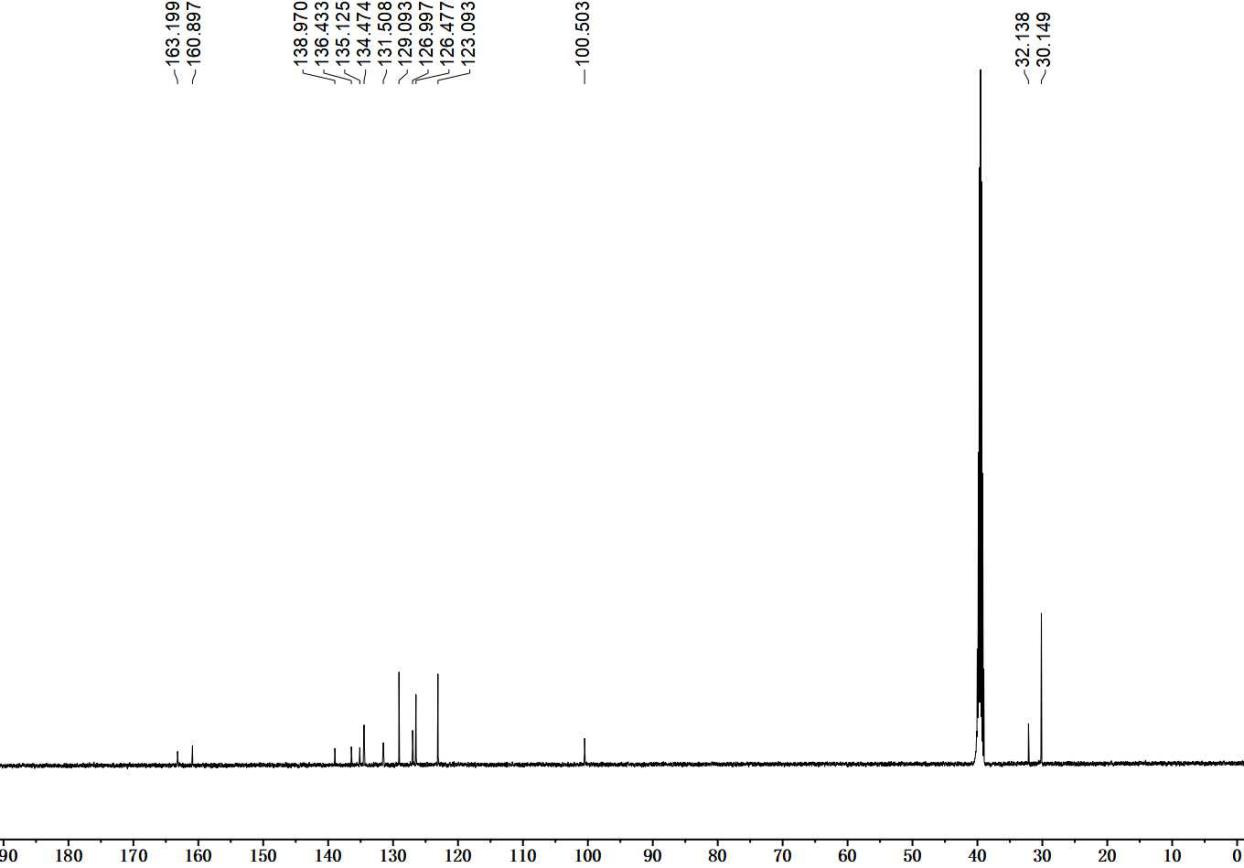
**

^1^H NMR Spectra of **Py-11**

**
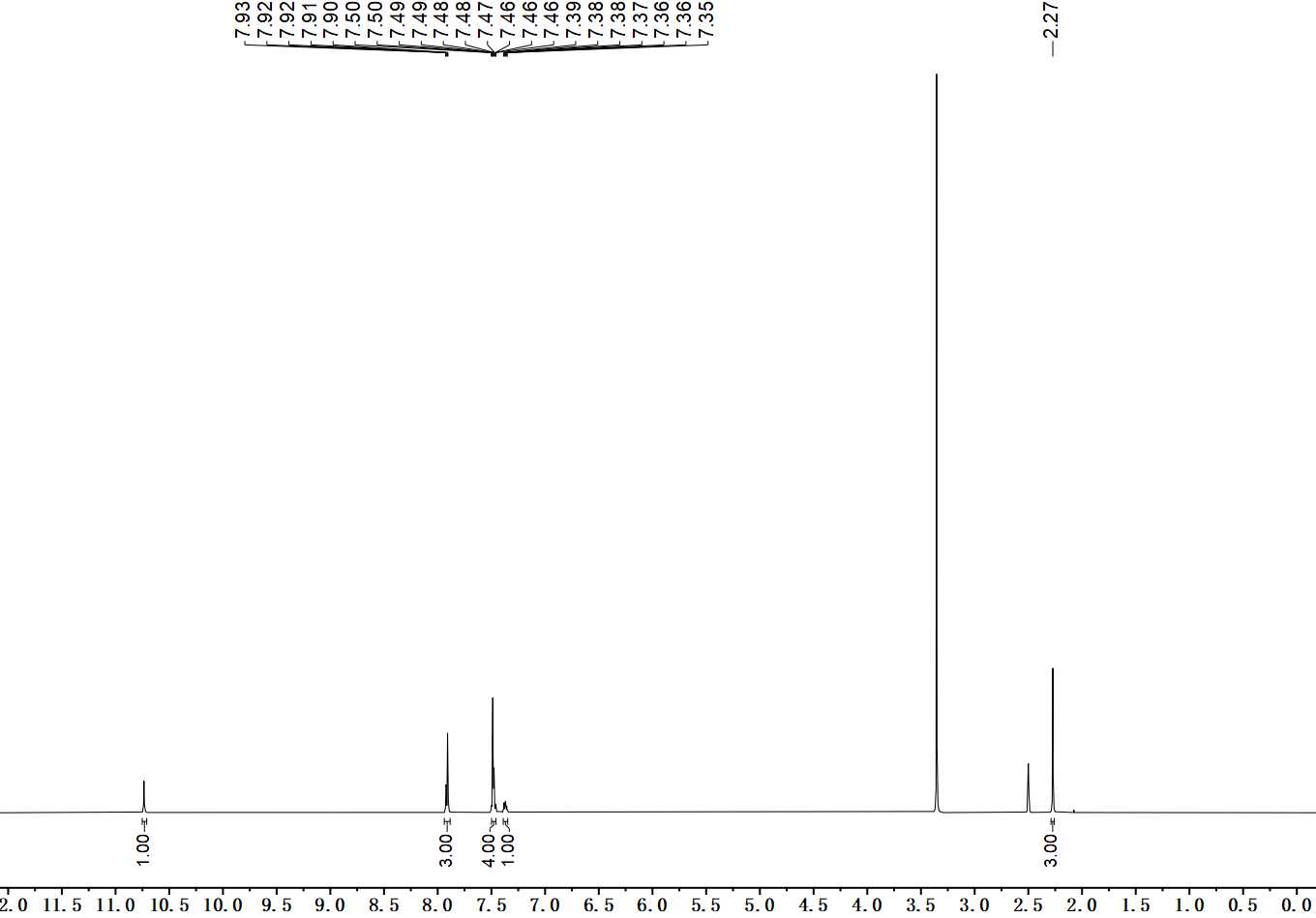
**

^13^C NMR Spectra of **Py-11**

**
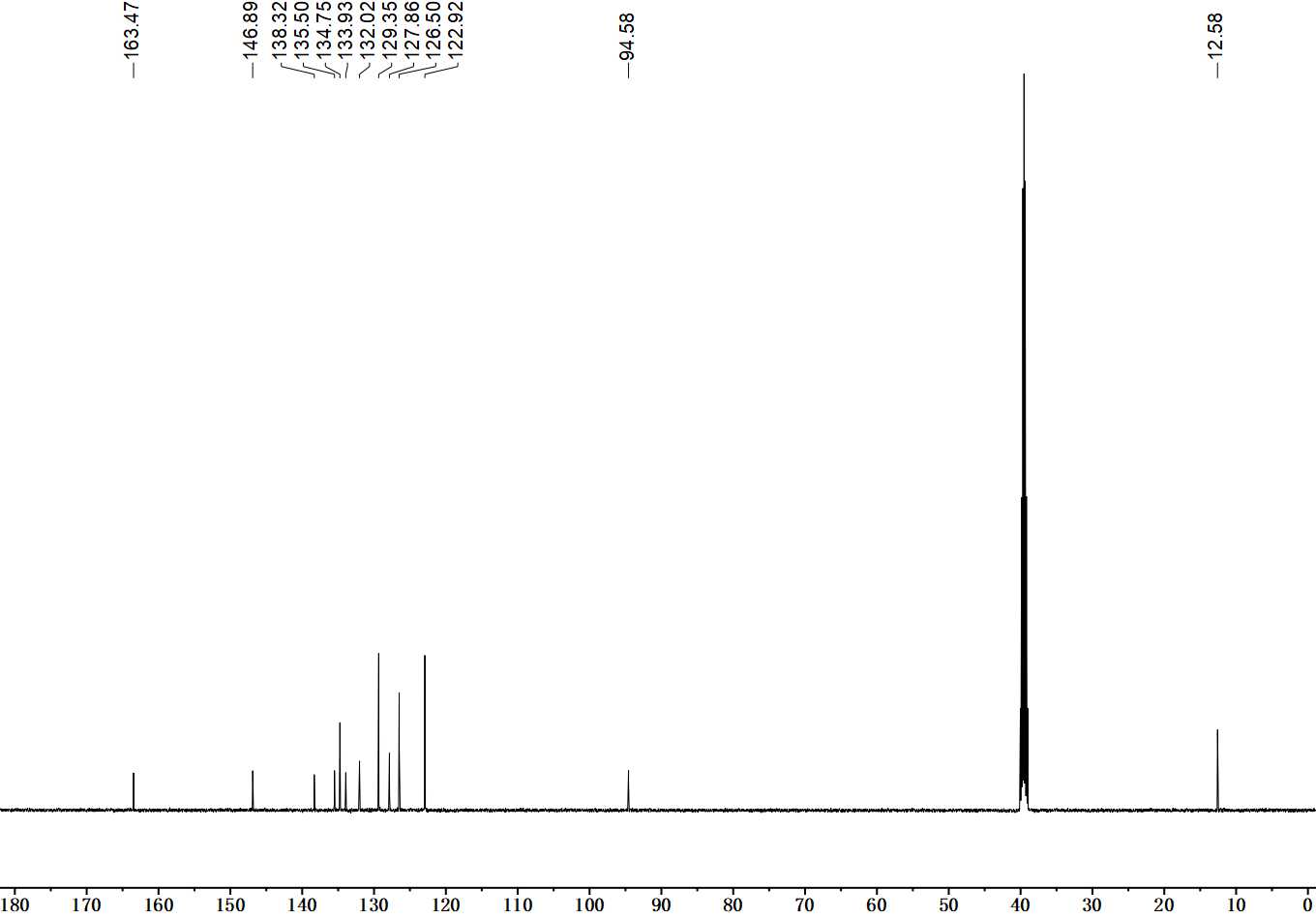
**

^1^H NMR Spectra of **Py-12**

**
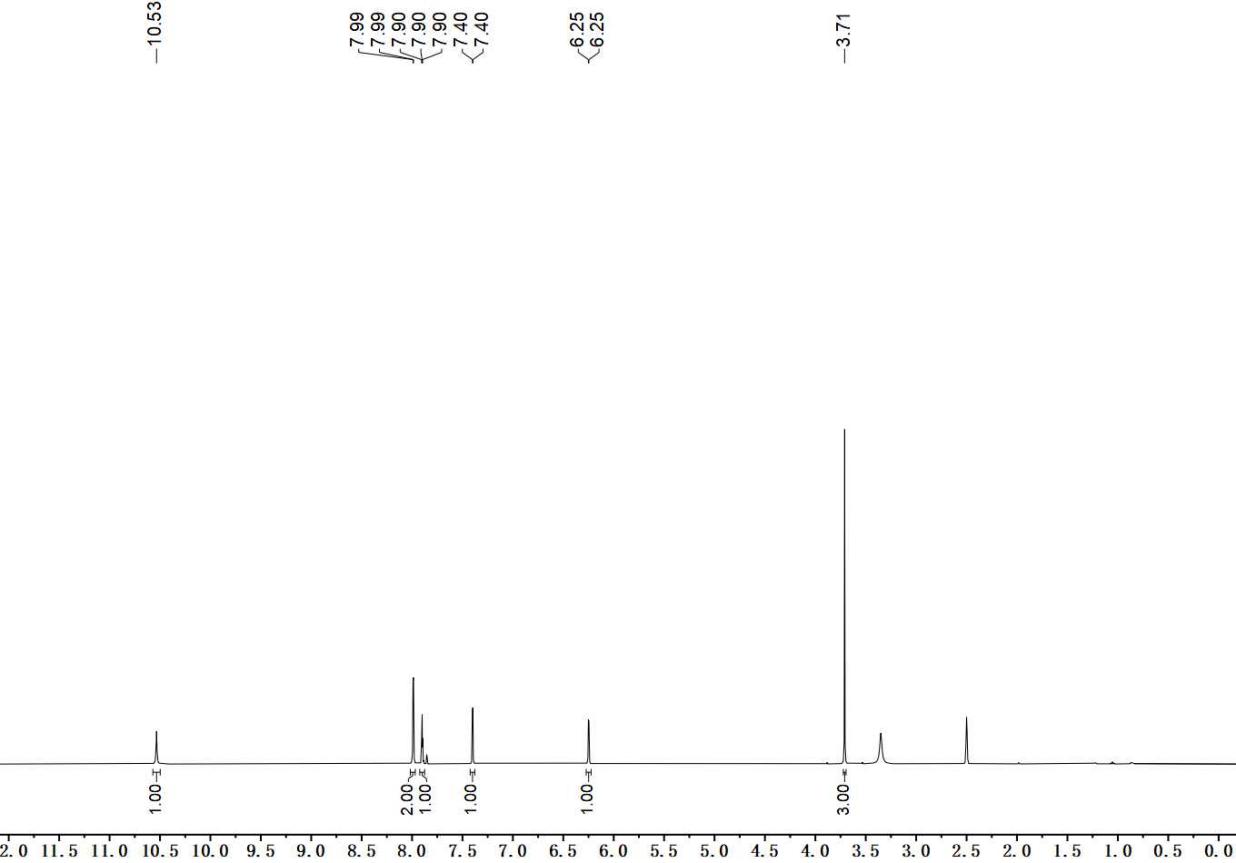
**

^13^C NMR Spectra of **Py-12**

**
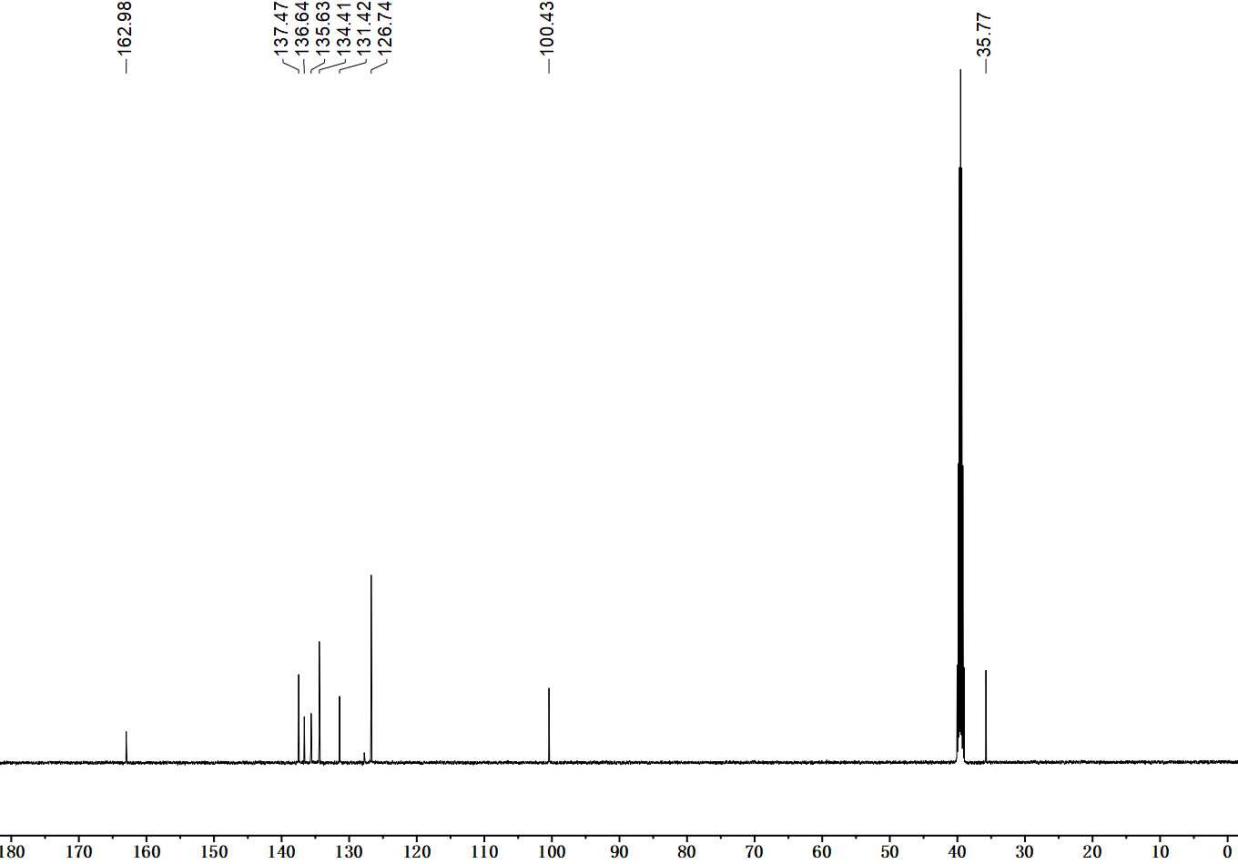
**

^1^H NMR Spectra of **Py-13**

**
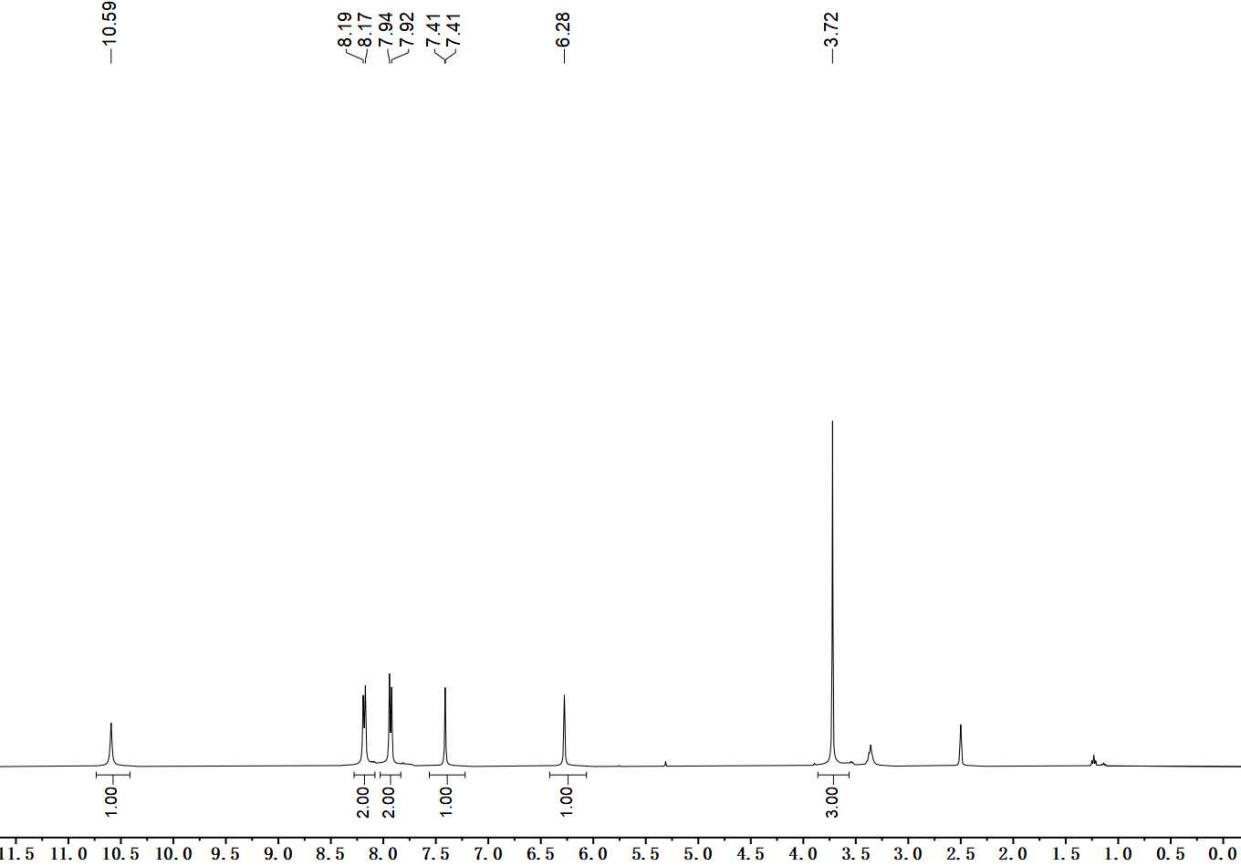
**

^13^C NMR Spectra of **Py-13**

**
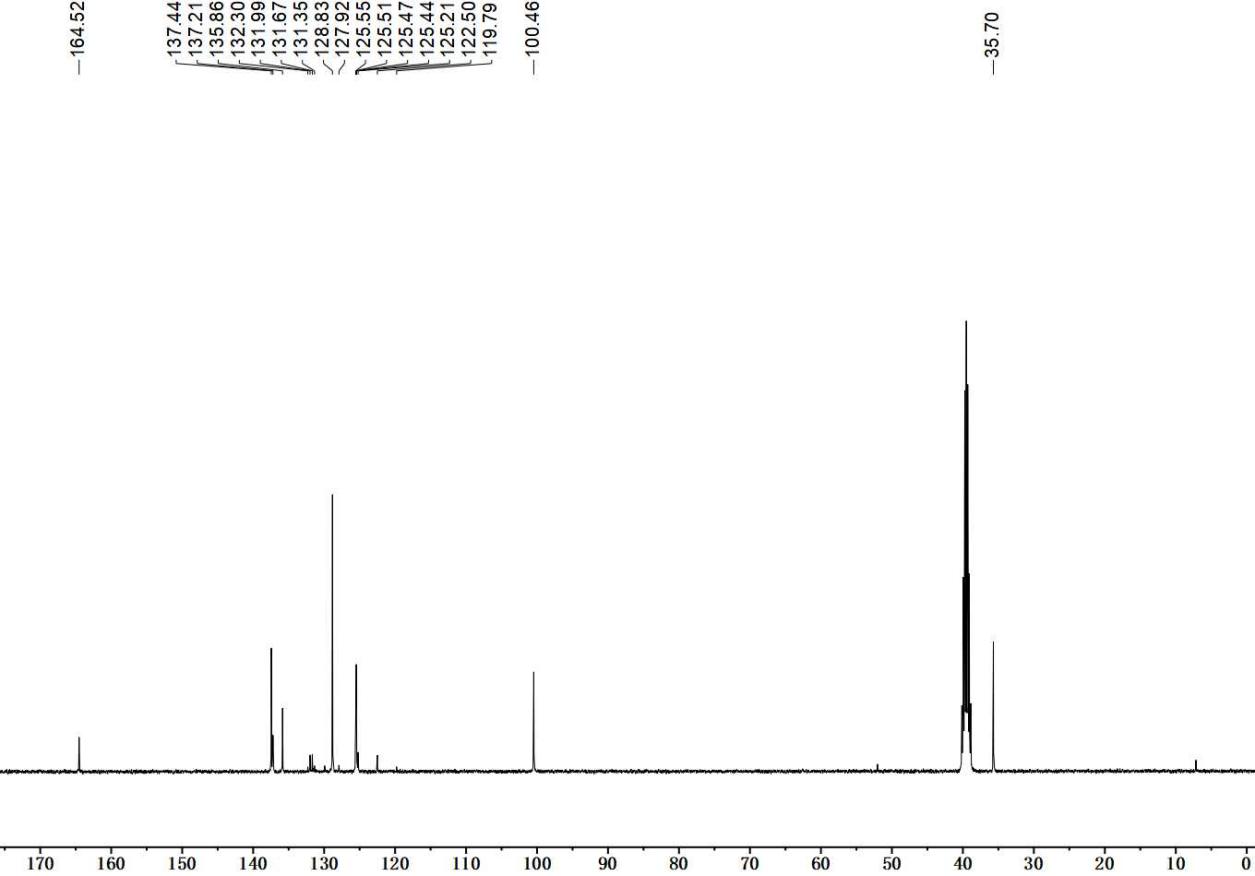
**

^1^H NMR Spectra of **Py-14**

**
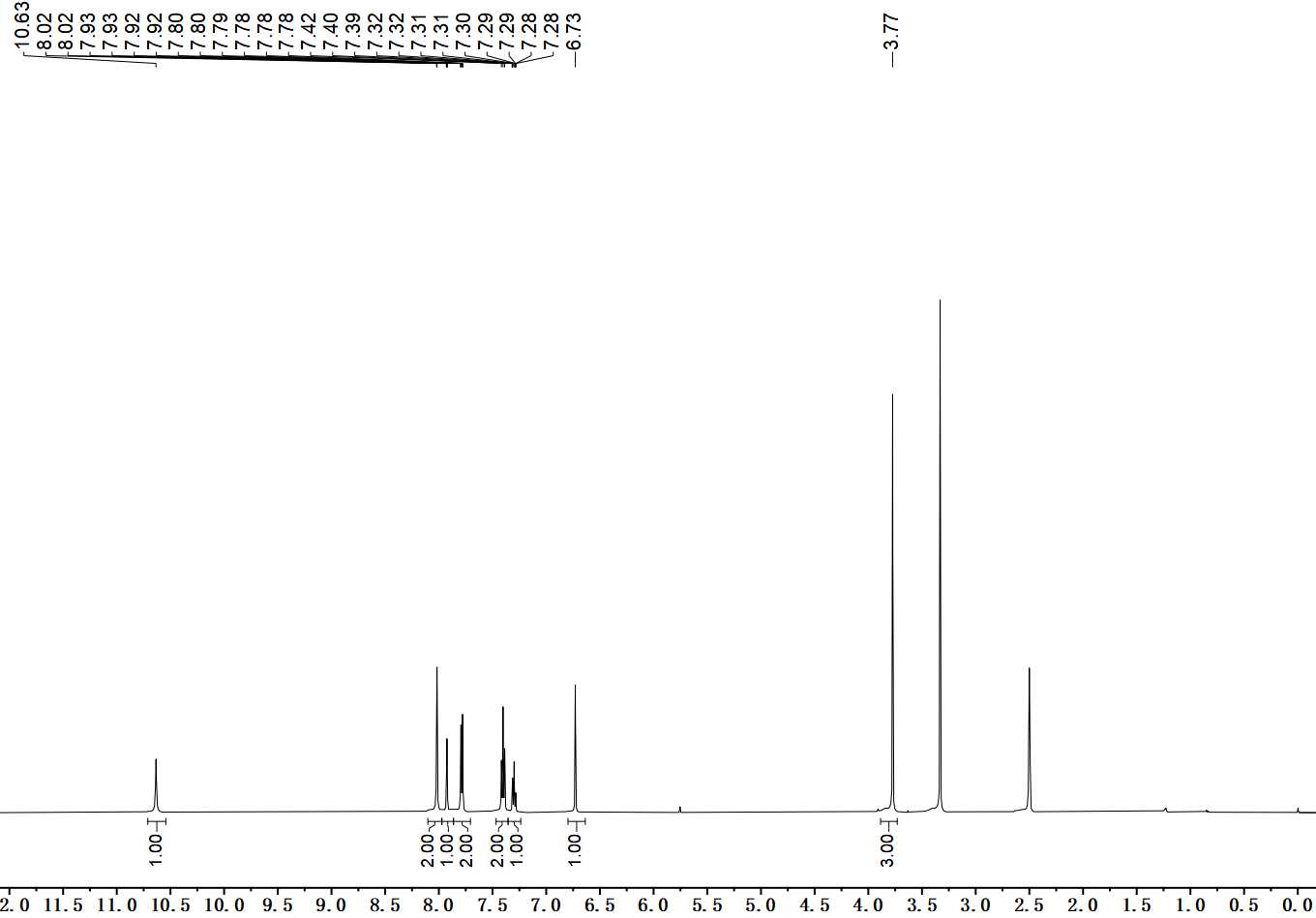
**

^13^C NMR Spectra of **Py-14**

**
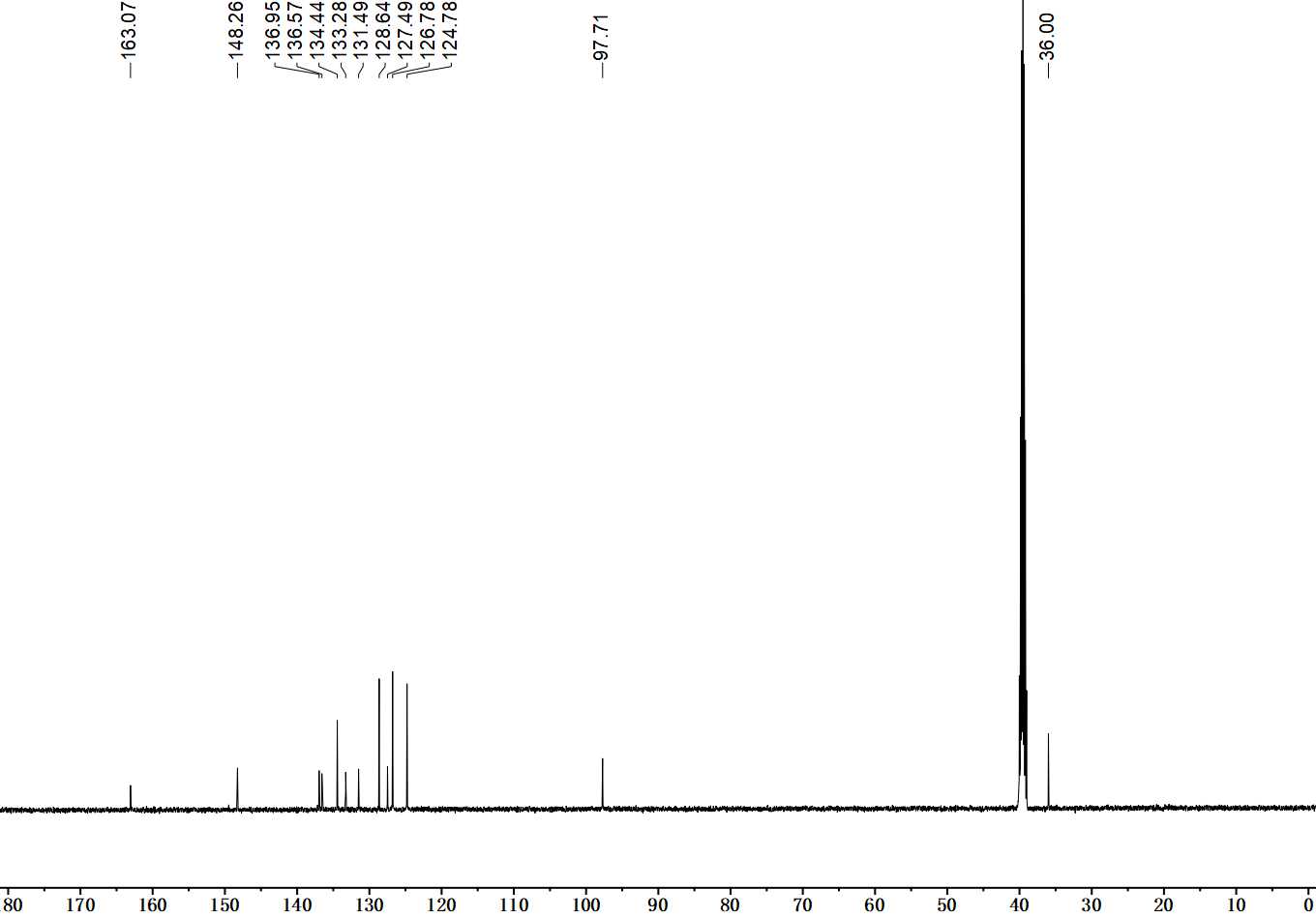
**

^1^H NMR Spectra of **Py-15**

**
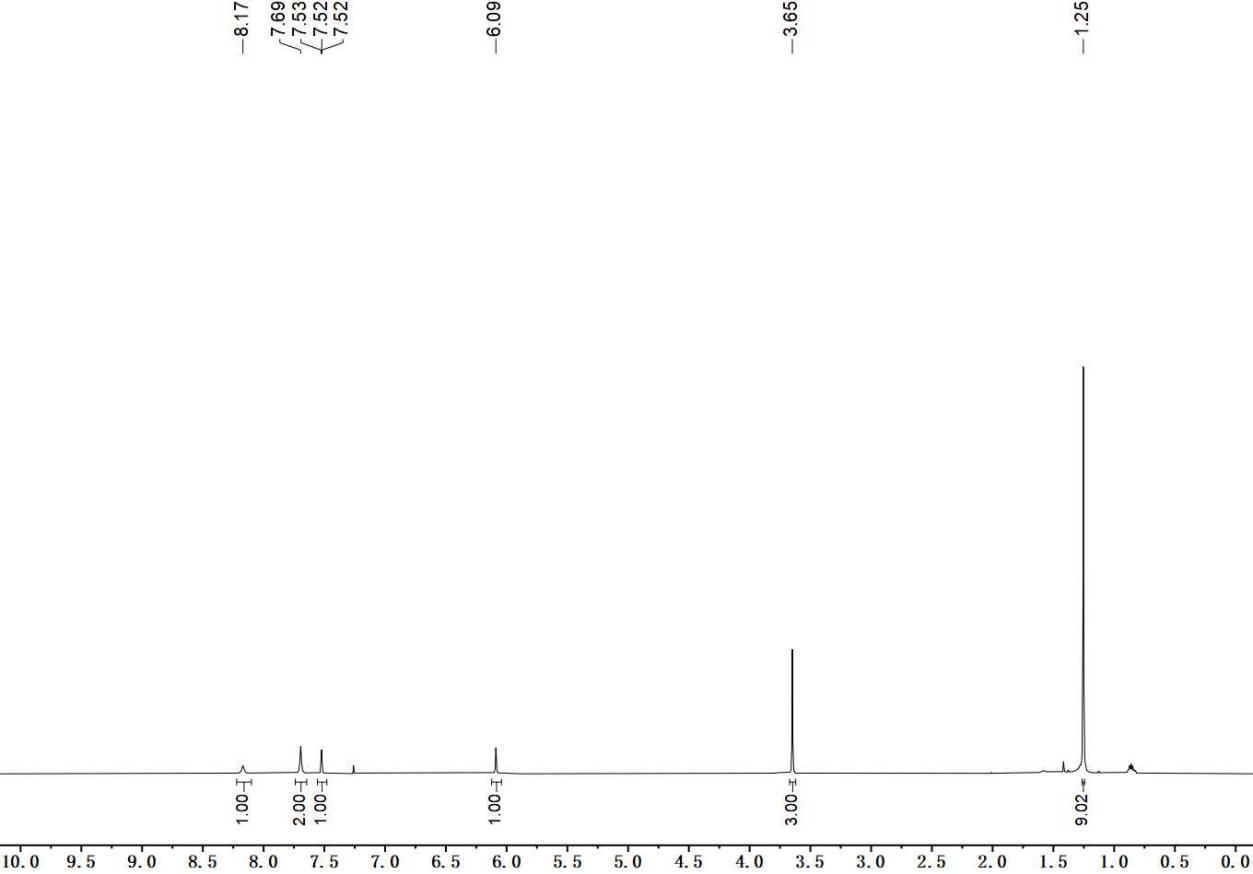
**

^13^C NMR Spectra of **Py-15**

**
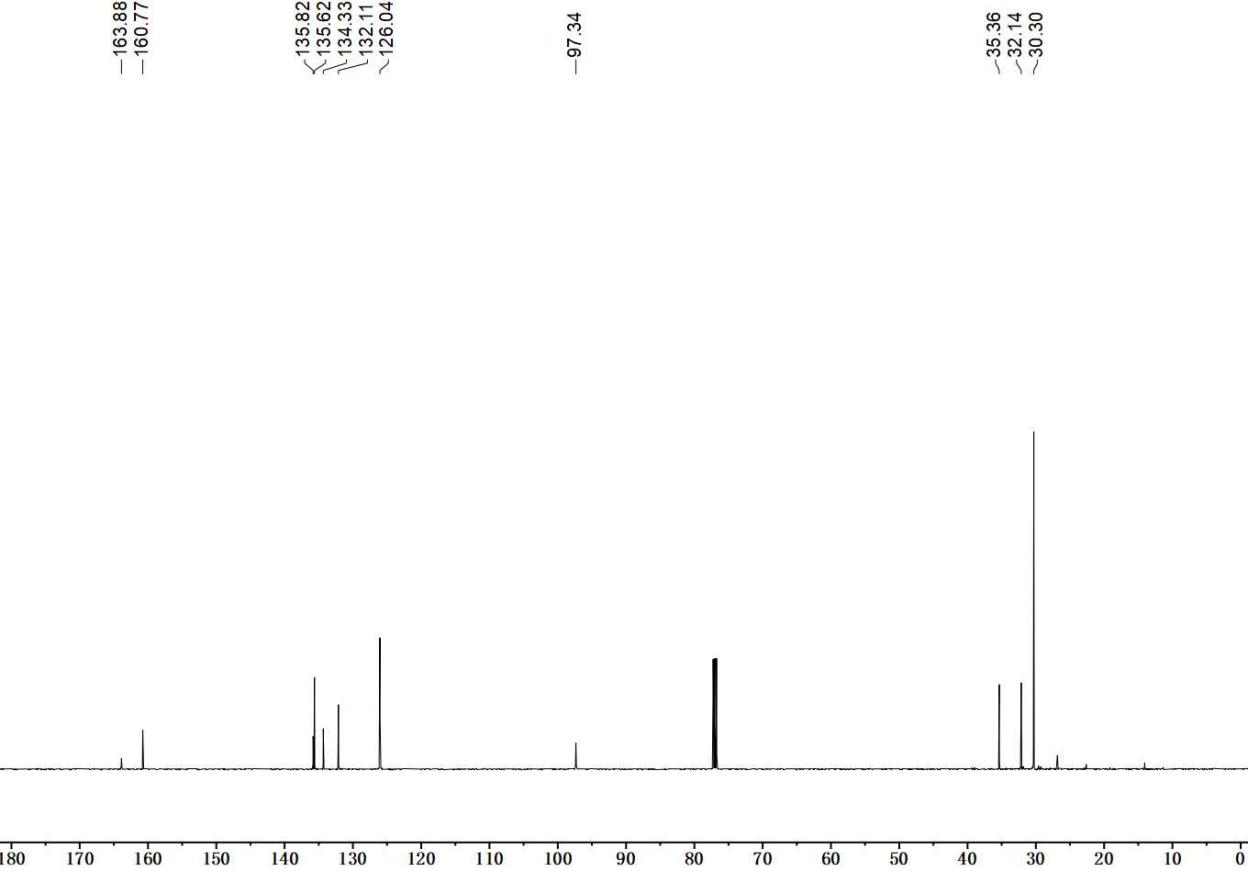
**

^1^H NMR Spectra of **Py-16**

**
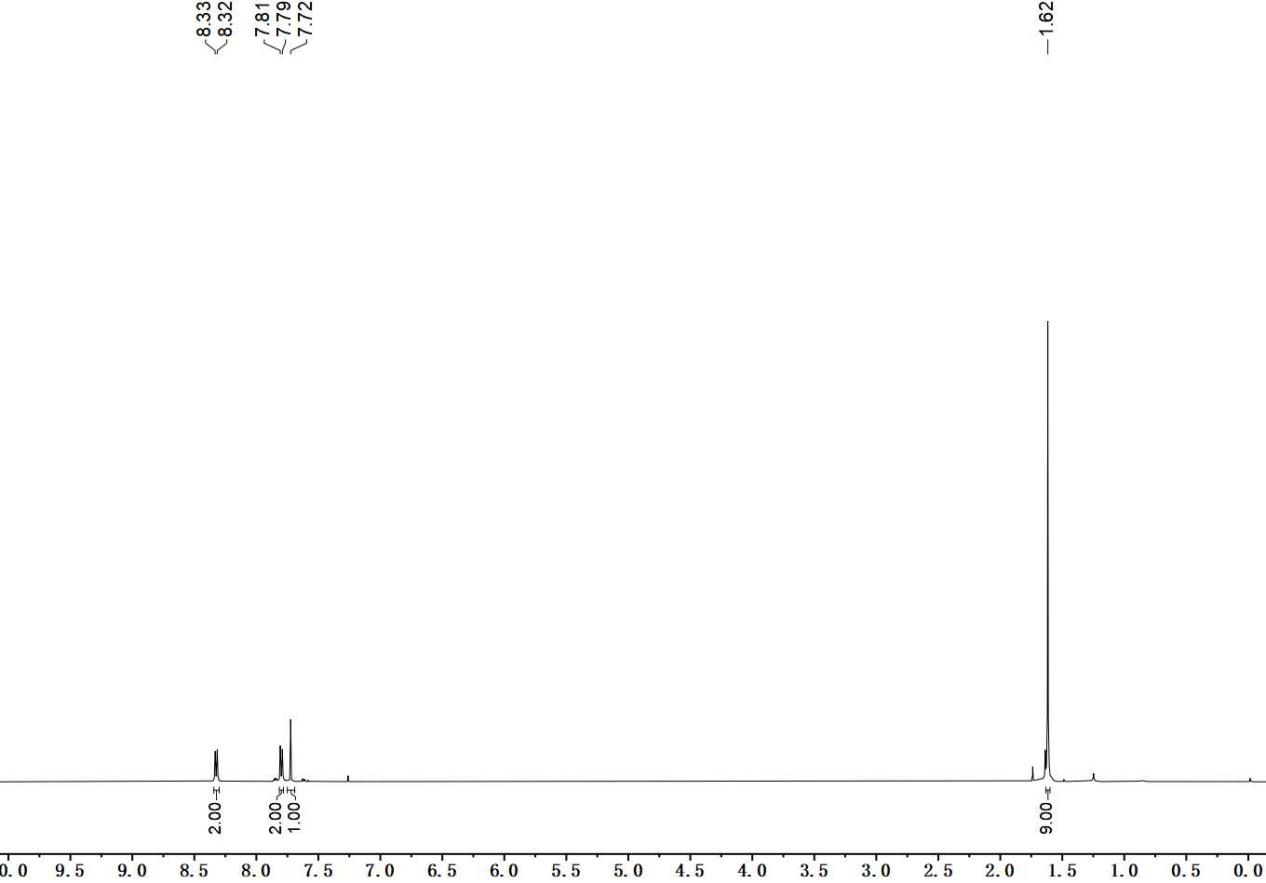
**

^13^C NMR Spectra of **Py-16**

**
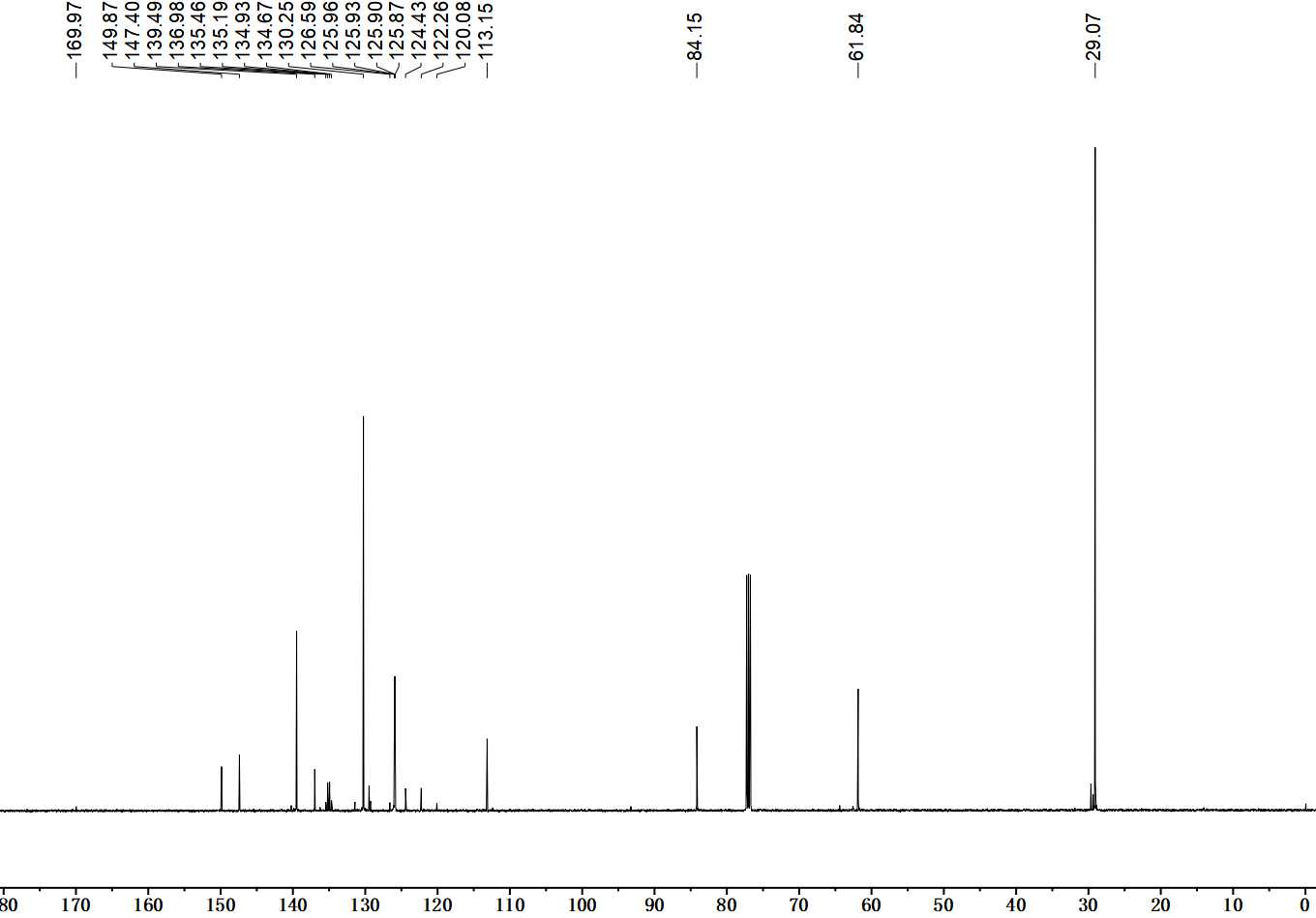
**

^1^H NMR Spectra of **Py-17**

**
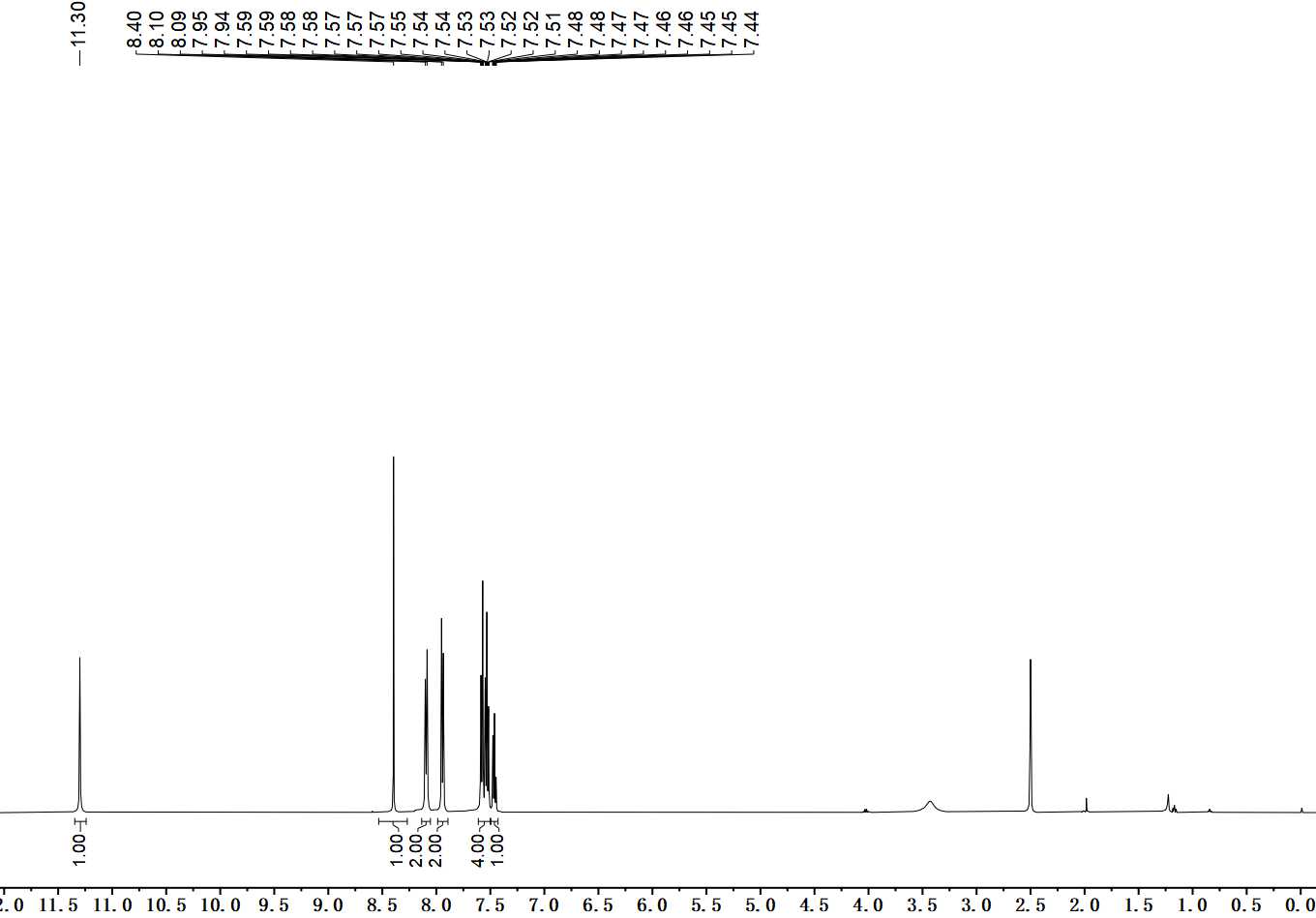
**

^13^C NMR Spectra of **Py-17**

**
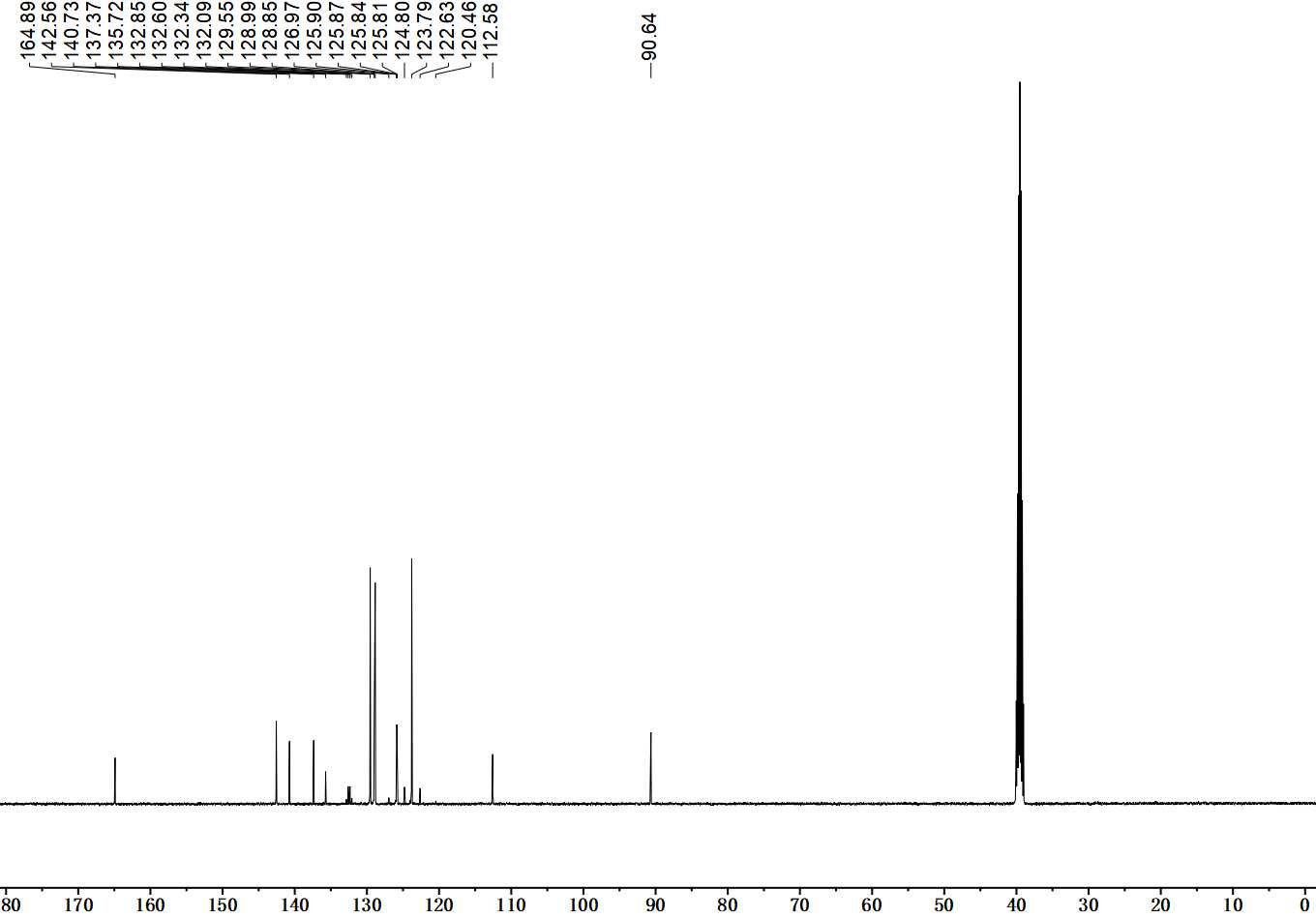
**

^1^H NMR Spectra of **Py-18**

**
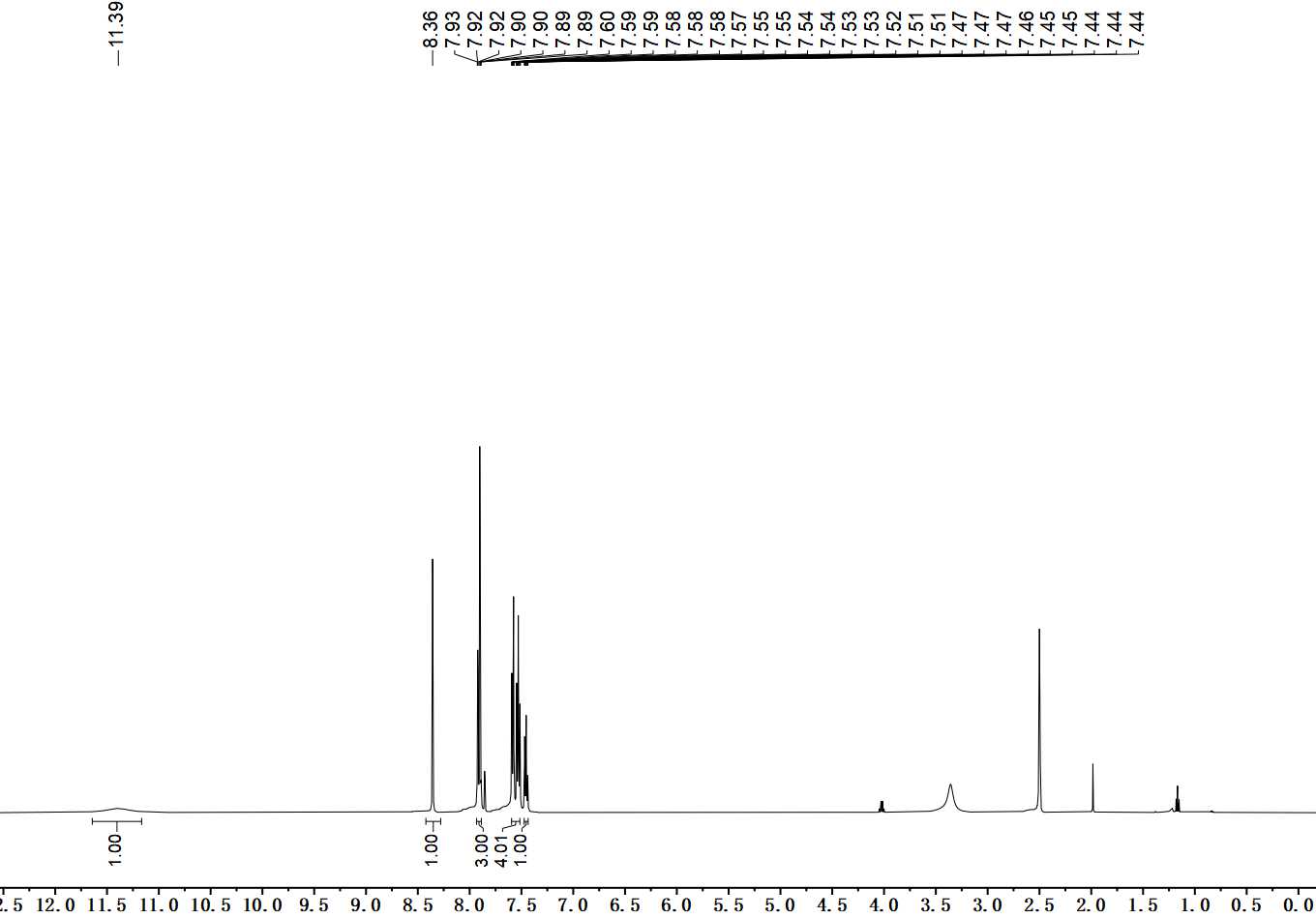
**

^13^C NMR Spectra of **Py-18**

**
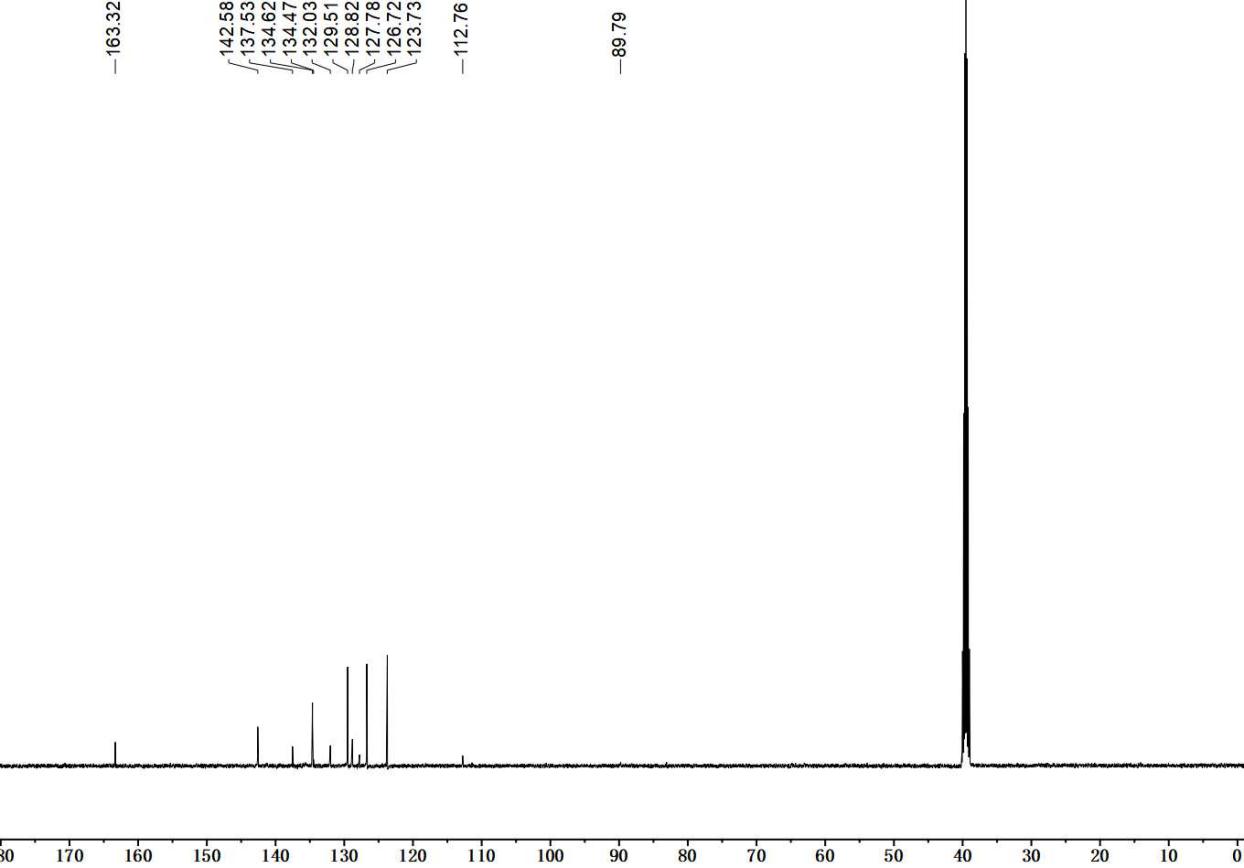
**

^1^H NMR Spectra of **Py-19**

**
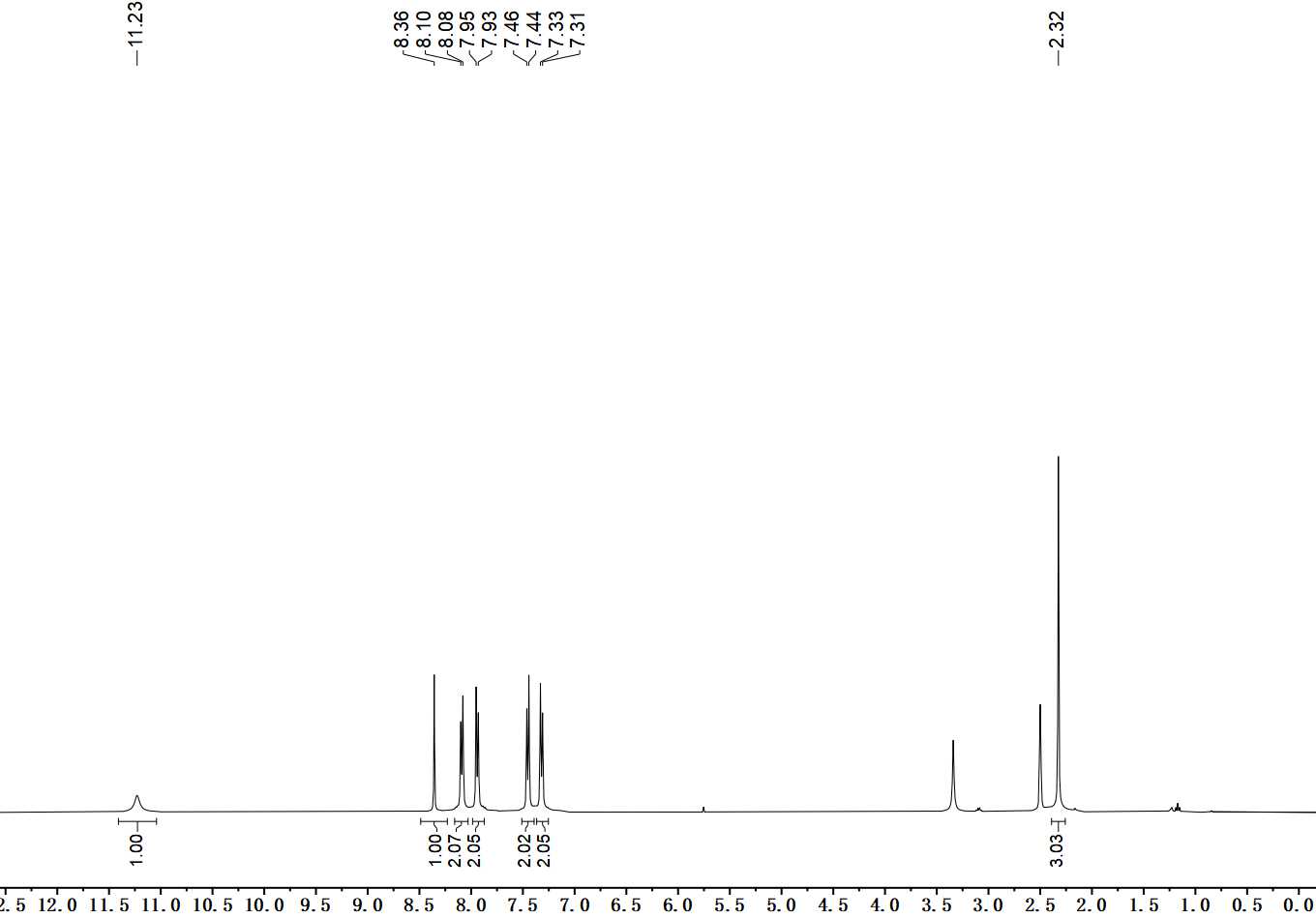
**

^13^C NMR Spectra of **Py-19**

**
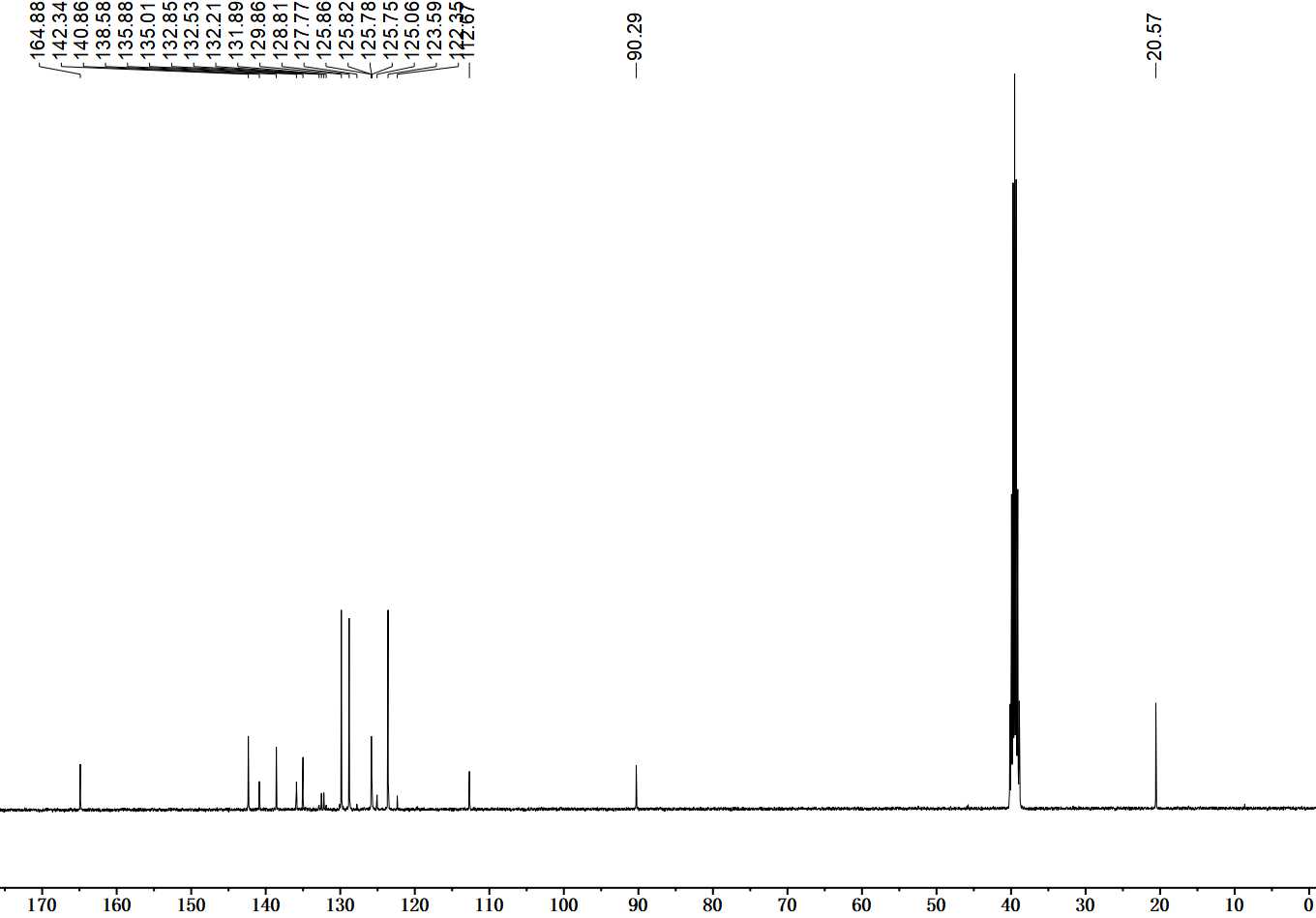
**

^1^H NMR Spectra of **Py-20**

**
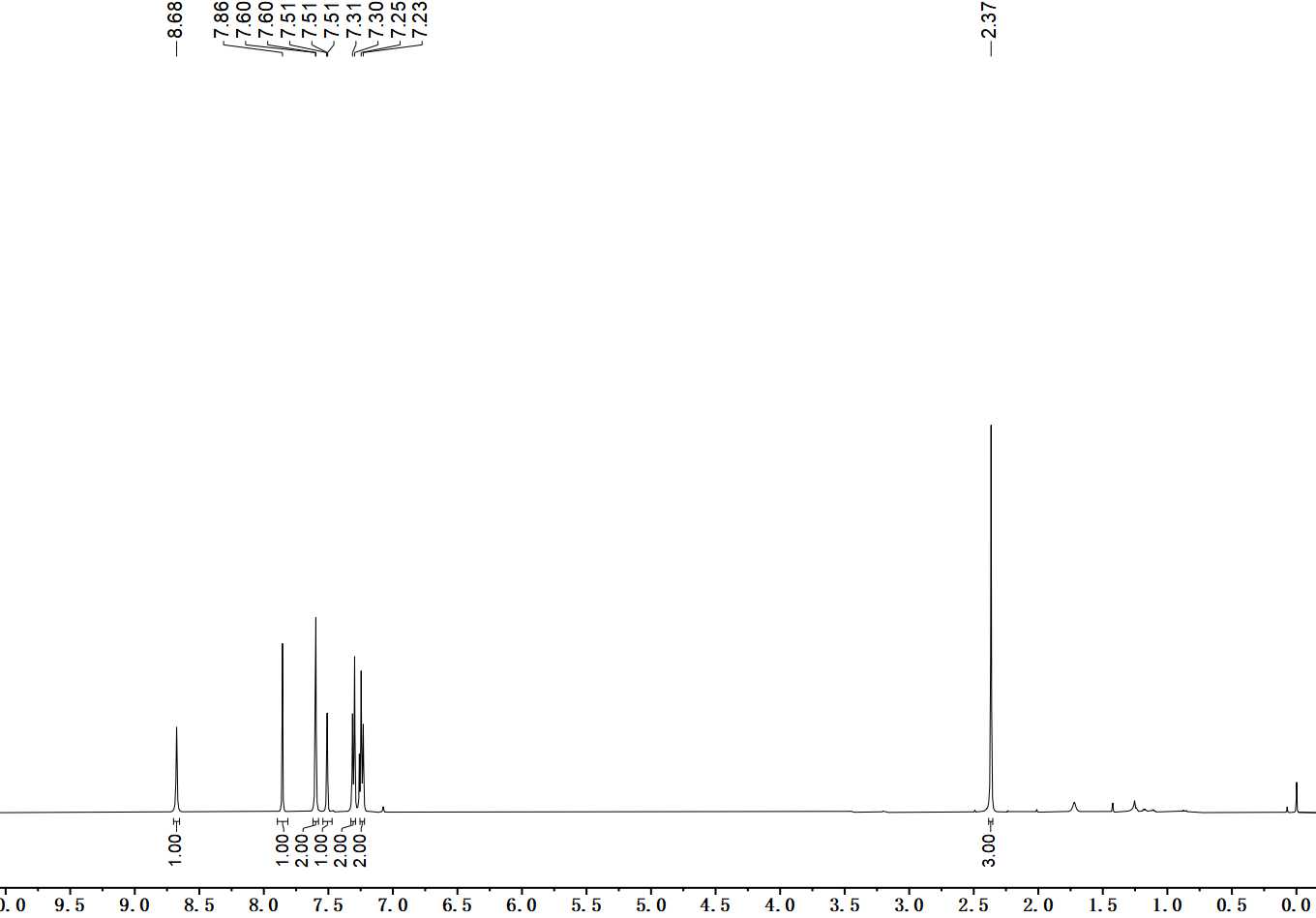
**

^13^C NMR Spectra of **Py-20**

**
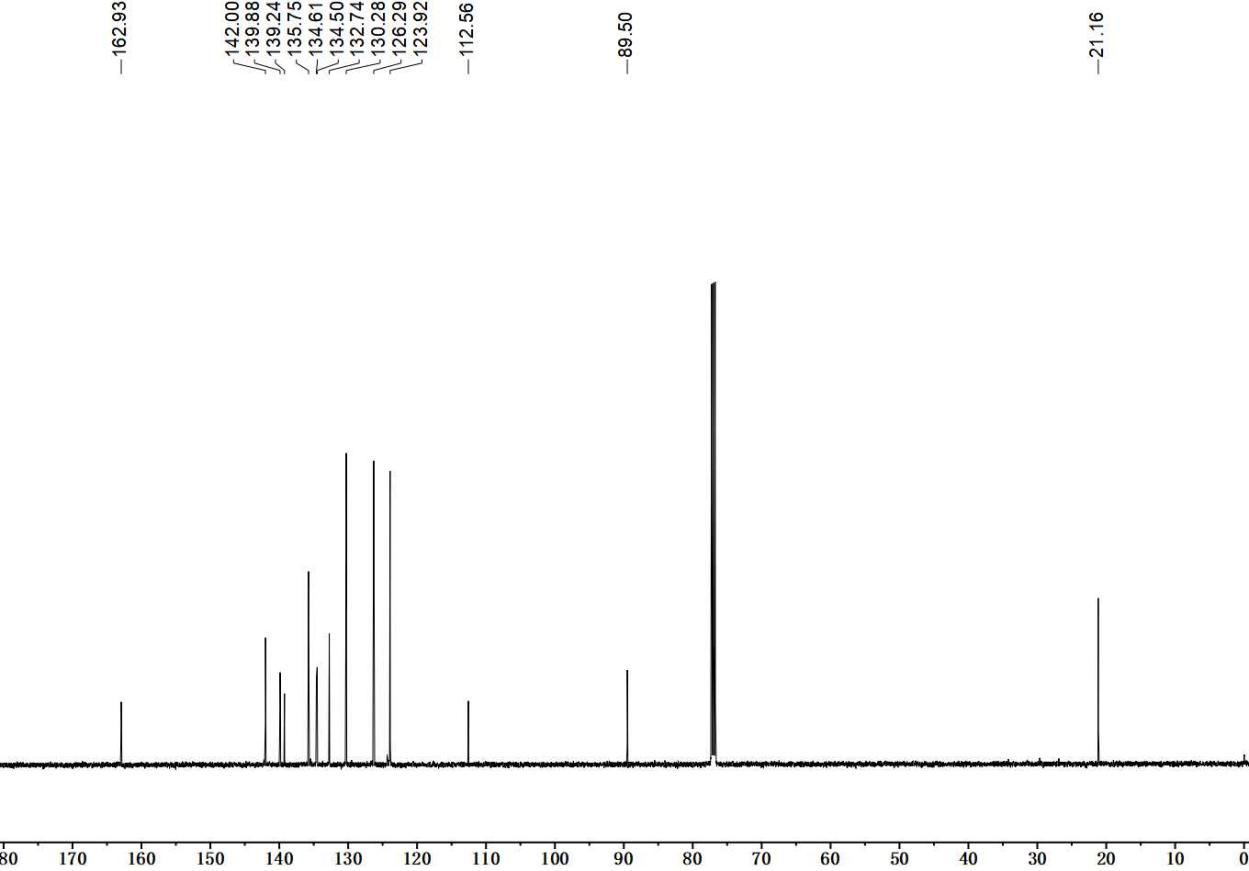
**

^1^H NMR Spectra of **Py-21**

**
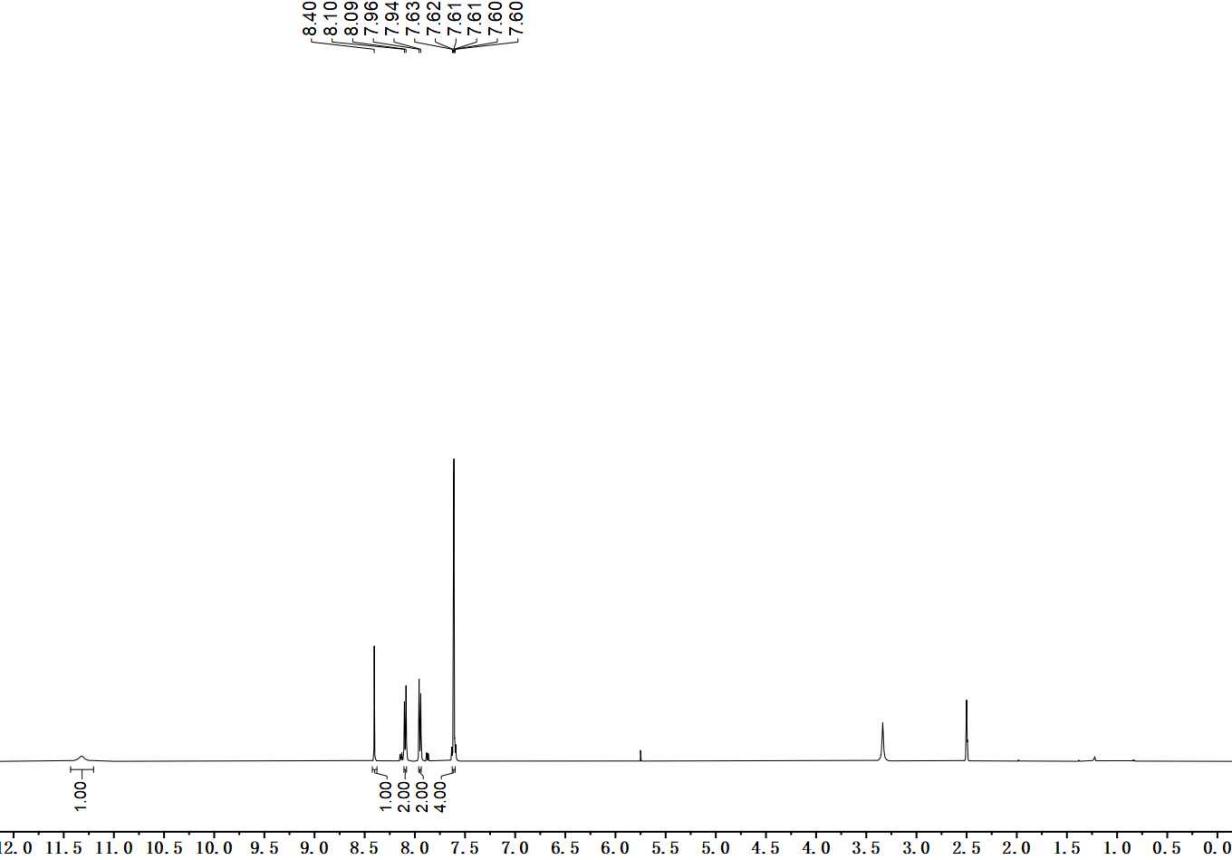
**

^13^C NMR Spectra of **Py-21**

**
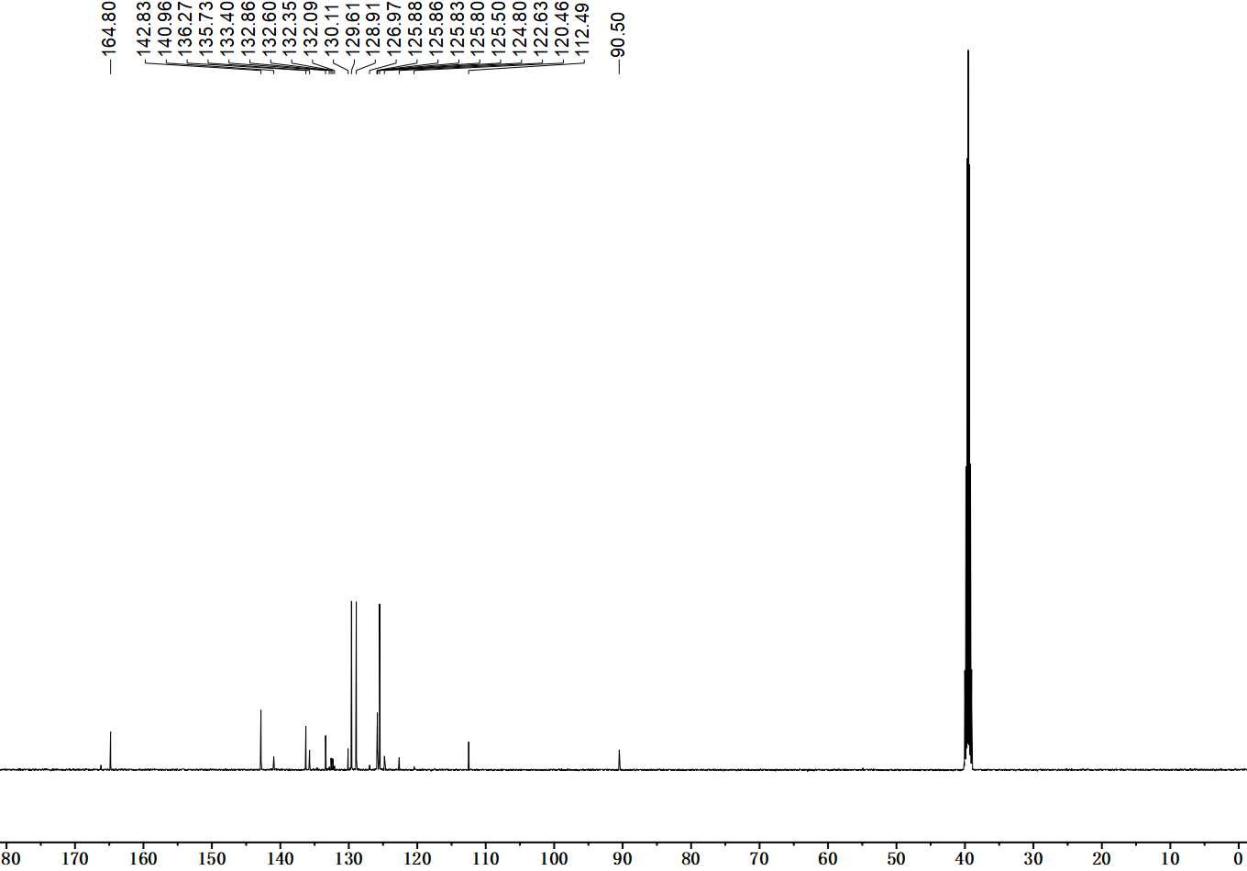
**

^1^H NMR Spectra of **Py-22**

**
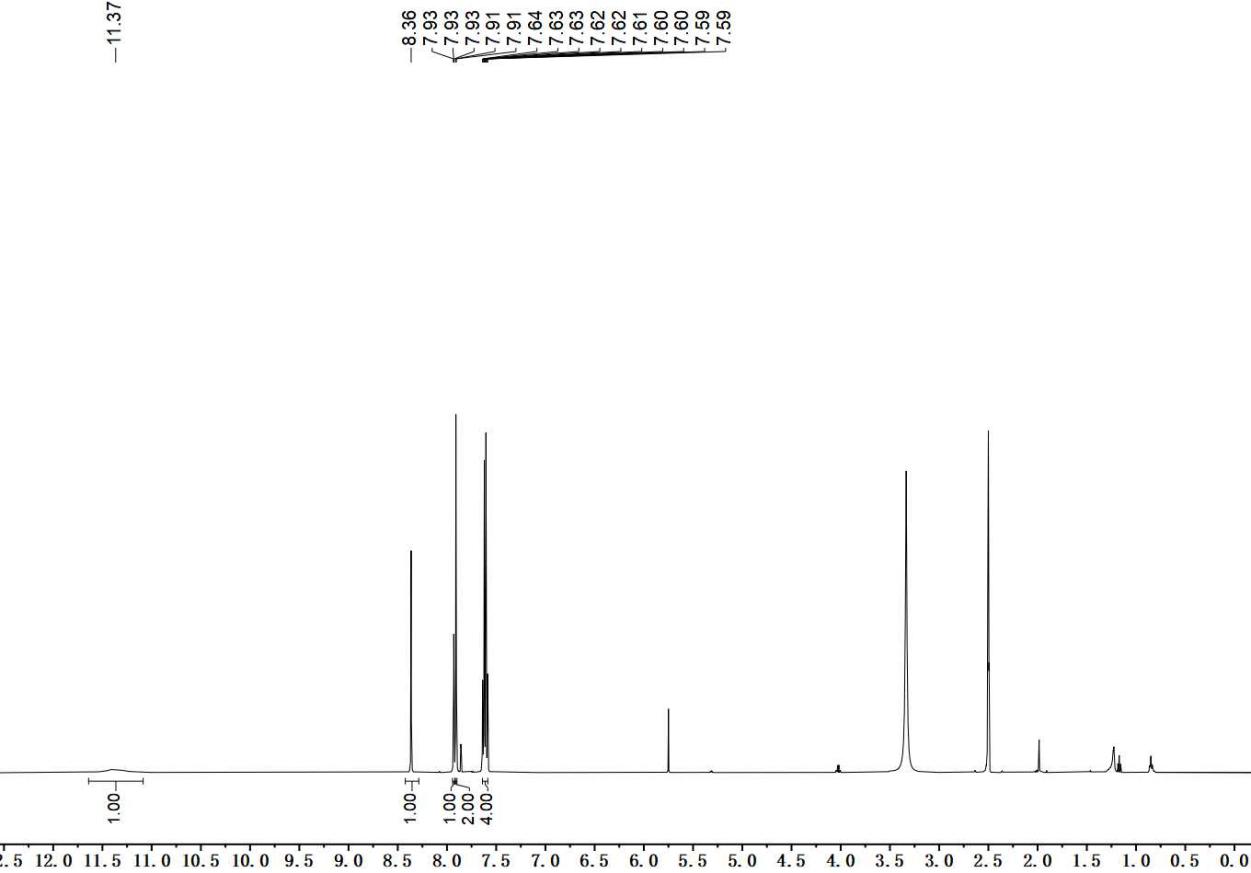
**

^13^C NMR Spectra of **Py-22**

**
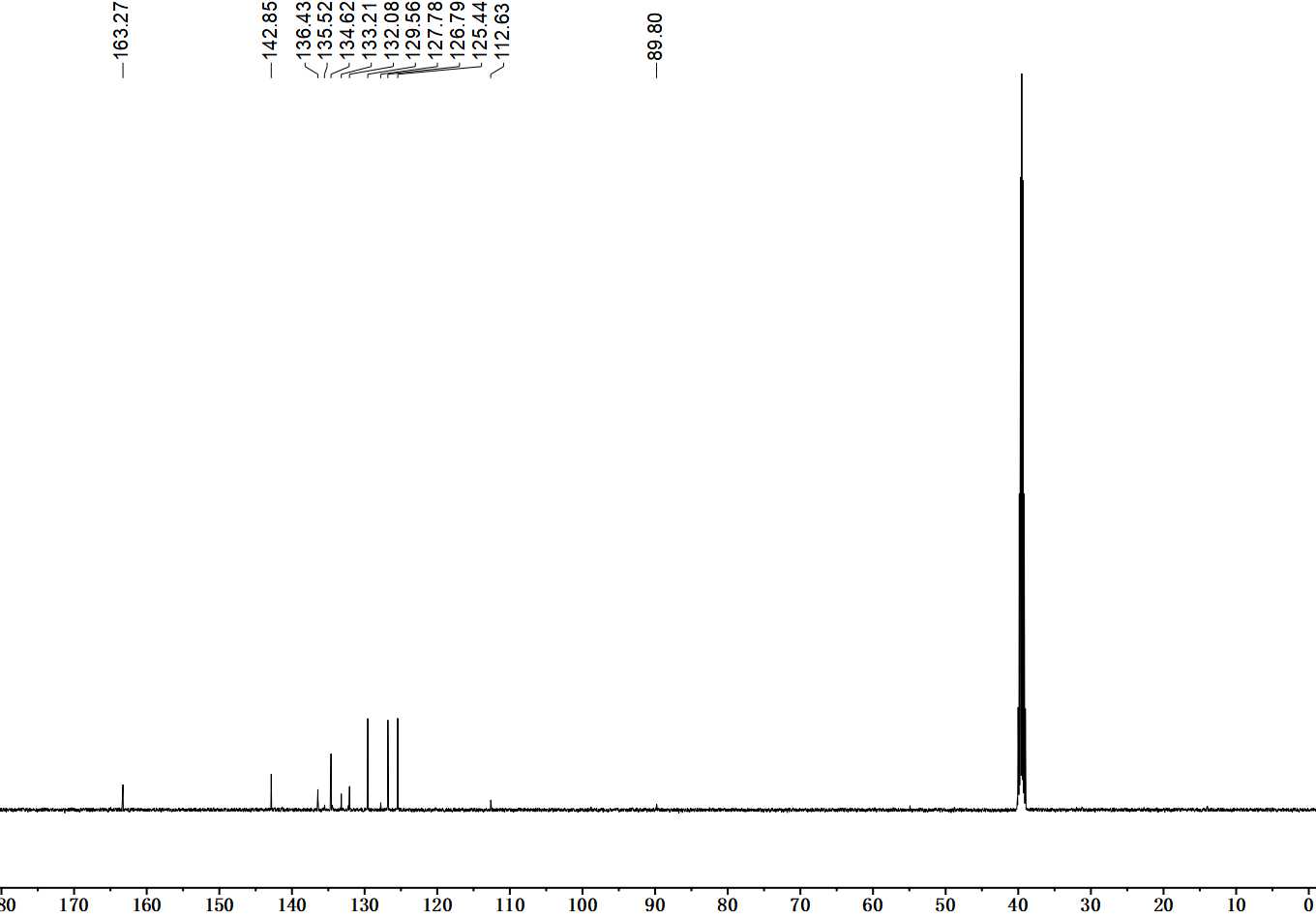
**

^1^H NMR Spectra of **Py-23**

**
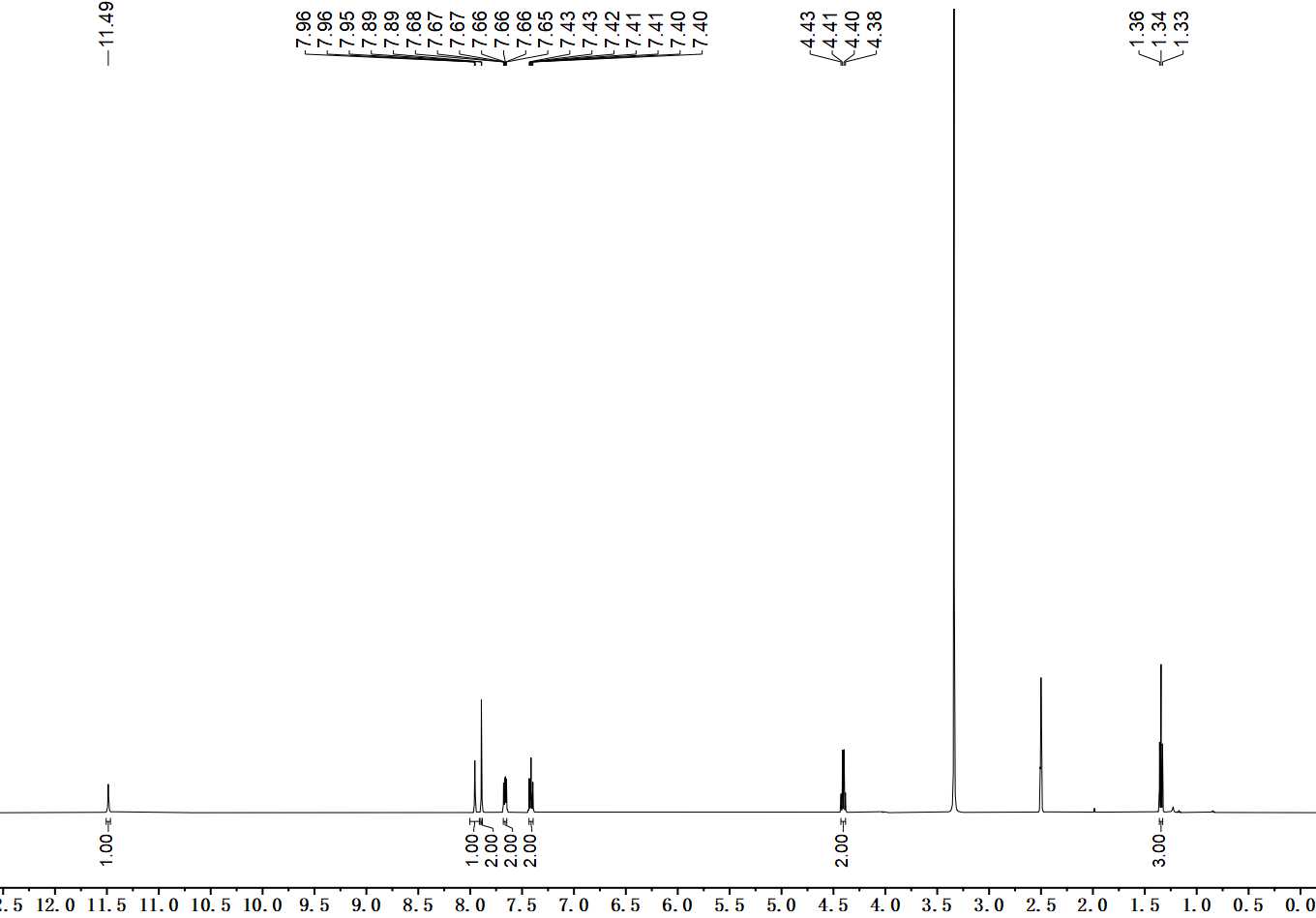
**

^13^C NMR Spectra of **Py-23**

**
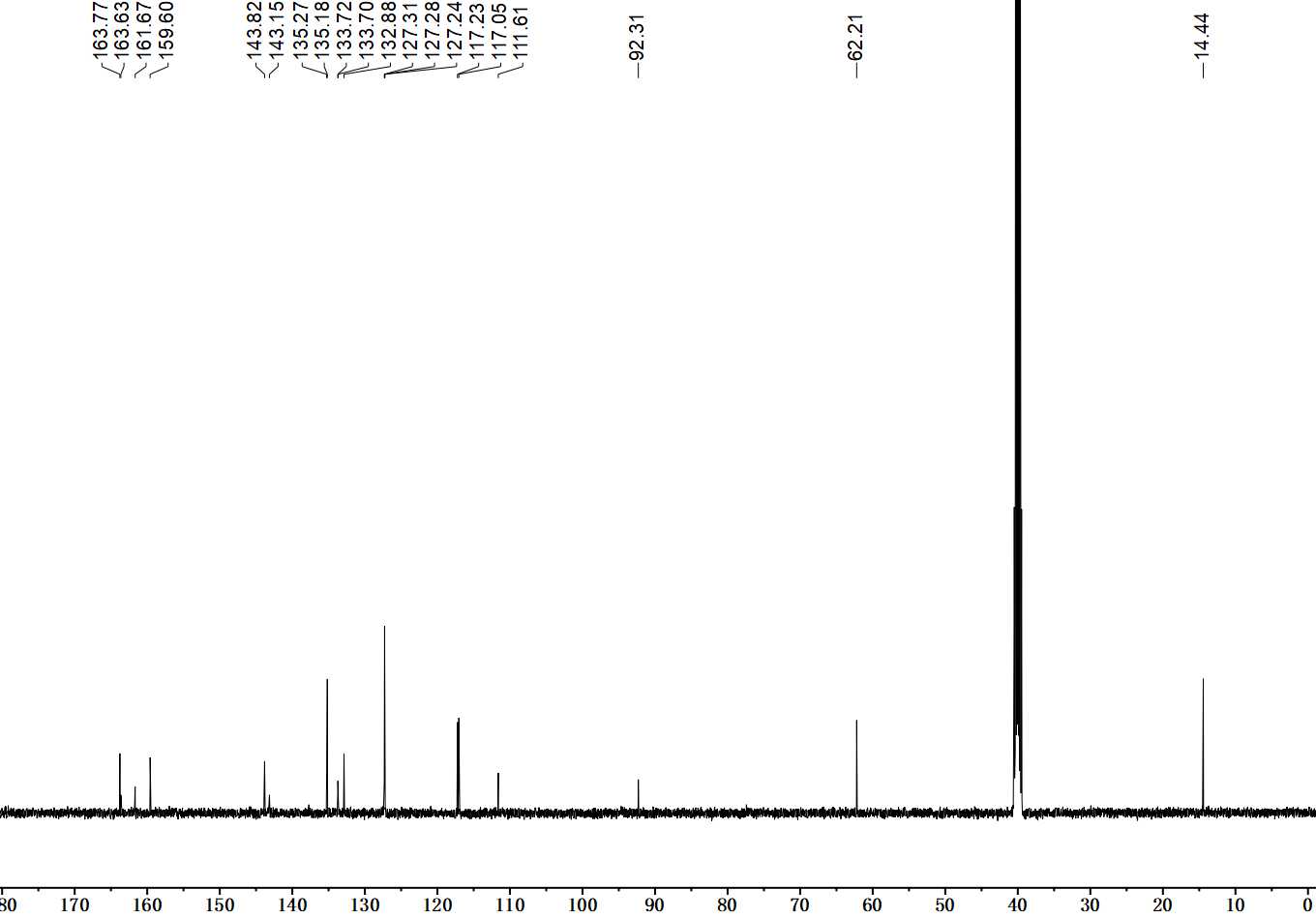
**

^1^H NMR Spectra of **Py-24**

**
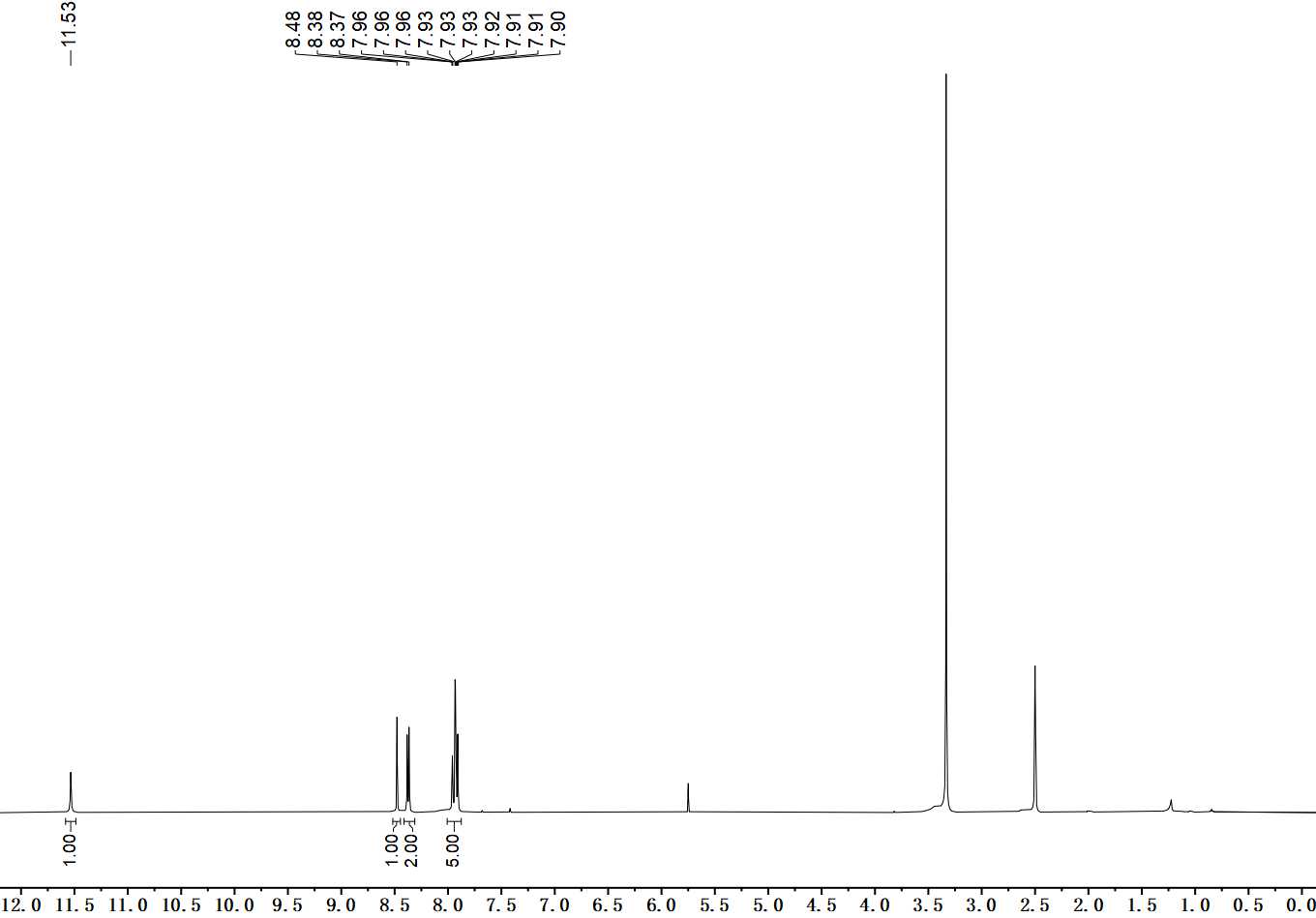
**

^13^C NMR Spectra of **Py-24**

**
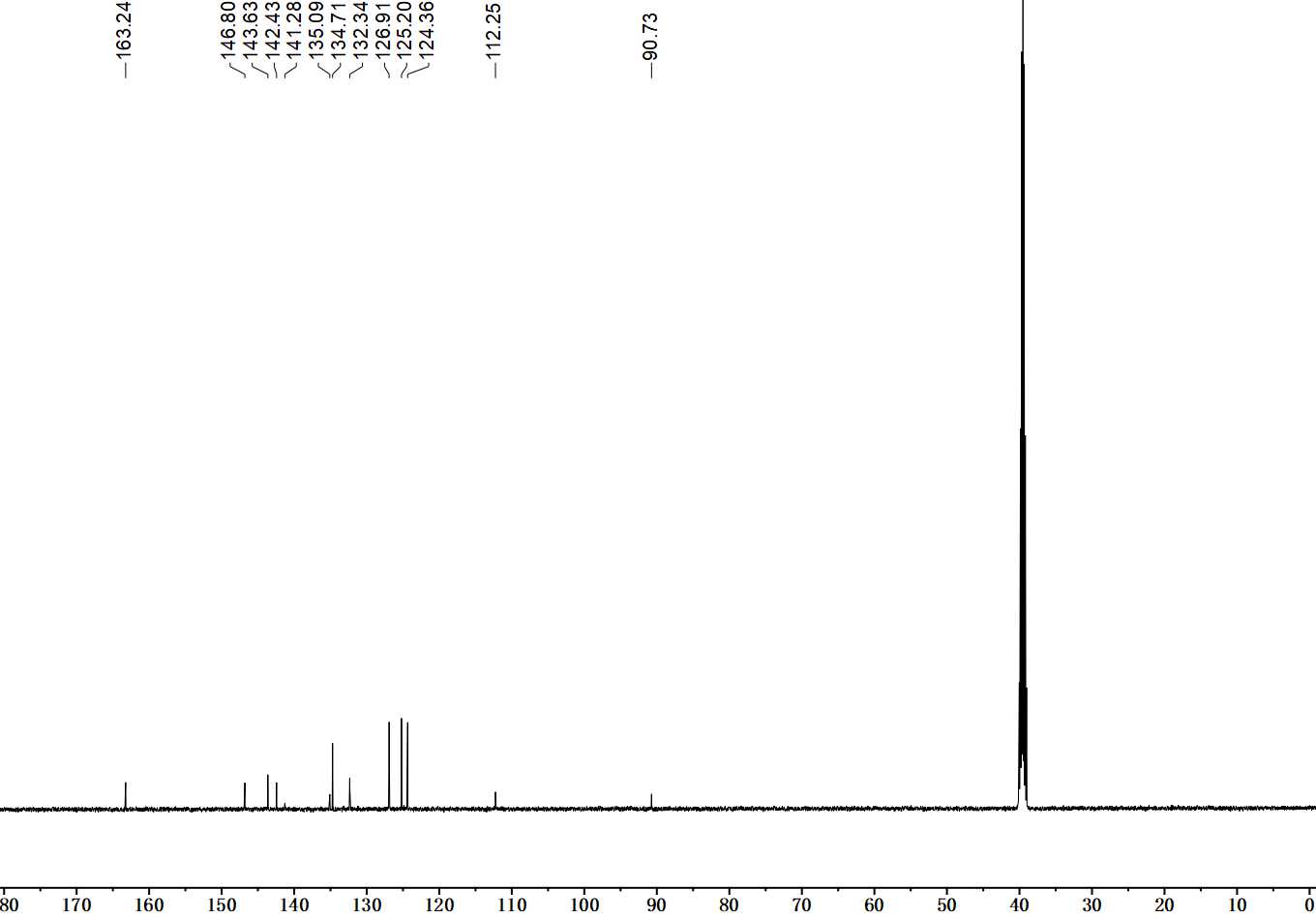
**

^1^H NMR Spectra of **Py-25**

**
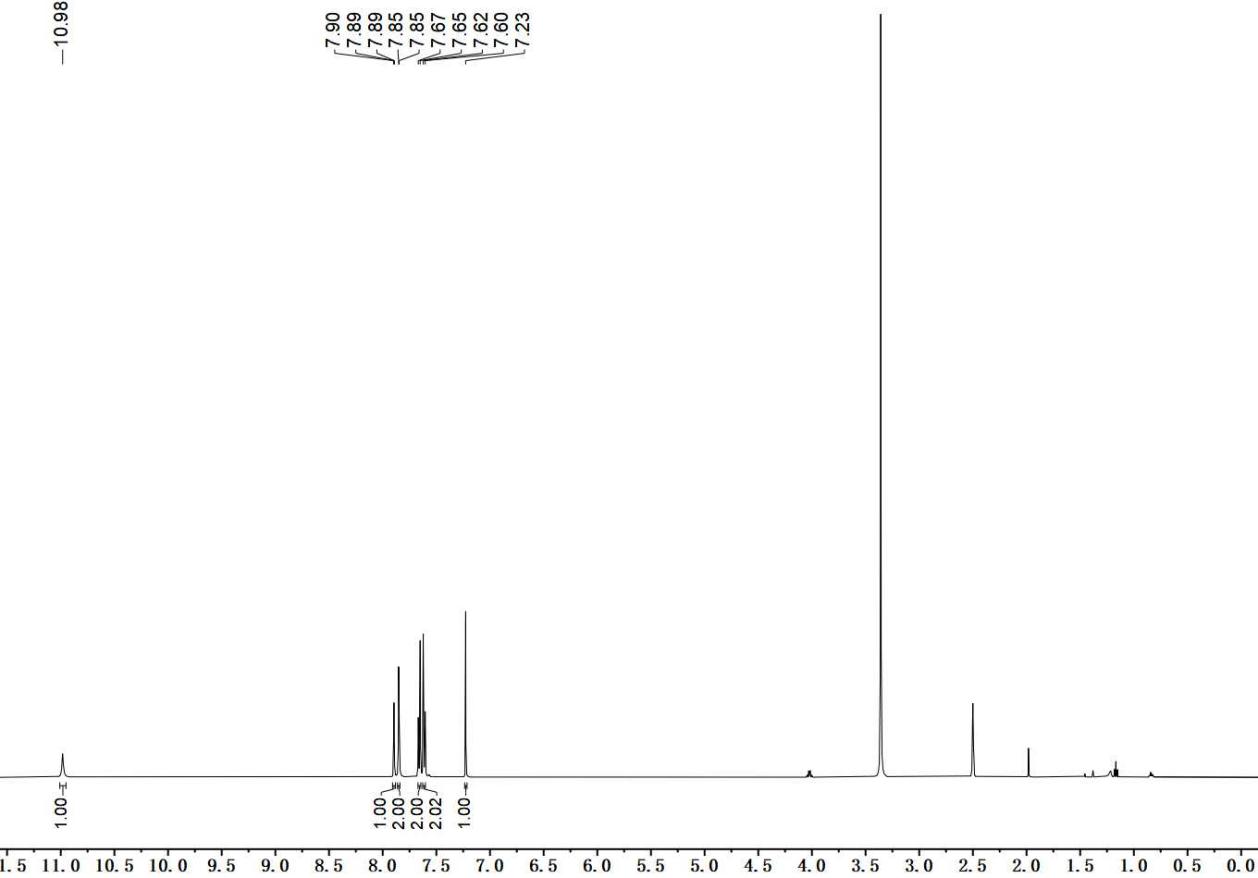
**

^13^C NMR Spectra of **Py-25**

**
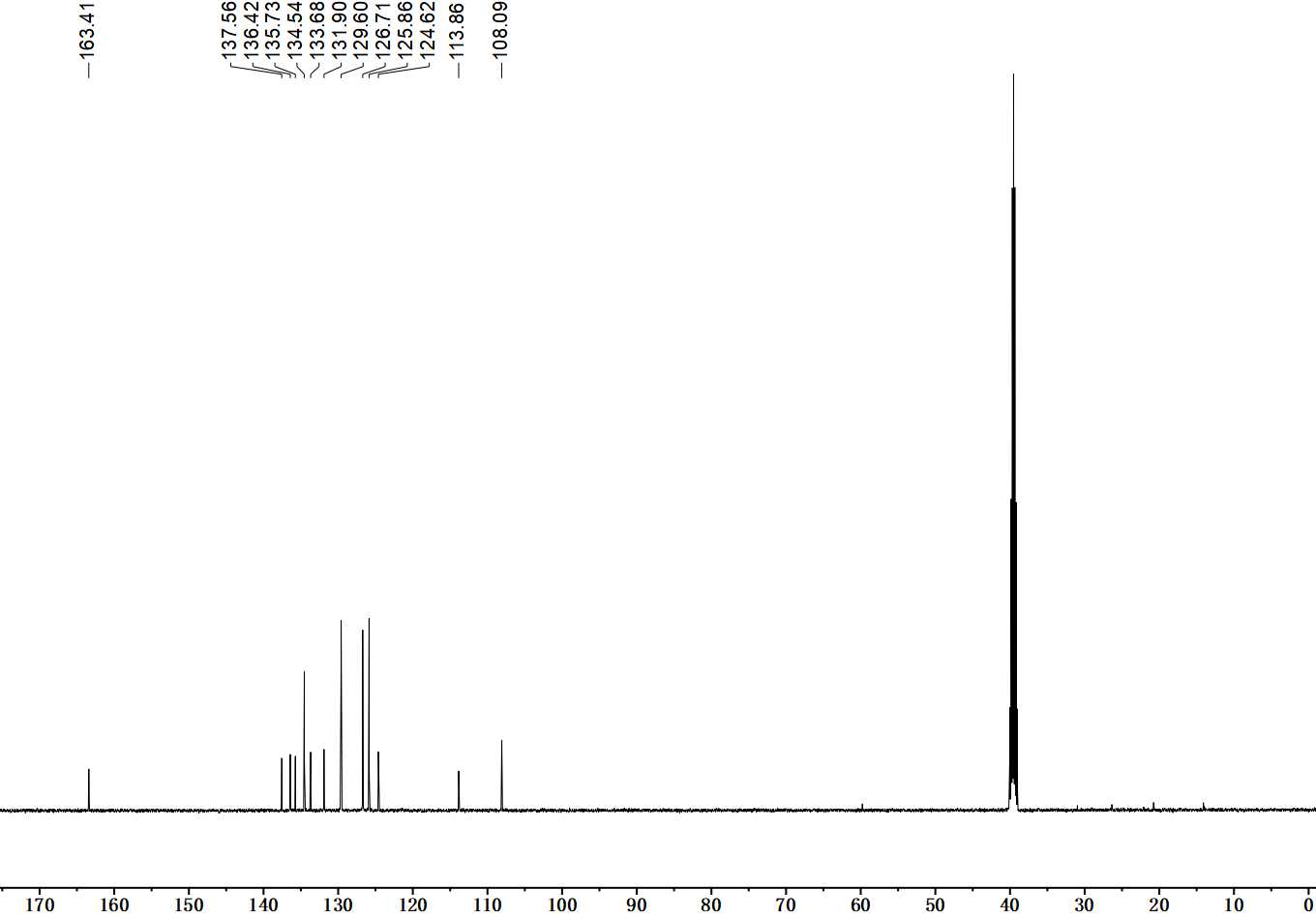
**

^1^H NMR Spectra of **Py-26**

**
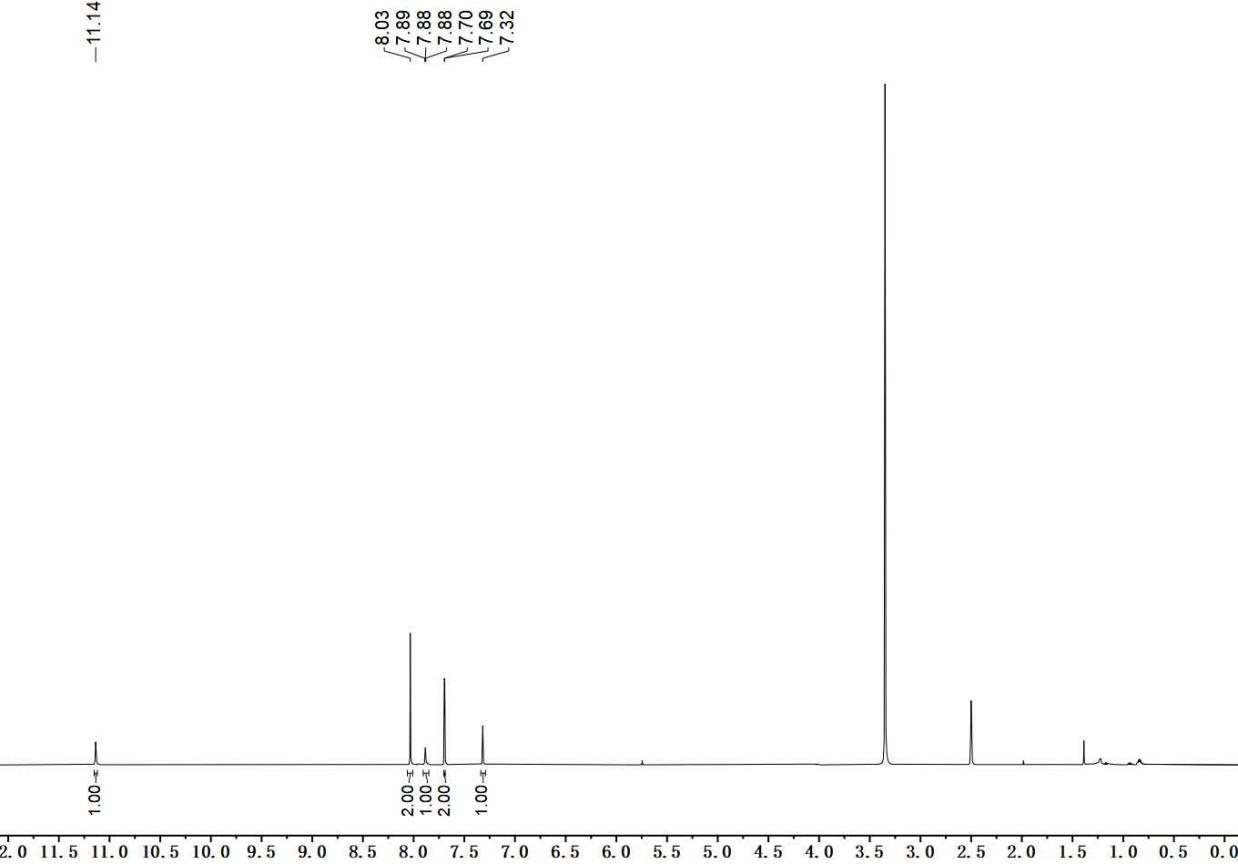
**

^13^C NMR Spectra of **Py-26**

**
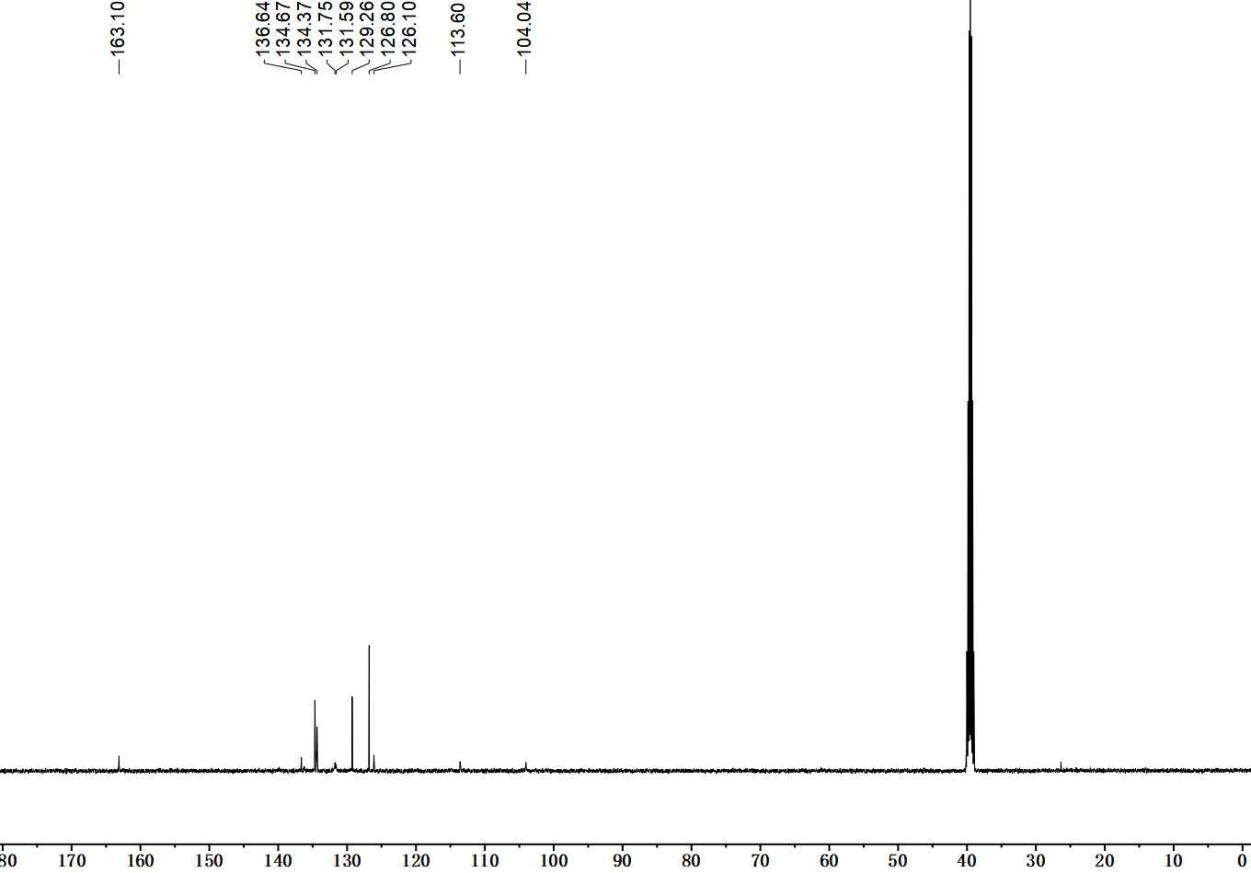
**

^1^H NMR Spectra of **Py-27**

**
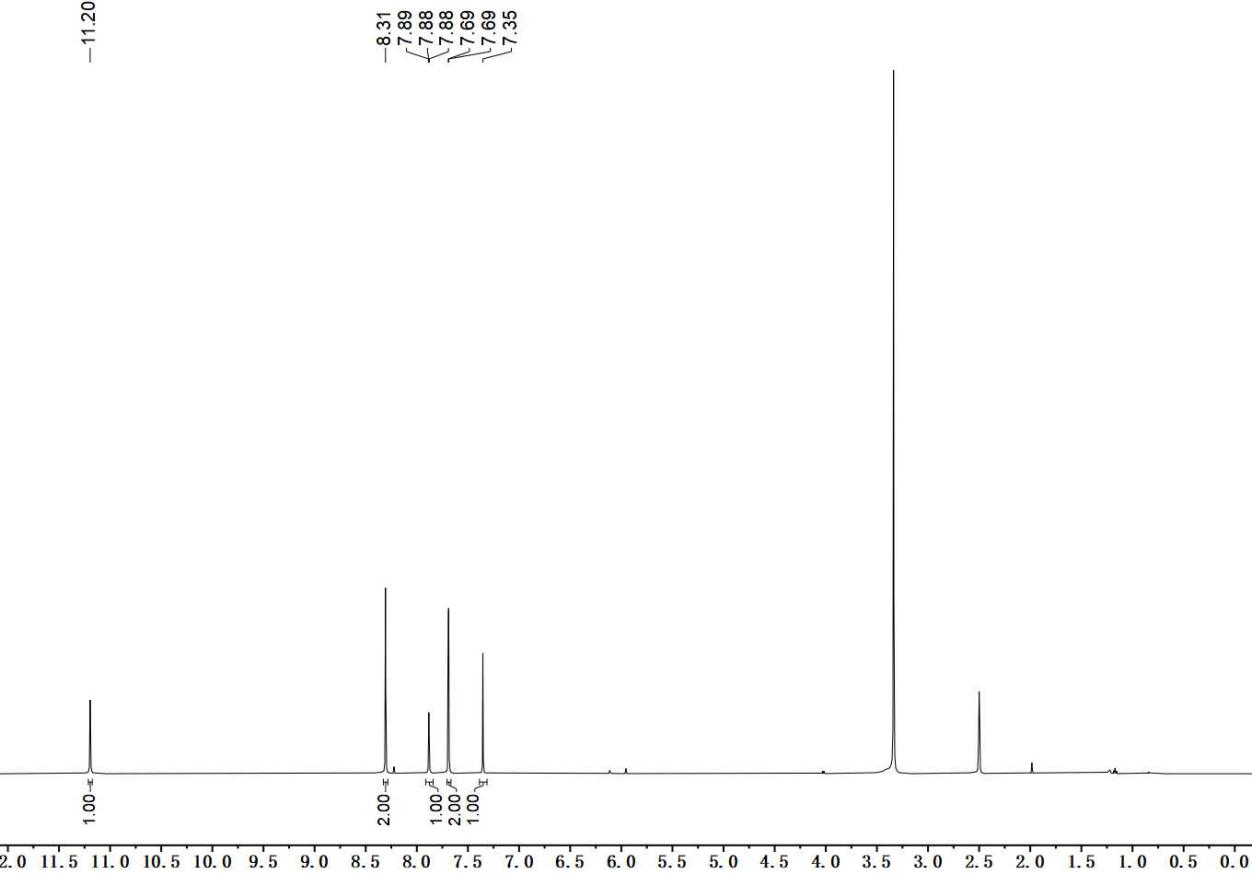
**

^13^C NMR Spectra of **Py-27**

**
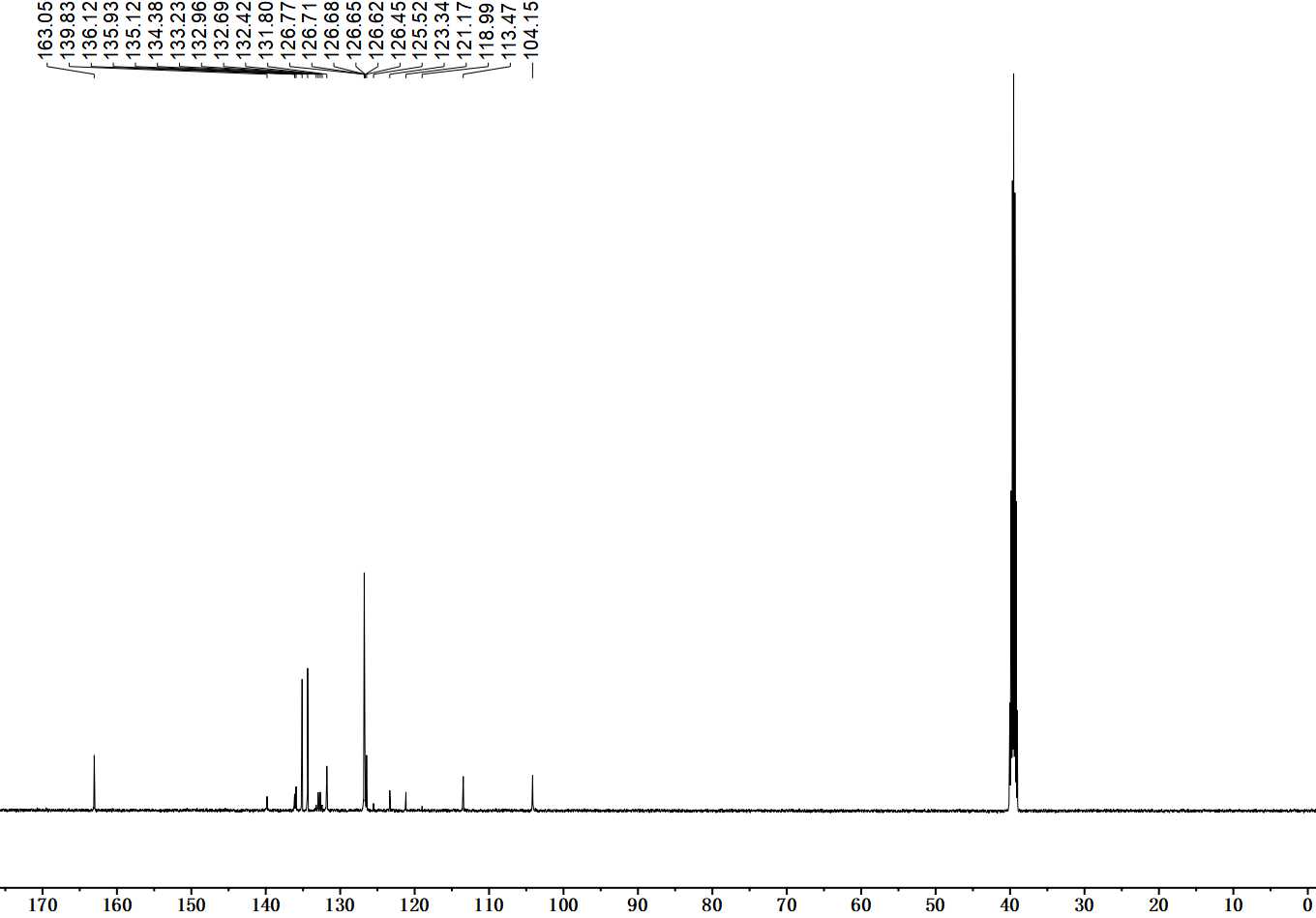
**

^1^H NMR Spectra of **Py-28**

**
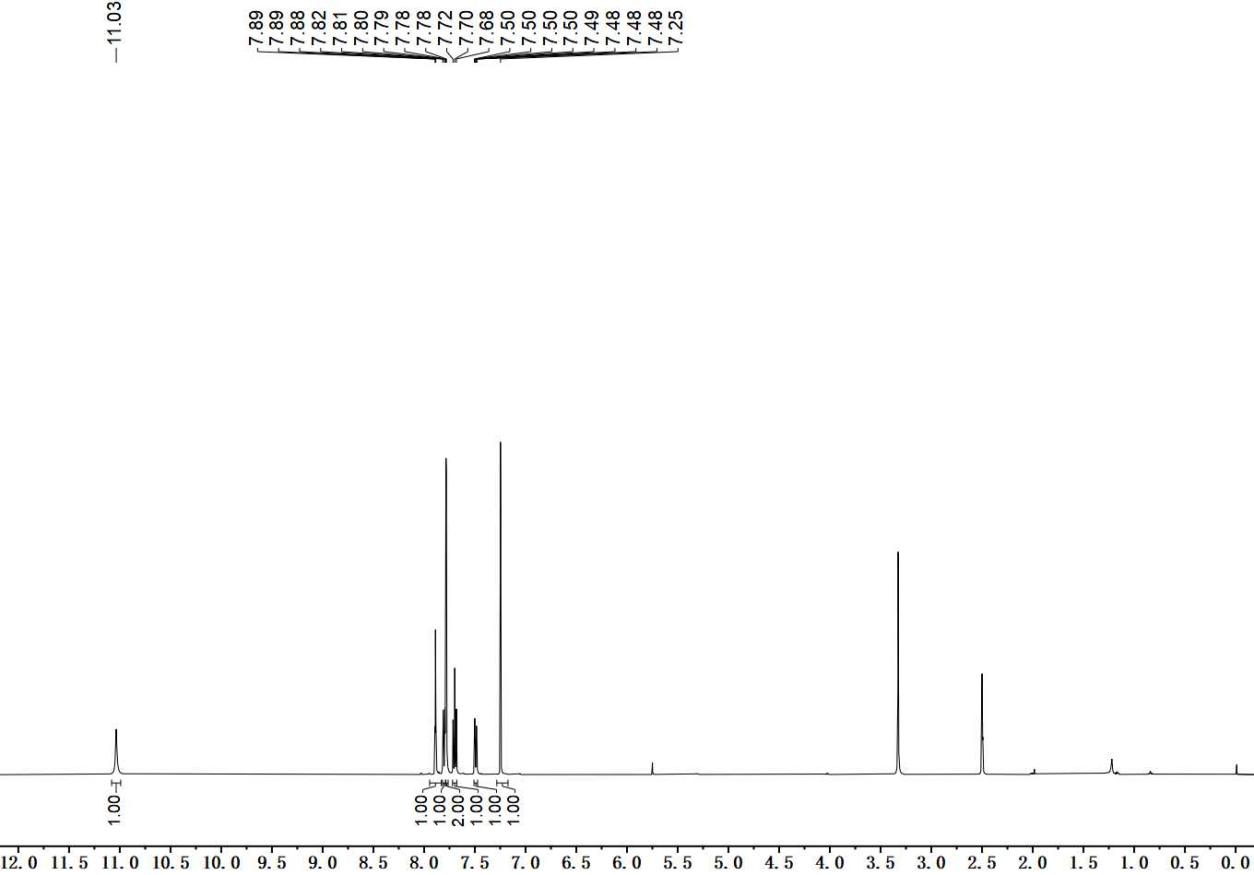
**

^13^C NMR Spectra of **Py-28**

**
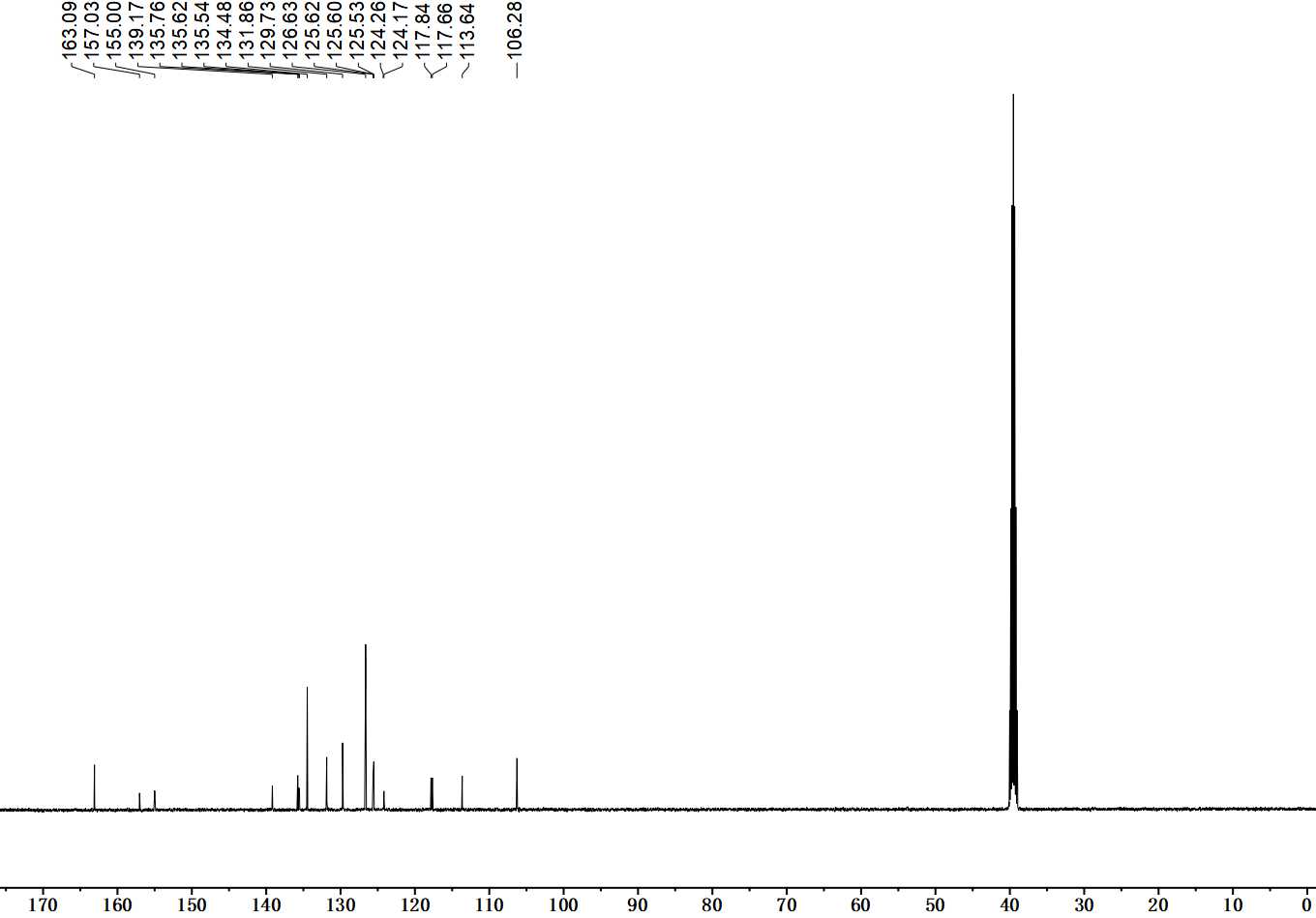
**

^1^H NMR Spectra of **Py-29**

**
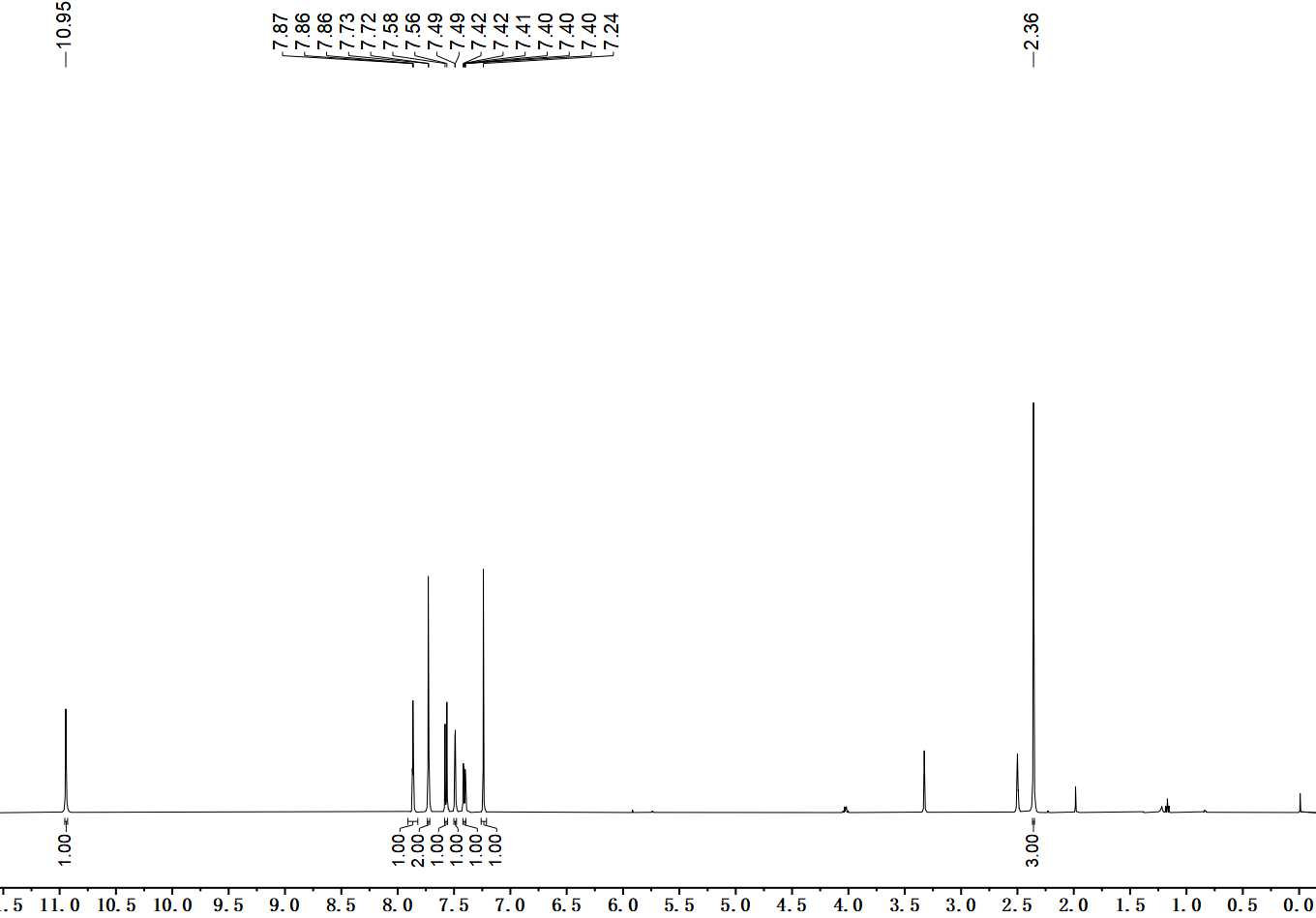
**

^13^C NMR Spectra of **Py-29**

**
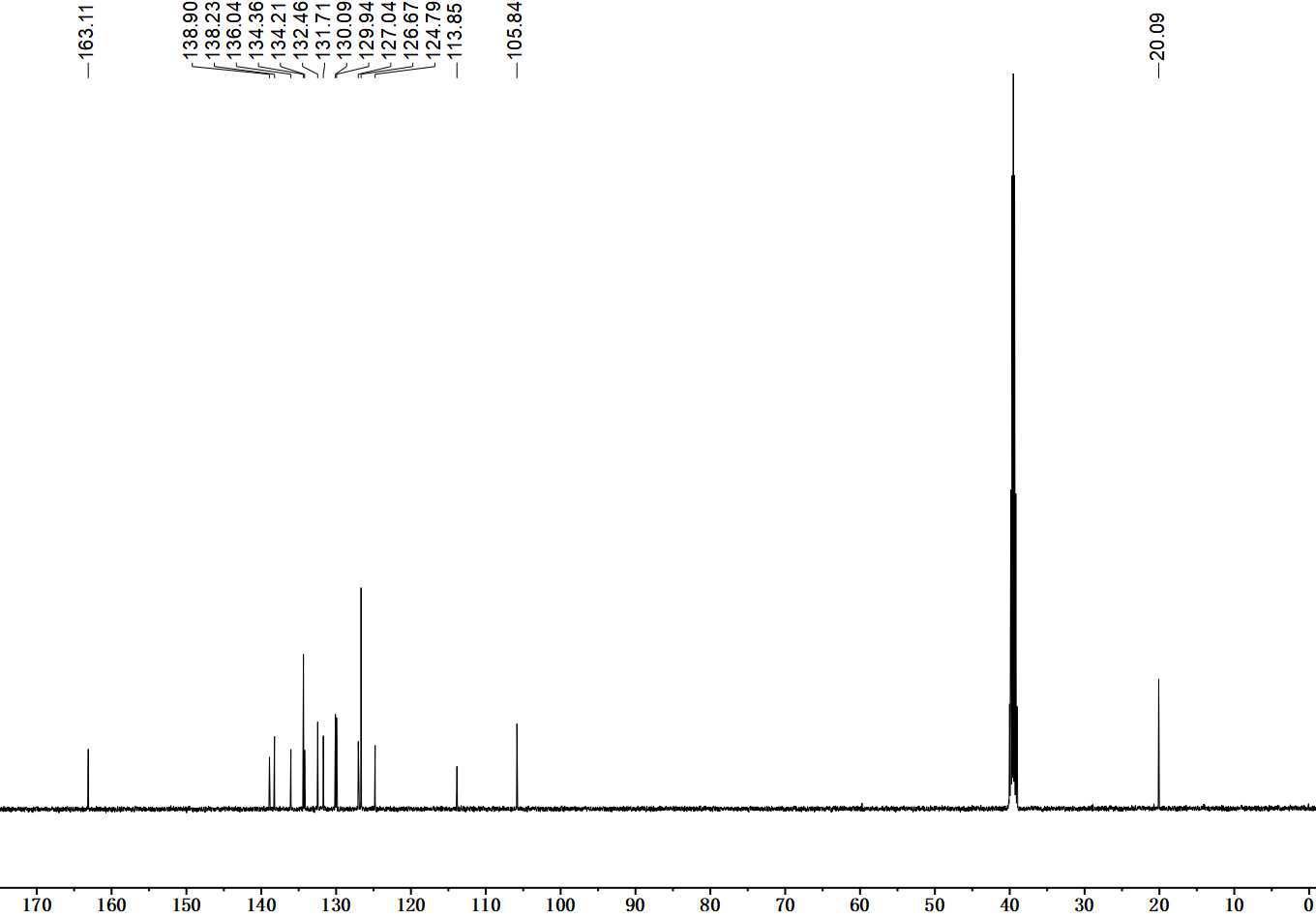
**

^1^H NMR Spectra of **Py-30**

**
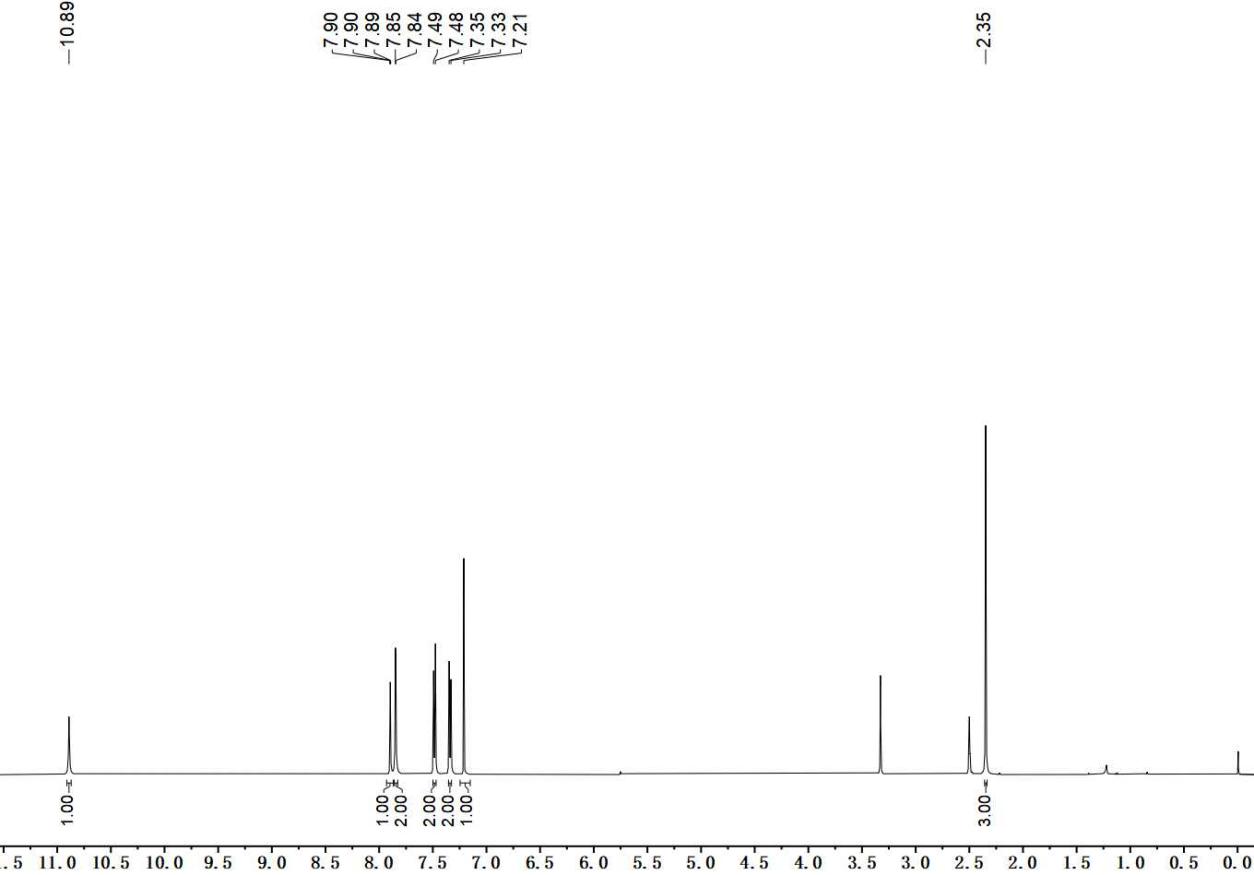
**

^13^C NMR Spectra of **Py-30**

**
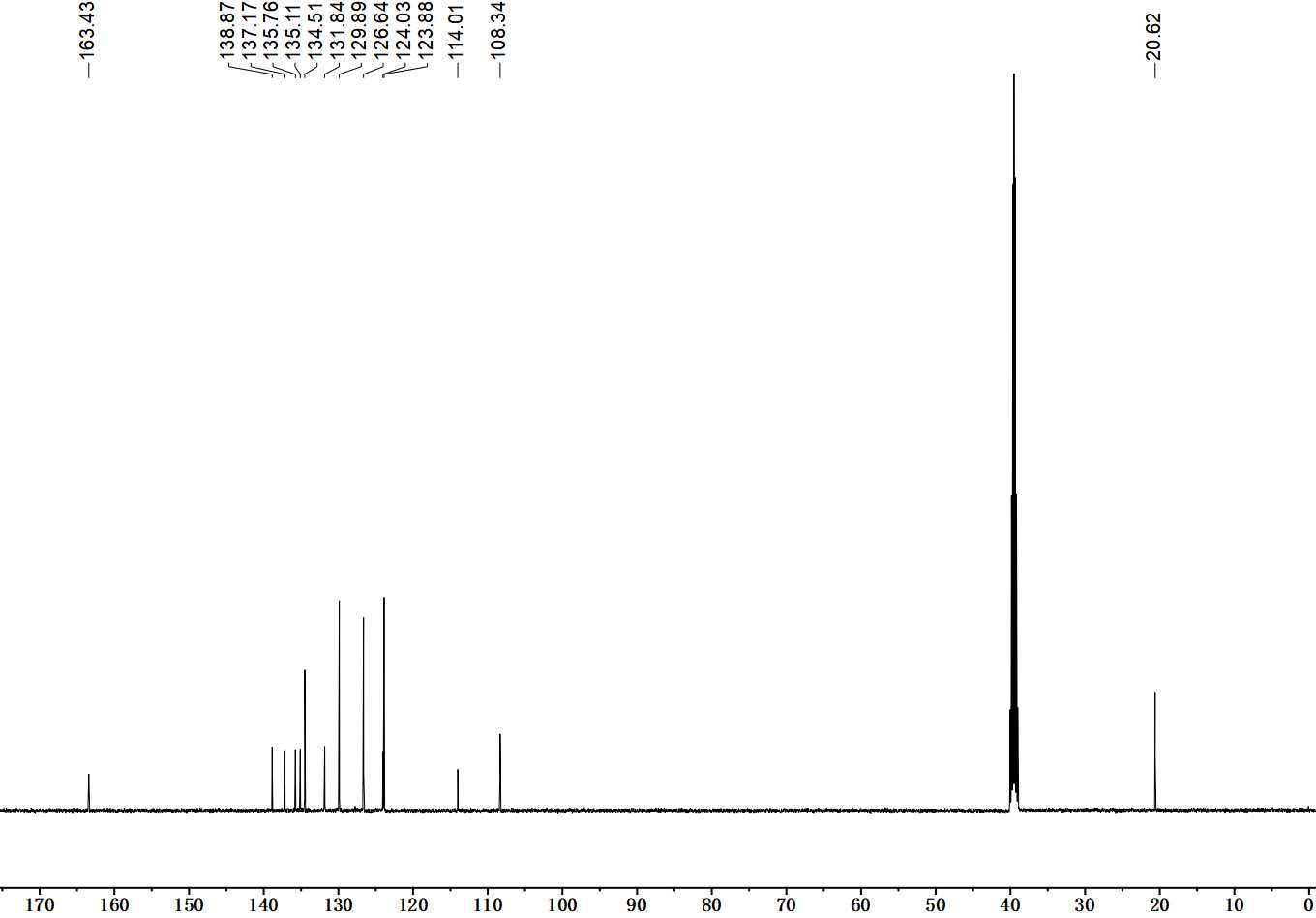
**

^1^H NMR Spectra of **Py-31**

**
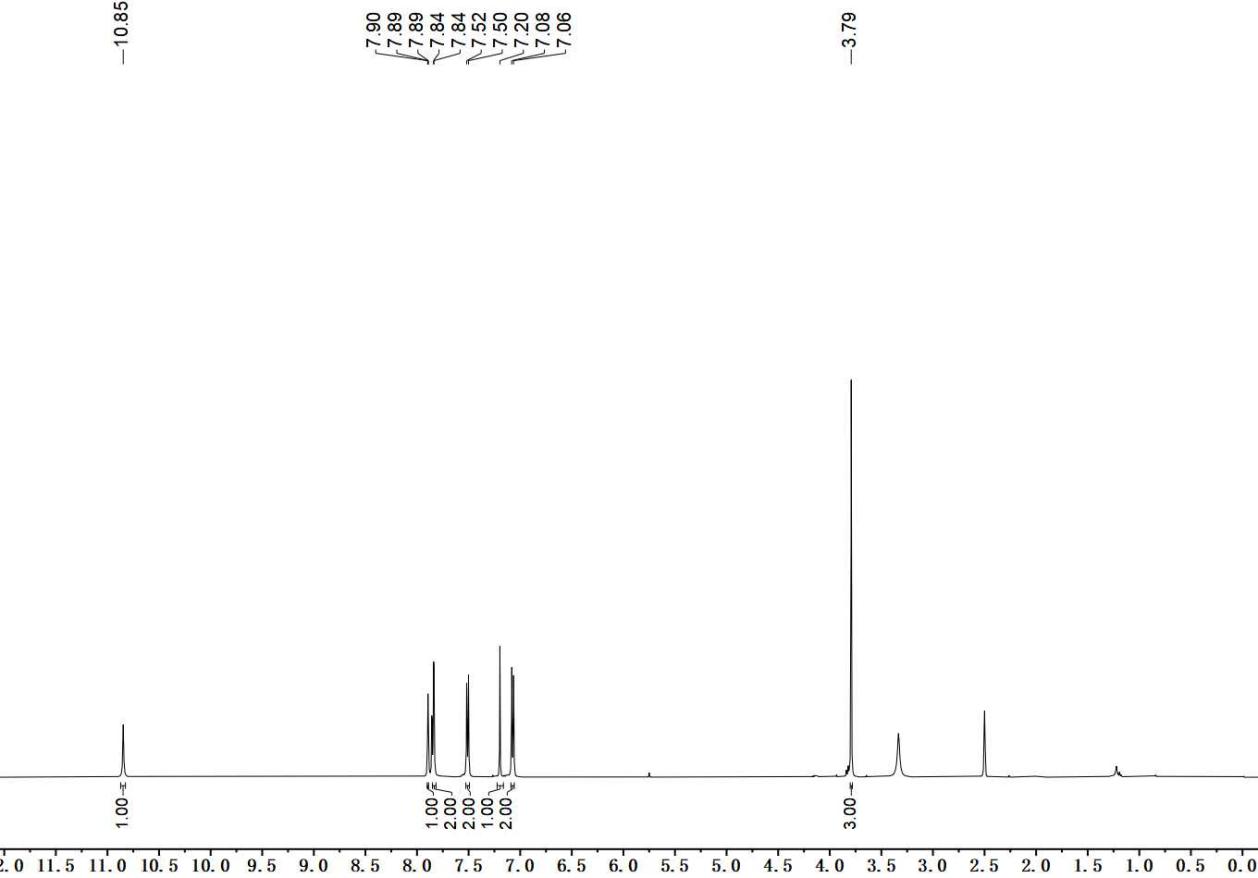
**

^13^C NMR Spectra of **Py-31**

**
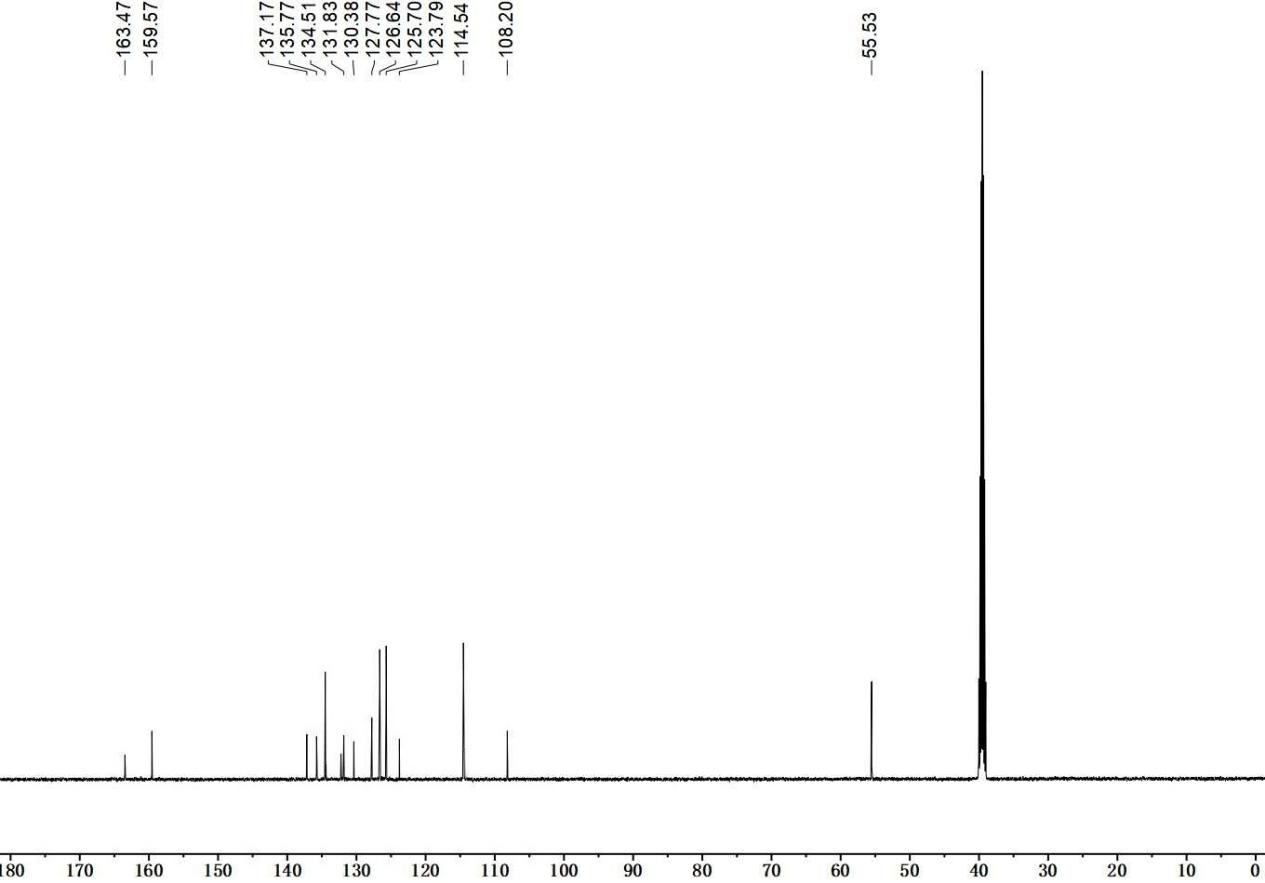
**

^1^H NMR Spectra of **Py-32**

**
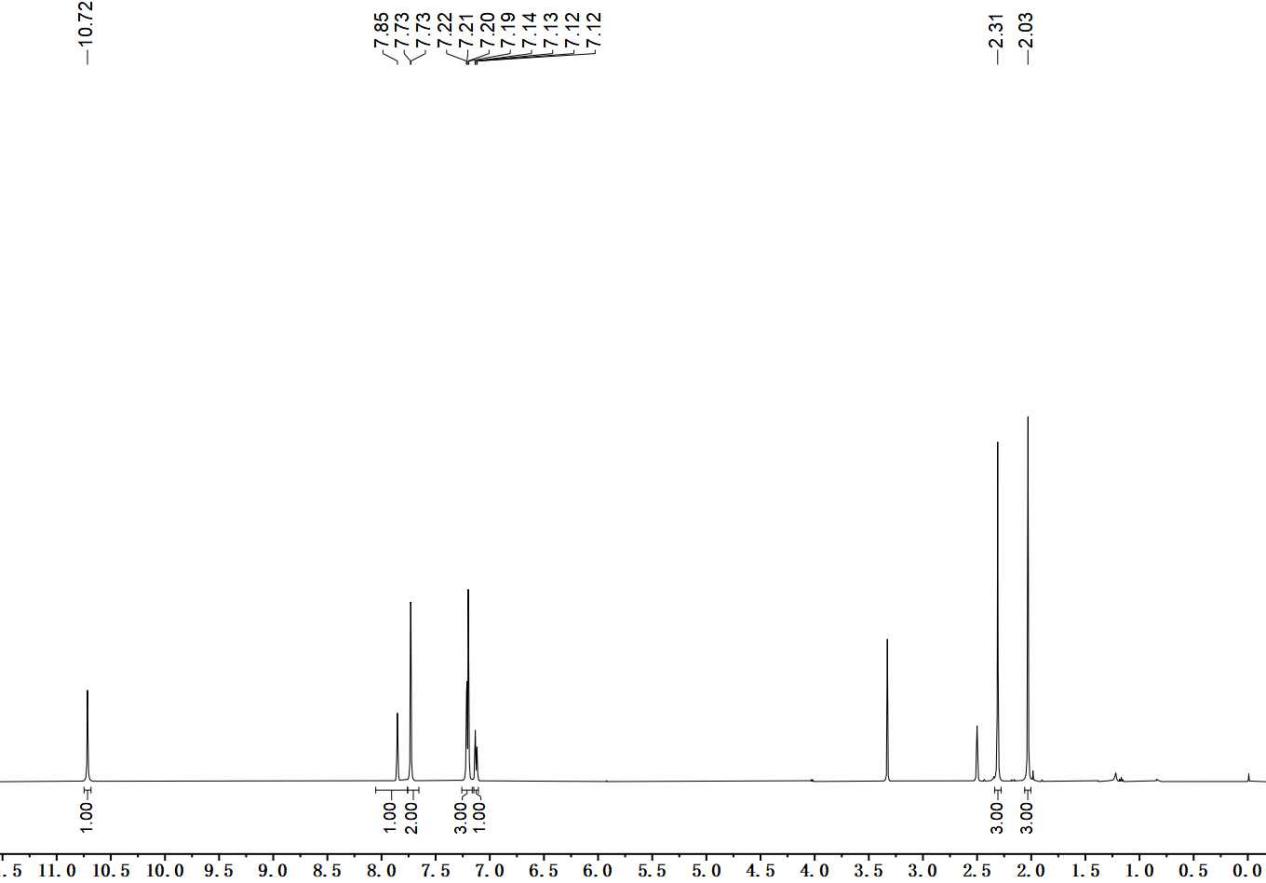
**

^13^C NMR Spectra of **Py-32**

**
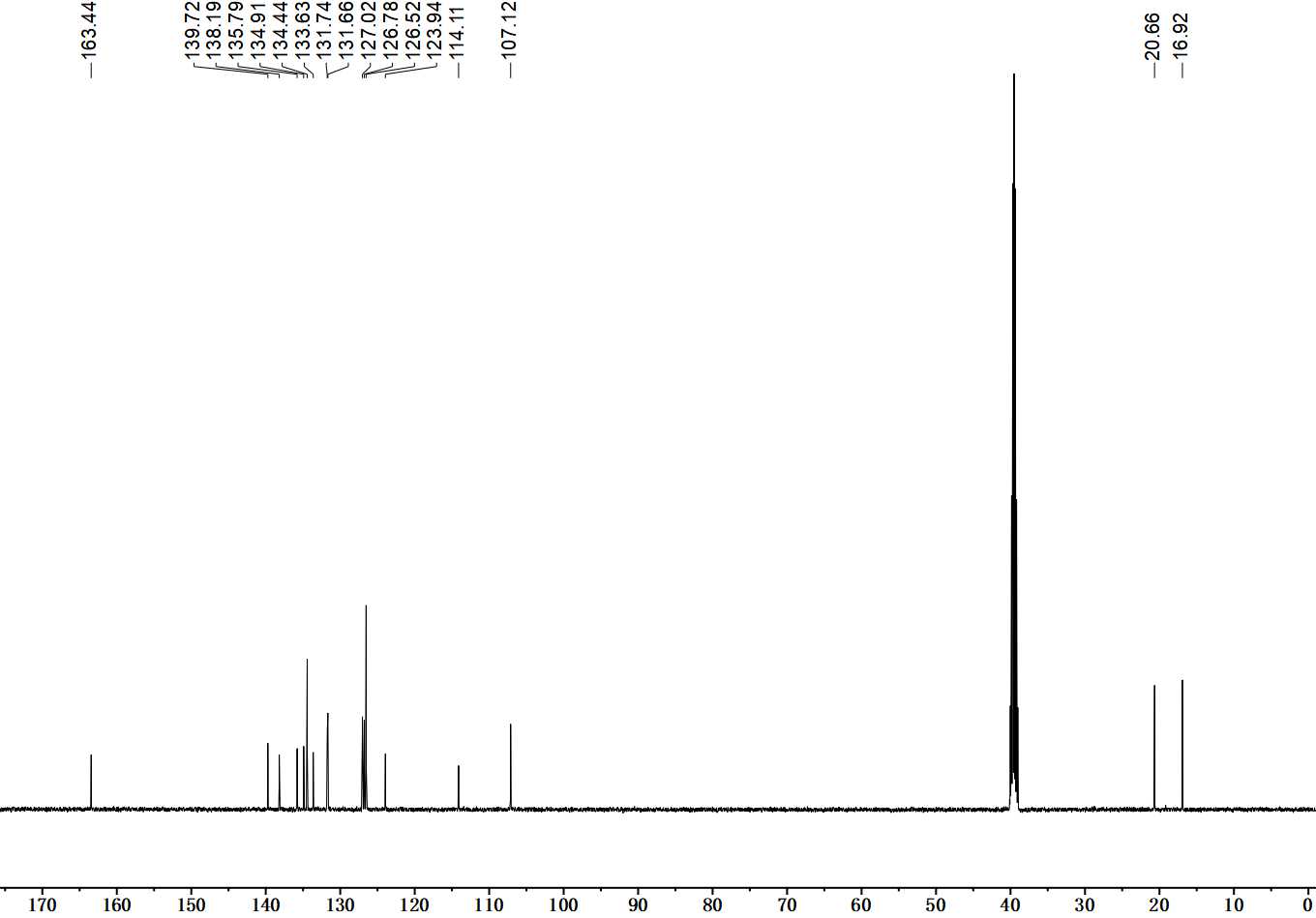
**

^1^H NMR Spectra of **Py-33**

**
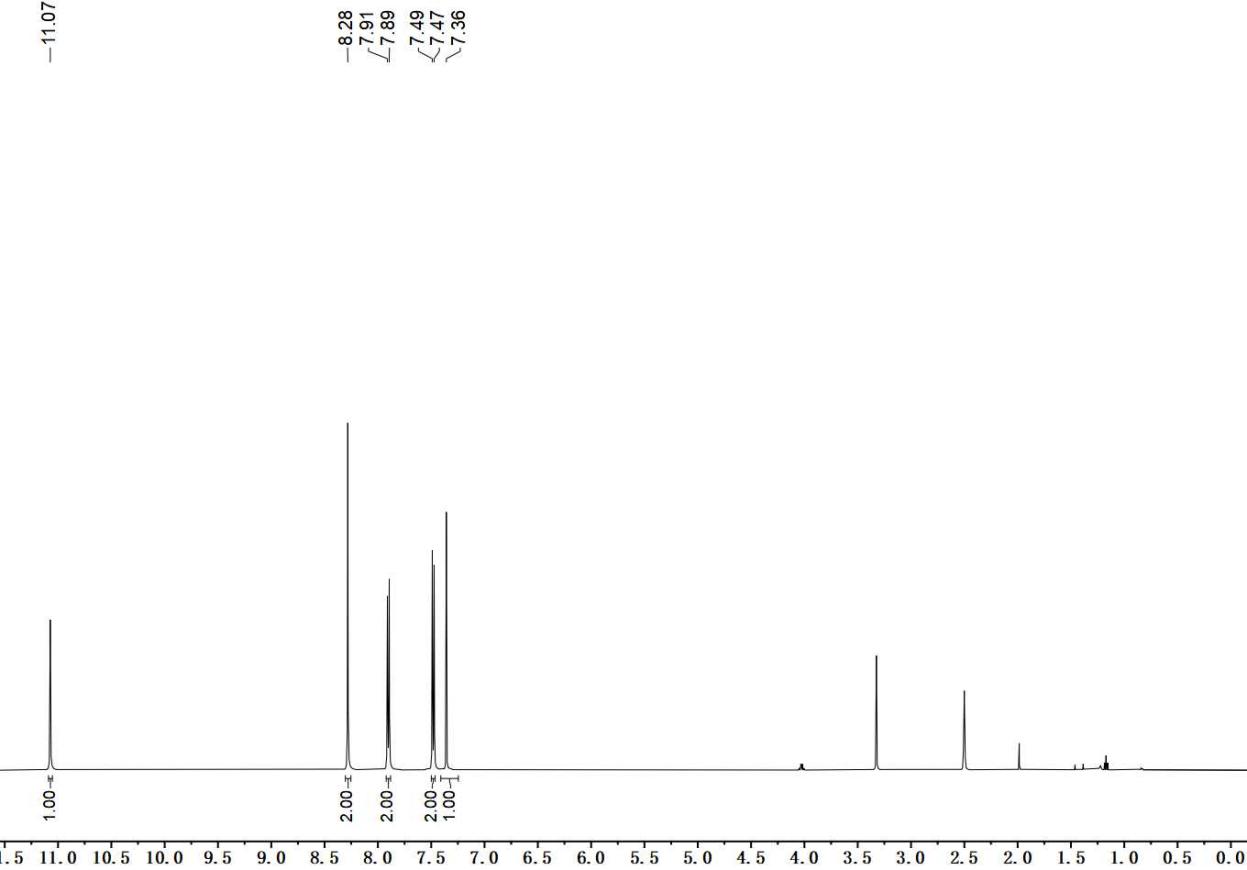
**

^13^C NMR Spectra of **Py-33**

**
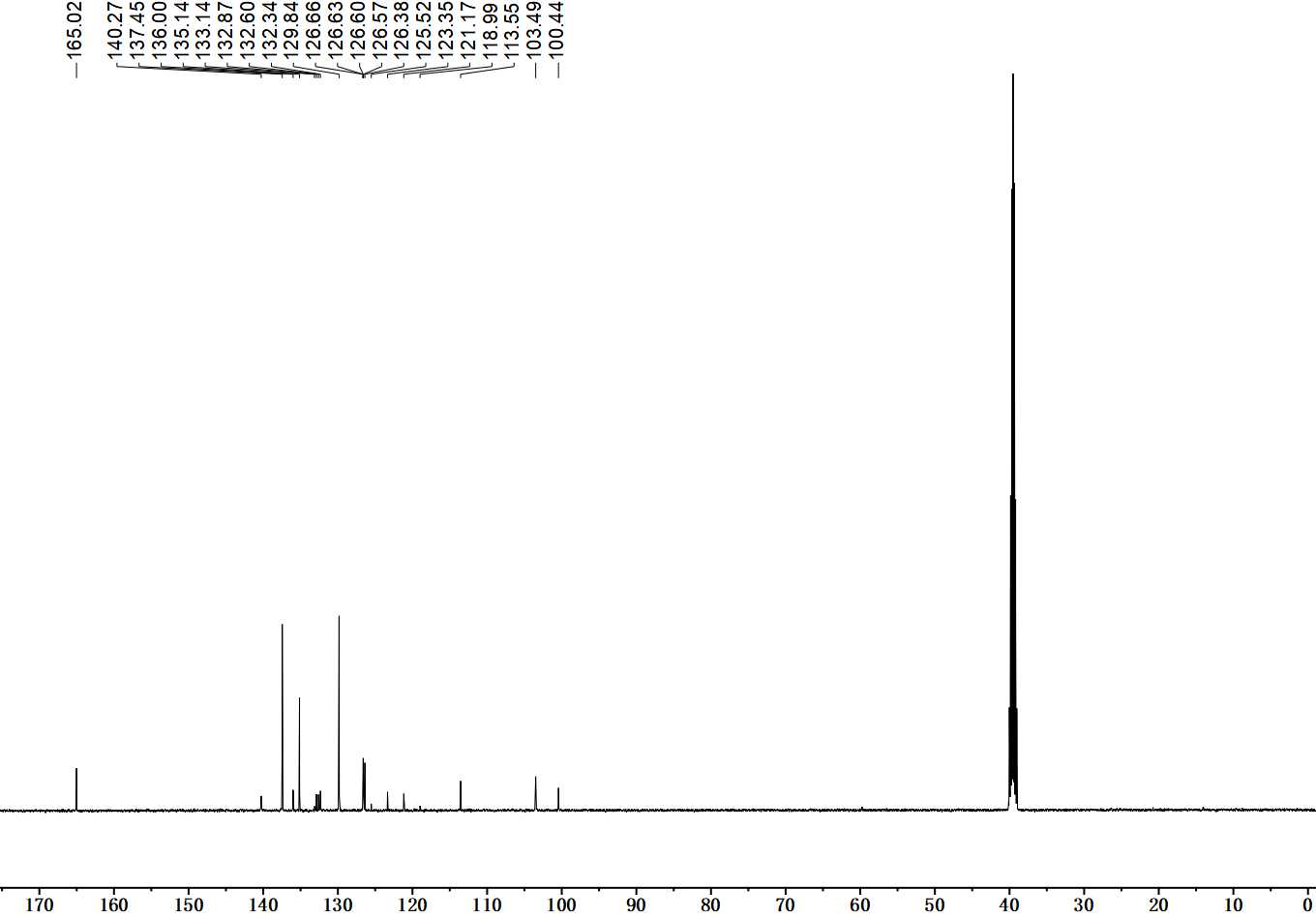
**

^1^H NMR Spectra of **Py-34**

**
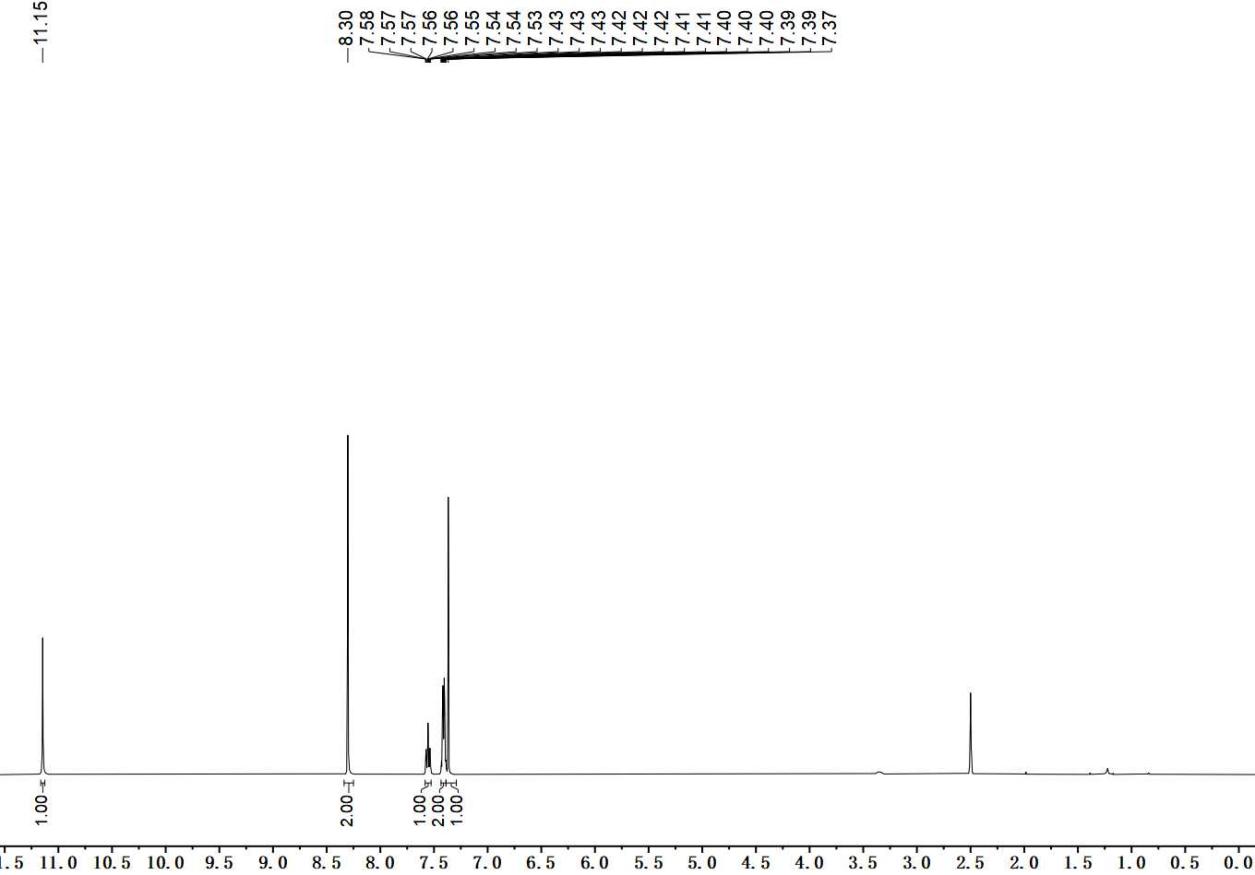
**

^13^C NMR Spectra of **Py-34**

**
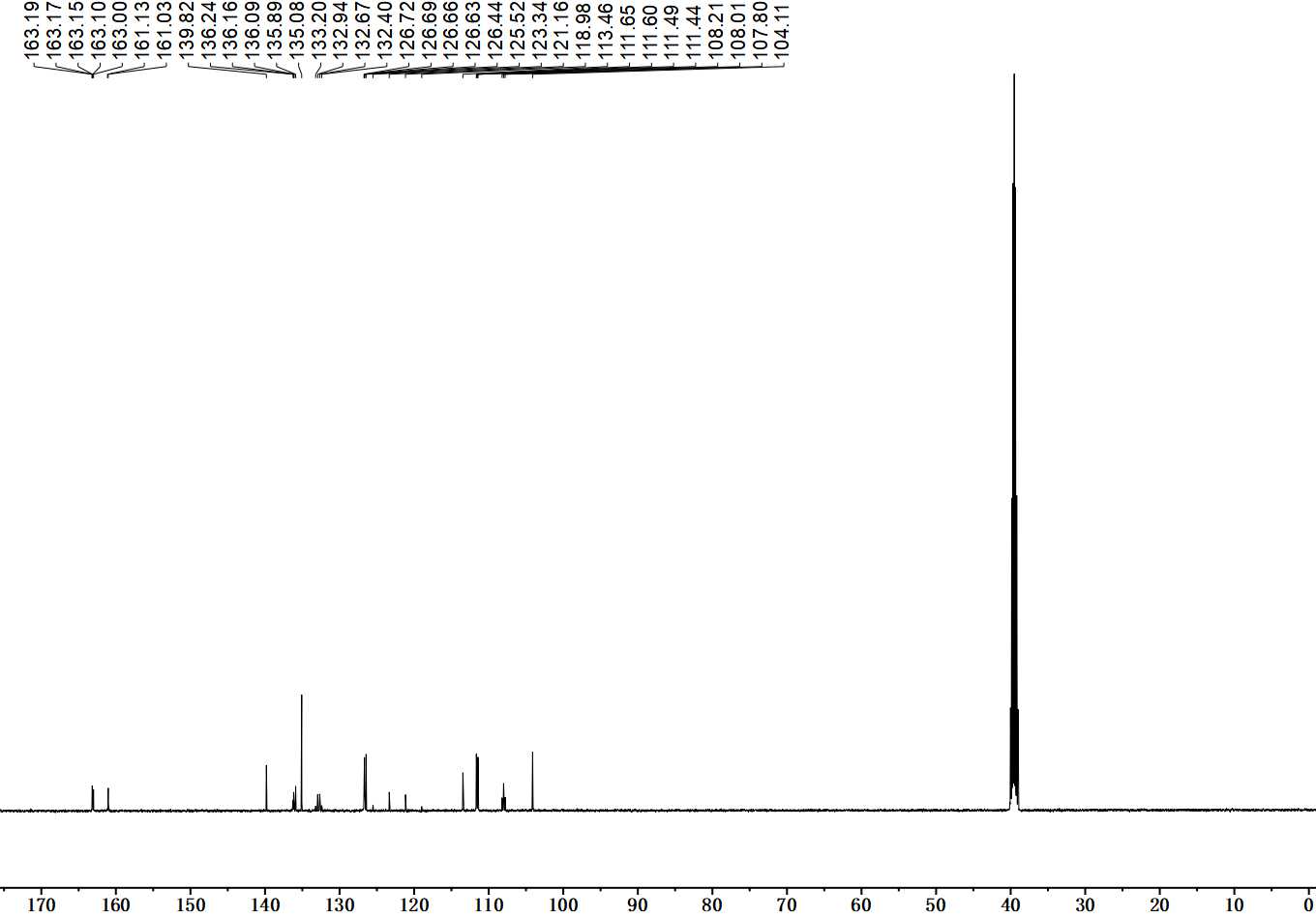
**

^1^H NMR Spectra of **Py-35**

**
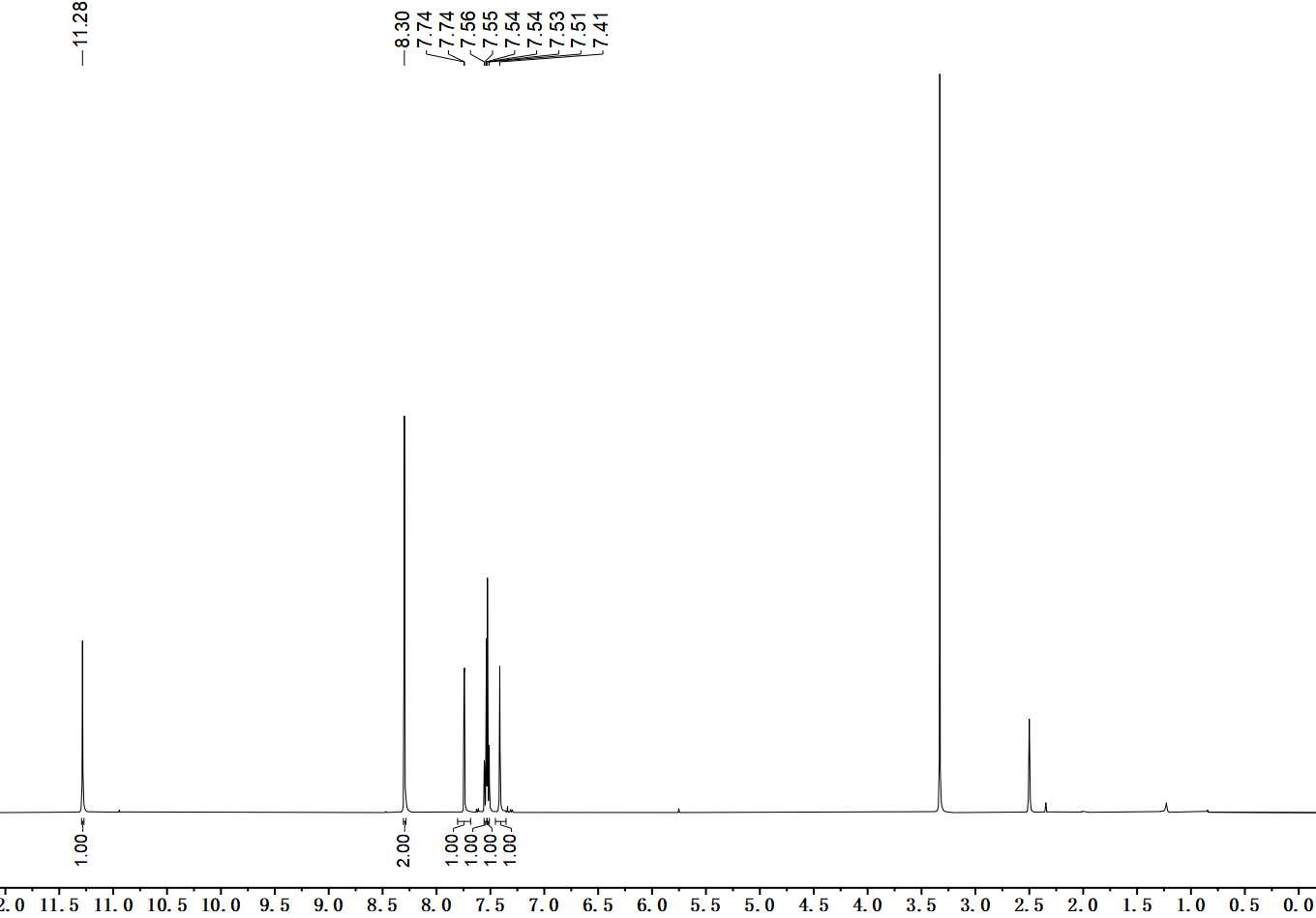
**

^13^C NMR Spectra of **Py-35**

**
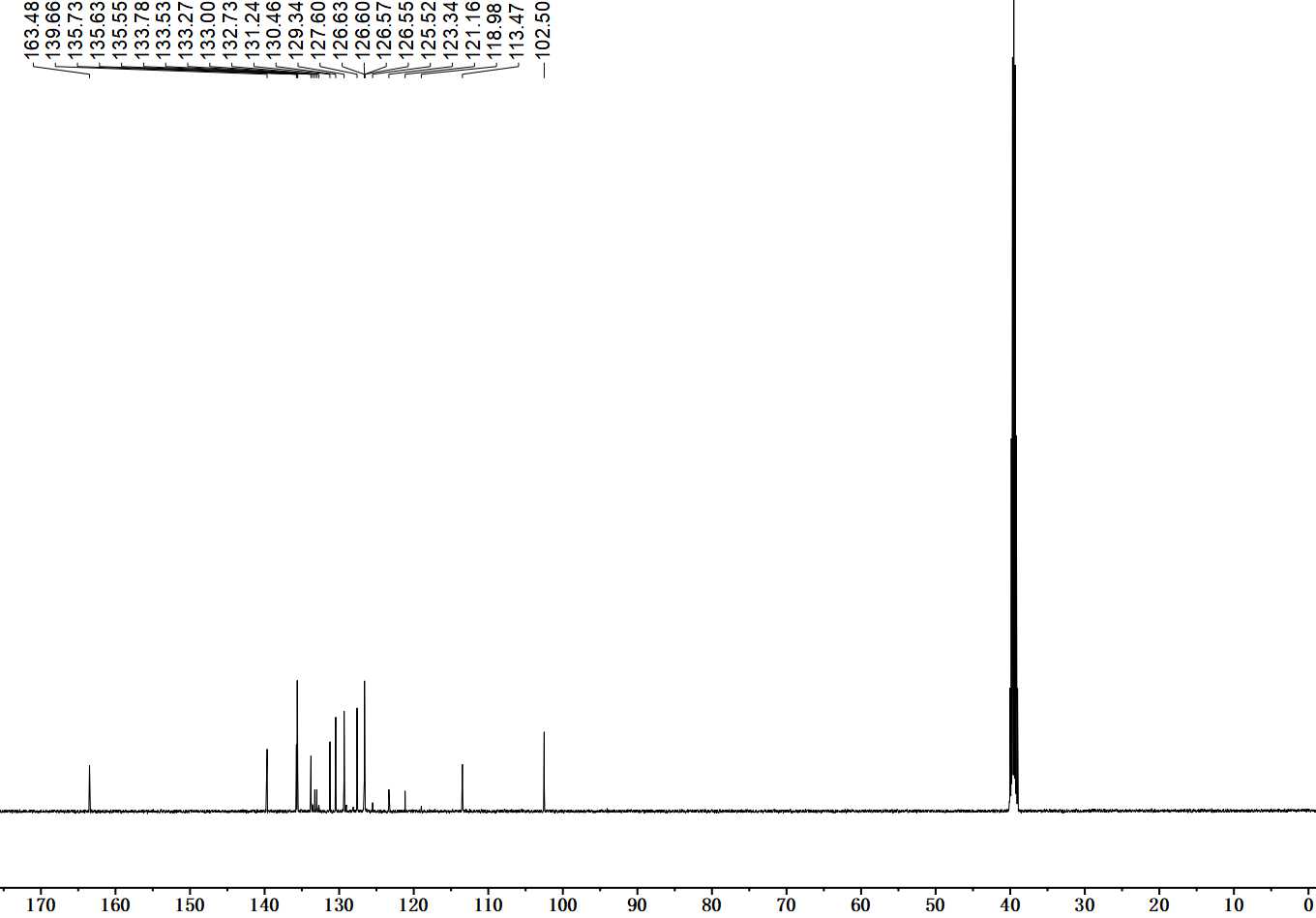
**

^1^H NMR Spectra of **Py-36**

**
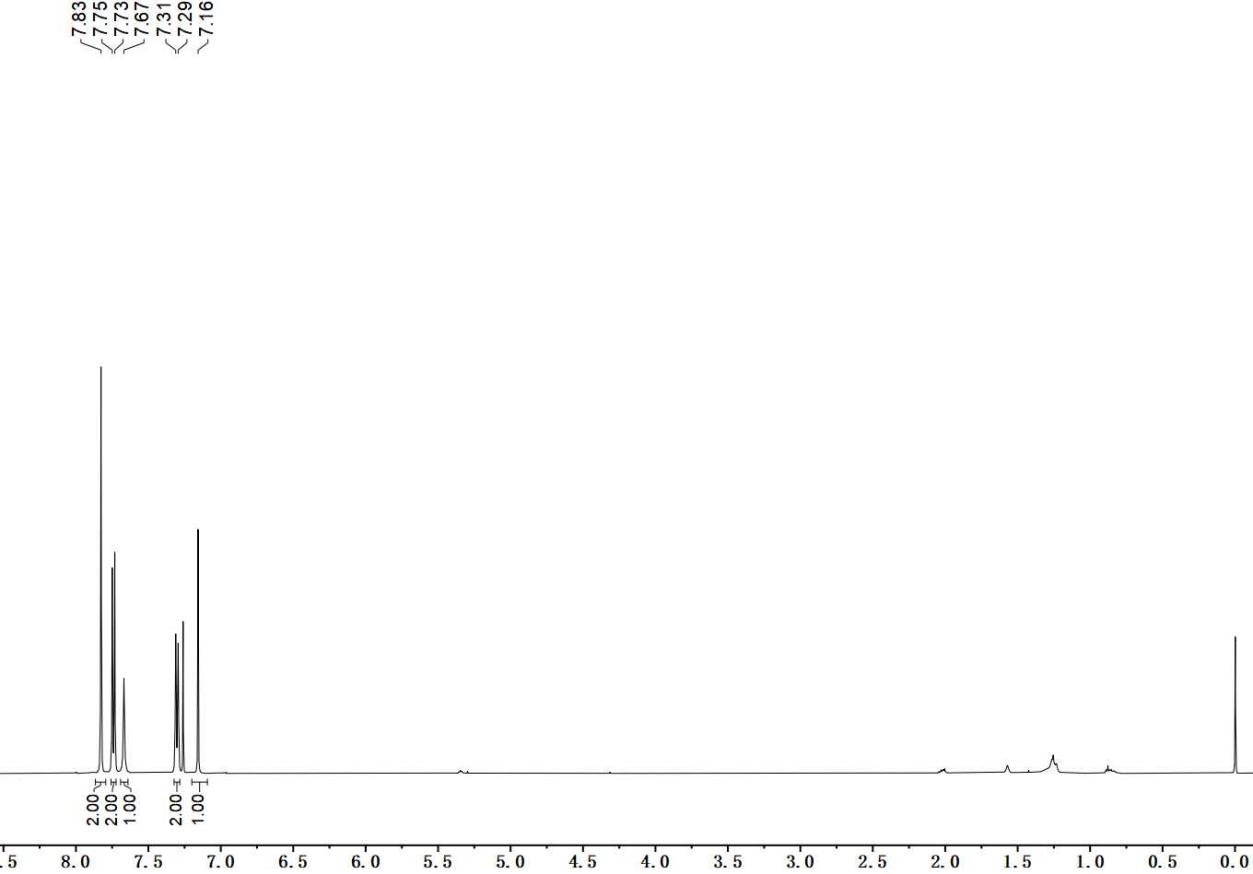
**

^13^C NMR Spectra of **Py-36**

**
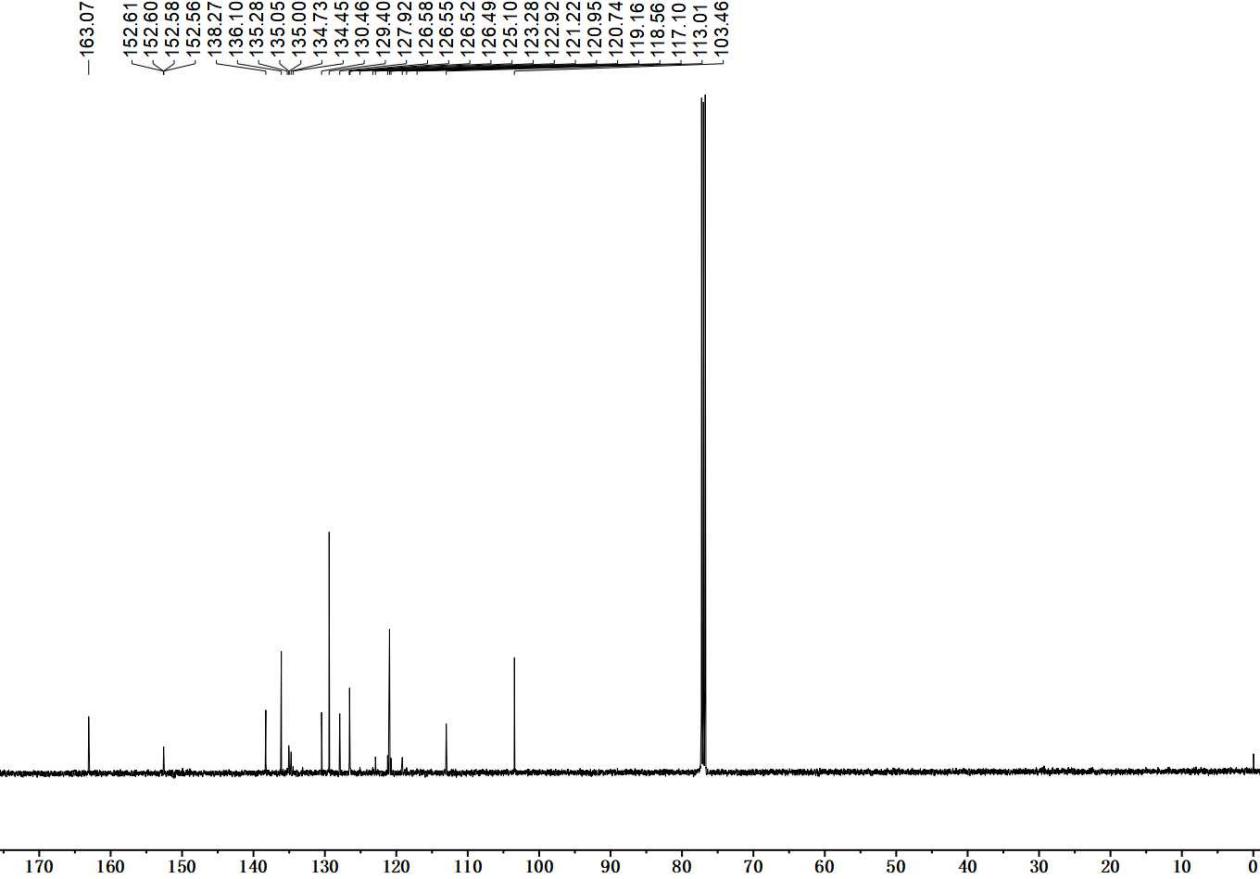
**

^1^H NMR Spectra of **Py-37**

**
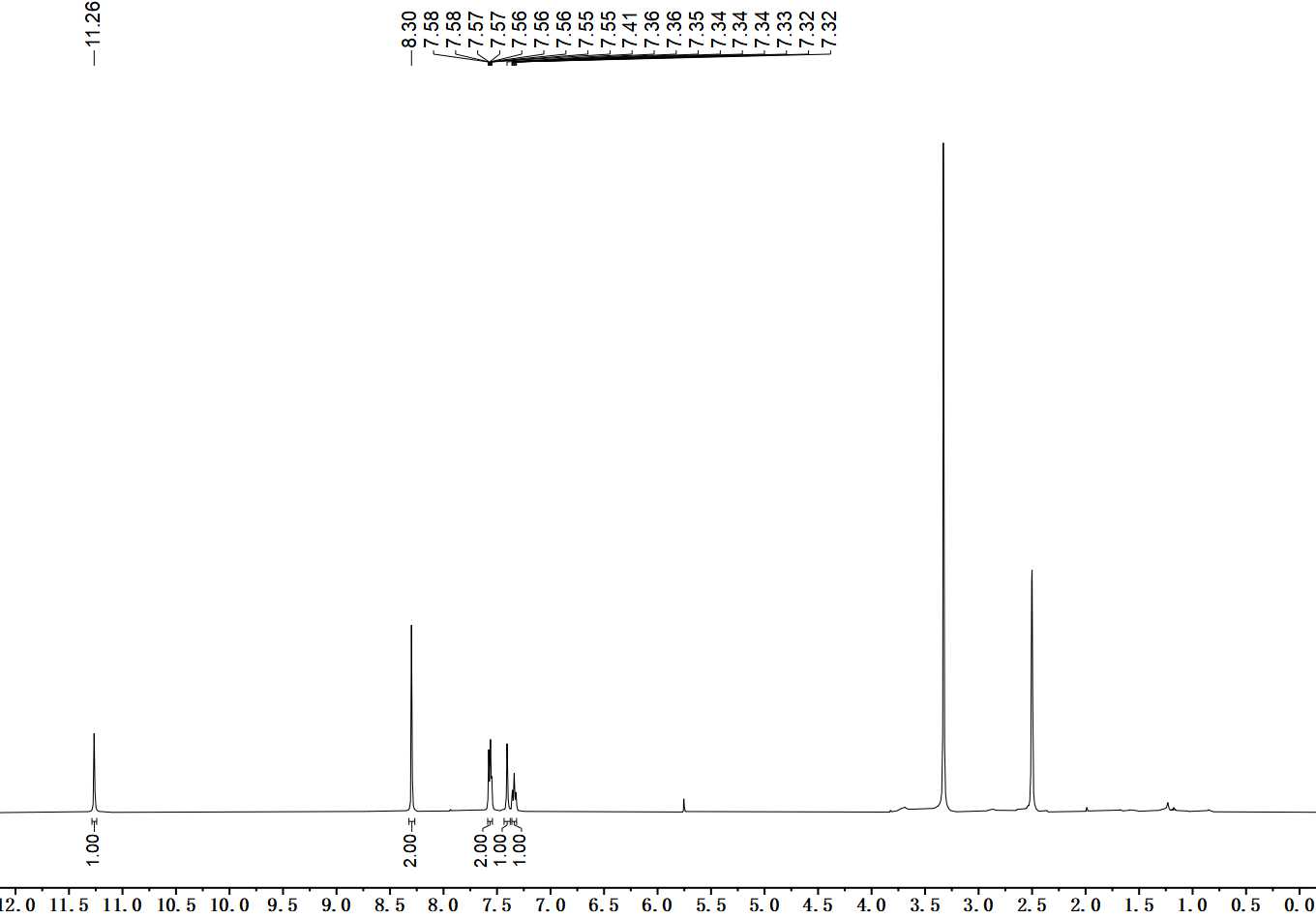
**

^13^C NMR Spectra of **Py-37**

**
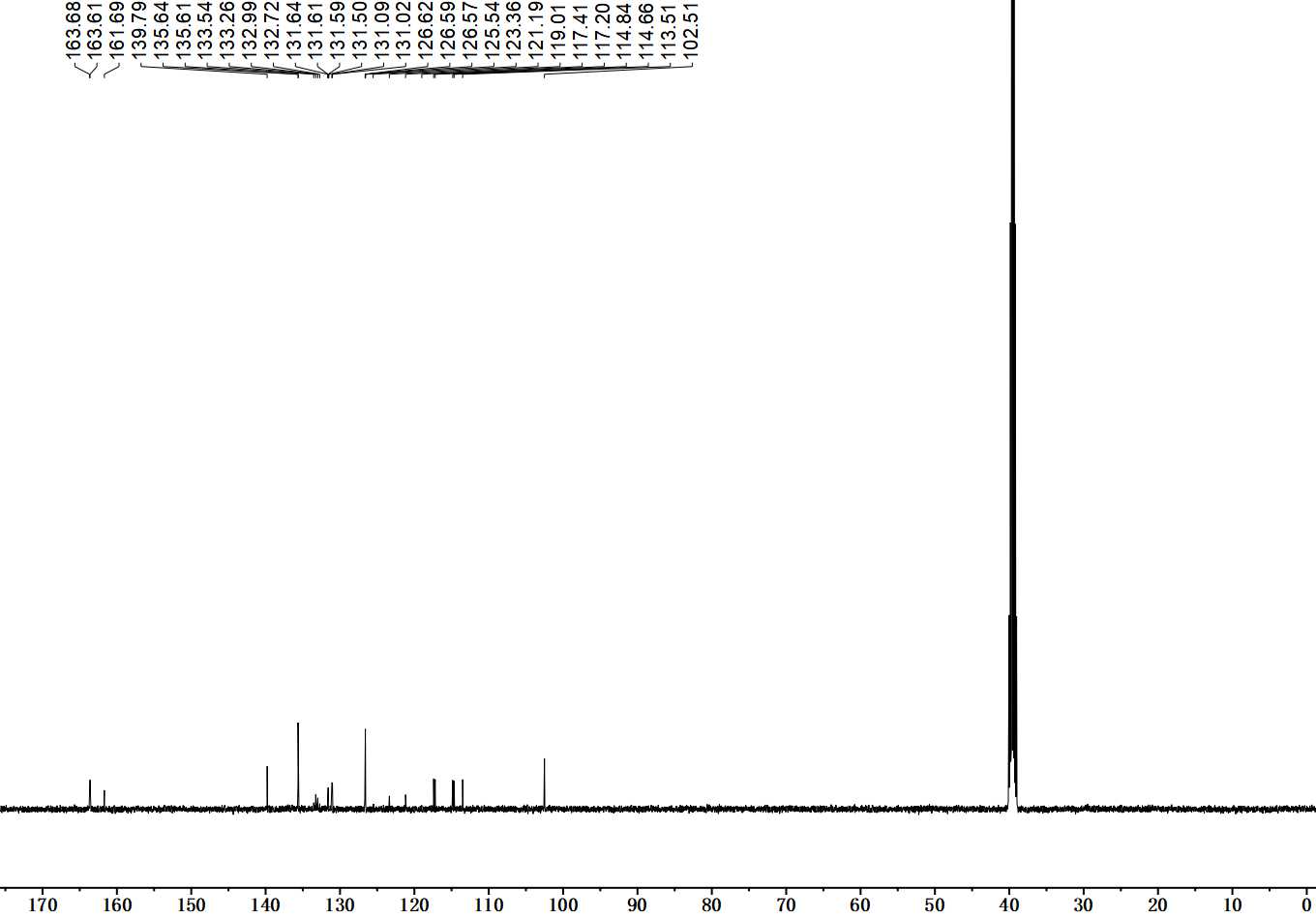
**

^1^H NMR Spectra of **Py-38**

**
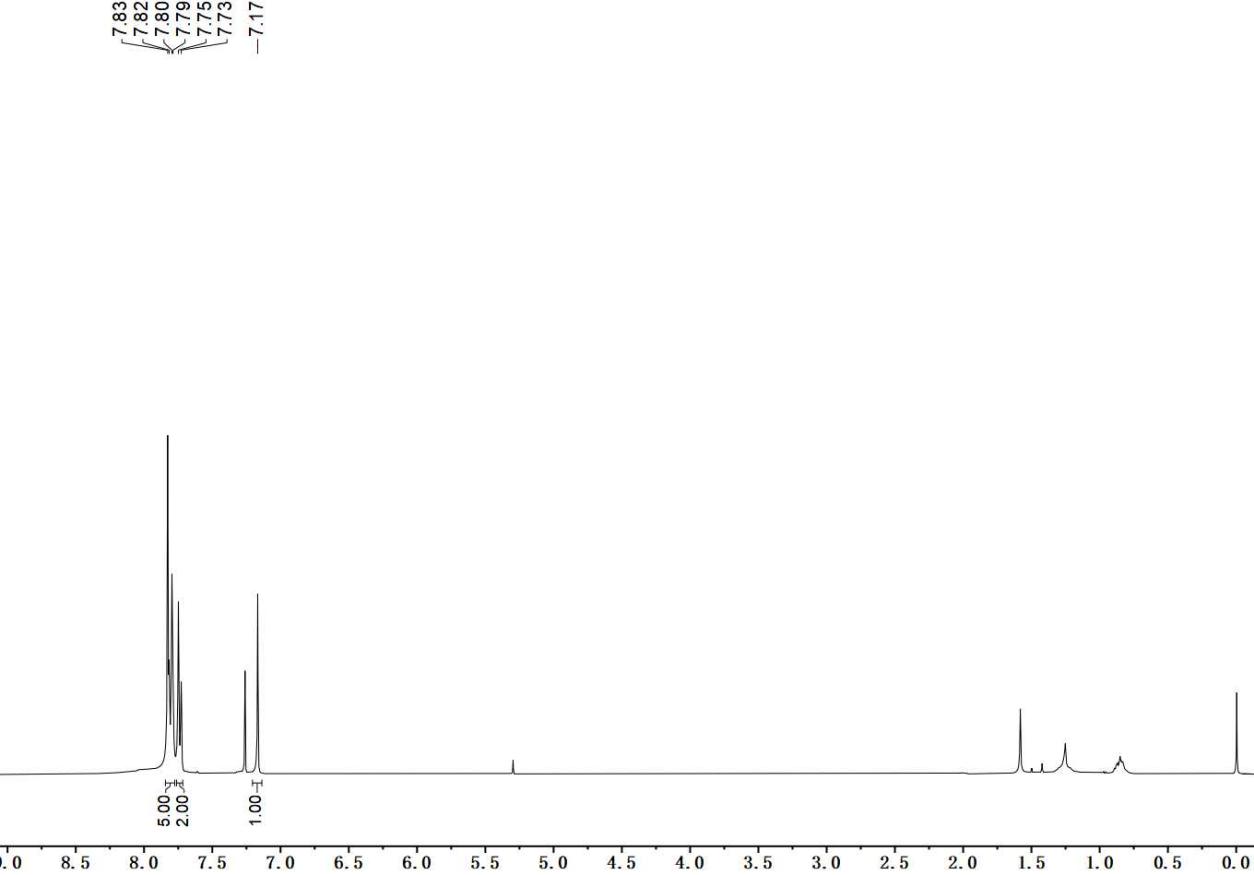
**

^13^C NMR Spectra of **Py-38**

**
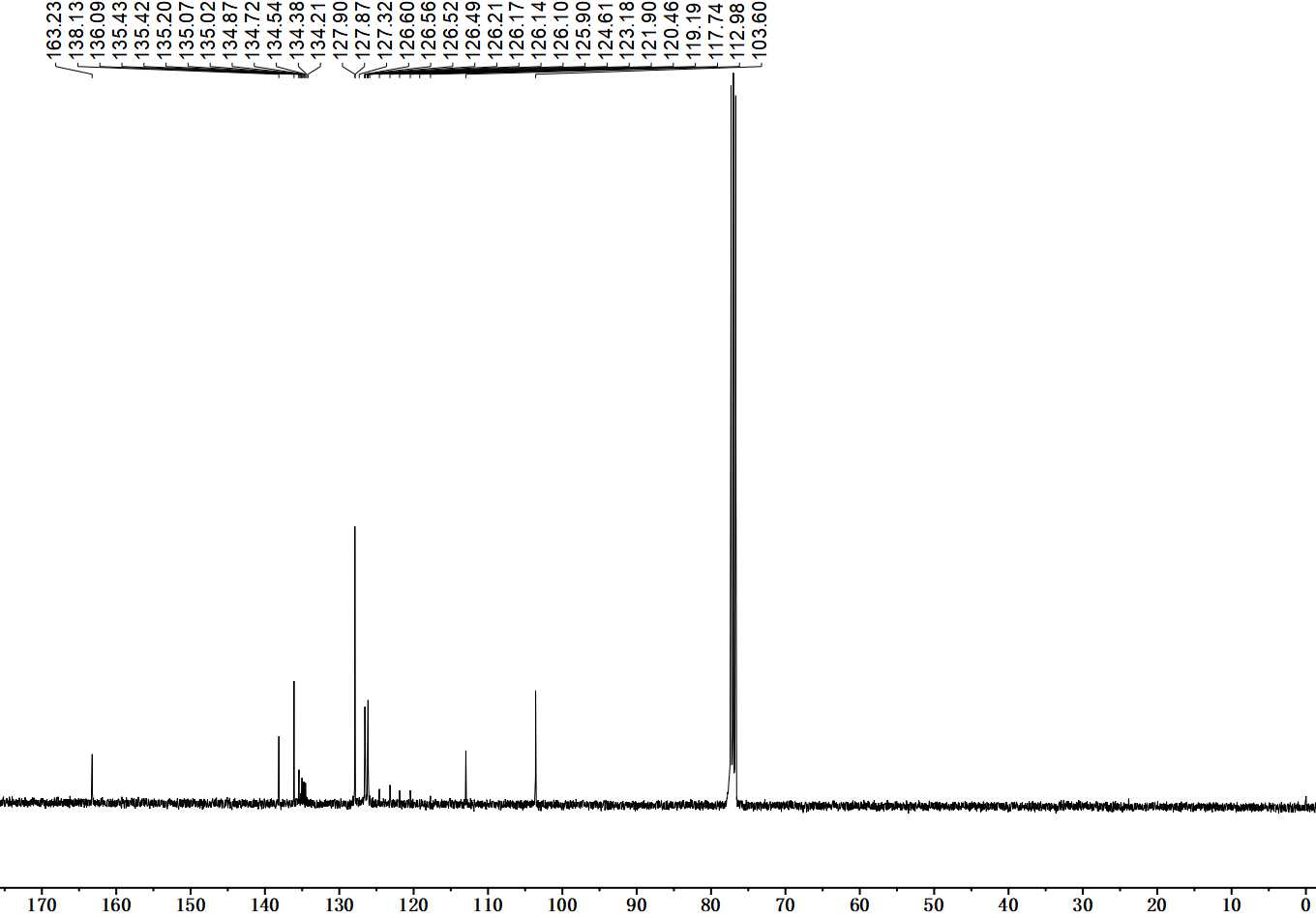
**

^1^H NMR Spectra of **Py-39**

**
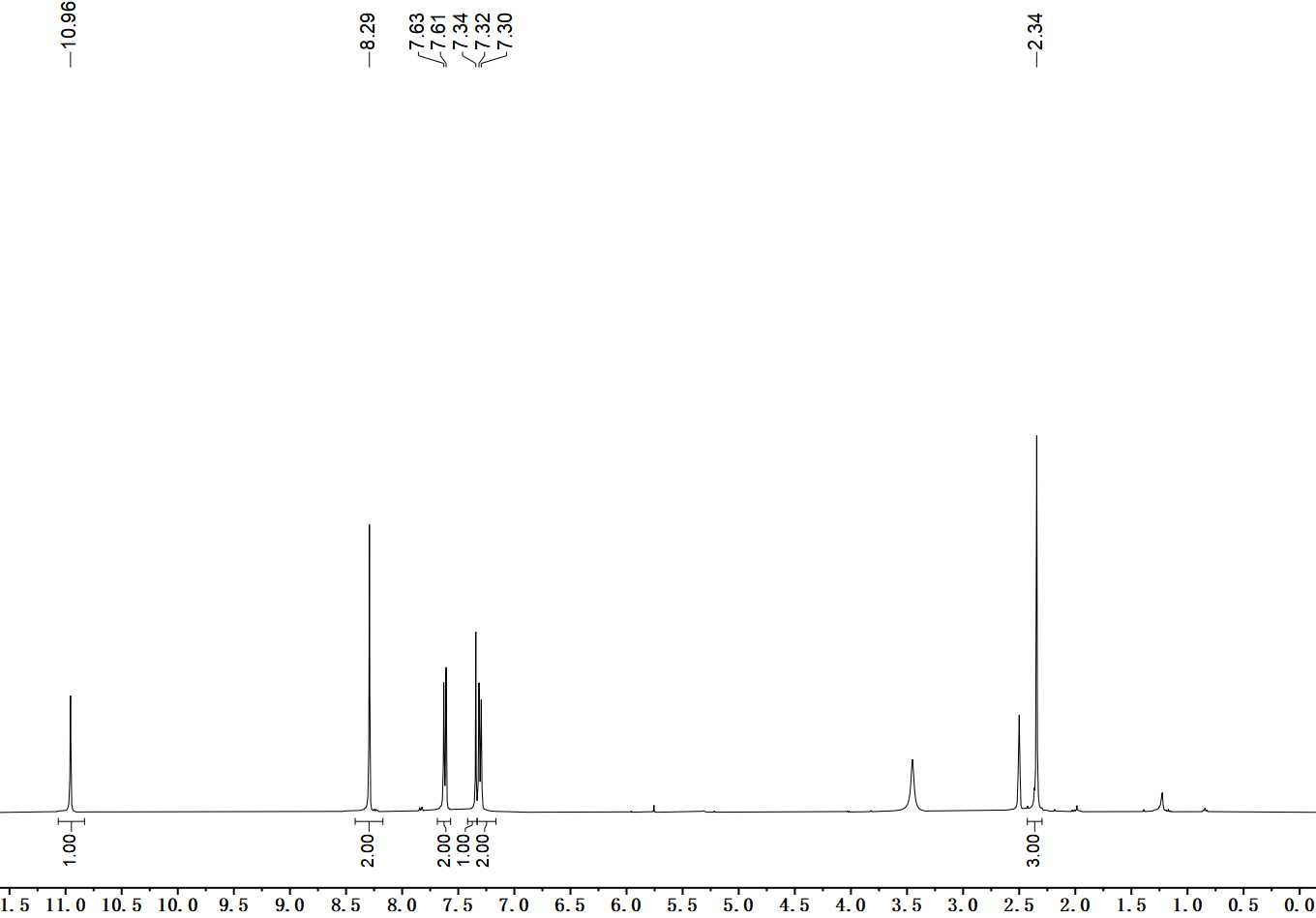
**

^13^C NMR Spectra of **Py-39**

**
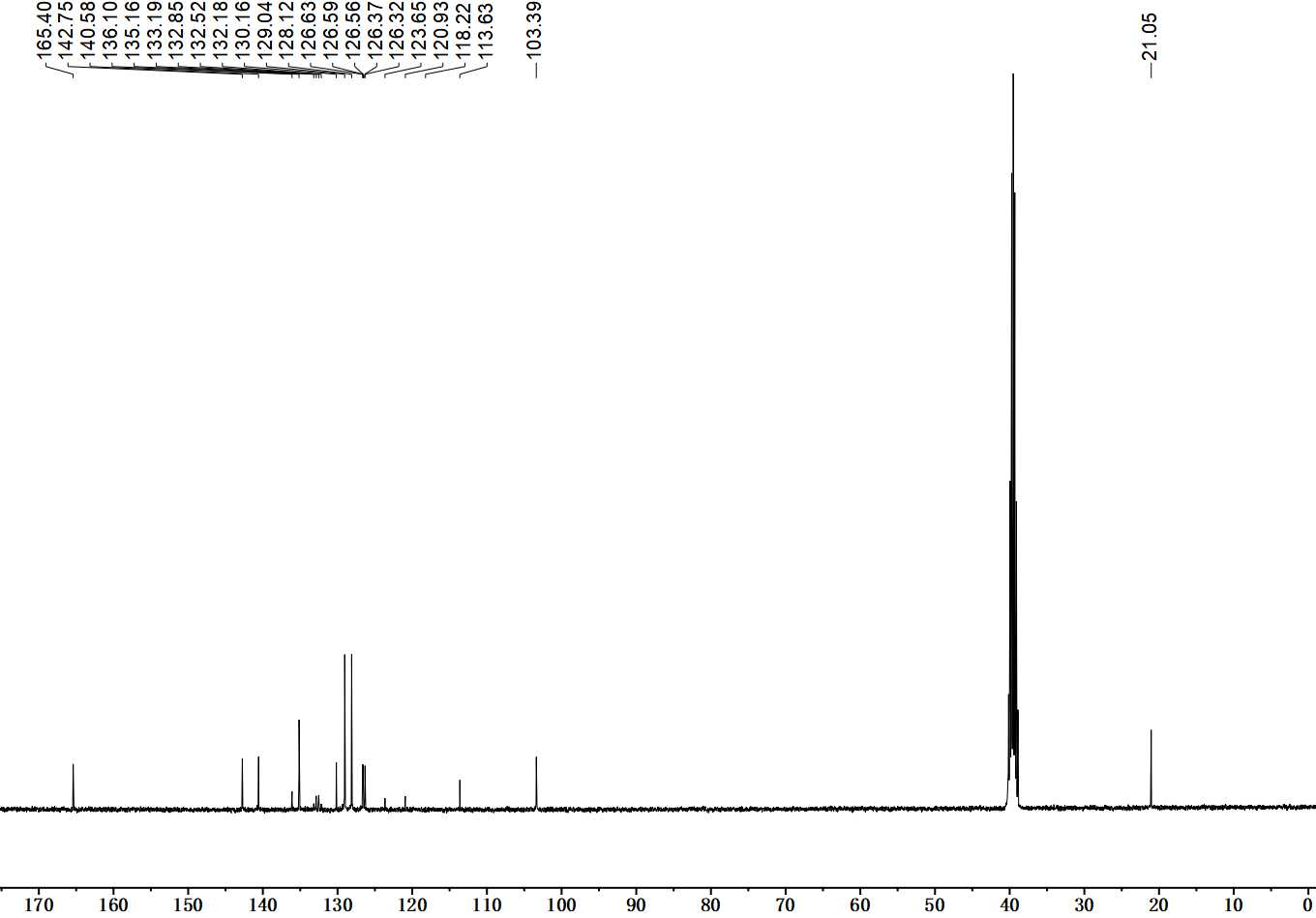
**

^1^H NMR Spectra of **Py-40**

**
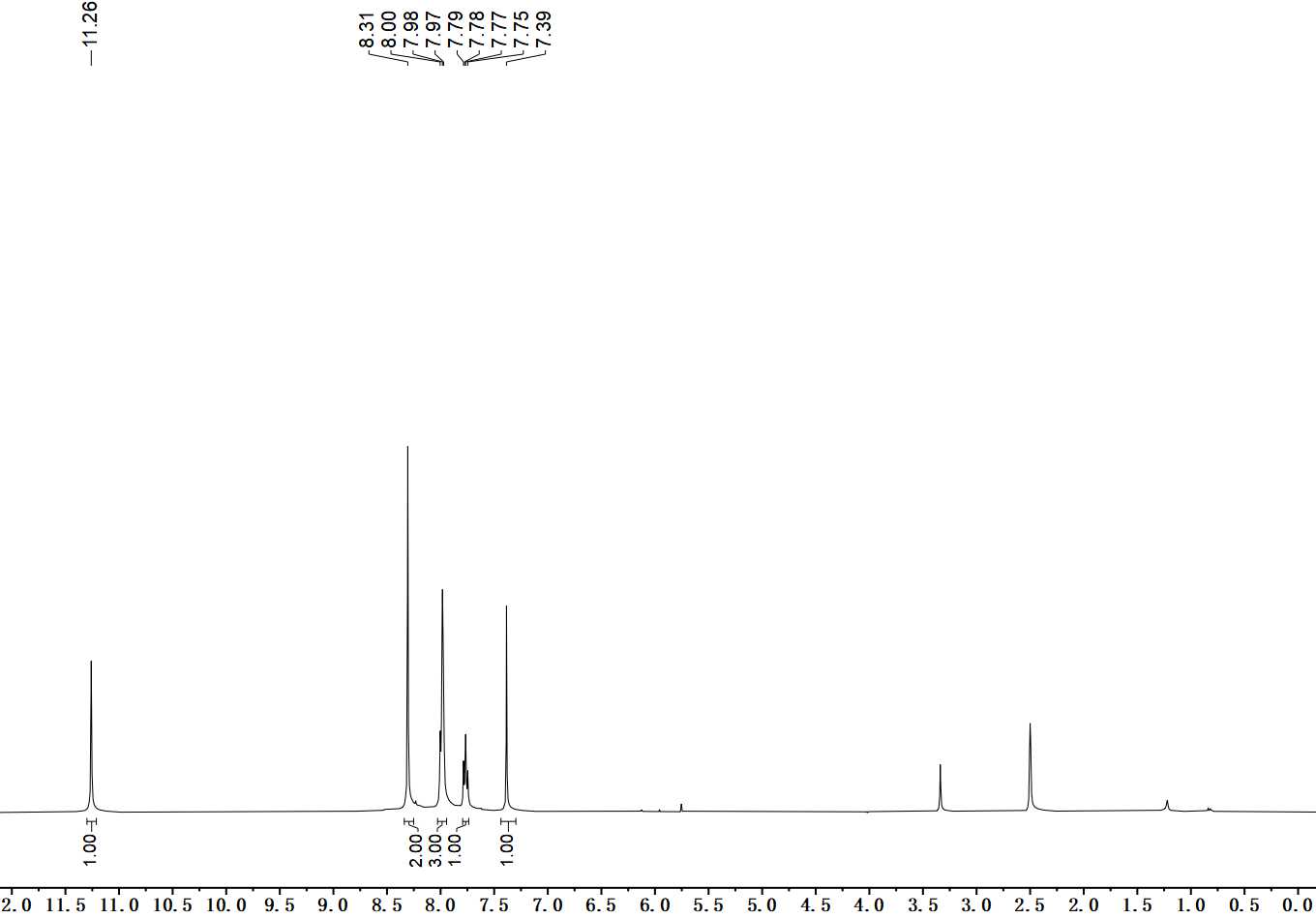
**

^13^C NMR Spectra of **Py-40**

^1^H NMR Spectra of **Py-41**

^13^C NMR Spectra of **Py-41**

^1^H NMR Spectra of **Py-42**

^13^C NMR Spectra of **Py-42**

^1^H NMR Spectra of **Py-43**

^13^C NMR Spectra of **Py-43**

^1^H NMR Spectra of **Py-44**

^13^C NMR Spectra of **Py-44**

^1^H NMR Spectra of **Py-45**

^13^C NMR Spectra of **Py-45**

^1^H NMR Spectra of **Py-27-1**

^13^C NMR Spectra of **Py-27-1**

^1^H NMR Spectra of **Py-27-2**

^1^H NMR Spectra of **Py-27-P**

^13^C NMR Spectra of **Py-27-P**
